# Pharmacogenetic investigation of efficacy response to mepolizumab in eosinophilic granulomatosis with polyangiitis

**DOI:** 10.1007/s00296-020-04523-6

**Published:** 2020-02-03

**Authors:** Lynn D. Condreay, Laura R. Parham, Xiaoyan A. Qu, Jonathan Steinfeld, Michael E. Wechsler, Benjamin A. Raby, Steven W. Yancey, Soumitra Ghosh

**Affiliations:** 1 Genomic Medicine, Parexel International, Durham, NC USA; 2 Genetics, GlaxoSmithKline, Research Triangle Park, NC USA; 3 Computational Biology, GlaxoSmithKline, Research Triangle Park, Raleigh, NC USA; 4 Clinical Development, Respiratory Diseases, GlaxoSmithKline, Upper Providence, PA USA; 5 Department of Medicine, National Jewish Health Cohen Family Asthma Institute, Denver, CO USA; 6 Division of Pulmonary Medicine, Channing Division of Network Medicine, Boston Children’s Hospital, Brigham and Women’s Hospital Harvard Medical School, Boston, MA USA; 7 Medicine Development, GlaxoSmithKline, Research Triangle Park, Raleigh, NC USA; 8 Genetics, GlaxoSmithKline, Upper Providence, PA USA

**Keywords:** Churg–Strauss syndrome, Genetic studies, Complementary therapies, Mepolizumab, Eosinophilic granulomatosis

## Abstract

Treatment of patients with the rare disease eosinophilic granulomatosis with polyangiitis (EGPA) with mepolizumab, a monoclonal antibody to interleukin-5 (IL-5) that reduces blood eosinophil counts, as an add-on therapy to glucocorticoid treatment, results in more accrued weeks in remission, reductions in glucocorticoid use and reductions in relapse rate. However, treatment response varies across a continuum. Therefore, to investigate if large genetic effects could identify responders, the impact of genetic variants on efficacy in EGPA subjects taking mepolizumab and glucocorticoids was assessed in this post hoc study. Using linear regression and a negative binomial model, genetic variant association with three endpoints (accrued duration of remission, average oral glucocorticoid dose, and frequency of relapse) was tested in 61 EGPA subjects dosed with mepolizumab from MIRRA, a phase 3 trial. Candidate gene and genome-wide approaches were used. The candidate gene analysis was designed to investigate drug target effects with eight gene regions selected that were focused on the intersection of the glucocorticoid response (steroidal response) and IL-5 response mechanisms and recognizing potential overlap between EGPA and severe eosinophilic asthma diseases for which mepolizumab is used. The sample size was insufficient to enable testing of rare variants for effects. No genetic variant from either the candidate gene analysis or the GWAS associated with any endpoint. Thresholds to declare significance were *p* < 0.0008 (candidate variant) and *p* < 2.5 × 10^–8^ (genome-wide) analyses. Large genetic effects on mepolizumab-treatment response were not identified which could help differentiate responders from non-responders.

**Electronic supplementary material:**

The online version of this article (10.1007/s00296-020-04523-6) contains supplementary material, which is available to authorized users.

## Short communication

### Background/objective

Eosinophilic granulomatosis with polyangiitis (EGPA; Churg–Strauss Syndrome) is a rare disorder characterized by eosinophilia and vasculitis of one or more organs [1, 2]. EGPA patients receiving mepolizumab, a monoclonal antibody to interleukin-5 (IL-5) that reduces blood eosinophil counts, as an add-on therapy to glucocorticoid treatment, resulted in more accrued weeks in remission, reductions in glucocorticoid use and a reduction in relapse rate [3]. Treatment response varied across a continuum. In the 52 week phase 3 trial evaluating mepolizumab in EGPA patients taking a stable prednisolone or prednisone dose (MIRRA, MEA115921; NCT02020889), 53% of the mepolizumab-treated patients achieved remission compared to 19% of placebo-treated patients [3]. Thus, identification of predictors of response could be valuable for treatment decisions.

Pharmacogenetic (PGx) studies can identify predictors of responses. Such investigations have identified genetic predictors of efficacy with clinically meaningful effects in both candidate gene and unbiased genome-wide association study analyses (GWAS) with more genetic associations identified using endpoints of quantitative continuous biomarker measures of drug response than when using clinical outcomes [4]. Therefore, a PGx study was conducted using candidate gene analysis and GWAS exploring clinical endpoints and a quantitative, continuous measure of drug response.

## Methods

Genetic association with response to mepolizumab was tested using data from MIRRA [3]. MIRRA was conducted in accordance with the ethical principles of the Declaration of Helsinki, the International Council on Harmonisation Good Clinical Practice guidelines, and applicable country specific regulations. Ethics committee/Institutional Review Board approvals were obtained for all 31 sites (e.g., main site: The National Jewish Health IRB Approval number HS-2818, approval date Feb 5, 2019). The intent-to-treat (ITT) study population included 136 EGPA subjects, randomized 1:1 to treatment with mepolizumab or placebo in addition to their baseline glucocorticoids. This exploratory PGx analysis included ITT subjects who provided written informed consent and a blood sample for PGx investigations (*n* = 129). Genotypes were generated using the Affymetrix Axiom Biobank Genotyping Array with custom content v2 (BioStorage Technologies/Bioprocessing Solutions Alliance, Piscataway, NJ, USA). Haplotype reference data from the 1000 Genomes phase 3 project [5] was used to impute over 7 million variants across the genome including HLA variants imputed using HIBAG [6] for association analyses. Genotype and clinical data were available for 116 subjects following quality control analyses. Of these 116 subjects, 61 subjects treated with mepolizumab composed the mepolizumab genetics analysis population (GAP); 55 subjects randomized to placebo were not included in this genetic analysis.

Three efficacy endpoints were evaluated for genetic association: (i) accrued duration of remission defined as the accrued number of weeks where a subject achieved a Birmingham Vasculitis Activity Score (BVAS) score of 0 and a prednisolone/prednisone dose of ≤ 4 mg/day over the 52 week study treatment period; (ii) average oral glucocorticoid (OGC; prednisolone/prednisone) daily dose during the last 4 weeks of the study treatment period (weeks 48–52), and (iii) frequency of EGPA relapse. EGPA relapse was defined as worsening or persistence of active disease since the last visit. This was characterized by active vasculitis (BVAS > 0) or active asthma symptoms and/or signs with a corresponding worsening in the Asthma Control Questionnaire (ACQ-6) score or active nasal and/or sinus disease with a worsening in at least one of the sino-nasal symptom questions. To meet criteria for a relapse, increase in disease activity needed to warrant an increased dose of OGC (or other systemic glucocorticoid therapy) to > 4 mg/day prednisolone or an increased dose or addition of immunosuppressive therapy or hospitalization related to EGPA worsening.

**Fig. 1:**
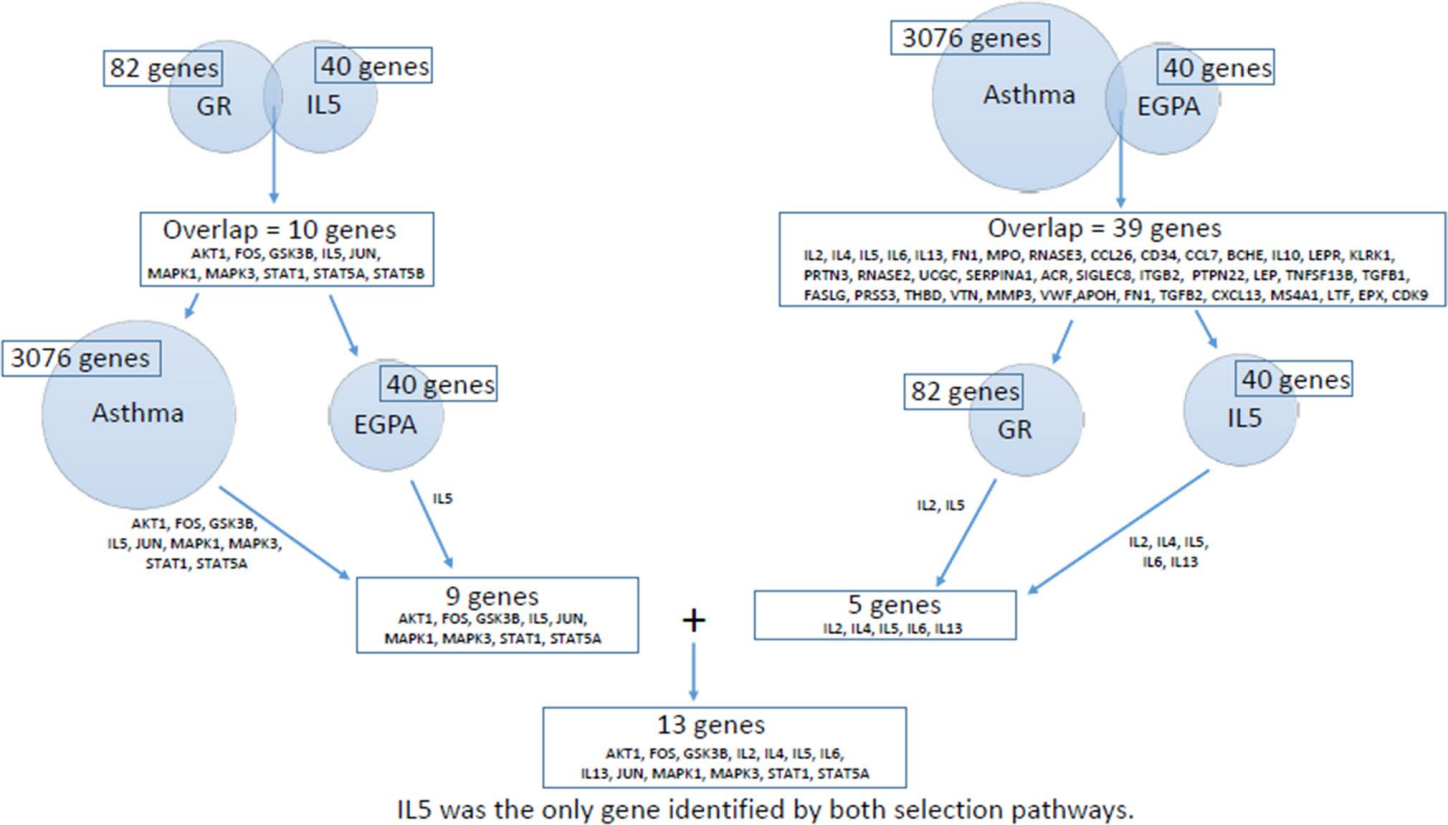
Key pathway and disease association overlap for candidate gene selection. Candidate gene analysis was designed to investigate drug target effects focusing on genes identified in both target pathways (glucocorticoid receptor and IL-5) and in relevant diseases (EGPA and asthma). To enhance power, the number of genes to be evaluated in candidate gene analysis was reduced from > 3000 to 13 with *IL5* identified as common to both selection pathways

Two analytical approaches were used: a candidate gene variant analysis and a genome-wide association study (GWAS). The candidate gene analysis was designed to investigate drug target effects. Gene selection recognized that both glucocorticoids and mepolizumab are used for the treatment of asthma [7-9] and that the primary treatments for EGPA in MIRRA involved an intersection of the glucocorticoid response (steroidal response) and IL-5 response mechanisms. Databases including Open Target Platform (https://www.targetvalidation.org/, [10]), GWAS (https://www.ebi.ac.uk/gwas/, [11]), NCBI BioSystems [12], and literature were mined to select key genes involved in IL-5 and glucocorticoid receptor signaling pathways and genes associated with the eosinophil-related diseases of interest, EGPA and asthma. Candidate genes were involved in either the intersection of IL-5 and the glucocorticoid receptor regulatory signaling pathways and either asthma- or EGPA-associated genes or the intersection of asthma- and EGPA-associated genes and either IL-5 or glucocorticoid receptor regulatory signaling pathways (see Fig. 1 with all genes listed in Supplemental Table 1A, B and Supplemental Table 2A, B).

Thirteen genes were identified: *IL5*, *AKT1*, *FOS*, *GSK3B*, *JUN*, *MAPK1*, *MAPK3*, *STAT1*, *STAT5A*, *IL13*, *IL2*, *IL4*, and *IL6*. Variants representing these genes had evidence of association with the relevant disease or trait by GWAS Catalog [11] and STOPGAP database [13]. Additional variants with no prior evidence of association with any relevant disease or trait with potential deleterious effects in exons of candidate genes from the pathway analysis were identified using a separate tool, SIFT [14] in the ExAC database (https://exac.broadinstitute.org/). Analysis was limited to genetic variants having a minor allele frequency (MAF) of ≥ 0.05 and an imputation *r*^2^ ≥ 0.3. This further restricted the candidate gene list as no variants met the selection criteria for the pathway genes *JUN*, *MAPK1*, *MAPK3*, *STAT1*, or *IL6*. The final candidate variant list included 30 variants from 8 genes (Supplemental Table 3). All other variants in these genes were included in the GWAS.

For each endpoint, two models were assessed, one with and one without clinical covariates significantly associated with the endpoint under investigation. Covariates evaluated included: age, gender, and baseline measures (prednisolone/prednisone daily dose, BVAS, eosinophil count, vasculitis damage index (VDI), historic anti-neutrophil cytoplasmic antibodies (ANCA status), immunosuppressive therapy usage, and duration of EGPA disease). All models included the first two genetic ancestry principal components to correct for potential confounding from population stratification.

The association analysis used a linear model for continuous endpoints (accrued duration of remission and average OGC dose). In models without clinical covariates, endpoints were inverse normal transformed prior to analysis. In models including clinical covariates, after adjusting for relevant covariates, residuals were inverse normal transformed for analysis. Frequency of EGPA relapse was analyzed using a negative binomial model.

An analysis wide type 1 (false positive) error rate of 0.05 was maintained accounting for number of common genetic variants analyzed. Pre-specified significance thresholds were *p* < 0.0008 (*p* < 0.025/number of variants analyzed) for the candidate variant analysis and *p* ≤ 2.5 × 10^–8^ for the GWAS. No corrections were made for multiple endpoints and models. Genomic control adjustments were applied to control for test statistic inflation in testing for genetic main effects.

## Results

**Fig. 2:**
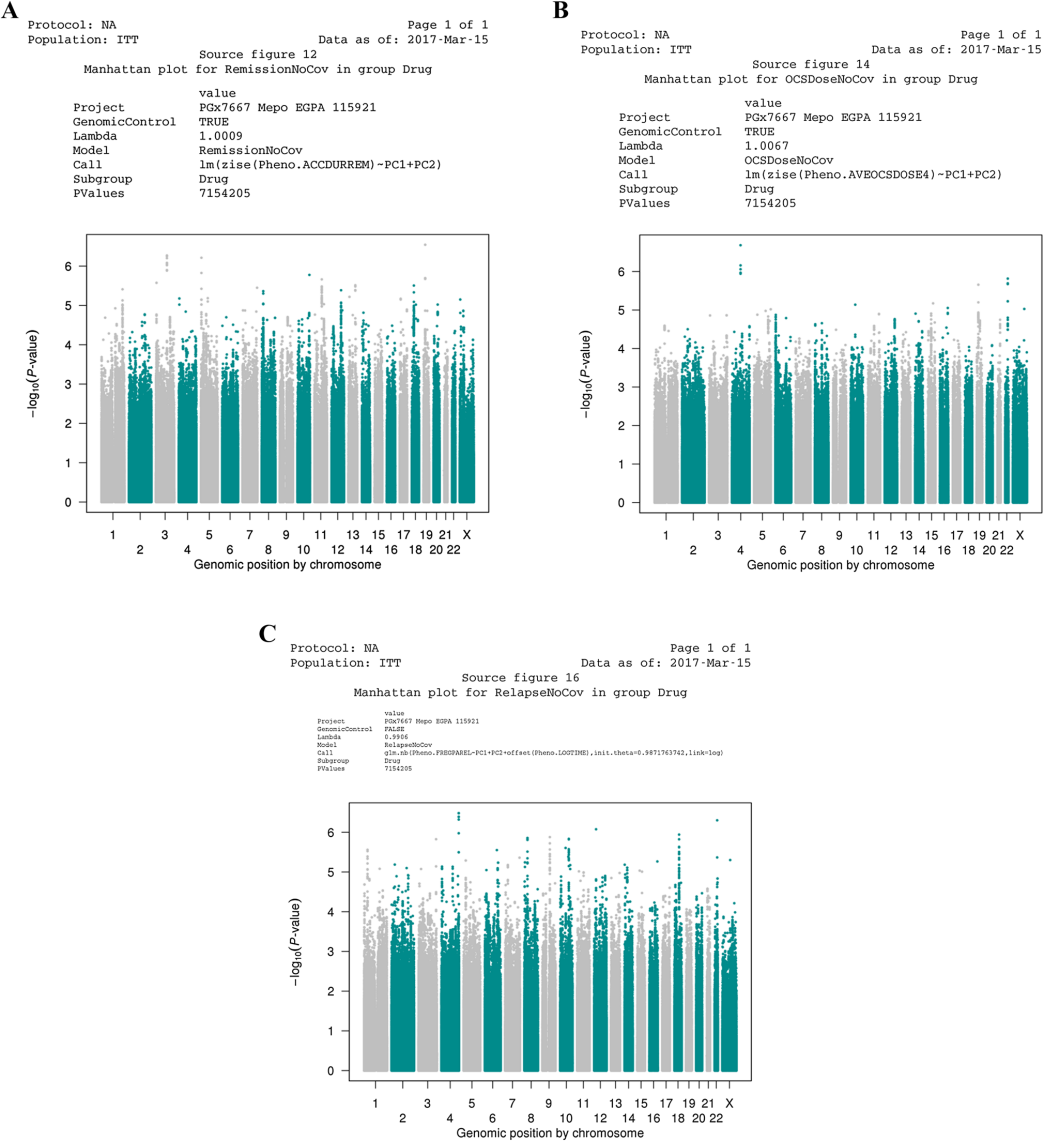
No genetic variant associated with any endpoint in genome-wide analysis. **a** No association with accrued duration of remission. **b** No association with average OGC daily dose during the last 4 weeks of the study treatment period. **c** No association with frequency of EGPA relapse. Manhattan plots of *p*-values obtained testing association with selected endpoints versus genomic position in analysis including significant covariates. All *p*-values were greater than the threshold to declare significance, 2.5 × 10^–8^, for all analyses. **a** No genetic variant was significantly associated with the endpoint of accrued duration of remission. **b** No significant association was identified with the endpoint, average OGC daily dose during the last 4 weeks of the study treatment period. **c** No significant association was identified with the endpoint frequency of EGPA relapse

Demographic characteristics were similar between the intent-to-treat and genetic analysis study populations (Table 1). Four of nine covariates evaluated in the GAP associated with at least one endpoint. Baseline prednisolone/prednisone daily dose associated with all three endpoints tested (Table 2). For the candidate variant approach, the power is ~ 80% to detect effect size of 0.8 assuming a MAF > 0.3. For the GWAS, this study had approximately 25% power to detect effect size > 0.95 assuming variants with a MAF > 0.3. No genetic variant was associated, with or without covariates in analysis, with accrued duration of remission, average OGC Dose during last 4 weeks, or frequency of relapse for either the candidate variant analysis (Data not shown) or the GWAS (see Fig. 2 and Supplemental Table 4, Analysis with no covariates shown, data not shown for analysis with covariates; although no variants met the thresholds to declare significant effect on any endpoint, variants included in the GWAS are identified in Supplemental Table 4 if the *p* value for effect on any one endpoint achieved a *p* value of < 10^–4^).

**Table 1:** Demographic characteristics

Characteristics	ITT^a^ placebo	ITT^a^ mepolizumab	Placebo GAP	Mepolizumab GAP
Total *N*	68	68	55	61
Sex, female (% female)	38 (56%)	42 (62%)	32 (58%)	38 (62%)
Mean age in years (median: min^c^, max^d^)	48.2 (51: 22, 71)	48.7 (50.5: 20, 71)	48.1 (51: 22, 71)	49.4 (51: 20, 71)
< 65 years (%)	59 (87%)	60 (88%)	47 (85%)	53 (87%)

^a^*ITT* intent-to-treat

^b^*GAP* genetics analysis population

^c^*Min* minimum

^d^*Max* maximum

**Table 2:** Covariates associated with endpoints in the mepolizumab genetic analysis population (GAP)

PGx variable description^a^	Accrued duration of remission, *p* value	Average OGC^b^ dose during last 4 weeks, *p* value	Frequency of EGPA^c^ relapse, *p* value
Baseline prednisolone/prednisone daily dose	8.10 × 10^–5^	1.50 × 10^–3^	4.70 × 10^–3^
Baseline birmingham vasculitis activity score (BVAS)	4.51 × 10^–2^	0.60	1.50 × 10^–3^
Baseline vasculitis damage index (VDI), categorical (< 5 vs. ≥ 5)	0.54	0.39	0.02
Baseline vasculitis damage index (VDI), continuous	0.63	0.16	1.00 × 10^–3^
Historic anti-neutrophil cytoplasmic antibodies (ANCA)	0.19	0.47	0.05

(*n *= 61), *p* < 0.05

^a^Potential clinical covariates with no evidence of association with any endpoint included: age [categorical (< 65 or > 65) or continuous], sex, baseline eosinophil count [categorical (< 0.150 GI/L vs. ≥ 0.150 GI/L) or continuous], concomitant immunosuppressive therapy usage, duration of EGPA relapse [categorical (≤ 4 years vs. > 4 years) or continuous]

^b^*OGC* oral glucocorticoid

^c^*EGPA* eosinophilic granulomatosis with polyangiitis

## Discussion

PGx studies can identify predictors of efficacy with large effects in small studies. In this investigation into genetic effects on mepolizumab efficacy in subjects with EGPA, a candidate gene analysis focusing on the intersections of drug target pathways and disease and a GWAS were used. No genetic variant influenced mepolizumab-treatment efficacy in either analysis.

Due to the challenge of enrolling patients with rare diseases, sample size presents limitations for PGx analyses. Though small in subject numbers when compared to many clinical studies in more common diseases, MIRRA was the largest placebo-controlled trial ever performed in EGPA. The small sample size for this PGx study restricted the power of this study to only be able to identify common variants with very large effects. GWAS are designed to identify common variants; we cannot exclude the presence of rare coding variants with substantially larger pharmacogenetic effects.

In addition, EGPA is heterogeneous with multiple presentations. Genetic effects may differ across subtypes but sample sizes may be too small to effectively investigate differences in genetic association between subtypes. Effects from treatments beside mepolizumab may have obfuscated potential PGx effects. The effect of glucocorticoid treatment may have confounded the study as subjects were receiving drug or placebo and simultaneously tapering glucocorticoid doses. Concomitant treatment with immunosuppressants in some patients may have had a similar effect (although dose adjustment was not permitted). Thus, challenges to analysis may reflect challenges of EGPA.

In summary, as in a recent investigation into genetic effects on mepolizumab response in severe asthma [15], this investigation into PGx effects on the efficacy of mepolizumab as an add-on treatment did not detect any genetic effects likely to impact clinical outcomes.

## Electronic supplementary material

Below is the link to the electronic supplementary material.

Supplementary file1 (PDF 2725 kb)
