# Supplementary material for: Pharmacogenetic investigation of efficacy response to mepolizumab in eosinophilic granulomatosis with polyangiitis
Source: Rheumatol Int. 2020 Feb 3;40(8):1301–7. doi: 10.1007/s00296-020-04523-6 (PMC7316687; doi:10.1007/s00296-020-04523-6)
Supplement: Supplementary file 1 — Supplementary file1 (PDF 2725 kb) [file 296_2020_4523_MOESM1_ESM.pdf]

SUPPLEMENTAL TABLE 1. Disease Associated Genes: A) EGPA associated genes

**Symbol**	**Gene Name**
IL5	interleukin 5
MPO	myeloperoxidase
RNASE3	ribonuclease A family member 3
CCL26	C-C motif chemokine ligand 26
CD34	CD34 molecule
IL2	interleukin 2
CCL17	C-C motif chemokine ligand 17
BCHE	butyrylcholinesterase
IL10	interleukin 10
LEPR	leptin receptor
KLRK1	killer cell lectin like receptor K1
IL6	interleukin 6
PRTN3	proteinase 3
IL13	interleukin 13
IL4	interleukin 4
RNASE2	ribonuclease A family member 2
UGCG	UDP-glucose ceramide glucosyltransferase
SERPINA1	serpin family A member 1
ACR	acrosin
SIGLEC8	sialic acid binding Ig like lectin 8
SSRP1	structure specific recognition protein 1
ITGB2	integrin subunit beta 2

PTPN22	protein tyrosine phosphatase, non-receptor type 22
LEP	leptin
TNFSF13B	tumor necrosis factor superfamily member 13b
TGFB1	transforming growth factor beta 1
FASLG	Fas ligand
PRSS3	protease, serine 3
THBD	thrombomodulin
VTN	vitronectin
MMP3	matrix metallopeptidase 3
VWF	von Willebrand factor
APOH	apolipoprotein H
FN1	fibronectin 1
TGFB2	transforming growth factor beta 2
CXCL13	C-X-C motif chemokine ligand 13
MS4A1	membrane spanning 4-domains A1
LTF	lactotransferrin
EPX	eosinophil peroxidase
CDK9	cyclin dependent kinase 9

SUPPLEMENTAL TABLE 1. Disease Associated Genes: B) Asthma associated genes

**Symbol**	**Gene Name**
PDE4D	phosphodiesterase 4D
ORMDL3	ORMDL sphingolipid biosynthesis regulator 3
PTGDR	prostaglandin D2 receptor
PTGS2	prostaglandin-endoperoxide synthase 2
ALOX5	arachidonate 5-lipoxygenase
IL5	interleukin 5
CHI3L1	chitinase 3 like 1
ADRB2	adrenoceptor beta 2
ADAM33	ADAM metallopeptidase domain 33
NR3C1	nuclear receptor subfamily 3 group C member 1
NPSR1	neuropeptide S receptor 1
PLA2G7	phospholipase A2 group VII
PTGS1	prostaglandin-endoperoxide synthase 1
VDR	vitamin D (1,25- dihydroxyvitamin D3) receptor
CYSLTR1	cysteinyl leukotriene receptor 1
MUC7	mucin 7, secreted
DPP10	dipeptidyl peptidase like 10
IRAK3	interleukin 1 receptor associated kinase 3
HRH1	histamine receptor H1
ADRB1	adrenoceptor beta 1
CHRM3	cholinergic receptor muscarinic 3
DENND1B	DENN domain containing 1B
PDE4B	phosphodiesterase 4B

ADRA1B	adrenoceptor alpha 1B
IGHE	immunoglobulin heavy constant epsilon
ADRB3	adrenoceptor beta 3
ADORA2B	adenosine A2b receptor
ADRA2A	adrenoceptor alpha 2A
PDE4A	phosphodiesterase 4A
CHRNB2	cholinergic receptor nicotinic beta 2 subunit
ADRA1A	adrenoceptor alpha 1A
ADRA1D	adrenoceptor alpha 1D
ADORA3	adenosine A3 receptor
PDE3A	phosphodiesterase 3A
PDE4C	phosphodiesterase 4C
ATP4A	ATPase H+/K+ transporting alpha subunit
ATP4B	ATPase H+/K+ transporting beta subunit
PDE3B	phosphodiesterase 3B
CHRNA4	cholinergic receptor nicotinic alpha 4 subunit
ADRA2C	adrenoceptor alpha 2C
ADORA2A	adenosine A2a receptor
ADRA2B	adrenoceptor alpha 2B
ADORA1	adenosine A1 receptor
PTGDR2	prostaglandin D2 receptor 2
GSDMB	gasdermin B
HMGCR	3-hydroxy-3-methylglutaryl-CoA reductase
GSDMA	gasdermin A
TSLP	thymic stromal lymphopoietin

IL1RL1	interleukin 1 receptor like 1
PDGFRB	platelet derived growth factor receptor beta
KIT	KIT proto-oncogene receptor tyrosine kinase
PDGFRA	platelet derived growth factor receptor alpha
FGFR3	fibroblast growth factor receptor 3
TRPV1	transient receptor potential cation channel subfamily V member 1
FAAH	fatty acid amide hydrolase
DHFR	dihydrofolate reductase
CRB1	crumbs 1, cell polarity complex component
IL33	interleukin 33
HLA-DQB1	major histocompatibility complex, class II, DQ beta 1
CDHR3	cadherin related family member 3
PBX2	PBX homeobox 2
C6orf10	chromosome 6 open reading frame 10
IL18R1	interleukin 18 receptor 1
HLA-DRA	major histocompatibility complex, class II, DR alpha
IKZF4	IKAROS family zinc finger 4
TLR1	toll like receptor 1
SLC30A8	solute carrier family 30 member 8
GATA3	GATA binding protein 3
GPSM3	G-protein signaling modulator 3
SMAD3	SMAD family member 3
CXCL11	C-X-C motif chemokine ligand 11
PMEL	premelanosome protein
CDK2	cyclin dependent kinase 2

WDR36	WD repeat domain 36
HLA-DPA1	major histocompatibility complex, class II, DP alpha 1
IL13	interleukin 13
USP38	ubiquitin specific peptidase 38
CXCL9	C-X-C motif chemokine ligand 9
HLA-DQA2	major histocompatibility complex, class II, DQ alpha 2
PYHIN1	pyrin and HIN domain family member 1
CCL5	C-C motif chemokine ligand 5
IL6R	interleukin 6 receptor
IFNL1	interferon lambda 1
IL2RB	interleukin 2 receptor subunit beta
CLEC16A	C-type lectin domain family 16 member A
IFNL2	interferon lambda 2
RAD50	RAD50 double strand break repair protein
IFNL3	interferon lambda 3
RORA	RAR related orphan receptor A
GAB1	GRB2 associated binding protein 1
HLA-DQA1	major histocompatibility complex, class II, DQ alpha 1
ZBP1	Z-DNA binding protein 1
RSAD2	radical S-adenosyl methionine domain containing 2
PPARG	peroxisome proliferator activated receptor gamma
SMPD1	sphingomyelin phosphodiesterase 1
SLC22A5	solute carrier family 22 member 5
IFIT3	interferon induced protein with tetratricopeptide repeats 3
LGALS17A	Charcot-Leyden crystal protein pseudogene

CXCR2	C-X-C motif chemokine receptor 2
EDNRA	endothelin receptor type A
TNFSF13B	tumor necrosis factor superfamily member 13b
ZBTB10	zinc finger and BTB domain containing 10
IKZF3	IKAROS family zinc finger 3
IRF1	interferon regulatory factor 1
EDNRB	endothelin receptor type B
CXCR1	C-X-C motif chemokine receptor 1
RAP1GAP2	RAP1 GTPase activating protein 2
BRD2	bromodomain containing 2
DDX58	DEXD/H-box helicase 58
XKR6	XK related 6
IL4I1	interleukin 4 induced 1
KCNN4	potassium calcium-activated channel subfamily N member 4
VAV3	vav guanine nucleotide exchange factor 3
TNS1	tensin 1
PRKG1	protein kinase, cGMP-dependent, type I
APOBEC3A	apolipoprotein B mRNA editing enzyme catalytic subunit 3A
SOCS1	suppressor of cytokine signaling 1
BTNL2	butyrophilin like 2
ERBB4	erb-b2 receptor tyrosine kinase 4
C5orf56	chromosome 5 open reading frame 56
HERC5	HECT and RLD domain containing E3 ubiquitin protein ligase 5
LRRC32	leucine rich repeat containing 32
IFIH1	interferon induced with helicase C domain 1

IDO1	indoleamine 2,3-dioxygenase 1
HTR2A	5-hydroxytryptamine receptor 2A
BATF2	basic leucine zipper ATF-like transcription factor 2
ETV7	ETS variant 7
RBM17	RNA binding motif protein 17
SAMD9L	sterile alpha motif domain containing 9 like
ISG15	ISG15 ubiquitin-like modifier
FGD2	FYVE, RhoGEF and PH domain containing 2
FAM19A2	family with sequence similarity 19 member A2, C-C motif chemokine like
LAG3	lymphocyte activating 3
DDX60L	DEAD-box helicase 60-like
MX2	MX dynamin like GTPase 2
MMP13	matrix metallopeptidase 13
COMMD10	COMM domain containing 10
ACO1	aconitase 1
ZNF665	zinc finger protein 665
MX1	MX dynamin like GTPase 1
IL17A	interleukin 17A
SOCS3	suppressor of cytokine signaling 3
IL17C	interleukin 17C
KCNQ4	potassium voltage-gated channel subfamily Q member 4
CD274	CD274 molecule
S1PR3	sphingosine-1-phosphate receptor 3
C6orf118	chromosome 6 open reading frame 118
TMEM232	transmembrane protein 232

EPSTI1	epithelial stromal interaction 1 (breast)
GMPR	guanosine monophosphate reductase
ISG20	interferon stimulated exonuclease gene 20
CD86	CD86 molecule
CD80	CD80 molecule
SEMA3D	semaphorin 3D
PTHLH	parathyroid hormone like hormone
HRH4	histamine receptor H4
P2RY12	purinergic receptor P2Y12
OAS1	2'-5'-oligoadenylate synthetase 1
TMEM171	transmembrane protein 171
S1PR1	sphingosine-1-phosphate receptor 1
RAB11FIP2	RAB11 family interacting protein 2
CMPK2	cytidine/uridine monophosphate kinase 2
GBP1P1	guanylate binding protein 1 pseudogene 1
IFI35	interferon induced protein 35
USP18	ubiquitin specific peptidase 18
OAS3	2'-5'-oligoadenylate synthetase 3
GBP5	guanylate binding protein 5
SYK	spleen associated tyrosine kinase
EDIL3	EGF like repeats and discoidin domains 3
CRCT1	cysteine rich C-terminal 1
DUOXA2	dual oxidase maturation factor 2
HELZ2	helicase with zinc finger 2
RBM7	RNA binding motif protein 7

MAP3K13	mitogen-activated protein kinase kinase kinase 13
IFNAR1	interferon alpha and beta receptor subunit 1
S1PR2	sphingosine-1-phosphate receptor 2
S1PR5	sphingosine-1-phosphate receptor 5
LIFR	leukemia inhibitory factor receptor alpha
MMADHC	methylmalonic aciduria and homocystinuria, cblD type
SLC15A3	solute carrier family 15 member 3
TRANK1	tetratricopeptide repeat and ankyrin repeat containing 1
NCF1	neutrophil cytosolic factor 1
TAP1	transporter 1, ATP binding cassette subfamily B member
IFNAR2	interferon alpha and beta receptor subunit 2
MYCN	v-myc avian myelocytomatosis viral oncogene neuroblastoma derived homolog
FDPS	farnesyl diphosphate synthase
S1PR4	sphingosine-1-phosphate receptor 4
ABL1	ABL proto-oncogene 1, non-receptor tyrosine kinase
MAPK14	mitogen-activated protein kinase 14
FGFR2	fibroblast growth factor receptor 2
PDCD1LG2	programmed cell death 1 ligand 2
CD38	CD38 molecule
GBP1	guanylate binding protein 1
THEMIS2	thymocyte selection associated family member 2
BCL2A1	BCL2 related protein A1
IRF7	interferon regulatory factor 7
NLRC5	NLR family CARD domain containing 5
BRIP1	BRCA1 interacting protein C-terminal helicase 1

SAMD9	sterile alpha motif domain containing 9
OAS2	2'-5'-oligoadenylate synthetase 2
STAT1	signal transducer and activator of transcription 1
NOS2	nitric oxide synthase 2
CLDN14	claudin 14
RANBP6	RAN binding protein 6
HSH2D	hematopoietic SH2 domain containing
IL19	interleukin 19
C19orf66	chromosome 19 open reading frame 66
BST2	bone marrow stromal cell antigen 2
IL36G	interleukin 36, gamma
PLAUR	plasminogen activator, urokinase receptor
NDFIP1	Nedd4 family interacting protein 1
IL23A	interleukin 23 subunit alpha
DDX60	DEXD/H-box helicase 60
LMO2	LIM domain only 2
APOBEC3G	apolipoprotein B mRNA editing enzyme catalytic subunit 3G
PLA2G4E	phospholipase A2 group IVE
MLKL	mixed lineage kinase domain like
IFI44L	interferon induced protein 44 like
SPRR2F	small proline rich protein 2F
HERC6	HECT and RLD domain containing E3 ubiquitin protein ligase family member 6
XAF1	XIAP associated factor 1
SP110	SP110 nuclear body protein
APOL3	apolipoprotein L3

IFITM1	interferon induced transmembrane protein 1
SCG3	secretogranin III
LAMP3	lysosomal associated membrane protein 3
URI1	URI1, prefoldin like chaperone
CX3CL1	C-X3-C motif chemokine ligand 1
GCH1	GTP cyclohydrolase 1
IL18BP	interleukin 18 binding protein
FRMD3	FERM domain containing 3
PPM1K	protein phosphatase, Mg2+/Mn2+ dependent 1K
NT5C3A	5'-nucleotidase, cytosolic IIIA
RASGRP3	RAS guanyl releasing protein 3
CEACAM1	carcinoembryonic antigen related cell adhesion molecule 1
SECTM1	secreted and transmembrane 1
CASP1	caspase 1
IL15RA	interleukin 15 receptor subunit alpha
RNF213	ring finger protein 213
PIK3AP1	phosphoinositide-3-kinase adaptor protein 1
DUOX2	dual oxidase 2
IFI44	interferon induced protein 44
TGFB2	transforming growth factor beta 2
TGFB1	transforming growth factor beta 1
APOBEC3B	apolipoprotein B mRNA editing enzyme catalytic subunit 3B
SERPINB9P1	serpin family B member 9 pseudogene 1
PLEKHA4	pleckstrin homology domain containing A4
NUB1	negative regulator of ubiquitin like proteins 1

RTP4	receptor transporter protein 4
NUPR1	nuclear protein 1, transcriptional regulator
KLHDC7B	kelch domain containing 7B
CXCL8	C-X-C motif chemokine ligand 8
SERPINB9	serpin family B member 9
TLR3	toll like receptor 3
DST	dystonin
CFP	complement factor properdin
CYP2J2	cytochrome P450 family 2 subfamily J member 2
MAVS	mitochondrial antiviral signaling protein
IL1RN	interleukin 1 receptor antagonist
TLX1NB	TLX1 neighbor
TAGAP	T-cell activation RhoGTPase activating protein
ZNF618	zinc finger protein 618
TMEM92	transmembrane protein 92
APOL2	apolipoprotein L2
CLEC7A	C-type lectin domain family 7 member A
LEP	leptin
SLC25A28	solute carrier family 25 member 28
CTA-384D8.31	
TRIM22	tripartite motif containing 22
UBE2L6	ubiquitin conjugating enzyme E2 L6
PARP12	poly(ADP-ribose) polymerase family member 12
MMRN1	multimerin 1

BIRC3	baculoviral IAP repeat containing 3
LGALS9	galectin 9
IFI6	interferon alpha inducible protein 6
JAK2	Janus kinase 2
STAT2	signal transducer and activator of transcription 2
IL6	interleukin 6
PLA2G10	phospholipase A2 group X
ITGB6	integrin subunit beta 6
TYMP	thymidine phosphorylase
TRIM21	tripartite motif containing 21
BCL2L14	BCL2 like 14
DHX58	DEXH-box helicase 58
ADAM8	ADAM metallopeptidase domain 8
LBP	lipopolysaccharide binding protein
AIM2	absent in melanoma 2
NCF1B	neutrophil cytosolic factor 1B pseudogene
STARD5	StAR related lipid transfer domain containing 5
TRIB2	tribbles pseudokinase 2
LRRC3C	leucine rich repeat containing 3C
IL18	interleukin 18
SFTPD	surfactant protein D
TNFAIP3	TNF alpha induced protein 3
TRIM5	tripartite motif containing 5
STARD4	StAR related lipid transfer domain containing 4
ZNFX1	zinc finger NFX1-type containing 1

EREG	epiregulin
MXD1	MAX dimerization protein 1
LIF	leukemia inhibitory factor
IL1A	interleukin 1 alpha
FGF20	fibroblast growth factor 20
ECSCR	endothelial cell surface expressed chemotaxis and apoptosis regulator
CMYA5	cardiomyopathy associated 5
NFKBIA	NFKB inhibitor alpha
CRLF2	cytokine receptor-like factor 2
SYNPO2	synaptopodin 2
PARP10	poly(ADP-ribose) polymerase family member 10
ENDOD1	endonuclease domain containing 1
PMAIP1	phorbol-12-myristate-13-acetate-induced protein 1
APOBEC3F	apolipoprotein B mRNA editing enzyme catalytic subunit 3F
PSMB9	proteasome subunit beta 9
HLA-F	major histocompatibility complex, class I, F
POU2F3	POU class 2 homeobox 3
GALM	galactose mutarotase
RP11-400K9.4	
KLHL6	kelch like family member 6
CSRNP1	cysteine and serine rich nuclear protein 1
TAP2	transporter 2, ATP binding cassette subfamily B member
PNPT1	polyribonucleotide nucleotidyltransferase 1
MT2A	metallothionein 2A

TLR2	toll like receptor 2
GRIN3A	glutamate ionotropic receptor NMDA type subunit 3A
CNP	2',3'-cyclic nucleotide 3' phosphodiesterase
SPATS2L	spermatogenesis associated serine rich 2 like
SP100	SP100 nuclear antigen
PRICKLE4	prickle planar cell polarity protein 4
OPTN	optineurin
PARP9	poly(ADP-ribose) polymerase family member 9
TMEM106A	transmembrane protein 106A
TOR1B	torsin family 1 member B
SRY	sex determining region Y
IL10	interleukin 10
POSTN	periostin
RIPK2	receptor interacting serine/threonine kinase 2
FAM46A	family with sequence similarity 46 member A
RETN	resistin
IL2	interleukin 2
DDIAS	DNA damage induced apoptosis suppressor
IL1B	interleukin 1 beta
RBM11	RNA binding motif protein 11
CCL22	C-C motif chemokine ligand 22
ADCY4	adenylate cyclase 4
CIC	capicua transcriptional repressor
UCKL1-AS1	UCKL1 antisense RNA 1
RNASE3	ribonuclease A family member 3

HMGB1	high mobility group box 1
CCL11	C-C motif chemokine ligand 11
DUSP5	dual specificity phosphatase 5
NMI	N-myc and STAT interactor
TRIM38	tripartite motif containing 38
IFI27	interferon alpha inducible protein 27
HRASLS2	HRAS like suppressor 2
PCGF5	polycomb group ring finger 5
STOML1	stomatin like 1
BLZF1	basic leucine zipper nuclear factor 1
MASTL	microtubule associated serine/threonine kinase like
IFITM3	interferon induced transmembrane protein 3
RP11-638I2.9	
RP11-288L9.4	
PARP14	poly(ADP-ribose) polymerase family member 14
PLA2G4A	phospholipase A2 group IVA
CACNA1I	calcium voltage-gated channel subunit alpha1 I
MYD88	myeloid differentiation primary response 88
ACE2	angiotensin I converting enzyme 2
EMP1	epithelial membrane protein 1
IKBKE	inhibitor of kappa light polypeptide gene enhancer in B-cells, kinase epsilon
ALOX15B	arachidonate 15-lipoxygenase, type B
CH25H	cholesterol 25-hydroxylase

TICAM1	toll like receptor adaptor molecule 1
NEURL3	neuralized E3 ubiquitin protein ligase 3
MMP9	matrix metallopeptidase 9
SCO2	SCO2 cytochrome c oxidase assembly protein
MRGPRX3	MAS related GPR family member X3
CSF2	colony stimulating factor 2
APOL6	apolipoprotein L6
EHD4	EH domain containing 4
RNF114	ring finger protein 114
LAP3	leucine aminopeptidase 3
CLUHP3	clustered mitochondria homolog pseudogene 3
APOL1	apolipoprotein L1
GBP2	guanylate binding protein 2
TRIM25	tripartite motif containing 25
TRIM14	tripartite motif containing 14
TGM1	transglutaminase 1
SRGAP3-AS2	SRGAP3 antisense RNA 2
KRT6B	keratin 6B
ZFYVE26	zinc finger FYVE-type containing 26
MCUB	mitochondrial calcium uniporter dominant negative beta subunit
EIF2AK2	eukaryotic translation initiation factor 2 alpha kinase 2
IRAK2	interleukin 1 receptor associated kinase 2
CHST9	carbohydrate sulfotransferase 9
KITLG	KIT ligand
ICAM1	intercellular adhesion molecule 1

TBXA2R	thromboxane A2 receptor
GSTM1	glutathione S-transferase mu 1
CTLA4	cytotoxic T-lymphocyte associated protein 4
AKAP6	A-kinase anchoring protein 6
KIF26B	kinesin family member 26B
SBK1	SH3 domain binding kinase 1
PLSCR1	phospholipid scramblase 1
STOML3	stomatin like 3
ADAMTSL3	ADAMTS like 3
MAP1A	microtubule associated protein 1A
NCOA7	nuclear receptor coactivator 7
XRN1	5'-3' exoribonuclease 1
RHBDF2	rhomboid 5 homolog 2
MOV10	Mov10 RISC complex RNA helicase
IFITM2	interferon induced transmembrane protein 2
STX11	syntaxin 11
DTX3L	deltex E3 ubiquitin ligase 3L
TNFAIP2	TNF alpha induced protein 2
IL4	interleukin 4
EDN1	endothelin 1
MUC5AC	mucin 5AC, oligomeric mucus/gel-forming
ZNF804A	zinc finger protein 804A
CTGF	connective tissue growth factor
IFNG	interferon gamma
SERPINE1	serpin family E member 1

CCL2	C-C motif chemokine ligand 2
PPM1J	protein phosphatase, Mg2+/Mn2+ dependent 1J
AGXT	alanine-glyoxylate aminotransferase
SOD2	superoxide dismutase 2, mitochondrial
CD14	CD14 molecule
HESX1	HESX homeobox 1
LTA	lymphotoxin alpha
CCR8	C-C motif chemokine receptor 8
KIAA0101	KIAA0101
ARSA	arylsulfatase A
CMTR1	cap methyltransferase 1
TGM2	transglutaminase 2
PHF11	PHD finger protein 11
ZC3HAV1	zinc finger CCCH-type containing, antiviral 1
TMEM140	transmembrane protein 140
GNB4	G protein subunit beta 4
UBA7	ubiquitin like modifier activating enzyme 7
TRAFD1	TRAF-type zinc finger domain containing 1
LDLRAD1	low density lipoprotein receptor class A domain containing 1
LGMN	legumain
TDRD7	tudor domain containing 7
IFNB1	interferon beta 1
BCL7A	BCL tumor suppressor 7A
SPP1	secreted phosphoprotein 1
VEGFA	vascular endothelial growth factor A

PHLPP2	PH domain and leucine rich repeat protein phosphatase 2
ELOVL7	ELOVL fatty acid elongase 7
IL9	interleukin 9
CLU	clusterin
RNASE2	ribonuclease A family member 2
KRT24	keratin 24
TNFSF14	tumor necrosis factor superfamily member 14
CDKN1A	cyclin dependent kinase inhibitor 1A
GSTP1	glutathione S-transferase pi 1
BDNF	brain derived neurotrophic factor
RIPK3	receptor interacting serine/threonine kinase 3
HLA-E	major histocompatibility complex, class I, E
RUBCN	RUN and cysteine rich domain containing beclin 1 interacting protein
NINJ1	ninjurin 1
CFB	complement factor B
IFIT5	interferon induced protein with tetratricopeptide repeats 5
HHLA2	HERV-H LTR-associating 2
OGFR	opioid growth factor receptor
TRIM69	tripartite motif containing 69
MOB3C	MOB kinase activator 3C
SP140L	SP140 nuclear body protein like
TNIP1	TNFAIP3 interacting protein 1
CPSF4	cleavage and polyadenylation specific factor 4
FOXP3	forkhead box P3
HMOX1	heme oxygenase 1

TLR4	toll like receptor 4
TLR7	toll like receptor 7
IL31	interleukin 31
CCL26	C-C motif chemokine ligand 26
TULP2	tubby like protein 2
IFI16	interferon gamma inducible protein 16
SQRDL	sulfide quinone reductase-like (yeast)
ADH1C	alcohol dehydrogenase 1C (class I), gamma polypeptide
CCL13	C-C motif chemokine ligand 13
HAVCR1	hepatitis A virus cellular receptor 1
MPO	myeloperoxidase
TNFSF4	tumor necrosis factor superfamily member 4
NGF	nerve growth factor
C8orf4	chromosome 8 open reading frame 4
SP6	Sp6 transcription factor
MMP1	matrix metallopeptidase 1
PRKD2	protein kinase D2
BAK1	BCL2 antagonist/killer 1
PCNA	proliferating cell nuclear antigen
CXCL2	C-X-C motif chemokine ligand 2
HERC2	HECT and RLD domain containing E3 ubiquitin protein ligase 2
CARD11	caspase recruitment domain family member 11
TUBA4B	tubulin alpha 4b
BTN3A3	butyrophilin subfamily 3 member A3
C21orf91	chromosome 21 open reading frame 91

SIDT1	SID1 transmembrane family member 1
FBXO6	F-box protein 6
N4BP1	NEDD4 binding protein 1
KIAA1551	KIAA1551
CPEB2	cytoplasmic polyadenylation element binding protein 2
PLEK2	pleckstrin 2
VAMP5	vesicle associated membrane protein 5
PCSK7	proprotein convertase subtilisin/kexin type 7
ATF3	activating transcription factor 3
SLN	sarcolipin
TNFRSF10A	TNF receptor superfamily member 10a
CCR3	C-C motif chemokine receptor 3
CEACAM5	carcinoembryonic antigen related cell adhesion molecule 5
MS4A2	membrane spanning 4-domains A2
SPINK5	serine peptidase inhibitor, Kazal type 5
IL4R	interleukin 4 receptor
EGFR	epidermal growth factor receptor
HPSE2	heparanase 2 (inactive)
DRD4	dopamine receptor D4
DRD2	dopamine receptor D2
DRD3	dopamine receptor D3
DDC	dopa decarboxylase
HTR2C	5-hydroxytryptamine receptor 2C
ANKRD66	ankyrin repeat domain 66
NANOS1	nanos C2HC-type zinc finger 1

CD28	CD28 molecule
CXCL12	C-X-C motif chemokine ligand 12
ABI3BP	ABI family member 3 binding protein
KLF6	Kruppel like factor 6
NPY	neuropeptide Y
PI3	peptidase inhibitor 3
ANGPT2	angiopoietin 2
TIMP1	TIMP metallopeptidase inhibitor 1
PSMB8	proteasome subunit beta 8
FGF2	fibroblast growth factor 2
IL17F	interleukin 17F
HEG1	heart development protein with EGF like domains 1
USP2	ubiquitin specific peptidase 2
PSAP	prosaposin
ARAP2	ArfGAP with RhoGAP domain, ankyrin repeat and PH domain 2
AP5B1	adaptor related protein complex 5 beta 1 subunit
RAB36	RAB36, member RAS oncogene family
TMEM229B	transmembrane protein 229B
TMEM212	transmembrane protein 212
C1GALT1	core 1 synthase, glycoprotein-N-acetylgalactosamine 3-beta-galactosyltransferase 1
JADE2	jade family PHD finger 2
FAM135A	family with sequence similarity 135 member A
C1R	complement C1r
KIAA0040	KIAA0040

SLFN5	schlafen family member 5
CYB5R2	cytochrome b5 reductase 2
CDC25B	cell division cycle 25B
RBCK1	RANBP2-type and C3HC4-type zinc finger containing 1
RP11-356K23.1	
CXCL10	C-X-C motif chemokine ligand 10
CD47	CD47 molecule
HIF1A	hypoxia inducible factor 1 alpha subunit
COL22A1	collagen type XXII alpha 1 chain
SELL	selectin L
IGFBP4	insulin like growth factor binding protein 4
IL32	interleukin 32
AC009948.5	
BTN3A1	butyrophilin subfamily 3 member A1
SOAT1	sterol O-acyltransferase 1
LY6E	lymphocyte antigen 6 complex, locus E
SERPING1	serpin family G member 1
IL22	interleukin 22
LTB4R	leukotriene B4 receptor
C15orf48	chromosome 15 open reading frame 48
ALOX15	arachidonate 15-lipoxygenase
TNIP3	TNFAIP3 interacting protein 3
SLPI	secretory leukocyte peptidase inhibitor
MTFMT	mitochondrial methionyl-tRNA formyltransferase

EPHX1	epoxide hydrolase 1
STAT6	signal transducer and activator of transcription 6
EPX	eosinophil peroxidase
F2RL1	F2R like trypsin receptor 1
GLCCI1	glucocorticoid induced 1
GLRX	glutaredoxin
LTA4H	leukotriene A4 hydrolase
PSMA6	proteasome subunit alpha 6
CD40	CD40 molecule
GPT2	glutamic--pyruvic transaminase 2
MARCKSL1	MARCKS like 1
RNF19B	ring finger protein 19B
PELI1	pellino E3 ubiquitin protein ligase 1
HK2	hexokinase 2
PARP8	poly(ADP-ribose) polymerase family member 8
ADAR	adenosine deaminase, RNA specific
TMTC1	transmembrane and tetratricopeptide repeat containing 1
TBX21	T-box 21
CD34	CD34 molecule
CRP	C-reactive protein, pentraxin-related
CCL17	C-C motif chemokine ligand 17
TNC	tenascin C
IL21	interleukin 21
ALOX5AP	arachidonate 5-lipoxygenase activating protein
TNFRSF8	TNF receptor superfamily member 8

GTPBP1	GTP binding protein 1
HPGDS	hematopoietic prostaglandin D synthase
SELP	selectin P
DPP4	dipeptidyl peptidase 4
TLE4	transducin like enhancer of split 4
CD40LG	CD40 ligand
PTEN	phosphatase and tensin homolog
MBL2	mannose binding lectin 2
CDH1	cadherin 1
IL15	interleukin 15
FEV	FEV, ETS transcription factor
IL17RB	interleukin 17 receptor B
SCGB3A2	secretoglobin family 3A member 2
CSF3	colony stimulating factor 3
IL12B	interleukin 12B
CTD-3128G10.7	
LTC4S	leukotriene C4 synthase
TCHH	trichohyalin
ZFP36	ZFP36 ring finger protein
AREG	amphiregulin
CD276	CD276 molecule
CAV1	caveolin 1
SCGB1A1	secretoglobin family 1A member 1
PI4K2B	phosphatidylinositol 4-kinase type 2 beta

PLAT	plasminogen activator, tissue type
IL3	interleukin 3
KLHL5	kelch like family member 5
IL37	interleukin 37
ACP2	acid phosphatase 2, lysosomal
CCL24	C-C motif chemokine ligand 24
SLC17A5	solute carrier family 17 member 5
LRRC3	leucine rich repeat containing 3
MMP12	matrix metallopeptidase 12
TACR1	tachykinin receptor 1
TMCC2	transmembrane and coiled-coil domain family 2
CMA1	chymase 1
VIP	vasoactive intestinal peptide
CFH	complement factor H
ITGB3	integrin subunit beta 3
PCYT1B	phosphate cytidylyltransferase 1, choline, beta
STS	steroid sulfatase (microsomal), isozyme S
ELN	elastin
SERPINF2	serpin family F member 2
MAPK3	mitogen-activated protein kinase 3
GATS	GATS, stromal antigen 3 opposite strand
EPHB2	EPH receptor B2
DPEP1	dipeptidase 1 (renal)
CFLAR	CASP8 and FADD like apoptosis regulator
ACSL1	acyl-CoA synthetase long-chain family member 1

ID1	inhibitor of DNA binding 1, HLH protein
PSMA4	proteasome subunit alpha 4
RNF149	ring finger protein 149
FYB	FYN binding protein
KLF4	Kruppel like factor 4
ID2	inhibitor of DNA binding 2, HLH protein
ZC3H12A	zinc finger CCCH-type containing 12A
CTNNBL1	catenin beta like 1
ARL5B	ADP ribosylation factor like GTPase 5B
PPA1	pyrophosphatase (inorganic) 1
STEAP3	STEAP3 metalloreductase
SIRT1	sirtuin 1
NPPA	natriuretic peptide A
EHD1	EH domain containing 1
ICOS	inducible T-cell costimulator
TYRP1	tyrosinase related protein 1
SCARB2	scavenger receptor class B member 2
UTS2	urotensin 2
CXCL3	C-X-C motif chemokine ligand 3
SPOCD1	SPOC domain containing 1
HCAR2	hydroxycarboxylic acid receptor 2
CD44	CD44 molecule (Indian blood group)
ATP10A	ATPase phospholipid transporting 10A (putative)
CD46	CD46 molecule
APOE	apolipoprotein E

IL11	interleukin 11
CRH	corticotropin releasing hormone
CEBPA	CCAAT/enhancer binding protein alpha
HSPD1	heat shock protein family D (Hsp60) member 1
CFTR	cystic fibrosis transmembrane conductance regulator
PCDH1	protocadherin 1
CD83	CD83 molecule
HDAC9	histone deacetylase 9
CHIA	chitinase, acidic
LGALS3	galectin 3
CCR4	C-C motif chemokine receptor 4
CST3	cystatin C
C3AR1	complement C3a receptor 1
FCER2	Fc fragment of IgE receptor II
CAT	catalase
ETS1	ETS proto-oncogene 1, transcription factor
UGCG	UDP-glucose ceramide glucosyltransferase
TLR5	toll like receptor 5
AHCYL2	adenosylhomocysteinase like 2
DAB2IP	DAB2 interacting protein
RBMS2	RNA binding motif single stranded interacting protein 2
DNAH12	dynein axonemal heavy chain 12
LRIG1	leucine rich repeats and immunoglobulin like domains 1
TMEM268	transmembrane protein 268
SNTN	sentan, cilia apical structure protein

ARRDC3	arrestin domain containing 3
TINF2	TERF1 interacting nuclear factor 2
ANG	angiogenin
STAT3	signal transducer and activator of transcription 3
DEFB1	defensin beta 1
FAM216B	family with sequence similarity 216 member B
PRRT3	proline rich transmembrane protein 3
MMP8	matrix metallopeptidase 8
IL7R	interleukin 7 receptor
SMAD7	SMAD family member 7
RARRES2	retinoic acid receptor responder 2
IL22RA1	interleukin 22 receptor subunit alpha 1
ZNF841	zinc finger protein 841
RP1-71H24.1	
NFE2L3	nuclear factor, erythroid 2 like 3
CCR5	C-C motif chemokine receptor 5 (gene/pseudogene)
CMTM4	CKLF like MARVEL transmembrane domain containing 4
SLC16A1	solute carrier family 16 member 1
PATL1	PAT1 homolog 1, processing body mRNA decay factor
PLXNA2	plexin A2
NCF1C	neutrophil cytosolic factor 1C pseudogene
CYSLTR2	cysteinyl leukotriene receptor 2
ALPL	alkaline phosphatase, liver/bone/kidney
TRIM26	tripartite motif containing 26
ADAM12	ADAM metallopeptidase domain 12

TIMD4	T-cell immunoglobulin and mucin domain containing 4
JUNB	JunB proto-oncogene, AP-1 transcription factor subunit
ENO1	enolase 1
KYNU	kynureninase
NFE2L2	nuclear factor, erythroid 2 like 2
RPL17	ribosomal protein L17
TPT1	tumor protein, translationally-controlled 1
APOA1	apolipoprotein A1
NTRK1	neurotrophic receptor tyrosine kinase 1
SELE	selectin E
PECAM1	platelet and endothelial cell adhesion molecule 1
FAM65B	family with sequence similarity 65 member B
ARG1	arginase 1
LTF	lactotransferrin
CCL7	C-C motif chemokine ligand 7
CASP10	caspase 10
XPR1	xenotropic and polytropic retrovirus receptor 1
CASP8	caspase 8
NAMPT	nicotinamide phosphoribosyltransferase
ARID3B	AT-rich interaction domain 3B
AQP5	aquaporin 5
SCIN	scinderin
AGR2	anterior gradient 2, protein disulphide isomerase family member
ANPEP	alanyl aminopeptidase, membrane
SERPINB3	serpin family B member 3

CYBA	cytochrome b-245 alpha chain
CCL18	C-C motif chemokine ligand 18
ATG3	autophagy related 3
FAS	Fas cell surface death receptor
CALCA	calcitonin related polypeptide alpha
SLC26A9	solute carrier family 26 member 9
PAICS	phosphoribosylaminoimidazole carboxylase; phosphoribosylaminoimidazolesuccinocarboxamide synthase
NOD1	nucleotide binding oligomerization domain containing 1
SMS	spermine synthase
CLCA1	chloride channel accessory 1
BATF	basic leucine zipper ATF-like transcription factor
REN	renin
SEMA3A	semaphorin 3A
LRRN1	leucine rich repeat neuronal 1
SLC26A4	solute carrier family 26 member 4
OMG	oligodendrocyte myelin glycoprotein
VCAM1	vascular cell adhesion molecule 1
GSTA1	glutathione S-transferase alpha 1
STAT4	signal transducer and activator of transcription 4
IL5RA	interleukin 5 receptor subunit alpha
ACTB	actin beta
RAPGEF5	Rap guanine nucleotide exchange factor 5
ITK	IL2 inducible T-cell kinase
BRD1	bromodomain containing 1

IGFBP3	insulin like growth factor binding protein 3
S100A12	S100 calcium binding protein A12
SLURP1	secreted LY6/PLAUR domain containing 1
NAT2	N-acetyltransferase 2
PTPRH	protein tyrosine phosphatase, receptor type H
FYTTD1	forty-two-three domain containing 1
CCDC113	coiled-coil domain containing 113
RANGAP1	Ran GTPase activating protein 1
DCP1A	decapping mRNA 1A
NADK	NAD kinase
PSME2	proteasome activator subunit 2
SMCHD1	structural maintenance of chromosomes flexible hinge domain containing 1
WDR38	WD repeat domain 38
CSTB	cystatin B
NUDT4	nudix hydrolase 4
TOP1	topoisomerase (DNA) I
SLC27A3	solute carrier family 27 member 3
PLA2G1B	phospholipase A2 group IB
NOS3	nitric oxide synthase 3
NR0B2	nuclear receptor subfamily 0 group B member 2
MGAT3	mannosyl (beta-1,4-)-glycoprotein beta-1,4-N-acetylglucosaminyltransferase
OVOL1	ovo like transcriptional repressor 1
IL17D	interleukin 17D
AQP1	aquaporin 1 (Colton blood group)
LAT	linker for activation of T-cells

PF4	platelet factor 4
TUBA1A	tubulin alpha 1a
HDX	highly divergent homeobox
ASS1	argininosuccinate synthase 1
SNX6	sorting nexin 6
CBX5	chromobox 5
RIN2	Ras and Rab interactor 2
CHST6	carbohydrate sulfotransferase 6
CHAC2	ChaC cation transport regulator homolog 2
OSR2	odd-skipped related transciption factor 2
CD27	CD27 molecule
DNAL1	dynein axonemal light chain 1
ACKR4	atypical chemokine receptor 4
ITGB4	integrin subunit beta 4
IL13RA1	interleukin 13 receptor subunit alpha 1
TNFRSF18	TNF receptor superfamily member 18
FASLG	Fas ligand
NOTCH1	notch 1
MYLK	myosin light chain kinase
ACHE	acetylcholinesterase (Cartwright blood group)
SERPINB7	serpin family B member 7
ITIH6	inter-alpha-trypsin inhibitor heavy chain family member 6
HCAR3	hydroxycarboxylic acid receptor 3
GSTO2	glutathione S-transferase omega 2
FGFBP2	fibroblast growth factor binding protein 2

GRAMD1B	GRAM domain containing 1B
MKLN1	muskelin 1
CLCA3P	chloride channel accessory 3, pseudogene
MARCKS	myristoylated alanine rich protein kinase C substrate
HAO1	hydroxyacid oxidase 1
CCL3	C-C motif chemokine ligand 3
CR1	complement C3b/C4b receptor 1 (Knops blood group)
RGS22	regulator of G-protein signaling 22
ITGB2	integrin subunit beta 2
ARRB2	arrestin beta 2
CRTAM	cytotoxic and regulatory T-cell molecule
PRKCA	protein kinase C alpha
S100A8	S100 calcium binding protein A8
DYNC2H1	dynein cytoplasmic 2 heavy chain 1
TPI1	triosephosphate isomerase 1
SPESP1	sperm equatorial segment protein 1
KIF3A	kinesin family member 3A
EDARADD	EDAR associated death domain
RTKN2	rhotekin 2
CX3CR1	C-X3-C motif chemokine receptor 1
ANGPT1	angiopoietin 1
IGSF3	immunoglobulin superfamily member 3
OSM	oncostatin M
CCL20	C-C motif chemokine ligand 20
SERPINA1	serpin family A member 1

NLRP1	NLR family pyrin domain containing 1
CHM	CHM, Rab escort protein 1
ENTPD1	ectonucleoside triphosphate diphosphohydrolase 1
CXCR4	C-X-C motif chemokine receptor 4
CXCR3	C-X-C motif chemokine receptor 3
FBXL7	F-box and leucine rich repeat protein 7
APH1B	aph-1 homolog B, gamma-secretase subunit
DCLK1	doublecortin like kinase 1
S100A9	S100 calcium binding protein A9
TTC39B	tetratricopeptide repeat domain 39B
TLR6	toll like receptor 6
PLAU	plasminogen activator, urokinase
PGF	placental growth factor
DNAH6	dynein axonemal heavy chain 6
SNPH	syntaphilin
NPS	neuropeptide S
GH1	growth hormone 1
UNC119B	unc-119 lipid binding chaperone B
MTMR11	myotubularin related protein 11
CFAP221	cilia and flagella associated protein 221
COL1A1	collagen type I alpha 1 chain
LPA	lipoprotein(a)
GAPDH	glyceraldehyde-3-phosphate dehydrogenase
RAB8B	RAB8B, member RAS oncogene family
MED28	mediator complex subunit 28

TBCC	tubulin folding cofactor C
PTPRC	protein tyrosine phosphatase, receptor type C
PEPD	peptidase D
COLCA1	colorectal cancer associated 1
HLA-G	major histocompatibility complex, class I, G
CCL28	C-C motif chemokine ligand 28
RP11-430C7.5	
ESR1	estrogen receptor 1
SLCO6A1	solute carrier organic anion transporter family member 6A1
MMP2	matrix metallopeptidase 2
CTNNA3	catenin alpha 3
HNMT	histamine N-methyltransferase
IRF4	interferon regulatory factor 4
CCR1	C-C motif chemokine receptor 1
TACR2	tachykinin receptor 2
TNFAIP8L2	TNF alpha induced protein 8 like 2
PARP1	poly(ADP-ribose) polymerase 1
MOGAT1	monoacylglycerol O-acyltransferase 1
ADM	adrenomedullin
MAP1B	microtubule associated protein 1B
AMELX	amelogenin, X-linked
HLX	H2.0 like homeobox
BBC3	BCL2 binding component 3
CD69	CD69 molecule

SLC16A4	solute carrier family 16 member 4
HPSE	heparanase
ANXA1	annexin A1
CYP3A4	cytochrome P450 family 3 subfamily A member 4
TFDP2	transcription factor Dp-2
ATF5	activating transcription factor 5
LEPR	leptin receptor
THSD1	thrombospondin type 1 domain containing 1
TRPV2	transient receptor potential cation channel subfamily V member 2
TNFRSF6B	TNF receptor superfamily member 6b
SLAMF7	SLAM family member 7
CADM1	cell adhesion molecule 1
INSRR	insulin receptor related receptor
ADAM28	ADAM metallopeptidase domain 28
ZNF107	zinc finger protein 107
RGS2	regulator of G-protein signaling 2
LCN2	lipocalin 2
STAT5A	signal transducer and activator of transcription 5A
ARPP21	cAMP regulated phosphoprotein 21
RBP4	retinol binding protein 4
CFL1	cofilin 1
UCN	urocortin
AOAH	acyloxyacyl hydrolase
VASP	vasodilator-stimulated phosphoprotein
ZBTB43	zinc finger and BTB domain containing 43

FCER1A	Fc fragment of IgE receptor Ia
SERPINA3	serpin family A member 3
GRAMD2	GRAM domain containing 2
CSF1	colony stimulating factor 1
WFDC21P	WAP four-disulfide core domain 21, pseudogene
EPS8L1	EPS8 like 1
NQO1	NAD(P)H quinone dehydrogenase 1
VTN	vitronectin
ACE	angiotensin I converting enzyme
HDAC2	histone deacetylase 2
C5AR1	complement C5a receptor 1
SYT1	synaptotagmin 1
SERPINE2	serpin family E member 2
NLRP3	NLR family pyrin domain containing 3
CD177	CD177 molecule
HP	haptoglobin
TPO	thyroid peroxidase
TRPM2	transient receptor potential cation channel subfamily M member 2
RAMP1	receptor activity modifying protein 1
CCR2	C-C motif chemokine receptor 2
IL7	interleukin 7
ANO7L1	anoctamin 7 like 1
THUMPD2	THUMP domain containing 2
LAMA1	laminin subunit alpha 1
ITGB1	integrin subunit beta 1

FN1	fibronectin 1
IL12A	interleukin 12A
CCDC33	coiled-coil domain containing 33
CCR6	C-C motif chemokine receptor 6
TLR9	toll like receptor 9
MSRB2	methionine sulfoxide reductase B2
CYP2R1	cytochrome P450 family 2 subfamily R member 1
TAC1	tachykinin precursor 1
RGS4	regulator of G-protein signaling 4
CCR9	C-C motif chemokine receptor 9
AICDA	activation induced cytidine deaminase
RP11-327F22.1	
FKBP1B	FK506 binding protein 1B
SAA1	serum amyloid A1
IL1R2	interleukin 1 receptor type 2
PRDM1	PR/SET domain 1
CLDN18	claudin 18
HDAC1	histone deacetylase 1
NELFCD	negative elongation factor complex member C/D
PNOC	prepronociceptin
NF2	neurofibromin 2
HTR4	5-hydroxytryptamine receptor 4
SELPLG	selectin P ligand
CENPJ	centromere protein J

RP11-1008C21.1	
ATP5A1P10	ATP synthase, H+ transporting, mitochondrial F1 complex, alpha subunit 1 pseudogene 10
KCNMB2	potassium calcium-activated channel subfamily M regulatory beta subunit 2
DUSP26	dual specificity phosphatase 26 (putative)
PPY	pancreatic polypeptide
CD151	CD151 molecule (Raph blood group)
CCL3L3	C-C motif chemokine ligand 3 like 3
KRT18	keratin 18
PGP	phosphoglycolate phosphatase
C1orf100	chromosome 1 open reading frame 100
CCDC190	coiled-coil domain containing 190
TENM3	teneurin transmembrane protein 3
ELF5	E74 like ETS transcription factor 5
SLC6A12	solute carrier family 6 member 12
BGLAP	bone gamma-carboxyglutamate protein
PAPPA	pappalysin 1
PPIL6	peptidylprolyl isomerase like 6
TMEM27	transmembrane protein 27
MME	membrane metalloendopeptidase
A2M	alpha-2-macroglobulin
CDH17	cadherin 17
IRF5	interferon regulatory factor 5
FOXA2	forkhead box A2

COL26A1	collagen type XXVI alpha 1 chain
CHRNA1	cholinergic receptor nicotinic alpha 1 subunit
TNFRSF9	TNF receptor superfamily member 9
PTGIR	prostaglandin I2 (prostacyclin) receptor (IP)
PPARGC1B	PPARG coactivator 1 beta
GUCA2A	guanylate cyclase activator 2A
TRPA1	transient receptor potential cation channel subfamily A member 1
CLN3	CLN3, battenin
UBXN10	UBX domain protein 10
ABO	ABO blood group (transferase A, alpha 1-3-N-acetylgalactosaminyltransferase; transferase B, alpha 1-3-galactosyltransferase)
TET1	tet methylcytosine dioxygenase 1
CSF1R	colony stimulating factor 1 receptor
TXN	thioredoxin
IL1R1	interleukin 1 receptor type 1
RECK	reversion inducing cysteine rich protein with kazal motifs
GAS1	growth arrest specific 1
ATPAF1	ATP synthase mitochondrial F1 complex assembly factor 1
LGALS7B	galectin 7B
TIMP2	TIMP metallopeptidase inhibitor 2
PAX5	paired box 5
RBM45	RNA binding motif protein 45
PTAFR	platelet activating factor receptor
LTB	lymphotoxin beta
FST	follistatin

NPTX1	neuronal pentraxin 1
GC	GC, vitamin D binding protein
TFF2	trefoil factor 2
SPRR2B	small proline rich protein 2B
CCR7	C-C motif chemokine receptor 7
ATG5	autophagy related 5
NEK10	NIMA related kinase 10
NDUFAB1	NADH:ubiquinone oxidoreductase subunit AB1
ANXA5	annexin A5
DUSP1	dual specificity phosphatase 1
TNFRSF13B	TNF receptor superfamily member 13B
GPX1	glutathione peroxidase 1
C7orf49	chromosome 7 open reading frame 49
CSH1	chorionic somatomammotropin hormone 1
MCAT	malonyl-CoA-acyl carrier protein transacylase
GPR35	G protein-coupled receptor 35
PMCH	pro-melanin concentrating hormone
SH3PXD2A	SH3 and PX domains 2A
CLSTN1	calsyntenin 1
SCRT1	scratch family transcriptional repressor 1
DCBLD2	discoidin, CUB and LCCL domain containing 2
CHRM2	cholinergic receptor muscarinic 2
KRT19	keratin 19
TP73	tumor protein p73
GJA1	gap junction protein alpha 1

KATNAL1	katanin catalytic subunit A1 like 1
TNFRSF19	TNF receptor superfamily member 19
MMP3	matrix metallopeptidase 3
F5	coagulation factor V
HELB	DNA helicase B
LIFR-AS1	LIFR antisense RNA 1
NOD2	nucleotide binding oligomerization domain containing 2
LDLR	low density lipoprotein receptor
MAF	MAF bZIP transcription factor
TFPI	tissue factor pathway inhibitor
MUC19	mucin 19, oligomeric
SIGLEC8	sialic acid binding Ig like lectin 8
LPAR6	lysophosphatidic acid receptor 6
PIK3CD	phosphatidylinositol-4,5-bisphosphate 3-kinase catalytic subunit delta
MTHFR	methylenetetrahydrofolate reductase
C1S	complement C1s
IL17RA	interleukin 17 receptor A
CRHR2	corticotropin releasing hormone receptor 2
CXCR5	C-X-C motif chemokine receptor 5
NOX4	NADPH oxidase 4
UBE3C	ubiquitin protein ligase E3C
ADGRA3	adhesion G protein-coupled receptor A3
C5AR2	complement component 5a receptor 2
HSP90AB1	heat shock protein 90 alpha family class B member 1
HGF	hepatocyte growth factor

IL16	interleukin 16
FCRL3	Fc receptor like 3
RBM24	RNA binding motif protein 24
MUC5B	mucin 5B, oligomeric mucus/gel-forming
DHRS4-AS1	DHRS4 antisense RNA 1
FLG	filaggrin
CYBB	cytochrome b-245 beta chain
CTSS	cathepsin S
CDA	cytidine deaminase
IER2	immediate early response 2
NPR1	natriuretic peptide receptor 1
GZMB	granzyme B
PPARGC1A	PPARG coactivator 1 alpha
HRH2	histamine receptor H2
MAP6	microtubule associated protein 6
ARHGAP24	Rho GTPase activating protein 24
PDIA3	protein disulfide isomerase family A member 3
FOXE1	forkhead box E1
HS3ST3A1	heparan sulfate-glucosamine 3-sulfotransferase 3A1
VWF	von Willebrand factor
ARID5A	AT-rich interaction domain 5A
AK7	adenylate kinase 7
C1orf174	chromosome 1 open reading frame 174
NFATC1	nuclear factor of activated T-cells 1
TRPM6	transient receptor potential cation channel subfamily M member 6

RORC	RAR related orphan receptor C
NOCT	nocturnin
PSMB10	proteasome subunit beta 10
PIM3	Pim-3 proto-oncogene, serine/threonine kinase
SOCS2	suppressor of cytokine signaling 2
ISLR2	immunoglobulin superfamily containing leucine rich repeat 2
ITGB7	integrin subunit beta 7
HAVCR2	hepatitis A virus cellular receptor 2
AC011997.1	
PDZRN4	PDZ domain containing ring finger 4
ALB	albumin
LRRN2	leucine rich repeat neuronal 2
MRC1	mannose receptor, C type 1
TNFSF9	tumor necrosis factor superfamily member 9
STIM1	stromal interaction molecule 1
NEDD9	neural precursor cell expressed, developmentally down-regulated 9
IFNA2	interferon alpha 2
FABP5	fatty acid binding protein 5
SMAD2	SMAD family member 2
CRHR1	corticotropin releasing hormone receptor 1
CYP1A2	cytochrome P450 family 1 subfamily A member 2
PPARA	peroxisome proliferator activated receptor alpha
EHF	ETS homologous factor
CCL15	C-C motif chemokine ligand 15
CA10	carbonic anhydrase 10

BIRC5	baculoviral IAP repeat containing 5
FOXN1	forkhead box N1
MFAP4	microfibrillar associated protein 4
DMTN	dematin actin binding protein
EFCAB1	EF-hand calcium binding domain 1
ADCYAP1R1	ADCYAP receptor type I
ANKUB1	ankyrin repeat and ubiquitin domain containing 1
EFHB	EF-hand domain family member B
GSN	gelsolin
ITCH	itchy E3 ubiquitin protein ligase
SLC6A7	solute carrier family 6 member 7
BMP7	bone morphogenetic protein 7
ADCY9	adenylate cyclase 9
CASP3	caspase 3
SLCO4A1	solute carrier organic anion transporter family member 4A1
LRRC10B	leucine rich repeat containing 10B
RYR2	ryanodine receptor 2
HAPLN3	hyaluronan and proteoglycan link protein 3
PTPN11	protein tyrosine phosphatase, non-receptor type 11
EFHC2	EF-hand domain containing 2
NWD1	NACHT and WD repeat domain containing 1
MMS22L	MMS22 like, DNA repair protein
USP30-AS1	USP30 antisense RNA 1
FSIP1	fibrous sheath interacting protein 1
LRTOMT	leucine rich transmembrane and O-methyltransferase domain containing

ICOSLG	inducible T-cell costimulator ligand
BDKRB2	bradykinin receptor B2
ST8SIA4	ST8 alpha-N-acetyl-neuraminide alpha-2,8-sialyltransferase 4
TACR3	tachykinin receptor 3
CERK	ceramide kinase
CHIT1	chitinase 1
JUN	Jun proto-oncogene, AP-1 transcription factor subunit
ELANE	elastase, neutrophil expressed
SLC25A3	solute carrier family 25 member 3
CYP2C19	cytochrome P450 family 2 subfamily C member 19
MBTPS1	membrane bound transcription factor peptidase, site 1
TXNIP	thioredoxin interacting protein
SRD5A2	steroid 5 alpha-reductase 2
RGS5	regulator of G-protein signaling 5
RP11-467J12.4	
MAG	myelin associated glycoprotein
C7orf26	chromosome 7 open reading frame 26
CXCL13	C-X-C motif chemokine ligand 13
ID3	inhibitor of DNA binding 3, HLH protein
ZBTB16	zinc finger and BTB domain containing 16
STX2	syntaxin 2
IL9R	interleukin 9 receptor
ID4	inhibitor of DNA binding 4, HLH protein
ADAM10	ADAM metallopeptidase domain 10

FZD8	frizzled class receptor 8
CYP1A1	cytochrome P450 family 1 subfamily A member 1
JAK3	Janus kinase 3
WISP1	WNT1 inducible signaling pathway protein 1
ECT2L	epithelial cell transforming 2 like
MORN5	MORN repeat containing 5
FYN	FYN proto-oncogene, Src family tyrosine kinase
04-Sep	septin 4
CP	ceruloplasmin
CDON	cell adhesion associated, oncogene regulated
CASP9	caspase 9
ORM1	orosomucoid 1
C17orf67	chromosome 17 open reading frame 67
MEFV	Mediterranean fever
PON1	paraoxonase 1
PRKCE	protein kinase C epsilon
IL13RA2	interleukin 13 receptor subunit alpha 2
ZNF614	zinc finger protein 614
NFKB2	nuclear factor kappa B subunit 2
SOCS5	suppressor of cytokine signaling 5
IL2RA	interleukin 2 receptor subunit alpha
DCN	decorin
CHRM1	cholinergic receptor muscarinic 1
DTNA	dystrobrevin alpha
LINC00094	long intergenic non-protein coding RNA 94

HSD11B2	hydroxysteroid 11-beta dehydrogenase 2
MIF	macrophage migration inhibitory factor (glycosylation-inhibiting factor)
PGD	phosphogluconate dehydrogenase
AMH	anti-Mullerian hormone
DBH	dopamine beta-hydroxylase
RAPGEF3	Rap guanine nucleotide exchange factor 3
JAK1	Janus kinase 1
LPCAT1	lysophosphatidylcholine acyltransferase 1
ATRN	attractin
CCL27	C-C motif chemokine ligand 27
SLC22A2	solute carrier family 22 member 2
ARL14	ADP ribosylation factor like GTPase 14
DUSP2	dual specificity phosphatase 2
IFNGR2	interferon gamma receptor 2 (interferon gamma transducer 1)
FCGR2A	Fc fragment of IgG receptor IIa
TTN	titin
VNN1	vanin 1
FDFT1	farnesyl-diphosphate farnesyltransferase 1
MAPK9	mitogen-activated protein kinase 9
PGAP1	post-GPI attachment to proteins 1
TREM1	triggering receptor expressed on myeloid cells 1
AP001610.5	
PTPRR	protein tyrosine phosphatase, receptor type R
CETP	cholesteryl ester transfer protein
CD200	CD200 molecule

EMD	emerin
TMEM132D	transmembrane protein 132D
KLK1	kallikrein 1
NPAS3	neuronal PAS domain protein 3
CPA1	carboxypeptidase A1
MILR1	mast cell immunoglobulin like receptor 1
ADA	adenosine deaminase
LPO	lactoperoxidase
NKX3-1	NK3 homeobox 1
PTH	parathyroid hormone
NUP43	nucleoporin 43
HSPB3	heat shock protein family B (small) member 3
CRIM1	cysteine rich transmembrane BMP regulator 1
FBLN1	fibulin 1
FABP4	fatty acid binding protein 4
SPDEF	SAM pointed domain containing ETS transcription factor
MYB	MYB proto-oncogene, transcription factor
C1orf141	chromosome 1 open reading frame 141
RP11-757G1.6	
ASXL3	additional sex combs like 3, transcriptional regulator
AHR	aryl hydrocarbon receptor
GSK3B	glycogen synthase kinase 3 beta
CYP21A2	cytochrome P450 family 21 subfamily A member 2
CD53	CD53 molecule

CTSD	cathepsin D
PDCD4	programmed cell death 4 (neoplastic transformation inhibitor)
TAAR6	trace amine associated receptor 6
ENPP5	ectonucleotide pyrophosphatase/phosphodiesterase 5 (putative)
TLR8	toll like receptor 8
CXCL1	C-X-C motif chemokine ligand 1
CDH2	cadherin 2
ITGA2	integrin subunit alpha 2
PITPNC1	phosphatidylinositol transfer protein, cytoplasmic 1
ACMSD	aminocarboxymuconate semialdehyde decarboxylase
BLK	BLK proto-oncogene, Src family tyrosine kinase
HHIP	hedgehog interacting protein
FAM167A	family with sequence similarity 167 member A
PTPN22	protein tyrosine phosphatase, non-receptor type 22
SCLT1	sodium channel and clathrin linker 1
OR52K3P	olfactory receptor family 52 subfamily K member 3 pseudogene
FOXA3	forkhead box A3
ACSL3	acyl-CoA synthetase long-chain family member 3
DNMT1	DNA methyltransferase 1
THPO	thrombopoietin
MUC1	mucin 1, cell surface associated
AKR1B1	aldo-keto reductase family 1 member B
NEDD4L	neural precursor cell expressed, developmentally down-regulated 4-like, E3 ubiquitin protein ligase
IKBKB	inhibitor of kappa light polypeptide gene enhancer in B-cells, kinase beta

P2RX1	purinergic receptor P2X 1
XRCC1	X-ray repair cross complementing 1
SFSWAP	splicing factor SWAP homolog
CIITA	class II major histocompatibility complex transactivator
KIR2DL4	killer cell immunoglobulin like receptor, two Ig domains and long cytoplasmic tail 4
THBD	thrombomodulin
USF2	upstream transcription factor 2, c-fos interacting
CEBPB	CCAAT/enhancer binding protein beta
PZP	PZP, alpha-2-macroglobulin like
NRL	neural retina leucine zipper
PRH1	proline rich protein HaeIII subfamily 1
NEXN	nexilin F-actin binding protein
ZPBP2	zona pellucida binding protein 2
AQP3	aquaporin 3 (Gill blood group)
KCNMA1	potassium calcium-activated channel subfamily M alpha 1
PLCG1	phospholipase C gamma 1
STOML2	stomatin like 2
NTF3	neurotrophin 3
CIART	circadian associated repressor of transcription
SLC26A4-AS1	SLC26A4 antisense RNA 1
TNFAIP6	TNF alpha induced protein 6
MICA	MHC class I polypeptide-related sequence A
CD59	CD59 molecule
LARGE1	LARGE xylosyl- and glucuronyltransferase 1

INPP4A	inositol polyphosphate-4-phosphatase type I A
IRF2	interferon regulatory factor 2
ST13	suppression of tumorigenicity 13 (colon carcinoma) (Hsp70 interacting protein)
ENPP1	ectonucleotide pyrophosphatase/phosphodiesterase 1
NFATC2	nuclear factor of activated T-cells 2
MMP7	matrix metallopeptidase 7
CISH	cytokine inducible SH2 containing protein
DUOX1	dual oxidase 1
AP3B1	adaptor related protein complex 3 beta 1 subunit
BNC2	basonuclin 2
PARD3B	par-3 family cell polarity regulator beta
FTCDNL1	formiminotransferase cyclodeaminase N-terminal like
MPP7	membrane palmitoylated protein 7
SLC5A12	solute carrier family 5 member 12
KRT81	keratin 81
GREB1	growth regulation by estrogen in breast cancer 1
CCDC167	coiled-coil domain containing 167
ADAMTSL1	ADAMTS like 1
OR5V1	olfactory receptor family 5 subfamily V member 1
ITGA8	integrin subunit alpha 8
CTC-432M15.3	
TMEM26	transmembrane protein 26
RAPGEF6	Rap guanine nucleotide exchange factor 6
LUC7L2	LUC7 like 2, pre-mRNA splicing factor

PRICKLE2	prickle planar cell polarity protein 2
OTOG	otogelin
DPH1	diphthamide biosynthesis 1
KRT86	keratin 86
C7orf55-LUC7L2	C7orf55-LUC7L2 readthrough
KRT7	keratin 7
IGFBP2	insulin like growth factor binding protein 2
SLC28A3	solute carrier family 28 member 3
CSMD1	CUB and Sushi multiple domains 1
CD163	CD163 molecule
ACAA1	acetyl-CoA acyltransferase 1
MYCL	v-myc avian myelocytomatosis viral oncogene lung carcinoma derived homolog
RYR1	ryanodine receptor 1
CCDC138	coiled-coil domain containing 138
PTPRD	protein tyrosine phosphatase, receptor type D
CSTA	cystatin A
AIMP1	aminoacyl tRNA synthetase complex interacting multifunctional protein 1
GPR68	G protein-coupled receptor 68
MTX1	metaxin 1
VIPR1	vasoactive intestinal peptide receptor 1
NWD2	NACHT and WD repeat domain containing 2
MIR3142HG	MIR3142 host gene
GLB1	galactosidase beta 1
CD48	CD48 molecule

FAM92B	family with sequence similarity 92 member B
TNFRSF21	TNF receptor superfamily member 21
SPTBN5	spectrin beta, non-erythrocytic 5
F2R	coagulation factor II thrombin receptor
CCL19	C-C motif chemokine ligand 19
GLI2	GLI family zinc finger 2
OAF	out at first homolog
LGALS1	galectin 1
P2RX7	purinergic receptor P2X 7
CCDC151	coiled-coil domain containing 151
ALDH2	aldehyde dehydrogenase 2 family (mitochondrial)
ORAI2	ORAI calcium release-activated calcium modulator 2
MCAM	melanoma cell adhesion molecule
ZCCHC6	zinc finger CCHC-type containing 6
TNFSF10	tumor necrosis factor superfamily member 10
LY6D	lymphocyte antigen 6 complex, locus D
GCA	grancalcin
KCNIP4	potassium voltage-gated channel interacting protein 4
SLC12A2	solute carrier family 12 member 2
ANGPTL1	angiopoietin like 1
CDC42EP2	CDC42 effector protein 2
IL23R	interleukin 23 receptor
AIPL1	aryl hydrocarbon receptor interacting protein like 1
GALR1	galanin receptor 1
POLR2M	RNA polymerase II subunit M

FIGN	fidgetin, microtubule severing factor
ALK	anaplastic lymphoma receptor tyrosine kinase
DDAH1	dimethylarginine dimethylaminohydrolase 1
ATP7A	ATPase copper transporting alpha
FAM122C	family with sequence similarity 122C
CASP5	caspase 5
HS3ST1	heparan sulfate-glucosamine 3-sulfotransferase 1
SP140	SP140 nuclear body protein
AGBL2	ATP/GTP binding protein like 2
HRH3	histamine receptor H3
SIRPB2	signal regulatory protein beta 2
GPR156	G protein-coupled receptor 156
HAS2	hyaluronan synthase 2
CPB2	carboxypeptidase B2
GFRA2	GDNF family receptor alpha 2
CBL	Cbl proto-oncogene
MAP3K8	mitogen-activated protein kinase kinase kinase 8
DRAM1	DNA damage regulated autophagy modulator 1
EFCAB12	EF-hand calcium binding domain 12
C12orf49	chromosome 12 open reading frame 49
NREP	neuronal regeneration related protein
GALNT18	polypeptide N-acetylgalactosaminyltransferase 18
PTPRT	protein tyrosine phosphatase, receptor type T
PARVA	parvin alpha
MKX	mohawk homeobox

RARRES1	retinoic acid receptor responder 1
CREB5	cAMP responsive element binding protein 5
SYT9	synaptotagmin 9
SNAP25	synaptosome associated protein 25
AGFG1	ArfGAP with FG repeats 1
VCAN	versican
FHL3	four and a half LIM domains 3
BCHE	butyrylcholinesterase
CRYZ	crystallin zeta
DEFB4A	defensin beta 4A
SLC35E2	solute carrier family 35 member E2
CD209	CD209 molecule
AOC1	amine oxidase, copper containing 1
SMC3	structural maintenance of chromosomes 3
AIRE	autoimmune regulator
EPHA7	EPH receptor A7
KCND3	potassium voltage-gated channel subfamily D member 3
SDC4	syndecan 4
CCL21	C-C motif chemokine ligand 21
GCLM	glutamate-cysteine ligase modifier subunit
RGS7BP	regulator of G-protein signaling 7 binding protein
PPP1R12A	protein phosphatase 1 regulatory subunit 12A
GPX3	glutathione peroxidase 3
KCCAT211	renal clear cell carcinoma-associated transcript 211
GABRA5	gamma-aminobutyric acid type A receptor alpha5 subunit

MUC2	mucin 2, oligomeric mucus/gel-forming
DGKA	diacylglycerol kinase alpha
IPO13	importin 13
CLDN1	claudin 1
NAT8	N-acetyltransferase 8 (putative)
ZNF704	zinc finger protein 704
COX7A2	cytochrome c oxidase subunit 7A2
MRO	maestro
REEP1	receptor accessory protein 1
APOL4	apolipoprotein L4
IFT122	intraflagellar transport 122
CDH13	cadherin 13
RP2	retinitis pigmentosa 2 (X-linked recessive)
SEMA7A	semaphorin 7A (John Milton Hagen blood group)
DHRS2	dehydrogenase/reductase 2
EHHADH	enoyl-CoA hydratase and 3-hydroxyacyl CoA dehydrogenase
SSH1	slingshot protein phosphatase 1
DDR1	discoidin domain receptor tyrosine kinase 1
ATF6B	activating transcription factor 6 beta
MYOCD	myocardin
TFG	TRK-fused gene
DTD1	D-tyrosyl-tRNA deacylase 1
CD226	CD226 molecule
SRF	serum response factor
MAN1A1	mannosidase alpha class 1A member 1

MTOR	mechanistic target of rapamycin
IRAK1	interleukin 1 receptor associated kinase 1
STK10	serine/threonine kinase 10
TLN1	talin 1
ZNF71	zinc finger protein 71
RP1-140K8.5	
MMP14	matrix metallopeptidase 14
ZBED3	zinc finger BED-type containing 3
CHUK	conserved helix-loop-helix ubiquitous kinase
CNR1	cannabinoid receptor 1
TF	transferrin
YME1L1	YME1 like 1 ATPase
TNFRSF25	TNF receptor superfamily member 25
BMPER	BMP binding endothelial regulator
SPIN1	spindlin 1
UBR5	ubiquitin protein ligase E3 component n-recognin 5
FAM104B	family with sequence similarity 104 member B
EPO	erythropoietin
F3	coagulation factor III, tissue factor
CCDC81	coiled-coil domain containing 81
MAP2K1	mitogen-activated protein kinase kinase 1
GSTO1	glutathione S-transferase omega 1
CD81	CD81 molecule
PGAP3	post-GPI attachment to proteins 3
SCD	stearoyl-CoA desaturase

ANXA6	annexin A6
CEACAM6	carcinoembryonic antigen related cell adhesion molecule 6
MUC21	mucin 21, cell surface associated
CD19	CD19 molecule
DPT	dermatopontin
PRTFDC1	phosphoribosyl transferase domain containing 1
LRP1B	LDL receptor related protein 1B
KIAA0922	KIAA0922
CCND1	cyclin D1
MLYCD	malonyl-CoA decarboxylase
SMTN	smoothelin
CTD-2015G9.2	
IFIT1B	interferon induced protein with tetratricopeptide repeats 1B
CDK9	cyclin dependent kinase 9
PYGB	phosphorylase, glycogen; brain
ATP6V0A4	ATPase H+ transporting V0 subunit a4
AC093495.4	
ZNF432	zinc finger protein 432
SUMF2	sulfatase modifying factor 2
KRT6C	keratin 6C
RASSF10	Ras association domain family member 10
CCK	cholecystokinin
RORB	RAR related orphan receptor B
CXCL5	C-X-C motif chemokine ligand 5

CDC14A	cell division cycle 14A
LMNTD1	lamin tail domain containing 1
PIK3CG	phosphatidylinositol-4,5-bisphosphate 3-kinase catalytic subunit gamma
PRPH	peripherin
RACK1	receptor for activated C kinase 1
DEFA1	defensin alpha 1
PCSK2	proprotein convertase subtilisin/kexin type 2
RND1	Rho family GTPase 1
ENPP2	ectonucleotide pyrophosphatase/phosphodiesterase 2
FLT1	fms related tyrosine kinase 1
FKBP5	FK506 binding protein 5
TLR10	toll like receptor 10
CREM	cAMP responsive element modulator
SKIL	SKI like proto-oncogene
KLK10	kallikrein related peptidase 10
GUSB	glucuronidase beta
SLCO1A2	solute carrier organic anion transporter family member 1A2
NIN	ninein
SLC29A3	solute carrier family 29 member 3
CACNG3	calcium voltage-gated channel auxiliary subunit gamma 3
PRKCDBP	protein kinase C delta binding protein
KLF13	Kruppel like factor 13
DARS	aspartyl-tRNA synthetase
FAM172A	family with sequence similarity 172 member A
IGF1	insulin like growth factor 1

TFDP3	transcription factor Dp family member 3
C10orf107	chromosome 10 open reading frame 107
SLFN13	schlafen family member 13
MGEA5	meningioma expressed antigen 5 (hyaluronidase)
HSD11B1	hydroxysteroid 11-beta dehydrogenase 1
CCL8	C-C motif chemokine ligand 8
ABTB2	ankyrin repeat and BTB domain containing 2
GPI	glucose-6-phosphate isomerase
B2M	beta-2-microglobulin
SHH	sonic hedgehog
SOD3	superoxide dismutase 3, extracellular
KIAA2012	KIAA2012
MMP25	matrix metallopeptidase 25
CLC	Charcot-Leyden crystal galectin
CCDC140	coiled-coil domain containing 140
CD63	CD63 molecule
ARG2	arginase 2
PGRMC1	progesterone receptor membrane component 1
EPHA4	EPH receptor A4
CD7	CD7 molecule
SRC	SRC proto-oncogene, non-receptor tyrosine kinase
ADGRA1	adhesion G protein-coupled receptor A1
SST	somatostatin
F8	coagulation factor VIII
TFAM	transcription factor A, mitochondrial

UCN3	urocortin 3
ACRC	acidic repeat containing
FCGR2B	Fc fragment of IgG receptor IIb
FOXN2	forkhead box N2
TSC22D3	TSC22 domain family member 3
FAM166B	family with sequence similarity 166 member B
C14orf177	chromosome 14 open reading frame 177
GLIS3-AS1	GLIS3 antisense RNA 1
SPHK1	sphingosine kinase 1
SPRR1A	small proline rich protein 1A
SEMA6A	semaphorin 6A
RELB	RELB proto-oncogene, NF-kB subunit
RABL2A	RAB, member of RAS oncogene family-like 2A
FLT3LG	fms related tyrosine kinase 3 ligand
C1orf87	chromosome 1 open reading frame 87
BAMBI	BMP and activin membrane bound inhibitor
FCGR3A	Fc fragment of IgG receptor IIIa
OPRK1	opioid receptor kappa 1
RNGTT	RNA guanylyltransferase and 5'-phosphatase
KRT39	keratin 39
GIF	gastric intrinsic factor
OLR1	oxidized low density lipoprotein receptor 1
IKBKAP	inhibitor of kappa light polypeptide gene enhancer in B-cells, kinase complex-associated protein
NTF4	neurotrophin 4

HES4	hes family bHLH transcription factor 4
GAS2L2	growth arrest specific 2 like 2
CNTN4	contactin 4
APP	amyloid beta precursor protein
CLEC2D	C-type lectin domain family 2 member D
CTTN	cortactin
KLRD1	killer cell lectin like receptor D1
HNF4G	hepatocyte nuclear factor 4 gamma
ATP8A2	ATPase phospholipid transporting 8A2
RARS	arginyl-tRNA synthetase
GPX2	glutathione peroxidase 2
APLN	apelin
DANCR	differentiation antagonizing non-protein coding RNA
SLC33A1	solute carrier family 33 member 1
AGTR1	angiotensin II receptor type 1
PLEKHO2	pleckstrin homology domain containing O2
JAZF1	JAZF zinc finger 1
OTC	ornithine carbamoyltransferase
NTRK2	neurotrophic receptor tyrosine kinase 2
NTS	neurotensin
CCR10	C-C motif chemokine receptor 10
P3H3	prolyl 3-hydroxylase 3
TRPC1	transient receptor potential cation channel subfamily C member 1
ZFPM1	zinc finger protein, FOG family member 1
CALM3	calmodulin 3

MTURN	maturin, neural progenitor differentiation regulator homolog
PIK3CA	phosphatidylinositol-4,5-bisphosphate 3-kinase catalytic subunit alpha
LPAR2	lysophosphatidic acid receptor 2
TAPBP	TAP binding protein
TBXAS1	thromboxane A synthase 1
EPGN	epithelial mitogen
TNFRSF1A	TNF receptor superfamily member 1A
RP11-403I13.8	
ANK1	ankyrin 1
NARFL	nuclear prelamin A recognition factor like
KDM4C	lysine demethylase 4C
CDH18	cadherin 18
TRIM31	tripartite motif containing 31
EFNA1	ephrin A1
XCL1	X-C motif chemokine ligand 1
GOT2	glutamic-oxaloacetic transaminase 2
FPR2	formyl peptide receptor 2
PRDX2	peroxiredoxin 2
INPP5D	inositol polyphosphate-5-phosphatase D
DEGS1	delta 4-desaturase, sphingolipid 1
FAM189A1	family with sequence similarity 189 member A1
IL25	interleukin 25
APOH	apolipoprotein H
FILIP1	filamin A interacting protein 1

SFTPA1	surfactant protein A1
TMEM74B	transmembrane protein 74B
C2CD4B	C2 calcium dependent domain containing 4B
SIGIRR	single Ig and TIR domain containing
PCDH17	protocadherin 17
THBS2	thrombospondin 2
HDC	histidine decarboxylase
CLCF1	cardiotrophin-like cytokine factor 1
DBX1	developing brain homeobox 1
DMRT1	doublesex and mab-3 related transcription factor 1
NR0B1	nuclear receptor subfamily 0 group B member 1
TMEM219	transmembrane protein 219
TRPC3	transient receptor potential cation channel subfamily C member 3
HSD3B1	hydroxy-delta-5-steroid dehydrogenase, 3 beta- and steroid delta-isomerase 1
EPB41L4B	erythrocyte membrane protein band 4.1 like 4B
NDRG1	N-myc downstream regulated 1
SPRY1	sprouty RTK signaling antagonist 1
ARRDC4	arrestin domain containing 4
BID	BH3 interacting domain death agonist
GALNT5	polypeptide N-acetylgalactosaminyltransferase 5
FURIN	furin, paired basic amino acid cleaving enzyme
CTDP1	CTD phosphatase subunit 1
MCPH1	microcephalin 1
ETV1	ETS variant 1
SLITRK5	SLIT and NTRK like family member 5

GNAI1	G protein subunit alpha i1
CACNA2D1	calcium voltage-gated channel auxiliary subunit alpha2delta 1
TWIST1	twist family bHLH transcription factor 1
TCL1B	T-cell leukemia/lymphoma 1B
ASAP1	ArfGAP with SH3 domain, ankyrin repeat and PH domain 1
NFASC	neurofascin
SOX4	SRY-box 4
PCDH7	protocadherin 7
FOXL1	forkhead box L1
MYLIP	myosin regulatory light chain interacting protein
NUFIP1	NUFIP1, FMR1 interacting protein 1
MICAL2	microtubule associated monooxygenase, calponin and LIM domain containing 2
ZFP42	ZFP42 zinc finger protein
TBX1	T-box 1
AKAP2	A-kinase anchoring protein 2
GUCY2C	guanylate cyclase 2C
EIF4E	eukaryotic translation initiation factor 4E
ZMYND15	zinc finger MYND-type containing 15
TGFBR2	transforming growth factor beta receptor 2
SERPINB2	serpin family B member 2
C11orf16	chromosome 11 open reading frame 16
DUSP8	dual specificity phosphatase 8
GPR148	G protein-coupled receptor 148
MUC15	mucin 15, cell surface associated
MYLK3	myosin light chain kinase 3

RAG2	recombination activating 2
MS4A1	membrane spanning 4-domains A1
FAM229B	family with sequence similarity 229 member B
DYX1C1	dyslexia susceptibility 1 candidate 1
SFTPB	surfactant protein B
CD24	CD24 molecule
CD93	CD93 molecule
ANO3	anoctamin 3
ATF4	activating transcription factor 4
KCNMB1	potassium calcium-activated channel subfamily M regulatory beta subunit 1
STIP1	stress induced phosphoprotein 1
CCL4	C-C motif chemokine ligand 4
OR7E47P	olfactory receptor family 7 subfamily E member 47 pseudogene
SPEF1	sperm flagellar 1
IL12RB1	interleukin 12 receptor subunit beta 1
RP1	retinitis pigmentosa 1 (autosomal dominant)
CARD17	caspase recruitment domain family member 17
PDCD1	programmed cell death 1
AMBN	ameloblastin
IVL	involucrin
CTSB	cathepsin B
NFIA	nuclear factor I A
FANCA	Fanconi anemia complementation group A
MPL	MPL proto-oncogene, thrombopoietin receptor
TAGLN	transgelin

C6	complement C6
MAP3K5	mitogen-activated protein kinase kinase kinase 5
DLL1	delta like canonical Notch ligand 1
SLC30A1	solute carrier family 30 member 1
PNN	pinin, desmosome associated protein
TRPV4	transient receptor potential cation channel subfamily V member 4
SDCBP2	syndecan binding protein 2
RP4-529N6.2	
MEP1B	meprin A subunit beta
NR3C2	nuclear receptor subfamily 3 group C member 2
PLD2	phospholipase D2
NRP1	neuropilin 1
ASPN	asporin
CTH	cystathionine gamma-lyase
GK	glycerol kinase
LTK	leukocyte receptor tyrosine kinase
NRTN	neurturin
PDCD5	programmed cell death 5
MT-CYB	mitochondrially encoded cytochrome b
CBS	cystathionine-beta-synthase
RCC1	regulator of chromosome condensation 1
MTNR1A	melatonin receptor 1A
BST1	bone marrow stromal cell antigen 1
RP4-568C11.4	

GZMA	granzyme A
CABIN1	calcineurin binding protein 1
JADE1	jade family PHD finger 1
PTPRN2	protein tyrosine phosphatase, receptor type N2
SERPINC1	serpin family C member 1
BMP2	bone morphogenetic protein 2
GIGYF2	GRB10 interacting GYF protein 2
LMO3	LIM domain only 3
HDAC4	histone deacetylase 4
OR10J3	olfactory receptor family 10 subfamily J member 3
ST3GAL1	ST3 beta-galactoside alpha-2,3-sialyltransferase 1
KLK4	kallikrein related peptidase 4
TRIQK	triple QxxK/R motif containing
CCDC178	coiled-coil domain containing 178
SARAF	store-operated calcium entry associated regulatory factor
LY96	lymphocyte antigen 96
ITPR2	inositol 1,4,5-trisphosphate receptor type 2
SSB	Sjogren syndrome antigen B
SLC11A1	solute carrier family 11 member 1
CTNNB1	catenin beta 1
DPP6	dipeptidyl peptidase like 6
IGSF10	immunoglobulin superfamily member 10
LRRC18	leucine rich repeat containing 18
WNT9A	Wnt family member 9A
IL3RA	interleukin 3 receptor subunit alpha

ESR2	estrogen receptor 2
CNGA4	cyclic nucleotide gated channel alpha 4
PEX6	peroxisomal biogenesis factor 6
CYP27A1	cytochrome P450 family 27 subfamily A member 1
PCDH20	protocadherin 20
DAW1	dynein assembly factor with WD repeats 1
SLC7A2	solute carrier family 7 member 2
TLX2	T-cell leukemia homeobox 2
EGR1	early growth response 1
ATG7	autophagy related 7
MUC3A	mucin 3A, cell surface associated
RAB24	RAB24, member RAS oncogene family
SALL3	spalt like transcription factor 3
GTDC1	glycosyltransferase like domain containing 1
ASAP2	ArfGAP with SH3 domain, ankyrin repeat and PH domain 2
DTWD2	DTW domain containing 2
GPER1	G protein-coupled estrogen receptor 1
CCNA2	cyclin A2
CYP39A1	cytochrome P450 family 39 subfamily A member 1
NGFR	nerve growth factor receptor
GDNF	glial cell derived neurotrophic factor
RAET1L	retinoic acid early transcript 1L
ATOH8	atonal bHLH transcription factor 8
ANO1	anoctamin 1
MAPKAPK2	mitogen-activated protein kinase-activated protein kinase 2

HBEGF	heparin binding EGF like growth factor
KLKB1	kallikrein B1
CCL1	C-C motif chemokine ligand 1
ATP2A2	ATPase sarcoplasmic/endoplasmic reticulum Ca2+ transporting 2
SLC2A6	solute carrier family 2 member 6
OPN3	opsin 3
AC006262.11	
UBE2E2	ubiquitin conjugating enzyme E2 E2
LOX	lysyl oxidase
CYBRD1	cytochrome b reductase 1
OR6X1	olfactory receptor family 6 subfamily X member 1
SVEP1	sushi, von Willebrand factor type A, EGF and pentraxin domain containing 1
OPRM1	opioid receptor mu 1
ANKFN1	ankyrin repeat and fibronectin type III domain containing 1
MICB	MHC class I polypeptide-related sequence B
ACVR2B	activin A receptor type 2B
GRP	gastrin releasing peptide
TLE2	transducin like enhancer of split 2
IRF9	interferon regulatory factor 9
TBCD	tubulin folding cofactor D
PLAGL1	PLAG1 like zinc finger 1
PAIP2B	poly(A) binding protein interacting protein 2B
BDKRB1	bradykinin receptor B1
CPN1	carboxypeptidase N subunit 1
CYP21A1P	cytochrome P450 family 21 subfamily A member 1, pseudogene

TRPM8	transient receptor potential cation channel subfamily M member 8
DNTT	DNA nucleotidylexotransferase
LRMP	lymphoid restricted membrane protein
FCAR	Fc fragment of IgA receptor
DONSON	downstream neighbor of SON
LALBA	lactalbumin alpha
GPX4	glutathione peroxidase 4
PPP1R12B	protein phosphatase 1 regulatory subunit 12B
STIL	SCL/TAL1 interrupting locus
IL21R	interleukin 21 receptor
KRT10	keratin 10
HSPG2	heparan sulfate proteoglycan 2
CPOX	coproporphyrinogen oxidase
ARHGDIB	Rho GDP dissociation inhibitor beta
APOBR	apolipoprotein B receptor
SPACA7	sperm acrosome associated 7
CD68	CD68 molecule
PAOX	polyamine oxidase (exo-N4-amino)
DCAF4	DDB1 and CUL4 associated factor 4
CTSC	cathepsin C
PRMT1	protein arginine methyltransferase 1
MAB21L3	mab-21 like 3
CEP44	centrosomal protein 44
AL365202.1	
ALDH7A1	aldehyde dehydrogenase 7 family member A1

QKI	QKI, KH domain containing RNA binding
IGFBP7	insulin like growth factor binding protein 7
NCAM2	neural cell adhesion molecule 2
CXXC4	CXXC finger protein 4
TCF7	transcription factor 7 (T-cell specific, HMG-box)
MMP19	matrix metallopeptidase 19
RCAN1	regulator of calcineurin 1
GRIP2	glutamate receptor interacting protein 2
EPS8L3	EPS8 like 3
PM20D1	peptidase M20 domain containing 1
MAPK8	mitogen-activated protein kinase 8
DCT	dopachrome tautomerase
GLA	galactosidase alpha
CLEC2B	C-type lectin domain family 2 member B
GLT1D1	glycosyltransferase 1 domain containing 1
FFAR2	free fatty acid receptor 2
HEATR9	HEAT repeat containing 9
SLC26A3	solute carrier family 26 member 3
PPOX	protoporphyrinogen oxidase
RIT1	Ras like without CAAX 1
LYST	lysosomal trafficking regulator
C21orf58	chromosome 21 open reading frame 58
SMTNL1	smoothelin like 1
RB1	RB transcriptional corepressor 1
TGFBI	transforming growth factor beta induced

PNPLA4	patatin like phospholipase domain containing 4
CD2	CD2 molecule
RSPH14	radial spoke head 14 homolog
NKX2-1	NK2 homeobox 1
ALKBH1	alkB homolog 1, histone H2A dioxygenase
NFKBIE	NFKB inhibitor epsilon
CDIP1	cell death inducing p53 target 1
MAP2K3	mitogen-activated protein kinase kinase 3
INS	insulin
ITGA1	integrin subunit alpha 1
IFNGR1	interferon gamma receptor 1
CYP24A1	cytochrome P450 family 24 subfamily A member 1
SERPINB4	serpin family B member 4
CCS	copper chaperone for superoxide dismutase
XIAP	X-linked inhibitor of apoptosis
TRPM7	transient receptor potential cation channel subfamily M member 7
BTK	Bruton tyrosine kinase
ZNF248	zinc finger protein 248
BGN	biglycan
MROH9	maestro heat like repeat family member 9
SLC16A7	solute carrier family 16 member 7
FAM184A	family with sequence similarity 184 member A
WFDC1	WAP four-disulfide core domain 1
CTNNAL1	catenin alpha like 1
PEBP1	phosphatidylethanolamine binding protein 1

SFTPC	surfactant protein C
MUC4	mucin 4, cell surface associated
KDR	kinase insert domain receptor
TP53AIP1	tumor protein p53 regulated apoptosis inducing protein 1
HTR2B	5-hydroxytryptamine receptor 2B
POU2F1	POU class 2 homeobox 1
NF1	neurofibromin 1
PPIA	peptidylprolyl isomerase A
KCNH3	potassium voltage-gated channel subfamily H member 3
JAKMIP1	janus kinase and microtubule interacting protein 1
CTSG	cathepsin G
T	T brachyury transcription factor
ANXA2	annexin A2
IL12RB2	interleukin 12 receptor subunit beta 2
C10orf95	chromosome 10 open reading frame 95
RUNX3	runt related transcription factor 3
MOCS1	molybdenum cofactor synthesis 1
ADCY10	adenylate cyclase 10, soluble
RP11-416L21.2	
PTGER4	prostaglandin E receptor 4
POR	cytochrome p450 oxidoreductase
ACVR1B	activin A receptor type 1B
SERTAD1	SERTA domain containing 1
SPI1	Spi-1 proto-oncogene

PPP6R3	protein phosphatase 6 regulatory subunit 3
DAP3	death associated protein 3
PLXND1	plexin D1
C7	complement C7
SNRNP27	small nuclear ribonucleoprotein U4/U6.U5 subunit 27
NSD1	nuclear receptor binding SET domain protein 1
PTPN1	protein tyrosine phosphatase, non-receptor type 1
SEMA4A	semaphorin 4A
C2orf81	chromosome 2 open reading frame 81
UMOD	uromodulin
TEKT4	tektin 4
EMB	embigin
GTF2H4	general transcription factor IIH subunit 4
CD79A	CD79a molecule
UTS2R	urotensin 2 receptor
GAST	gastrin
HCG22	HLA complex group 22
DNAH9	dynein axonemal heavy chain 9
PGLYRP1	peptidoglycan recognition protein 1
CBWD1	COBW domain containing 1
MLLT1	MLLT1, super elongation complex subunit
WNT1	Wnt family member 1
NOX5	NADPH oxidase 5
AIDA	axin interactor, dorsalization associated
PDGFB	platelet derived growth factor subunit B

ACP5	acid phosphatase 5, tartrate resistant
F11R	F11 receptor
FOXK2	forkhead box K2
LOR	loricrin
ROCK2	Rho associated coiled-coil containing protein kinase 2
OR10A4	olfactory receptor family 10 subfamily A member 4
TFAP4	transcription factor AP-4
SIAH3	siah E3 ubiquitin protein ligase family member 3
CAAP1	caspase activity and apoptosis inhibitor 1
NR1H4	nuclear receptor subfamily 1 group H member 4
LUM	lumican
TIMP3	TIMP metallopeptidase inhibitor 3
DPYS	dihydropyrimidinase
PRSS35	protease, serine 35
CSMD3	CUB and Sushi multiple domains 3
BIRC2	baculoviral IAP repeat containing 2
CSF2RA	colony stimulating factor 2 receptor alpha subunit
RAB1A	RAB1A, member RAS oncogene family
PSMA3	proteasome subunit alpha 3
HPN	hepsin
CDKN2A	cyclin dependent kinase inhibitor 2A
TGFBR3	transforming growth factor beta receptor 3
IKZF1	IKAROS family zinc finger 1
BAALC	brain and acute leukemia, cytoplasmic
LINC01132	long intergenic non-protein coding RNA 1132

BCAS2P2	breast carcinoma amplified sequence 2 pseudogene 2
ZMYND12	zinc finger MYND-type containing 12
RP11-93B14.9	
ADAMTS12	ADAM metallopeptidase with thrombospondin type 1 motif 12
HGS	hepatocyte growth factor-regulated tyrosine kinase substrate
GHRL	ghrelin and obestatin prepropeptide
PSMC6	proteasome 26S subunit, ATPase 6
RELA	RELA proto-oncogene, NF-kB subunit
HMOX2	heme oxygenase 2
FCGBP	Fc fragment of IgG binding protein
ASPHD2	aspartate beta-hydroxylase domain containing 2
CD5	CD5 molecule
CPA3	carboxypeptidase A3
DCTN3	dynactin subunit 3
IDS	iduronate 2-sulfatase
ZGLP1	zinc finger, GATA-like protein 1
SPRR2C	small proline rich protein 2C (pseudogene)
AOC2	amine oxidase, copper containing 2
IRF3	interferon regulatory factor 3
ACP1	acid phosphatase 1, soluble
CXCL16	C-X-C motif chemokine ligand 16
P2RY2	purinergic receptor P2Y2
NLRP12	NLR family pyrin domain containing 12
IGHG2	immunoglobulin heavy constant gamma 2 (G2m marker)

TRIM55	tripartite motif containing 55
HGSNAT	heparan-alpha-glucosaminide N-acetyltransferase
EXOC3L4	exocyst complex component 3 like 4
EMSY	EMSY, BRCA2 interacting transcriptional repressor
DGKH	diacylglycerol kinase eta
PARPBP	PARP1 binding protein
BCL2	BCL2, apoptosis regulator
RP11-333O1.1	
BCL6	B-cell CLL/lymphoma 6
REXO1L1P	REX1, RNA exonuclease 1 homolog-like 1, pseudogene
CYP2D6	cytochrome P450 family 2 subfamily D member 6
KRT14	keratin 14
FMO5	flavin containing monooxygenase 5
IRAK4	interleukin 1 receptor associated kinase 4
CFLAR-AS1	CFLAR antisense RNA 1
CD33	CD33 molecule
SLC7A6	solute carrier family 7 member 6
YY1	YY1 transcription factor
ZFAT	zinc finger and AT-hook domain containing
LMCD1	LIM and cysteine rich domains 1
AMY1A	amylase, alpha 1A (salivary)
CASR	calcium sensing receptor
CTSK	cathepsin K
SDK2	sidekick cell adhesion molecule 2

TNXB	tenascin XB
CD55	CD55 molecule (Cromer blood group)
RUNX1	runt related transcription factor 1
KMO	kynurenine 3-monooxygenase
TBCA	tubulin folding cofactor A
TPH1	tryptophan hydroxylase 1
CLDN9	claudin 9
CALCR	calcitonin receptor
DIS3L	DIS3 like exosome 3'-5' exoribonuclease
RP11-1399P15.1	
GDF15	growth differentiation factor 15
MMP10	matrix metallopeptidase 10
MAPK1	mitogen-activated protein kinase 1
CYP3A5	cytochrome P450 family 3 subfamily A member 5
TFCP2L1	transcription factor CP2-like 1
RIMS2	regulating synaptic membrane exocytosis 2
BPIFA1	BPI fold containing family A member 1
ALLC	allantoicase
S100A6	S100 calcium binding protein A6
PPBP	pro-platelet basic protein
MMACHC	methylmalonic aciduria (cobalamin deficiency) cblC type, with homocystinuria
KCNE4	potassium voltage-gated channel subfamily E regulatory subunit 4
FADS2	fatty acid desaturase 2
ADI1	acireductone dioxygenase 1

BMPR2	bone morphogenetic protein receptor type 2
DLAT	dihydrolipoamide S-acetyltransferase
NR4A1	nuclear receptor subfamily 4 group A member 1
STUB1	STIP1 homology and U-box containing protein 1
PSIP1	PC4 and SFRS1 interacting protein 1
PTGER3	prostaglandin E receptor 3
JAG1	jagged 1
FAM13C	family with sequence similarity 13 member C
RBMS3	RNA binding motif single stranded interacting protein 3
CEBPZ	CCAAT/enhancer binding protein zeta
RP11-149I23.3	
PRRX2	paired related homeobox 2
PUM3	pumilio RNA binding family member 3
AP001372.2	
GAD1	glutamate decarboxylase 1
GSR	glutathione-disulfide reductase
RAC2	ras-related C3 botulinum toxin substrate 2 (rho family, small GTP binding protein Rac2)
P2RY6	pyrimidinergic receptor P2Y6
NOV	nephroblastoma overexpressed
NRXN1	neurexin 1
SLC39A2	solute carrier family 39 member 2
HPS1	HPS1, biogenesis of lysosomal organelles complex 3 subunit 1
ABCB1	ATP binding cassette subfamily B member 1

FAXDC2	fatty acid hydroxylase domain containing 2
SH2D1B	SH2 domain containing 1B
SLC6A11	solute carrier family 6 member 11
CYP27B1	cytochrome P450 family 27 subfamily B member 1
PTPRE	protein tyrosine phosphatase, receptor type E
CYFIP2	cytoplasmic FMR1 interacting protein 2
MED24	mediator complex subunit 24
PNMT	phenylethanolamine N-methyltransferase
AOC3	amine oxidase, copper containing 3
COPA	coatomer protein complex subunit alpha
HM13	histocompatibility minor 13
NMU	neuromedin U
TRAF1	TNF receptor associated factor 1
SLC39A8	solute carrier family 39 member 8
MYL9	myosin light chain 9
RP11-339N8.1	
COPS8	COP9 signalosome subunit 8
RP11-483P21.2	
05-Sep	septin 5
ZDHHC2	zinc finger DHHC-type containing 2
LMAN1	lectin, mannose binding 1
FAM134B	family with sequence similarity 134 member B
SERPINF1	serpin family F member 1

NPC1L1	NPC1 like intracellular cholesterol transporter 1
CCT5	chaperonin containing TCP1 subunit 5
FMO2	flavin containing monooxygenase 2
RP11-402P6.9	
ZBTB32	zinc finger and BTB domain containing 32
TALDO1	transaldolase 1
MLLT3	MLLT3, super elongation complex subunit
ZNF74	zinc finger protein 74
HCRT	hypocretin neuropeptide precursor
ZBTB38	zinc finger and BTB domain containing 38
GRB10	growth factor receptor bound protein 10
CXorf57	chromosome X open reading frame 57
CASC2	cancer susceptibility candidate 2 (non-protein coding)
CARD16	caspase recruitment domain family member 16
KRT8	keratin 8
GLRB	glycine receptor beta
GJA4	gap junction protein alpha 4
ITGA2B	integrin subunit alpha 2b
NPY6R	neuropeptide Y receptor Y6 (pseudogene)
PDE4DIP	phosphodiesterase 4D interacting protein
GRK2	G protein-coupled receptor kinase 2
PIK3C3	phosphatidylinositol 3-kinase catalytic subunit type 3
RAB3B	RAB3B, member RAS oncogene family
DDT	D-dopachrome tautomerase

C4orf50	chromosome 4 open reading frame 50
LENG9	leukocyte receptor cluster member 9
ABCA1	ATP binding cassette subfamily A member 1
EVA1B	eva-1 homolog B
CFAP161	cilia and flagella associated protein 161
BEAN1	brain expressed, associated with NEDD4, 1
CAMP	cathelicidin antimicrobial peptide
IQGAP1	IQ motif containing GTPase activating protein 1
DSE	dermatan sulfate epimerase
FGF7	fibroblast growth factor 7
FUS	FUS RNA binding protein
WDR46	WD repeat domain 46
SMAP2	small ArfGAP2
SPTSSB	serine palmitoyltransferase small subunit B
ZUFSP	zinc finger with UFM1 specific peptidase domain
RP11-734K21.5	
ACSS1	acyl-CoA synthetase short-chain family member 1
PHEX	phosphate regulating endopeptidase homolog, X-linked
PLEKHF2	pleckstrin homology and FYVE domain containing 2
L3MBTL4	l(3)mbt-like 4 (Drosophila)
FHOD3	formin homology 2 domain containing 3
PITX2	paired like homeodomain 2
RP11-54O7.17	

CHD6	chromodomain helicase DNA binding protein 6
CNTF	ciliary neurotrophic factor
RBFOX2	RNA binding protein, fox-1 homolog 2
GIMAP5	GTPase, IMAP family member 5
GIMAP4	GTPase, IMAP family member 4
INSIG2	insulin induced gene 2
RAF1	Raf-1 proto-oncogene, serine/threonine kinase
IRX3	iroquois homeobox 3
FCER1G	Fc fragment of IgE receptor Ig
ELAVL1	ELAV like RNA binding protein 1
LRP2	LDL receptor related protein 2
PIK3CB	phosphatidylinositol-4,5-bisphosphate 3-kinase catalytic subunit beta
EDA	ectodysplasin A
AMN	amnion associated transmembrane protein
FGF1	fibroblast growth factor 1
GRK5	G protein-coupled receptor kinase 5
CRISPLD2	cysteine rich secretory protein LCCL domain containing 2
PYCARD	PYD and CARD domain containing
AC093724.2	
PRTN3	proteinase 3
LRRC73	leucine rich repeat containing 73
STAG3	stromal antigen 3
ATG9B	autophagy related 9B
LMBRD1	LMBR1 domain containing 1

RP11-890B15.2	
SDS	serine dehydratase
DES	desmin
SLC16A2	solute carrier family 16 member 2
CDCA7	cell division cycle associated 7
HLA-DPB1	major histocompatibility complex, class II, DP beta 1
CTF1	cardiotrophin 1
SMIM21	small integral membrane protein 21
HBE1	hemoglobin subunit epsilon 1
EFCAB11	EF-hand calcium binding domain 11
CTC-241N9.1	
RAB30	RAB30, member RAS oncogene family
CACNG4	calcium voltage-gated channel auxiliary subunit gamma 4
CD58	CD58 molecule
C11orf52	chromosome 11 open reading frame 52
NPAT	nuclear protein, coactivator of histone transcription
SLC25A20	solute carrier family 25 member 20
GAP43	growth associated protein 43
ALPK1	alpha kinase 1
USO1	USO1 vesicle transport factor
SPRN	shadow of prion protein homolog (zebrafish)
SEC14L2	SEC14 like lipid binding 2
CEBPD	CCAAT/enhancer binding protein delta

RP11-30O15.1	
PDC	phosducin
MUT	methylmalonyl-CoA mutase
CAND2	cullin associated and neddylation dissociated 2 (putative)
CNRIP1	cannabinoid receptor interacting protein 1
LINC01515	long intergenic non-protein coding RNA 1515
ERAP2	endoplasmic reticulum aminopeptidase 2
NANOS2	nanos C2HC-type zinc finger 2
CORT	cortistatin
TPRXL	tetrapeptide repeat homeobox like
UMODL1-AS1	UMODL1 antisense RNA 1
CCL25	C-C motif chemokine ligand 25
KIFC1	kinesin family member C1
MAS1	MAS1 proto-oncogene, G protein-coupled receptor
MC5R	melanocortin 5 receptor
TEF	TEF, PAR bZIP transcription factor
RUNX1T1	RUNX1 translocation partner 1
CHML	CHM like, Rab escort protein 2
HRG	histidine rich glycoprotein
PTGES	prostaglandin E synthase
CYCS	cytochrome c, somatic
CALR	calreticulin
PAEP	progestagen associated endometrial protein

CHRNA2	cholinergic receptor nicotinic alpha 2 subunit
HS2ST1	heparan sulfate 2-O-sulfotransferase 1
MB21D1	Mab-21 domain containing 1
RP11-856M7.6	
CD164L2	CD164 molecule like 2
ADAMTS1	ADAM metallopeptidase with thrombospondin type 1 motif 1
DNASE1L3	deoxyribonuclease 1 like 3
COL3A1	collagen type III alpha 1 chain
CLEC10A	C-type lectin domain family 10 member A
NUFIP2	NUFIP2, FMR1 interacting protein 2
HIST1H4H	histone cluster 1, H4h
ZFR	zinc finger RNA binding protein
FRMD6	FERM domain containing 6
NAAA	N-acylethanolamine acid amidase
PIM1	Pim-1 proto-oncogene, serine/threonine kinase
CDH5	cadherin 5
ACVR1	activin A receptor type 1
NNT	nicotinamide nucleotide transhydrogenase
POLR2A	RNA polymerase II subunit A
SLCO2B1	solute carrier organic anion transporter family member 2B1
SFTPA2	surfactant protein A2
NRG2	neuregulin 2
SIPA1L1	signal induced proliferation associated 1 like 1

CTD-2047H16.4	
CLEC6A	C-type lectin domain family 6 member A
USP51	ubiquitin specific peptidase 51
KIAA1257	KIAA1257
FFAR3	free fatty acid receptor 3
INHBA	inhibin beta A subunit
FCGR1A	Fc fragment of IgG receptor Ia
EBI3	Epstein-Barr virus induced 3
MUC16	mucin 16, cell surface associated
LTBR	lymphotoxin beta receptor
SUSD3	sushi domain containing 3
SETD2	SET domain containing 2
GPR155	G protein-coupled receptor 155
TNFRSF1B	TNF receptor superfamily member 1B
BPHL	biphenyl hydrolase like
MAP3K1	mitogen-activated protein kinase kinase kinase 1
IL20RA	interleukin 20 receptor subunit alpha
IL20RB	interleukin 20 receptor subunit beta
ORMDL1	ORMDL sphingolipid biosynthesis regulator 1
AHSP	alpha hemoglobin stabilizing protein
TAC4	tachykinin 4 (hemokinin)
GZMK	granzyme K
CYLD	CYLD lysine 63 deubiquitinase
CYP1B1	cytochrome P450 family 1 subfamily B member 1

HTR3A	5-hydroxytryptamine receptor 3A
UGT1A3	UDP glucuronosyltransferase family 1 member A3
ZIC3	Zic family member 3
STMN4	stathmin 4
TCTA	T-cell leukemia translocation altered
PRDX5	peroxiredoxin 5
PSMD3	proteasome 26S subunit, non-ATPase 3
PRMT5	protein arginine methyltransferase 5
ADIPOQ	adiponectin, C1Q and collagen domain containing
GATA1	GATA binding protein 1
HAND1	heart and neural crest derivatives expressed 1
PRMT2	protein arginine methyltransferase 2
ORMDL2	ORMDL sphingolipid biosynthesis regulator 2
ILK	integrin linked kinase
TAB1	TGF-beta activated kinase 1 (MAP3K7) binding protein 1
GRAP2	GRB2-related adaptor protein 2
CBR1	carbonyl reductase 1
MAOA	monoamine oxidase A
UGT1A1	UDP glucuronosyltransferase family 1 member A1
MRI1	methylthioribose-1-phosphate isomerase 1
RAP1GAP	RAP1 GTPase activating protein
TAC3	tachykinin 3
NAP1L4	nucleosome assembly protein 1 like 4
PXN	paxillin
ANXA4	annexin A4

ADAM17	ADAM metallopeptidase domain 17
IP6K3	inositol hexakisphosphate kinase 3
NOS1	nitric oxide synthase 1
RP11-38L15.3	
FTO	fat mass and obesity associated
F2	coagulation factor II, thrombin
RXFP1	relaxin/insulin like family peptide receptor 1
C1QTNF1	C1q and tumor necrosis factor related protein 1
OCLN	occludin
FAM13A	family with sequence similarity 13 member A
BACH2	BTB domain and CNC homolog 2
CARD10	caspase recruitment domain family member 10
ERICH5	glutamate rich 5
RP11-661D19.3	
RP11-67L2.2	
AQP7	aquaporin 7
FOS	Fos proto-oncogene, AP-1 transcription factor subunit
RNPEP	arginyl aminopeptidase
TMEM11	transmembrane protein 11
TGFB3	transforming growth factor beta 3
HLA-K	major histocompatibility complex, class I, K (pseudogene)
LINC00997	long intergenic non-protein coding RNA 997
CD36	CD36 molecule

ASCC1	activating signal cointegrator 1 complex subunit 1
HPRT1	hypoxanthine phosphoribosyltransferase 1
CFD	complement factor D
CAMK4	calcium/calmodulin dependent protein kinase IV
GHR	growth hormone receptor
TMEM143	transmembrane protein 143
SUOX	sulfite oxidase
PIR	pirin
STAG2	stromal antigen 2
NUCB2	nucleobindin 2
NR1H3	nuclear receptor subfamily 1 group H member 3
PVALB	parvalbumin
ZNF862	zinc finger protein 862
SCT	secretin
SLC25A10	solute carrier family 25 member 10
KRT5	keratin 5
FHL2	four and a half LIM domains 2
FNTB	farnesyltransferase, CAAX box, beta
RHO	rhodopsin
TBP	TATA-box binding protein
IL6ST	interleukin 6 signal transducer
LSM14B	LSM family member 14B
ATL3	atlastin GTPase 3
ACADM	acyl-CoA dehydrogenase, C-4 to C-12 straight chain
GSC	goosecoid homeobox

EP300	E1A binding protein p300
KPNA2	karyopherin subunit alpha 2
SKAP2	src kinase associated phosphoprotein 2
SGCA	sarcoglycan alpha
EFCAB14	EF-hand calcium binding domain 14
KLK3	kallikrein related peptidase 3
OXGR1	oxoglutarate receptor 1
FAM27C	family with sequence similarity 27 member C
10-Mar	membrane associated ring-CH-type finger 10
FAM81A	family with sequence similarity 81 member A
HCG4B	HLA complex group 4B (non-protein coding)
AKT1	AKT serine/threonine kinase 1
GGT1	gamma-glutamyltransferase 1
CYP2E1	cytochrome P450 family 2 subfamily E member 1
LINC01137	long intergenic non-protein coding RNA 1137
AC009133.12	
MYC	v-myc avian myelocytomatosis viral oncogene homolog
RP11-119D9.1	
NAGA	alpha-N-acetylgalactosaminidase
OR7E122P	olfactory receptor family 7 subfamily E member 122 pseudogene
SPRR2A	small proline rich protein 2A
SLC22A4	solute carrier family 22 member 4
FAM131A	family with sequence similarity 131 member A
IL17RC	interleukin 17 receptor C

PROC	protein C, inactivator of coagulation factors Va and VIIIa
CD207	CD207 molecule
C3	complement C3
IL31RA	interleukin 31 receptor A
HMGN4	high mobility group nucleosomal binding domain 4
TRPC6	transient receptor potential cation channel subfamily C member 6
AGT	angiotensinogen
ALOX12	arachidonate 12-lipoxygenase, 12S type
ATP5A1P5	ATP synthase, H+ transporting, mitochondrial F1 complex, alpha subunit 1 pseudogene 5
GBP7	guanylate binding protein 7
AC002511.2	
TMEM45A	transmembrane protein 45A
ANKRD33B	ankyrin repeat domain 33B
EPAS1	endothelial PAS domain protein 1
MCIDAS	multiciliate differentiation and DNA synthesis associated cell cycle protein
NXPH1	neurexophilin 1
CPS1	carbamoyl-phosphate synthase 1
CNIH2	cornichon family AMPA receptor auxiliary protein 2
LINC00890	long intergenic non-protein coding RNA 890
RP11-161H23.9	
G6PD	glucose-6-phosphate dehydrogenase
ERP27	endoplasmic reticulum protein 27
LRG1	leucine rich alpha-2-glycoprotein 1

FOLR2	folate receptor beta
VIPR2	vasoactive intestinal peptide receptor 2
KNG1	kininogen 1
WFDC2	WAP four-disulfide core domain 2
CA1	carbonic anhydrase 1
CXCL6	C-X-C motif chemokine ligand 6
PROCR	protein C receptor
RP11-640L9.2	
TINCR	tissue differentiation-inducing non-protein coding RNA
ABCC6P2	ATP binding cassette subfamily C member 6 pseudogene 2
RP11-640L9.1	
EPCAM	epithelial cell adhesion molecule
LRRIQ3	leucine rich repeats and IQ motif containing 3
ZBED5	zinc finger BED-type containing 5
RP11-345J18.2	
COL28A1	collagen type XXVIII alpha 1 chain
PARK7	Parkinsonism associated deglycase
EIF6	eukaryotic translation initiation factor 6
UCHL1	ubiquitin C-terminal hydrolase L1
NTRK3	neurotrophic receptor tyrosine kinase 3
PRDX6	peroxiredoxin 6
PRL	prolactin

CPD	carboxypeptidase D
ICAM3	intercellular adhesion molecule 3
FRMD4A	FERM domain containing 4A
LINC00964	long intergenic non-protein coding RNA 964
RP11-46H11.3	
C2orf66	chromosome 2 open reading frame 66
GPR85	G protein-coupled receptor 85
IVNS1ABP	influenza virus NS1A binding protein
TNFSF15	tumor necrosis factor superfamily member 15
GNRHR	gonadotropin releasing hormone receptor
CREBBP	CREB binding protein
IL1RAP	interleukin 1 receptor accessory protein
AIFM1	apoptosis inducing factor, mitochondria associated 1
PDE7A	phosphodiesterase 7A
ADH1B	alcohol dehydrogenase 1B (class I), beta polypeptide
P4HB	prolyl 4-hydroxylase subunit beta
SCARA3	scavenger receptor class A member 3
DDX41	DEAD-box helicase 41
RP11-368I7.4	
IGHG3	immunoglobulin heavy constant gamma 3 (G3m marker)
SERPINA5	serpin family A member 5
PTCHD3	patched domain containing 3
AQP4	aquaporin 4

GNPDA2	glucosamine-6-phosphate deaminase 2
IGHG1	immunoglobulin heavy constant gamma 1 (G1m marker)
TGFBR1	transforming growth factor beta receptor 1
SIGLEC14	sialic acid binding Ig like lectin 14
RFX1	regulatory factor X1
SLC19A1	solute carrier family 19 member 1
KRT13	keratin 13
AKR1C1	aldo-keto reductase family 1 member C1
TMX1	thioredoxin related transmembrane protein 1
SNRNP35	small nuclear ribonucleoprotein U11/U12 subunit 35
PRNP	prion protein
ATF6	activating transcription factor 6
ZBTB12	zinc finger and BTB domain containing 12
MORN4	MORN repeat containing 4
RRP15	ribosomal RNA processing 15 homolog
BNIP3P11	BCL2 interacting protein 3 pseudogene 11
CPNE5	copine 5
RASA1	RAS p21 protein activator 1
ERBB2	erb-b2 receptor tyrosine kinase 2
GATA2	GATA binding protein 2
DEXI	Dexi homolog
AGER	advanced glycosylation end-product specific receptor
PLCL1	phospholipase C like 1
PDE10A	phosphodiesterase 10A
CHGA	chromogranin A

ROBO1	roundabout guidance receptor 1
HNF1A	HNF1 homeobox A
MEN1	menin 1
FLT3	fms related tyrosine kinase 3
NRF1	nuclear respiratory factor 1
MS4A3	membrane spanning 4-domains A3
CHST1	carbohydrate sulfotransferase 1
SULT2A1	sulfotransferase family 2A member 1
HPX	hemopexin
GGACT	gamma-glutamylamine cyclotransferase
DHX9	DEAH-box helicase 9
LPP	LIM domain containing preferred translocation partner in lipoma
ITLN2	intelectin 2
KL	klotho
FSCN1	fascin actin-bundling protein 1
SPINT2	serine peptidase inhibitor, Kunitz type 2
KRTAP1-1	keratin associated protein 1-1
PSMA7	proteasome subunit alpha 7
C14orf166	chromosome 14 open reading frame 166
FAM168B	family with sequence similarity 168 member B
LINC00665	long intergenic non-protein coding RNA 665
RNASE1	ribonuclease A family member 1, pancreatic
PLA2G4C	phospholipase A2 group IVC
RAB43	RAB43, member RAS oncogene family
AQP4-AS1	AQP4 antisense RNA 1

HOXB2	homeobox B2
RP1-228H13.5	
PLK1	polo like kinase 1
WT1	Wilms tumor 1
RGS3	regulator of G-protein signaling 3
RHCE	Rh blood group CcEe antigens
ART1	ADP-ribosyltransferase 1
EBF1	early B-cell factor 1
CLYBL	citrate lyase beta like
CSK	c-src tyrosine kinase
SLC30A4	solute carrier family 30 member 4
VIM	vimentin
CENPF	centromere protein F
ACTR3B	ARP3 actin related protein 3 homolog B
GIPC2	GIPC PDZ domain containing family member 2
KEAP1	kelch like ECH associated protein 1
OXTR	oxytocin receptor
ARL10	ADP ribosylation factor like GTPase 10
PPM1E	protein phosphatase, Mg2+/Mn2+ dependent 1E
TPRG1	tumor protein p63 regulated 1
MTSS1	MTSS1, I-BAR domain containing
TRIT1	tRNA isopentenyltransferase 1
FBXW11	F-box and WD repeat domain containing 11
DOCK8	dedicator of cytokinesis 8

MGST2	microsomal glutathione S-transferase 2
VLDLR	very low density lipoprotein receptor
KRT37	keratin 37
TNFRSF11B	TNF receptor superfamily member 11b
CD70	CD70 molecule
ABCC2	ATP binding cassette subfamily C member 2
AKR1C3	aldo-keto reductase family 1 member C3
TNFSF8	tumor necrosis factor superfamily member 8
TSC1	tuberous sclerosis 1
ERICH2	glutamate rich 2
PDGFRL	platelet derived growth factor receptor like
FOLR1	folate receptor 1
FXN	frataxin
MOCS3	molybdenum cofactor synthesis 3
HSPA5	heat shock protein family A (Hsp70) member 5
CIB2	calcium and integrin binding family member 2
TMCO5A	transmembrane and coiled-coil domains 5A
PAQR7	progestin and adipoQ receptor family member 7
MYH16	myosin heavy chain 16 pseudogene
DOK7	docking protein 7
KLK5	kallikrein related peptidase 5
DKK1	dickkopf WNT signaling pathway inhibitor 1
PPEF2	protein phosphatase with EF-hand domain 2
PLN	phospholamban
JAML	junction adhesion molecule like

DNMT3A	DNA methyltransferase 3 alpha
DAPK3	death associated protein kinase 3
XPNPEP1	X-prolyl aminopeptidase 1
NEB	nebulin
SIGLEC10	sialic acid binding Ig like lectin 10
SRGN	serglycin
TIGIT	T-cell immunoreceptor with Ig and ITIM domains
KLRK1	killer cell lectin like receptor K1
VIT	vitrin
GNPDA1	glucosamine-6-phosphate deaminase 1
C9orf24	chromosome 9 open reading frame 24
FPR1	formyl peptide receptor 1
SUV39H1	suppressor of variegation 3-9 homolog 1
TIA1	TIA1 cytotoxic granule-associated RNA binding protein
EFCAB5	EF-hand calcium binding domain 5
RN7SL834P	RNA, 7SL, cytoplasmic 834, pseudogene
SLC6A4	solute carrier family 6 member 4
CTD-2047H16.3	
RP3-523K23.2	
WBSCR17	Williams-Beuren syndrome chromosome region 17
KIAA1211	KIAA1211
RPARP-AS1	RPARP antisense RNA 1
IL18RAP	interleukin 18 receptor accessory protein

PTPRU	protein tyrosine phosphatase, receptor type U
RP11-177C12.1	
KCNQ5	potassium voltage-gated channel subfamily Q member 5
PCBD1	pterin-4 alpha-carbinolamine dehydratase 1
INSR	insulin receptor
ZP3	zona pellucida glycoprotein 3
SATB1	SATB homeobox 1
PNP	purine nucleoside phosphorylase
TREM2	triggering receptor expressed on myeloid cells 2
LILRB4	leukocyte immunoglobulin like receptor B4
DBT	dihydrolipoamide branched chain transacylase E2
ERCC8	ERCC excision repair 8, CSA ubiquitin ligase complex subunit
PSEN2	presenilin 2
DUSP3	dual specificity phosphatase 3
EPPIN	epididymal peptidase inhibitor
LINC00623	long intergenic non-protein coding RNA 623
RP11-620J15.3	
GIPR	gastric inhibitory polypeptide receptor
GALT	galactose-1-phosphate uridylyltransferase
GABRQ	gamma-aminobutyric acid type A receptor theta subunit
ZNF382	zinc finger protein 382
CBX7	chromobox 7
IFNL4	interferon lambda 4 (gene/pseudogene)

RASD1	ras related dexamethasone induced 1
TFF1	trefoil factor 1
TXNRD3	thioredoxin reductase 3
EPB41L3	erythrocyte membrane protein band 4.1 like 3
MATN1	matrilin 1, cartilage matrix protein
ACPP	acid phosphatase, prostate
BTLA	B and T lymphocyte associated
FUCA1	fucosidase, alpha-L- 1, tissue
TYK2	tyrosine kinase 2
MTA2	metastasis associated 1 family member 2
NPY4R	neuropeptide Y receptor Y4
SETDB2	SET domain bifurcated 2
CALCB	calcitonin related polypeptide beta
ENPEP	glutamyl aminopeptidase
SGK1	serum/glucocorticoid regulated kinase 1
DLL4	delta like canonical Notch ligand 4
PDXK	pyridoxal (pyridoxine, vitamin B6) kinase
CERS2	ceramide synthase 2
PLD1	phospholipase D1
KLF5	Kruppel like factor 5
STX4	syntaxin 4
LVRN	laeverin
PDE5A	phosphodiesterase 5A
GPR65	G protein-coupled receptor 65
PKD1P6	polycystin 1, transient receptor potential channel interacting pseudogene 6

SIGLEC1	sialic acid binding Ig like lectin 1
C2CD4A	C2 calcium dependent domain containing 4A
ZNF215	zinc finger protein 215
ZC2HC1C	zinc finger C2HC-type containing 1C
FBXO27	F-box protein 27
CNR2	cannabinoid receptor 2
FANCC	Fanconi anemia complementation group C
IK	IK cytokine, down-regulator of HLA II
CTSH	cathepsin H
PPRC1	peroxisome proliferator-activated receptor gamma, coactivator-related 1
TAS2R14	taste 2 receptor member 14
QDPR	quinoid dihydropteridine reductase
LSR	lipolysis stimulated lipoprotein receptor
CHI3L2	chitinase 3 like 2
DDAH2	dimethylarginine dimethylaminohydrolase 2
GSTT2B	glutathione S-transferase theta 2B (gene/pseudogene)
DPCR1	diffuse panbronchiolitis critical region 1
LELP1	late cornified envelope like proline rich 1
C4A	complement C4A (Rodgers blood group)
HSPA1B	heat shock protein family A (Hsp70) member 1B
FUCA2	fucosidase, alpha-L- 2, plasma
NOTCH2	notch 2
PSD4	pleckstrin and Sec7 domain containing 4
FCN2	ficolin 2
PTK2	protein tyrosine kinase 2

SPRED1	sprouty related EVH1 domain containing 1
ACD	adrenocortical dysplasia homolog
NFKBIB	NFKB inhibitor beta
ST5	suppression of tumorigenicity 5
CYLC1	cylicin 1
CELA1	chymotrypsin like elastase family member 1
INA	internexin neuronal intermediate filament protein alpha
LTB4R2	leukotriene B4 receptor 2
TNFRSF14	TNF receptor superfamily member 14
ZNRD1	zinc ribbon domain containing 1
EFS	embryonal Fyn-associated substrate
RAB27A	RAB27A, member RAS oncogene family
KCTD7	potassium channel tetramerization domain containing 7
TMEM217	transmembrane protein 217
KNOP1	lysine rich nucleolar protein 1
C16orf54	chromosome 16 open reading frame 54
AR	androgen receptor
MINA	MYC induced nuclear antigen
PPP1R42	protein phosphatase 1 regulatory subunit 42
RAB3A	RAB3A, member RAS oncogene family
CH17-189H20.1	
NOMO1	NODAL modulator 1
CHDH	choline dehydrogenase
CHRNA5	cholinergic receptor nicotinic alpha 5 subunit

OBSCN	obscurin, cytoskeletal calmodulin and titin-interacting RhoGEF
SLC38A5	solute carrier family 38 member 5
SMN1	survival of motor neuron 1, telomeric
ZMYM2	zinc finger MYM-type containing 2
FOSL1	FOS like 1, AP-1 transcription factor subunit
LCN1	lipocalin 1
HYAL1	hyaluronoglucosaminidase 1
KLRF1	killer cell lectin like receptor F1
CD9	CD9 molecule
PIN1	peptidylprolyl cis/trans isomerase, NIMA-interacting 1
MTM1	myotubularin 1
NFKBIL1	NFKB inhibitor like 1
TBK1	TANK binding kinase 1
RPLP0	ribosomal protein lateral stalk subunit P0
SCGB1D4	secretoglobin family 1D member 4
SPATA13	spermatogenesis associated 13
PAF1	PAF1 homolog, Paf1/RNA polymerase II complex component
RUFY4	RUN and FYVE domain containing 4
MPZ	myelin protein zero
MYH9	myosin heavy chain 9
NFATC4	nuclear factor of activated T-cells 4
SULT1A3	sulfotransferase family 1A member 3
PPP1R14A	protein phosphatase 1 regulatory inhibitor subunit 14A
KCNS3	potassium voltage-gated channel modifier subfamily S member 3
PGLS	6-phosphogluconolactonase

CHRNA3	cholinergic receptor nicotinic alpha 3 subunit
RND3	Rho family GTPase 3
EXOSC3	exosome component 3
SLC23A1	solute carrier family 23 member 1
TNFRSF10B	TNF receptor superfamily member 10b
ABCB11	ATP binding cassette subfamily B member 11
PTN	pleiotrophin
TEX21P	testis expressed 21, pseudogene
HACE1	HECT domain and ankyrin repeat containing E3 ubiquitin protein ligase 1
CYGB	cytoglobin
SLC5A8	solute carrier family 5 member 8
EBF4	early B-cell factor 4
RP11-144A16.8	
CSF3R	colony stimulating factor 3 receptor
GSTM3	glutathione S-transferase mu 3
EPHA3	EPH receptor A3
FAM21C	family with sequence similarity 21 member C
CYP11A1	cytochrome P450 family 11 subfamily A member 1
ALDH1A1	aldehyde dehydrogenase 1 family member A1
AURKA	aurora kinase A
NOC3L	NOC3 like DNA replication regulator
UQCRC2	ubiquinol-cytochrome c reductase core protein II
G6PC3	glucose-6-phosphatase catalytic subunit 3
S100A16	S100 calcium binding protein A16

NEGR1	neuronal growth regulator 1
PRF1	perforin 1
NRG1	neuregulin 1
FAR2	fatty acyl-CoA reductase 2
HSPA12B	heat shock protein family A (Hsp70) member 12B
FAIM2	Fas apoptotic inhibitory molecule 2
SLC30A3	solute carrier family 30 member 3
PLCB1	phospholipase C beta 1
HNF1B	HNF1 homeobox B
PHGDH	phosphoglycerate dehydrogenase
PSMB3	proteasome subunit beta 3
TRAF6	TNF receptor associated factor 6
SLC25A16	solute carrier family 25 member 16
ERO1B	endoplasmic reticulum oxidoreductase 1 beta
NR1D2	nuclear receptor subfamily 1 group D member 2
ABCC1	ATP binding cassette subfamily C member 1
IL10RA	interleukin 10 receptor subunit alpha
PRPH2	peripherin 2
CYP3A7	cytochrome P450 family 3 subfamily A member 7
NBR1	NBR1, autophagy cargo receptor
HVCN1	hydrogen voltage gated channel 1
F13A1	coagulation factor XIII A chain
SAFB	scaffold attachment factor B
CNPY3	canopy FGF signaling regulator 3
TOR1A	torsin family 1 member A

USF1	upstream transcription factor 1
ARL2	ADP ribosylation factor like GTPase 2
MEPE	matrix extracellular phosphoglycoprotein
SLC30A5	solute carrier family 30 member 5
YBX1	Y-box binding protein 1
PPP1R14B	protein phosphatase 1 regulatory inhibitor subunit 14B
ELAVL4	ELAV like neuron-specific RNA binding protein 4
TRIM37	tripartite motif containing 37
KIDINS220	kinase D-interacting substrate 220kDa
TMEFF2	transmembrane protein with EGF like and two follistatin like domains 2
CDIPT	CDP-diacylglycerol--inositol 3-phosphatidyltransferase
SLC39A1	solute carrier family 39 member 1
TMEM132C	transmembrane protein 132C
SPRED2	sprouty related EVH1 domain containing 2
DEFB103A	defensin beta 103A
NTM	neurotrimin
DDX1	DEAD/H-box helicase 1
NR1I3	nuclear receptor subfamily 1 group I member 3
VIMP	VCP interacting membrane selenoprotein
OXA1L	OXA1L, mitochondrial inner membrane protein
FGF3	fibroblast growth factor 3
DSP	desmoplakin
LILRB2	leukocyte immunoglobulin like receptor B2
ITGA7	integrin subunit alpha 7
TAS2R10	taste 2 receptor member 10

S100A7	S100 calcium binding protein A7
PBRM1	polybromo 1
MRS2	MRS2, magnesium transporter
MTUS1	microtubule associated tumor suppressor 1
PRKDC	protein kinase, DNA-activated, catalytic polypeptide
ANXA13	annexin A13
ERAS	ES cell expressed Ras
IRS2	insulin receptor substrate 2
RP1-8B1.4	
NKAPL	NFKB activating protein like
FMO6P	flavin containing monooxygenase 6 pseudogene
SRP14-AS1	SRP14 antisense RNA1 (head to head)
ZEB1-AS1	ZEB1 antisense RNA 1
RP11-259N19.1	
SCARF2	scavenger receptor class F member 2
GPAT2	glycerol-3-phosphate acyltransferase 2, mitochondrial
RP13-314C10.5	
AADACP1	arylacetamide deacetylase pseudogene 1
ACAN	aggrecan
FFAR4	free fatty acid receptor 4
CA14	carbonic anhydrase 14
GFRA4	GDNF family receptor alpha 4
ALG1	ALG1, chitobiosyldiphosphodolichol beta-mannosyltransferase

ADAMTS15	ADAM metallopeptidase with thrombospondin type 1 motif 15
E2F1	E2F transcription factor 1
BACE2	beta-site APP-cleaving enzyme 2
ADAD1	adenosine deaminase domain containing 1
OSMR	oncostatin M receptor
ADCY2	adenylate cyclase 2
DOCK1	dedicator of cytokinesis 1
CXCR6	C-X-C motif chemokine receptor 6
PIAS1	protein inhibitor of activated STAT 1
ALCAM	activated leukocyte cell adhesion molecule
CDSN	corneodesmosin
CHFR	checkpoint with forkhead and ring finger domains, E3 ubiquitin protein ligase
TPR	translocated promoter region, nuclear basket protein
VCP	valosin containing protein
JPH1	junctophilin 1
FAM181A	family with sequence similarity 181 member A
CEP68	centrosomal protein 68
OGDH	oxoglutarate dehydrogenase
ADAM9	ADAM metallopeptidase domain 9
SMAD1	SMAD family member 1
RETNLB	resistin like beta
NFKB1	nuclear factor kappa B subunit 1
MAN1C1	mannosidase alpha class 1C member 1
EZR	ezrin
PRKD1	protein kinase D1

NAV1	neuron navigator 1
SERPINA6	serpin family A member 6
ACR	acrosin
TXN2	thioredoxin 2
FCGR2C	Fc fragment of IgG receptor IIc (gene/pseudogene)
PIWIL1	piwi like RNA-mediated gene silencing 1
STOM	stomatin
SHF	Src homology 2 domain containing F
PTGES2	prostaglandin E synthase 2
MUC6	mucin 6, oligomeric mucus/gel-forming
PRPF19	pre-mRNA processing factor 19
MC4R	melanocortin 4 receptor
PIM2	Pim-2 proto-oncogene, serine/threonine kinase
TNFSF13	tumor necrosis factor superfamily member 13
FOXA1	forkhead box A1
PGM1	phosphoglucomutase 1
PIAS3	protein inhibitor of activated STAT 3
KCNK15	potassium two pore domain channel subfamily K member 15
RPL13A	ribosomal protein L13a
SH2B1	SH2B adaptor protein 1
RRS1	ribosome biogenesis regulator homolog
DAD1	defender against cell death 1
TG	thyroglobulin
NR1I2	nuclear receptor subfamily 1 group I member 2
JPH2	junctophilin 2

SSFA2	sperm specific antigen 2
TTR	transthyretin
RPL32	ribosomal protein L32
FANK1	fibronectin type III and ankyrin repeat domains 1
P2RY14	purinergic receptor P2Y14
SLC9A3R1	SLC9A3 regulator 1
ESD	esterase D
UBQLN4	ubiquilin 4
CHAF1A	chromatin assembly factor 1 subunit A
PELP1	proline, glutamate and leucine rich protein 1
SLC6A15	solute carrier family 6 member 15
SLC2A1	solute carrier family 2 member 1
SOAT2	sterol O-acyltransferase 2
TES	testin LIM domain protein
AHNAK	AHNAK nucleoprotein
LIPE	lipase E, hormone sensitive type
AGTR2	angiotensin II receptor type 2
BMPR1A	bone morphogenetic protein receptor type 1A
GCKR	glucokinase regulator
NPL	N-acetylneuraminate pyruvate lyase
FOXP1	forkhead box P1
HPCAL1	hippocalcin like 1
HLA-DRB5	major histocompatibility complex, class II, DR beta 5
CRX	cone-rod homeobox
BRCA1	BRCA1, DNA repair associated

FIP1L1	factor interacting with PAPOLA and CPSF1
AWAT2	acyl-CoA wax alcohol acyltransferase 2
GGCT	gamma-glutamylcyclotransferase
FADS1	fatty acid desaturase 1
CHST8	carbohydrate sulfotransferase 8
BNIP3	BCL2 interacting protein 3
CARM1	coactivator associated arginine methyltransferase 1
RHOF	ras homolog family member F, filopodia associated
RBP7	retinol binding protein 7
PSORS1C1	psoriasis susceptibility 1 candidate 1
BMP4	bone morphogenetic protein 4
IL24	interleukin 24
SCP2	sterol carrier protein 2
NCR1	natural cytotoxicity triggering receptor 1
EFHC1	EF-hand domain containing 1
ITLN1	intelectin 1
PEF1	penta-EF-hand domain containing 1
ITGA5	integrin subunit alpha 5
KLK11	kallikrein related peptidase 11
OR52J3	olfactory receptor family 52 subfamily J member 3
NANOS3	nanos C2HC-type zinc finger 3
TMTC2	transmembrane and tetratricopeptide repeat containing 2
VAV1	vav guanine nucleotide exchange factor 1
S100P	S100 calcium binding protein P
EPB41	erythrocyte membrane protein band 4.1

PTCH1	patched 1
TNFRSF12A	TNF receptor superfamily member 12A
MRAP	melanocortin 2 receptor accessory protein
TUSC3	tumor suppressor candidate 3
PGR	progesterone receptor
GRM1	glutamate metabotropic receptor 1
DGKG	diacylglycerol kinase gamma
PTK7	protein tyrosine kinase 7 (inactive)
DNAH11	dynein axonemal heavy chain 11
KLC1	kinesin light chain 1
TNFRSF10D	TNF receptor superfamily member 10d
SERPINA7	serpin family A member 7
RPSA	ribosomal protein SA
CSF2RB	colony stimulating factor 2 receptor beta common subunit
ADGRG3	adhesion G protein-coupled receptor G3
FCGR3B	Fc fragment of IgG receptor IIIb
PPP3CB	protein phosphatase 3 catalytic subunit beta
PPIB	peptidylprolyl isomerase B
NOX3	NADPH oxidase 3
NOTCH3	notch 3
SLC25A18	solute carrier family 25 member 18
LRBA	LPS responsive beige-like anchor protein
C4BPA	complement component 4 binding protein alpha
BACE1	beta-secretase 1
TMEM18	transmembrane protein 18

MBP	myelin basic protein
SOX5	SRY-box 5
CASP4	caspase 4
AQP9	aquaporin 9
F11	coagulation factor XI
CDCA7L	cell division cycle associated 7 like
CCHCR1	coiled-coil alpha-helical rod protein 1
FUT2	fucosyltransferase 2
CBFB	core-binding factor beta subunit
CBLB	Cbl proto-oncogene B
DGAT2L6	diacylglycerol O-acyltransferase 2 like 6
IREB2	iron responsive element binding protein 2
FMR1	fragile X mental retardation 1
OPN1LW	opsin 1 (cone pigments), long-wave-sensitive
PSPH	phosphoserine phosphatase
STIM2	stromal interaction molecule 2
PRDX3	peroxiredoxin 3
TCERG1L	transcription elongation regulator 1 like
FOXB1	forkhead box B1
UBE2K	ubiquitin conjugating enzyme E2 K
CCAR1	cell division cycle and apoptosis regulator 1
ANKEF1	ankyrin repeat and EF-hand domain containing 1
GAS6	growth arrest specific 6
EPHA1	EPH receptor A1
ARIH2	ariadne RBR E3 ubiquitin protein ligase 2

ARSB	arylsulfatase B
DUSP4	dual specificity phosphatase 4
DNASE1	deoxyribonuclease 1
DFFB	DNA fragmentation factor subunit beta
HPD	4-hydroxyphenylpyruvate dioxygenase
CDK4	cyclin dependent kinase 4
MAPK10	mitogen-activated protein kinase 10
PON2	paraoxonase 2
NCAM1	neural cell adhesion molecule 1
PLA2G15	phospholipase A2 group XV
PPL	periplakin
TGM5	transglutaminase 5
NCR3	natural cytotoxicity triggering receptor 3
HDAC6	histone deacetylase 6
C9orf3	chromosome 9 open reading frame 3
OXR1	oxidation resistance 1
CACNA1E	calcium voltage-gated channel subunit alpha1 E
CDC42	cell division cycle 42
LOXL2	lysyl oxidase like 2
DNAH5	dynein axonemal heavy chain 5
ATXN7	ataxin 7
HAMP	hepcidin antimicrobial peptide
EBF3	early B-cell factor 3
TUFM	Tu translation elongation factor, mitochondrial
RARG	retinoic acid receptor gamma

OGG1	8-oxoguanine DNA glycosylase
ODC1	ornithine decarboxylase 1
PRDX1	peroxiredoxin 1
COL6A5	collagen type VI alpha 5 chain
UGT1A6	UDP glucuronosyltransferase family 1 member A6
P2RX5	purinergic receptor P2X 5
P2RX4	purinergic receptor P2X 4
PRMT3	protein arginine methyltransferase 3
PIP5K1B	phosphatidylinositol-4-phosphate 5-kinase type 1 beta
YWHAB	tyrosine 3-monooxygenase/tryptophan 5-monooxygenase activation protein beta
HTN1	histatin 1
KALRN	kalirin, RhoGEF kinase
TIRAP	TIR domain containing adaptor protein
SNX9	sorting nexin 9
TRIM63	tripartite motif containing 63
MFGE8	milk fat globule-EGF factor 8 protein
PRRC2A	proline rich coiled-coil 2A
XCR1	X-C motif chemokine receptor 1
NVL	nuclear VCP-like
TPSAB1	tryptase alpha/beta 1
PRLR	prolactin receptor
PLA2G6	phospholipase A2 group VI
TTC6	tetratricopeptide repeat domain 6
RXRA	retinoid X receptor alpha

RP11-638I2.10	
CTD-2562J17.6	
S100A11	S100 calcium binding protein A11
IGHEP2	immunoglobulin heavy constant epsilon P2 (pseudogene)
RP11-480I12.5	
CTC-479C5.12	
SCHLAP1	SWI/SNF complex antagonist associated with prostate cancer 1 (non-protein coding)
ANOS1	anosmin 1
RGS1	regulator of G-protein signaling 1
MIR429	microRNA 429
TTLL7	tubulin tyrosine ligase like 7
IL27	interleukin 27
TCN2	transcobalamin 2
PIGR	polymeric immunoglobulin receptor
ITGAL	integrin subunit alpha L
RPE	ribulose-5-phosphate-3-epimerase
MYH11	myosin heavy chain 11
PRPS1	phosphoribosyl pyrophosphate synthetase 1
AFF4	AF4/FMR2 family member 4
AP001347.6	

LRP2BP	LRP2 binding protein
BTG4	BTG anti-proliferation factor 4
TLDC2	TBC/LysM-associated domain containing 2
INHBB	inhibin beta B subunit
MAGI2-AS3	MAGI2 antisense RNA 3
DLG2	discs large MAGUK scaffold protein 2
RP11-12A20.7	
RP4-669K10.8	
ARMCX1	armadillo repeat containing, X-linked 1
POLR1E	RNA polymerase I subunit E
KIAA1456	KIAA1456
AKAP7	A-kinase anchoring protein 7
FAM86B3P	family with sequence similarity 86 member B3, pseudogene
C1orf147	chromosome 1 open reading frame 147
APBB1	amyloid beta precursor protein binding family B member 1
RP11-54A4.2	
CASP1P2	caspase 1 pseudogene 2
ARHGEF26-AS1	ARHGEF26 antisense RNA 1
CAPN9	calpain 9
NINJ2	ninjurin 2
ZDHHC14	zinc finger DHHC-type containing 14
C19orf38	chromosome 19 open reading frame 38

ZNF331	zinc finger protein 331
ZFPM2	zinc finger protein, FOG family member 2
CTD-2619J13.16	
TMEM170B	transmembrane protein 170B
GPR12	G protein-coupled receptor 12
PANK1	pantothenate kinase 1
UGT8	UDP glycosyltransferase 8
KCNJ16	potassium voltage-gated channel subfamily J member 16
CHIC1	cysteine rich hydrophobic domain 1
MT-TL1	mitochondrially encoded tRNA leucine 1 (UUA/G)
CCNB2	cyclin B2
CST9	cystatin 9
RP11-10H3.1	
SMO	smoothened, frizzled class receptor
MYEF2	myelin expression factor 2
CTD-2240E14.4	
CEP19	centrosomal protein 19
LHFP	lipoma HMGIC fusion partner
PBX3	PBX homeobox 3
SRM	spermidine synthase
ACKR1	atypical chemokine receptor 1 (Duffy blood group)
RGS14	regulator of G-protein signaling 14
TMEM178A	transmembrane protein 178A

SRL	sarcalumenin
STH	saitohin
PROM1	prominin 1
SLC24A2	solute carrier family 24 member 2
DCK	deoxycytidine kinase
AK1	adenylate kinase 1
CTTNBP2	cortactin binding protein 2
XIST	X inactive specific transcript (non-protein coding)
CTD-2368P22.1	
RP11-135F9.3	
ACBD7	acyl-CoA binding domain containing 7
AKR1B10	aldo-keto reductase family 1 member B10
PSMB8-AS1	PSMB8 antisense RNA 1 (head to head)
KCNAB2	potassium voltage-gated channel subfamily A regulatory beta subunit 2
CORO2B	coronin 2B
BANP	BTG3 associated nuclear protein
LCT	lactase
ADH6	alcohol dehydrogenase 6 (class V)
HLA-H	major histocompatibility complex, class I, H (pseudogene)
DBF4B	DBF4 zinc finger B
KLF15	Kruppel like factor 15
LDLRAP1	low density lipoprotein receptor adaptor protein 1
LAMP1	lysosomal associated membrane protein 1

PCAT6	prostate cancer associated transcript 6 (non-protein coding)
DUSP19	dual specificity phosphatase 19
CH17-340M24.3	uncharacterized protein BC009467
SH3RF3	SH3 domain containing ring finger 3
C2orf73	chromosome 2 open reading frame 73
FLNA	filamin A
AC006014.8	
MEF2A	myocyte enhancer factor 2A
PPP5C	protein phosphatase 5 catalytic subunit
AC138035.2	
MIR31HG	MIR31 host gene
SEC14L3	SEC14 like lipid binding 3
TM4SF1	transmembrane 4 L six family member 1
SMPD2	sphingomyelin phosphodiesterase 2
ROBO2	roundabout guidance receptor 2
KIAA0391	KIAA0391
NEU1	neuraminidase 1
ELK3	ELK3, ETS transcription factor
KIAA0825	KIAA0825
FLCN	folliculin
GSDMD	gasdermin D
CDC42EP3	CDC42 effector protein 3
FOXO4	forkhead box O4
LCK	LCK proto-oncogene, Src family tyrosine kinase

SOX9	SRY-box 9
RBPJ	recombination signal binding protein for immunoglobulin kappa J region
UNC93B1	unc-93 homolog B1 (C. elegans)
ITPR1	inositol 1,4,5-trisphosphate receptor type 1
FMO3	flavin containing monooxygenase 3
TWSG1	twisted gastrulation BMP signaling modulator 1
TXNDC16	thioredoxin domain containing 16
THBS1	thrombospondin 1
CHST2	carbohydrate sulfotransferase 2
BMI1	BMI1 proto-oncogene, polycomb ring finger
GSDMC	gasdermin C
SLC7A7	solute carrier family 7 member 7
GYPB	glycophorin B (MNS blood group)
ITGB5	integrin subunit beta 5
UTY	ubiquitously transcribed tetratricopeptide repeat containing, Y-linked
P2RX3	purinergic receptor P2X 3
SPRY2	sprouty RTK signaling antagonist 2
TNKS	tankyrase
CCDC69	coiled-coil domain containing 69
STAP2	signal transducing adaptor family member 2
RBFOX1	RNA binding protein, fox-1 homolog 1
AMY2A	amylase, alpha 2A (pancreatic)
CDC20	cell division cycle 20
PER1	period circadian clock 1
RIPK1	receptor interacting serine/threonine kinase 1

MAP3K7	mitogen-activated protein kinase kinase kinase 7
TRIM24	tripartite motif containing 24
COMT	catechol-O-methyltransferase
LIPA	lipase A, lysosomal acid type
WNT5A	Wnt family member 5A
S100B	S100 calcium binding protein B
VDAC1	voltage dependent anion channel 1
TAB2	TGF-beta activated kinase 1/MAP3K7 binding protein 2
PSMG1	proteasome assembly chaperone 1
SLC7A1	solute carrier family 7 member 1
ENAH	enabled homolog (Drosophila)
ATXN1	ataxin 1
XBP1	X-box binding protein 1
C6orf15	chromosome 6 open reading frame 15
MDH2	malate dehydrogenase 2
TNFRSF13C	TNF receptor superfamily member 13C
ZAP70	zeta chain of T cell receptor associated protein kinase 70
MVD	mevalonate diphosphate decarboxylase
GPR4	G protein-coupled receptor 4
CCL16	C-C motif chemokine ligand 16
IL26	interleukin 26
WAC	WW domain containing adaptor with coiled-coil
NR6A1	nuclear receptor subfamily 6 group A member 1
NES	nestin
IFNA13	interferon alpha 13

CACNA1B	calcium voltage-gated channel subunit alpha1 B
SLC24A1	solute carrier family 24 member 1
P2RY1	purinergic receptor P2Y1

SUPPLEMENTAL TABLE 2. (IL5 and GR Pathway Genes): A) IL5 pathway genes

**Symbol**	**Gene Name**
AFP	alpha fetoprotein
AKT1	AKT serine/threonine kinase 1
BAX	BCL2 associated X, apoptosis regulator
BGLAP	bone gamma-carboxyglutamate protein
CDK5	cyclin dependent kinase 5
CDK5R1	cyclin dependent kinase 5 regulatory subunit 1
CDKN1A	cyclin dependent kinase inhibitor 1A
CGA	glycoprotein hormones, alpha polypeptide
CREB1	cAMP responsive element binding protein 1
CREBBP	CREB binding protein
CSF2	colony stimulating factor 2
CSN2	casein beta
CXCL8	C-X-C motif chemokine ligand 8
EGR1	early growth response 1
EP300	E1A binding protein p300
FGG	fibrinogen gamma chain
FKBP4	FK506 binding protein 4
FKBP5	FK506 binding protein 5
FOS	Fos proto-oncogene, AP-1 transcription factor subunit
GATA3	GATA binding protein 3
GSK3B	glycogen synthase kinase 3 beta
HDAC1	histone deacetylase 1

HDAC2	histone deacetylase 2
HSP90AA1	heat shock protein 90 alpha family class A member 1
ICAM1	intercellular adhesion molecule 1
IFNG	interferon gamma
IL13	interleukin 13
IL2	interleukin 2
IL4	interleukin 4
IL5	interleukin 5
IL6	interleukin 6
IRF1	interferon regulatory factor 1
JUN	Jun proto-oncogene, AP-1 transcription factor subunit
KMT5B	lysine methyltransferase 5B
KRT14	keratin 14
KRT17	keratin 17
KRT5	keratin 5
MAPK1	mitogen-activated protein kinase 1
MAPK10	mitogen-activated protein kinase 10
MAPK11	mitogen-activated protein kinase 11
MAPK14	mitogen-activated protein kinase 14
MAPK3	mitogen-activated protein kinase 3
MAPK8	mitogen-activated protein kinase 8
MAPK9	mitogen-activated protein kinase 9
MDM2	MDM2 proto-oncogene
MMP1	matrix metallopeptidase 1
NCOA1	nuclear receptor coactivator 1

NCOA2	nuclear receptor coactivator 2
NFATC1	nuclear factor of activated T-cells 1
NFKB1	nuclear factor kappa B subunit 1
NR1I3	nuclear receptor subfamily 1 group I member 3
NR3C1	nuclear receptor subfamily 3 group C member 1
NR4A1	nuclear receptor subfamily 4 group A member 1
PBX1	PBX homeobox 1
PCK2	phosphoenolpyruvate carboxykinase 2, mitochondrial
POMC	proopiomelanocortin
POU1F1	POU class 1 homeobox 1
POU2F1	POU class 2 homeobox 1
PPP5C	protein phosphatase 5 catalytic subunit
PRKACA	protein kinase cAMP-activated catalytic subunit alpha
PRKACB	protein kinase cAMP-activated catalytic subunit beta
PRKACG	protein kinase cAMP-activated catalytic subunit gamma
PRL	prolactin
RELA	RELA proto-oncogene, NF-kB subunit
SELE	selectin E
SFN	stratifin
SGK1	serum/glucocorticoid regulated kinase 1
SMARCA4	SWI/SNF related, matrix associated, actin dependent regulator of chromatin, subfamily a, member 4
SMARCC1	SWI/SNF related, matrix associated, actin dependent regulator of chromatin subfamily c member 1

SMARCC2	SWI/SNF related, matrix associated, actin dependent regulator of chromatin subfamily c member 2
SMARCD1	SWI/SNF related, matrix associated, actin dependent regulator of chromatin, subfamily d, member 1
SPI1	Spi-1 proto-oncogene
STAT1	signal transducer and activator of transcription 1
STAT5A	signal transducer and activator of transcription 5A
STAT5B	signal transducer and activator of transcription 5B
SUMO2	small ubiquitin-like modifier 2
TBP	TATA-box binding protein
TBX21	T-box 21
TP53	tumor protein p53
TSG101	tumor susceptibility 101
VIPR1	vasoactive intestinal peptide receptor 1
YWHAH	tyrosine 3-monooxygenase/tryptophan 5-monooxygenase activation protein eta

SUPPLEMENTAL TABLE 2. (IL5 and GR Pathway Genes): B) GR pathway genes

**Symbol**	**Gene Name**
CSF2RB	colony stimulating factor 2 receptor beta common subunit
SPRED1	sprouty related EVH1 domain containing 1
ELK1	ELK1, ETS transcription factor
AKT1	AKT serine/threonine kinase 1
FOXO3	forkhead box O3

FOS	Fos proto-oncogene, AP-1 transcription factor subunit
GRB2	growth factor receptor bound protein 2
GSK3A	glycogen synthase kinase 3 alpha
GSK3B	glycogen synthase kinase 3 beta
IL2	interleukin 2
IL5RA	interleukin 5 receptor subunit alpha
JAK1	Janus kinase 1
JAK2	Janus kinase 2
JUN	Jun proto-oncogene, AP-1 transcription factor subunit
KRAS	KRAS proto-oncogene, GTPase
LYN	LYN proto-oncogene, Src family tyrosine kinase
MAPT	microtubule associated protein tau
MYC	MYC proto-oncogene, bHLH transcription factor
PIK3CG	phosphatidylinositol-4,5-bisphosphate 3-kinase catalytic subunit gamma
PIK3R1	phosphoinositide-3-kinase regulatory subunit 1
PIK3R2	phosphoinositide-3-kinase regulatory subunit 2
MAPK1	mitogen-activated protein kinase 1
MAPK3	mitogen-activated protein kinase 3
MAP2K1	mitogen-activated protein kinase kinase 1
MAP2K2	mitogen-activated protein kinase kinase 2
PTPN11	protein tyrosine phosphatase, non-receptor type 11
RAF1	Raf-1 proto-oncogene, serine/threonine kinase
BCL2	BCL2, apoptosis regulator
RPS6	ribosomal protein S6
RPS6KA1	ribosomal protein S6 kinase A1

RPS6KB1	ribosomal protein S6 kinase B1
RPS6KB2	ribosomal protein S6 kinase B2
SHC1	SHC adaptor protein 1
SOS1	SOS Ras/Rac guanine nucleotide exchange factor 1
STAT1	signal transducer and activator of transcription 1
STAT3	signal transducer and activator of transcription 3
STAT5A	signal transducer and activator of transcription 5A
STAT5B	signal transducer and activator of transcription 5B
SYK	spleen associated tyrosine kinase
BTK	Bruton tyrosine kinase

SUPPLEMENTAL TABLE 3. Candidate Variant List

	Variant	Location
RAD50, TH2LCRR (IL5)	rs2040704	5:131973177
RAD50, TH2LCRR (IL5)	rs6871536	5:131969874
IL5 (IL5)	rs12652920	5:131885240
none (IL5)	rs2079103	5:131864506
none (IL5)	rs4143832	5:131862977
C5orf56 (IL5)	rs11242111	5:131756058
C5orf56, LOC102723741 (IL5)	rs4451042	5:131755477
none (IL2)	rs17389644	4:123497697
none (IL2)	rs72669153	4:123429029
IL2 (IL2)	rs2069763	4:123377482
IL2 (IL2)	rs2069772	4:123373133
none (IL2)	rs17454584	4:123353432
KIAA1109 (IL2)	rs4505848	4:123132492
LOC100130701 (GSK3B)	rs4676776	3:120106539
LOC100130701 (GSK3B)	rs4676768	3:120069736
NT5C3B (STAT5A)	rs4796712	17:39987130
FKBP10 (STAT5A)	rs8072866	17:39977862
FOS (FOS)	rs138334429	14:75747512
FOS (FOS)	rs4645855	14:75746869
FOS (FOS)	rs1046117	14:75746690
none (AKT1)	rs10146871	14:105110417
none (AKT1)	rs4983514	14:105108578
none (AKT1)	rs11621214	14:104808984
none (AKT1)	rs8016091	14:104807712
IL13 (IL13)	rs20541	5:131995964
IL13 (IL13)	rs1295686	5:131995843
RAD50 (IL4/IL13)	rs2897443	5:131929594
RAD50 (IL4)	rs60490216	5:131921885
C5orf56 (IL4)	rs2522056	5:131801726
C5orf56 (IL4)	rs2706383	5:131792402

Category
IL5/GR and either EGPA or Asthma; EGPA/Asthma and either IL5 or GR
IL5/GR and either EGPA or Asthma; EGPA/Asthma and either IL5 or GR
IL5/GR and either EGPA or Asthma; EGPA/Asthma and either IL5 or GR
IL5/GR and either EGPA or Asthma; EGPA/Asthma and either IL5 or GR
IL5/GR and either EGPA or Asthma; EGPA/Asthma and either IL5 or GR
IL5/GR and either EGPA or Asthma; EGPA/Asthma and either IL5 or GR
IL5/GR and either EGPA or Asthma; EGPA/Asthma and either IL5 or GR
IL5/GR and either EGPA or Asthma; EGPA/Asthma and either IL5 or GR
IL5/GR and either EGPA or Asthma; EGPA/Asthma and either IL5 or GR
IL5/GR and either EGPA or Asthma; EGPA/Asthma and either IL5 or GR
IL5/GR and either EGPA or Asthma; EGPA/Asthma and either IL5 or GR
IL5/GR and either EGPA or Asthma; EGPA/Asthma and either IL5 or GR
IL5/GR and either EGPA or Asthma; EGPA/Asthma and either IL5 or GR
IL5/GR and either EGPA or Asthma
IL5/GR and either EGPA or Asthma
IL5/GR and either EGPA or Asthma
IL5/GR and either EGPA or Asthma
IL5/GR and either EGPA or Asthma
IL5/GR and either EGPA or Asthma
IL5/GR and either EGPA or Asthma
IL5/GR and either EGPA or Asthma
IL5/GR and either EGPA or Asthma
IL5/GR and either EGPA or Asthma
IL5/GR and either EGPA or Asthma
EGPA/Asthma and either IL5 or GR
EGPA/Asthma and either IL5 or GR
EGPA/Asthma and either IL5 or GR
EGPA/Asthma and either IL5 or GR
EGPA/Asthma and either IL5 or GR
EGPA/Asthma and either IL5 or GR

SUPPLEMENTAL TABLE 4. GWAS Results for selected variants by endpoint

					Accrued Duration of Remission			Frequency of EGPA Relapse			Average OGC Daily Dose During Last 4 Weeks		
Chromosome: GRCh37 Position	RS ID	Allele 1	Allele 2	Allele 1 Frequency	P value[1]	Beta	Standard Error	P value	Beta	Standard Error	P value	Beta	Standard Error
10:10471329	rs2488170	C	A	0.60	2.39E-05	-0.8222	0.1798	9.40E-04	0.9079	0.2813	6.04E-01	0.1077	0.2066
10:10471416	rs2248309	A	G	0.60	2.19E-05	-0.8227	0.1789	1.12E-03	0.8944	0.2814	6.32E-01	0.0992	0.2060
10:10481352	rs2244527	A	C	0.59	6.00E-06	-0.8380	0.1690	1.59E-03	0.7826	0.2523	7.62E-01	0.0605	0.1990
10:10486211	rs2243951	T	A	0.57	3.15E-06	-0.7938	0.1546	1.40E-03	0.7643	0.2445	5.89E-01	0.0999	0.1837
10:112355675	rs533163688	D	R	0.82	4.87E-05	-1.0751	0.2461	2.49E-01	0.5009	0.4493	5.26E-01	0.1782	0.2791
10:129160098	rs2296638	G	A	0.68	1.16E-05	-0.8775	0.1837	7.91E-02	0.4785	0.2725	6.10E-01	0.1095	0.2137
10:14537686	rs6602734	T	A	0.65	2.41E-03	0.5845	0.1852	3.22E-01	-0.2402	0.2510	8.44E-05	-0.7298	0.1723
10:14538274	rs943184	A	G	0.65	2.40E-03	0.5819	0.1843	3.34E-01	-0.2335	0.2499	7.61E-05	-0.7302	0.1712
10:14543038	rs7088212	T	C	0.62	5.60E-03	0.5544	0.1938	2.99E-01	-0.2591	0.2593	7.14E-05	-0.7598	0.1774
10:14543874	rs12772502	T	A	0.62	5.32E-03	0.5571	0.1935	2.98E-01	-0.2601	0.2594	6.93E-05	-0.7604	0.1771
10:14548168	rs10796200	T	A	0.61	5.27E-03	0.5572	0.1933	2.58E-01	-0.2872	0.2649	8.72E-05	-0.7505	0.1777
10:14548864	rs11259144	C	T	0.61	4.92E-03	0.5644	0.1941	2.60E-01	-0.2856	0.2634	5.78E-05	-0.7710	0.1774
10:14553356	rs7076947	A	T	0.61	4.17E-03	0.5704	0.1923	2.41E-01	-0.2981	0.2645	5.71E-05	-0.7664	0.1762
10:14556836	rs76919809	T	A	0.61	4.17E-03	0.5692	0.1919	2.31E-01	-0.3048	0.2648	5.31E-05	-0.7674	0.1756
10:1647948	rs11250661	C	T	0.79	8.56E-02	0.4085	0.2350	8.18E-01	0.0669	0.2910	2.62E-05	-0.9281	0.2029
10:1648568	rs74108950	C	G	0.83	8.53E-02	0.4724	0.2715	9.44E-01	-0.0233	0.3319	9.62E-05	-1.0051	0.2395
10:1648583	rs74108952	G	A	0.78	2.97E-01	0.2340	0.2238	9.39E-01	0.0214	0.2797	5.02E-05	-0.8429	0.1921
10:17339770	rs1822722	T	C	0.88	9.20E-05	-1.8386	0.4396	1.61E-01	1.0284	0.7393	1.75E-01	0.6691	0.4869
10:34127027	rs11596686	A	T	0.77	9.89E-06	-1.0768	0.2234	5.49E-02	0.6357	0.3450	4.12E-01	0.2143	0.2596
10:70070122	rs34887418	I	R	0.69	5.52E-02	-0.4377	0.2250	8.85E-05	1.1744	0.3152	2.42E-01	0.2667	0.2258
10:70130205	rs556378360	C	T	0.74	3.08E-02	-0.7583	0.3445	6.46E-05	1.8288	0.4736	1.11E-01	0.5594	0.3452
10:71065023	rs4364944	T	C	0.87	3.17E-01	0.3314	0.3306	6.22E-05	-1.3671	0.3195	5.42E-01	-0.2006	0.3268
10:87203302	rs61421482	T	A	0.85	8.52E-01	-0.0553	0.2963	2.74E-05	-1.3464	0.3099	4.86E-01	-0.2034	0.2902
10:87206297	rs2882714	T	C	0.86	9.70E-01	0.0115	0.3081	2.20E-05	-1.3615	0.3090	4.76E-01	-0.2163	0.3015
10:87206493	rs2351561	A	G	0.86	8.24E-01	-0.0706	0.3174	5.70E-05	-1.3596	0.3228	4.17E-01	-0.2538	0.3104
10:87209115	rs7896754	T	A	0.85	8.47E-01	-0.0566	0.2933	2.03E-01	-0.2802	0.2220	5.29E-01	-0.1822	0.2875
10:87213944	rs72827571	C	T	0.86	9.84E-01	0.0062	0.3060	2.13E-05	-1.3466	0.3044	4.98E-01	-0.2044	0.2997
10:94612458	rs78586043	T	G	0.91	2.72E-02	-0.8042	0.3570	8.01E-05	5.9941	3.9273	5.58E-02	0.6928	0.3548
10:94622388	rs79873247	A	C	0.90	2.52E-02	-0.7878	0.3448	7.60E-05	5.6244	3.4348	5.79E-02	0.6644	0.3433

10:94759305	rs12413378	A	G	0.91	3.77E-02	-0.7318	0.3461	3.06E-05	227.3440	124.8090	5.15E-02	0.6801	0.3419
10:94759873	rs835245	A	T	0.80	9.08E-02	-0.3932	0.2300	6.33E-05	1.6973	0.4865	7.41E-01	0.0769	0.2317
10:94764079	rs71942995	R	D	0.92	1.22E-02	-0.9099	0.3538	4.02E-05	245.1400	146.7470	3.59E-02	0.7600	0.3537
10:94767057	rs56087738	T	A	0.92	1.25E-02	-0.9075	0.3542	9.81E-05	95.6682	94.2747	3.62E-02	0.7594	0.3540
10:94768166	rs10509649	A	T	0.92	1.23E-02	-0.9101	0.3543	4.46E-05	239.2380	152.0010	3.63E-02	0.7594	0.3542
10:94770488	rs17108042	A	C	0.92	1.24E-02	-0.9092	0.3544	5.52E-05	243.2250	163.5500	3.62E-02	0.7598	0.3542
10:94770697	rs17108043	C	T	0.92	1.24E-02	-0.9092	0.3544	5.52E-05	243.2250	163.5500	3.62E-02	0.7598	0.3542
10:94772223	rs17108050	A	G	0.92	1.25E-02	-0.9091	0.3545	4.93E-05	250.9990	161.8840	3.63E-02	0.7598	0.3544
10:98960663	rs1147593	T	C	0.75	7.01E-05	0.8512	0.1997	5.70E-01	-0.1467	0.2553	4.63E-01	-0.1663	0.2248
10:98961141	rs1253415	C	T	0.75	7.04E-05	0.8507	0.1997	5.73E-01	-0.1457	0.2552	4.64E-01	-0.1657	0.2247
10:98962903	rs1253410	G	A	0.73	1.64E-04	0.8174	0.2039	6.71E-01	-0.1100	0.2556	4.95E-01	-0.1532	0.2231
10:98966882	rs877825	G	A	0.75	7.95E-05	0.8347	0.1975	6.26E-01	-0.1244	0.2510	4.91E-01	-0.1539	0.2220
10:98967359	rs902466	G	A	0.75	7.75E-05	0.8380	0.1980	6.30E-01	-0.1233	0.2516	4.90E-01	-0.1545	0.2226
11:101660830	rs141179114	R	I	0.90	8.07E-05	-1.1663	0.2763	6.39E-02	0.8920	0.5397	4.43E-01	0.2395	0.3101
11:106424291	rs1793068	C	G	0.77	9.91E-05	-0.8689	0.2089	9.88E-02	0.5384	0.3372	4.22E-01	0.1888	0.2335
11:106427636	rs1791600	C	T	0.77	7.48E-05	-0.8602	0.2027	9.25E-02	0.5349	0.3289	4.19E-01	0.1853	0.2277
11:11767404	rs113692989	G	T	0.94	1.55E-01	0.5999	0.4187	4.62E-05	-1.3901	0.2965	8.31E-01	-0.0901	0.4190
11:11767759	rs76459442	C	A	0.94	1.57E-01	0.5962	0.4186	4.90E-05	-1.3829	0.2958	7.75E-01	-0.1202	0.4186
11:11769698	rs74614980	C	T	0.94	1.55E-01	0.5991	0.4183	4.61E-05	-1.3898	0.2964	8.32E-01	-0.0890	0.4185
11:11770103	rs55639928	A	T	0.94	1.55E-01	0.5985	0.4181	4.63E-05	-1.3894	0.2964	8.33E-01	-0.0888	0.4183
11:11771343	rs58064499	A	T	0.94	1.55E-01	0.5980	0.4180	4.63E-05	-1.3895	0.2965	8.36E-01	-0.0872	0.4182
11:11772204	rs11826231	G	T	0.94	1.55E-01	0.5978	0.4178	4.64E-05	-1.3894	0.2965	8.36E-01	-0.0869	0.4181
11:11774937	rs7930827	G	C	0.94	1.57E-01	0.5952	0.4179	4.91E-05	-1.3819	0.2956	7.75E-01	-0.1200	0.4180
11:11776107	rs16910044	C	T	0.94	1.58E-01	0.5955	0.4185	4.91E-05	-1.3822	0.2956	7.79E-01	-0.1179	0.4186
11:11777749	rs7939727	G	A	0.94	1.29E-01	0.6428	0.4194	8.31E-05	-1.3636	0.3014	9.44E-01	-0.0295	0.4209
11:11780390	rs74769387	G	C	0.94	1.29E-01	0.6372	0.4159	6.79E-05	-1.3797	0.3019	9.51E-01	0.0259	0.4174
11:11780607	rs16924665	T	C	0.94	1.30E-01	0.6352	0.4159	6.73E-05	-1.3803	0.3019	9.51E-01	0.0259	0.4173
11:11781604	rs78105203	G	A	0.94	1.30E-01	0.6350	0.4160	6.72E-05	-1.3830	0.3026	9.10E-01	0.0474	0.4174
11:124060058	rs2724660	C	T	0.57	4.02E-01	-0.1564	0.1863	5.74E-01	-0.1232	0.2155	1.11E-05	-0.7486	0.1554
11:124064042	rs113288402	I	R	0.56	3.83E-01	-0.1653	0.1892	5.72E-01	-0.1251	0.2181	9.17E-06	-0.7669	0.1574
11:124065057	rs2508891	A	G	0.65	7.29E-01	-0.0666	0.1924	5.63E-01	-0.1293	0.2216	6.05E-05	-0.7114	0.1642
11:124066627	rs2846236	A	G	0.57	4.37E-01	-0.1466	0.1885	5.57E-01	-0.1294	0.2165	6.21E-06	-0.7746	0.1555
11:124070913	rs10667412	I	R	0.57	4.55E-01	-0.1414	0.1891	5.49E-01	-0.1321	0.2168	4.78E-06	-0.7846	0.1553
11:124070987	rs2724662	T	A	0.57	4.55E-01	-0.1413	0.1891	9.71E-01	-0.0181	0.4983	4.43E-01	-0.2432	0.3150

11:124071288	rs11607309	T	C	0.62	5.20E-01	0.1361	0.2117	3.33E-01	0.2528	0.2669	8.76E-05	0.7700	0.1823
11:124071802	rs4936909	A	G	0.62	5.25E-01	0.1343	0.2116	3.32E-01	0.2534	0.2668	8.66E-05	0.7702	0.1822
11:124072231	rs2724664	G	A	0.57	4.62E-01	-0.1391	0.1892	5.46E-01	-0.1330	0.2168	4.49E-06	-0.7868	0.1552
11:124073762	rs2846241	G	A	0.57	4.57E-01	-0.1409	0.1892	5.47E-01	-0.1327	0.2168	4.40E-06	-0.7874	0.1551
11:124075787	rs3018529	C	A	0.57	4.69E-01	-0.1370	0.1893	5.44E-01	-0.1339	0.2169	4.27E-06	-0.7885	0.1551
11:124077164	rs2846228	A	C	0.57	4.68E-01	-0.1374	0.1894	5.46E-01	-0.1330	0.2169	4.31E-06	-0.7886	0.1552
11:124077182	rs2724666	T	C	0.66	7.78E-01	-0.0547	0.1941	5.49E-01	-0.1345	0.2227	3.87E-05	-0.7337	0.1644
11:124079006	rs1939863	G	A	0.66	7.75E-01	-0.0553	0.1940	5.47E-01	-0.1350	0.2226	3.87E-05	-0.7332	0.1643
11:124079146	rs2846230	T	A	0.66	7.75E-01	-0.0553	0.1940	5.48E-01	-0.1350	0.2226	3.87E-05	-0.7332	0.1643
11:124079534	rs10893167	T	C	0.62	5.45E-01	0.1280	0.2114	3.28E-01	0.2552	0.2667	8.38E-05	0.7704	0.1819
11:124079608	rs56980257	R	I	0.62	5.45E-01	0.1280	0.2114	3.28E-01	0.2552	0.2667	8.38E-05	0.7704	0.1819
11:124080543	rs2508887	C	T	0.66	7.79E-01	-0.0544	0.1940	5.47E-01	-0.1353	0.2226	3.85E-05	-0.7334	0.1643
11:124081303	rs10893169	A	C	0.62	5.45E-01	0.1280	0.2114	3.28E-01	0.2553	0.2667	8.38E-05	0.7705	0.1819
11:124081628	rs4245049	G	A	0.62	5.45E-01	0.1280	0.2114	3.28E-01	0.2552	0.2667	8.37E-05	0.7705	0.1818
11:124083149	rs10790652	A	G	0.62	5.45E-01	0.1280	0.2114	3.28E-01	0.2552	0.2667	8.37E-05	0.7705	0.1818
11:124084653	rs2846232	T	C	0.66	7.79E-01	-0.0543	0.1940	5.47E-01	-0.1353	0.2226	3.84E-05	-0.7334	0.1643
11:124085462	rs948071	A	G	0.66	7.79E-01	-0.0543	0.1940	5.47E-01	-0.1353	0.2226	3.84E-05	-0.7334	0.1643
11:124087308	rs2846233	C	T	0.66	7.79E-01	-0.0543	0.1940	5.47E-01	-0.1352	0.2226	3.85E-05	-0.7333	0.1643
11:124092948	rs56319546	G	A	0.67	8.01E-01	-0.0490	0.1947	5.44E-01	-0.1376	0.2247	2.72E-05	-0.7480	0.1639
11:124093501	rs55893764	G	T	0.66	7.86E-01	-0.0526	0.1941	5.44E-01	-0.1361	0.2227	3.88E-05	-0.7334	0.1644
11:124093595	rs56220198	C	T	0.66	7.80E-01	-0.0542	0.1940	5.47E-01	-0.1353	0.2226	3.84E-05	-0.7334	0.1643
11:124093969	rs79704045	R	D	0.66	7.78E-01	-0.0548	0.1943	5.47E-01	-0.1353	0.2229	3.66E-05	-0.7365	0.1645
11:124096988	rs61907159	A	G	0.66	7.79E-01	-0.0543	0.1940	5.47E-01	-0.1353	0.2226	3.84E-05	-0.7334	0.1643
11:124097349	rs61908760	A	G	0.66	7.79E-01	-0.0543	0.1940	5.47E-01	-0.1353	0.2226	3.84E-05	-0.7334	0.1643
11:124097525	rs61908761	A	G	0.66	7.79E-01	-0.0543	0.1940	5.47E-01	-0.1353	0.2226	3.84E-05	-0.7334	0.1643
11:124102197	rs61313084	R	I	0.66	7.79E-01	-0.0543	0.1940	5.47E-01	-0.1354	0.2226	3.85E-05	-0.7334	0.1643
11:124102986	rs150770491	R	I	0.66	7.79E-01	-0.0543	0.1940	5.47E-01	-0.1354	0.2226	3.85E-05	-0.7335	0.1643
11:124103659	rs7482314	T	A	0.66	7.79E-01	-0.0545	0.1940	5.47E-01	-0.1353	0.2226	3.84E-05	-0.7336	0.1643
11:124105982	rs7114422	G	A	0.66	7.79E-01	-0.0544	0.1940	5.47E-01	-0.1354	0.2226	3.84E-05	-0.7336	0.1643
11:124108081	rs367723701	G	A	0.65	7.74E-01	-0.0561	0.1953	5.43E-01	-0.1376	0.2239	4.37E-05	-0.7340	0.1658
11:124108082	rs371165889	T	C	0.65	7.74E-01	-0.0561	0.1953	5.43E-01	-0.1376	0.2239	4.37E-05	-0.7340	0.1658
11:124109918	rs4482035	T	C	0.66	7.78E-01	-0.0545	0.1940	5.47E-01	-0.1352	0.2226	3.85E-05	-0.7334	0.1643
11:124110080	rs11219520	A	G	0.66	7.79E-01	-0.0543	0.1940	5.47E-01	-0.1353	0.2226	3.84E-05	-0.7334	0.1643
11:124111774	rs11219523	A	G	0.67	7.99E-01	-0.0495	0.1945	5.46E-01	-0.1366	0.2243	2.69E-05	-0.7475	0.1637

11:124114644	rs11219526	C	T	0.66	7.79E-01	-0.0544	0.1940	5.47E-01	-0.1353	0.2226	3.85E-05	-0.7335	0.1643
11:124115001	rs17771860	G	C	0.66	7.79E-01	-0.0544	0.1941	5.47E-01	-0.1353	0.2228	3.82E-05	-0.7341	0.1644
11:124116213	rs12804360	T	C	0.56	2.51E-01	0.2313	0.2010	7.03E-01	0.0868	0.2248	9.97E-05	0.7317	0.1748
11:124116657	rs11607806	G	T	0.51	3.03E-01	0.1888	0.1827	7.40E-01	0.0689	0.2039	9.91E-05	0.6641	0.1586
11:124116691	rs11603607	A	G	0.51	3.03E-01	0.1888	0.1827	7.40E-01	0.0689	0.2039	9.91E-05	0.6641	0.1586
11:124116707	rs11601644	T	G	0.51	3.03E-01	0.1888	0.1827	7.40E-01	0.0689	0.2039	9.91E-05	0.6641	0.1586
11:124118578	rs11219532	C	T	0.66	7.77E-01	-0.0550	0.1942	5.48E-01	-0.1352	0.2228	3.83E-05	-0.7345	0.1645
11:124120821	rs12099154	T	C	0.66	7.77E-01	-0.0549	0.1941	5.47E-01	-0.1353	0.2227	3.82E-05	-0.7342	0.1644
11:124123105	rs11219536	T	C	0.66	7.76E-01	-0.0552	0.1941	5.47E-01	-0.1352	0.2227	3.83E-05	-0.7341	0.1644
11:124124924	rs12224218	C	A	0.66	7.77E-01	-0.0549	0.1941	5.47E-01	-0.1353	0.2227	3.82E-05	-0.7342	0.1644
11:124126219	rs10502286	T	C	0.66	7.77E-01	-0.0549	0.1941	5.47E-01	-0.1353	0.2227	3.82E-05	-0.7342	0.1644
11:124132094	rs66595435	A	G	0.66	7.75E-01	-0.0553	0.1942	5.47E-01	-0.1353	0.2227	3.81E-05	-0.7344	0.1644
11:124133238	rs2512175	A	T	0.50	3.74E-01	0.1608	0.1804	8.00E-01	0.0516	0.1997	9.64E-05	0.6551	0.1562
11:124134552	rs2466636	C	T	0.50	6.20E-01	0.0901	0.1819	8.59E-01	0.0362	0.1997	5.18E-05	0.6787	0.1550
11:124142711	rs7120833	A	T	0.66	7.84E-01	-0.0533	0.1947	5.56E-01	-0.1327	0.2233	3.94E-05	-0.7351	0.1649
11:124144266	rs11219548	G	A	0.62	5.48E-01	0.1269	0.2116	3.28E-01	0.2556	0.2668	8.21E-05	0.7720	0.1819
11:124149102	rs34374349	R	D	0.66	7.89E-01	-0.0520	0.1950	5.62E-01	-0.1306	0.2236	4.07E-05	-0.7351	0.1653
11:124150614	rs11219554	C	G	0.65	7.90E-01	-0.0517	0.1950	5.64E-01	-0.1302	0.2236	4.09E-05	-0.7350	0.1653
11:124151297	rs11537156	A	G	0.65	7.91E-01	-0.0516	0.1950	5.64E-01	-0.1300	0.2236	4.11E-05	-0.7350	0.1653
11:124152329	rs61907298	C	A	0.65	7.92E-01	-0.0515	0.1951	5.65E-01	-0.1298	0.2237	4.12E-05	-0.7350	0.1654
11:124152515	rs12223329	C	T	0.67	6.69E-01	-0.0845	0.1976	6.21E-01	-0.1132	0.2273	5.00E-05	-0.7384	0.1683
11:124152889	rs11530584	C	T	0.65	7.92E-01	-0.0514	0.1951	5.65E-01	-0.1296	0.2237	4.13E-05	-0.7350	0.1654
11:124153111	rs11534469	G	C	0.65	7.92E-01	-0.0513	0.1951	5.65E-01	-0.1296	0.2237	4.13E-05	-0.7350	0.1654
11:124153183	rs201326530	R	D	0.65	9.76E-01	-0.0063	0.2093	7.52E-01	-0.0757	0.2365	1.94E-05	-0.8162	0.1751
11:124153273	rs11559532	A	G	0.65	7.92E-01	-0.0513	0.1951	5.66E-01	-0.1295	0.2237	4.14E-05	-0.7350	0.1654
11:124153615	rs12226556	A	G	0.65	7.92E-01	-0.0512	0.1951	5.66E-01	-0.1295	0.2237	4.14E-05	-0.7350	0.1654
11:124154583	rs61907299	C	G	0.65	7.94E-01	-0.0510	0.1952	5.67E-01	-0.1291	0.2238	4.17E-05	-0.7349	0.1655
11:124160014	rs11219561	C	T	0.65	8.01E-01	-0.0492	0.1955	5.76E-01	-0.1263	0.2241	4.38E-05	-0.7344	0.1659
11:124160443	rs7952230	A	G	0.65	8.01E-01	-0.0492	0.1955	5.76E-01	-0.1262	0.2241	4.38E-05	-0.7344	0.1659
11:124161917	rs11219562	A	G	0.62	5.54E-01	0.1251	0.2117	3.27E-01	0.2560	0.2669	8.00E-05	0.7734	0.1820
11:124162350	rs4606489	A	T	0.62	5.55E-01	0.1250	0.2117	3.27E-01	0.2560	0.2669	7.98E-05	0.7735	0.1820
11:124166827	rs373754923	A	G	0.65	8.89E-01	-0.0284	0.2046	5.53E-01	-0.1399	0.2343	3.31E-05	-0.7788	0.1727
11:124166908	rs112420563	A	G	0.63	7.72E-01	-0.0595	0.2057	5.73E-01	-0.1321	0.2328	3.22E-05	-0.7843	0.1737
11:124166923	rs73019676	A	G	0.64	7.79E-01	-0.0582	0.2074	6.36E-01	-0.1115	0.2341	2.81E-05	-0.7959	0.1747

11:124166935	rs73019677	A	G	0.63	7.84E-01	-0.0565	0.2059	5.85E-01	-0.1281	0.2330	3.50E-05	-0.7820	0.1741
11:124166950	rs73019679	A	G	0.63	7.69E-01	-0.0604	0.2064	5.84E-01	-0.1289	0.2337	4.20E-05	-0.7771	0.1751
11:124167366	rs61907300	A	G	0.65	8.23E-01	-0.0438	0.1961	6.00E-01	-0.1185	0.2246	5.10E-05	-0.7309	0.1668
11:124169990	rs59650091	T	C	0.65	8.25E-01	-0.0433	0.1961	6.03E-01	-0.1177	0.2246	5.19E-05	-0.7303	0.1668
11:124172609	rs59909754	R	I	0.63	6.87E-01	-0.0792	0.1967	7.06E-01	-0.0861	0.2254	9.63E-05	-0.7104	0.1693
11:124175370	rs56098955	C	A	0.65	8.30E-01	-0.0420	0.1961	6.09E-01	-0.1157	0.2247	5.43E-05	-0.7290	0.1670
11:124178666	rs11219580	A	T	0.65	8.33E-01	-0.0412	0.1962	6.12E-01	-0.1149	0.2247	5.53E-05	-0.7283	0.1671
11:124181011	rs519158	T	G	0.65	8.38E-01	-0.0401	0.1962	6.16E-01	-0.1133	0.2247	5.74E-05	-0.7269	0.1672
11:124181444	rs515403	A	G	0.65	8.38E-01	-0.0400	0.1962	6.17E-01	-0.1132	0.2247	5.76E-05	-0.7269	0.1672
11:124181449	rs515395	C	T	0.66	8.52E-01	-0.0366	0.1969	6.18E-01	-0.1138	0.2264	4.06E-05	-0.7423	0.1668
11:124181846	rs490740	T	C	0.65	8.39E-01	-0.0399	0.1962	6.18E-01	-0.1130	0.2247	5.77E-05	-0.7268	0.1672
11:124187240	rs500901	A	G	0.65	8.42E-01	-0.0390	0.1963	6.25E-01	-0.1107	0.2249	5.99E-05	-0.7260	0.1675
11:124193170	rs507104	T	C	0.65	8.46E-01	-0.0382	0.1964	6.30E-01	-0.1090	0.2249	6.16E-05	-0.7253	0.1676
11:124198828	rs4057919	C	T	0.67	8.41E-01	-0.0399	0.1991	5.93E-01	-0.1248	0.2313	1.82E-05	-0.7787	0.1664
11:124205935	rs142623810	R	I	0.66	8.28E-01	-0.0435	0.2007	6.96E-01	-0.0906	0.2298	5.64E-05	-0.7446	0.1711
11:124207477	rs200293461	R	D	0.65	8.58E-01	-0.0351	0.1967	6.49E-01	-0.1033	0.2252	6.67E-05	-0.7234	0.1681
11:124207479	rs61911101	G	C	0.65	8.58E-01	-0.0351	0.1967	6.49E-01	-0.1033	0.2252	6.67E-05	-0.7234	0.1681
11:124207510	rs11219597	G	A	0.71	3.84E-01	0.1988	0.2280	2.77E-01	0.2912	0.2701	4.16E-05	0.8640	0.1945
11:124209483	rs507118	C	T	0.65	8.56E-01	-0.0357	0.1968	6.53E-01	-0.1020	0.2253	6.85E-05	-0.7228	0.1682
11:124209777	rs509878	C	T	0.66	8.75E-01	-0.0311	0.1973	6.55E-01	-0.1020	0.2266	4.89E-05	-0.7369	0.1677
11:124213673	rs637057	G	A	0.65	8.64E-01	-0.0338	0.1968	6.57E-01	-0.1006	0.2252	7.02E-05	-0.7216	0.1682
11:124219110	rs564202	A	G	0.65	8.70E-01	-0.0322	0.1967	6.66E-01	-0.0978	0.2251	7.31E-05	-0.7200	0.1683
11:124224662	rs600686	A	C	0.65	8.75E-01	-0.0309	0.1967	6.74E-01	-0.0952	0.2250	7.63E-05	-0.7182	0.1684
11:124226577	rs504362	C	T	0.66	8.86E-01	-0.0283	0.1975	6.92E-01	-0.0902	0.2265	5.62E-05	-0.7325	0.1682
11:124262346	rs202110706	A	G	0.69	7.88E-01	-0.0611	0.2281	8.86E-01	-0.0360	0.2486	2.65E-05	-0.8774	0.1919
11:124696112	rs570483	G	A	0.66	7.79E-05	0.8041	0.1900	3.53E-01	-0.2310	0.2423	4.53E-03	-0.5905	0.1998
11:129490119	rs77436888	G	A	0.88	9.90E-05	1.8615	0.4474	3.56E-01	-0.6342	0.6644	1.37E-02	-1.2125	0.4768
11:130087978	rs858725	C	T	0.81	2.60E-05	0.8766	0.1927	9.01E-01	-0.0379	0.2891	7.78E-02	-0.3873	0.2156
11:130104498	rs17605148	A	T	0.83	7.78E-05	1.0443	0.2468	4.25E-01	-0.2869	0.3569	6.61E-02	-0.5066	0.2704
11:131843583	rs34692300	C	T	0.84	3.68E-01	0.2422	0.2685	9.89E-01	0.0047	0.3301	3.14E-05	-1.0315	0.2281
11:131848471	rs71485708	C	T	0.84	3.64E-01	0.2388	0.2626	9.99E-01	-0.0003	0.3249	4.32E-05	-0.9939	0.2243
11:132719696	rs4937735	T	C	0.77	2.92E-01	-0.2573	0.2434	8.69E-03	1.0046	0.3889	7.47E-05	0.8982	0.2103
11:18778478	rs10832977	A	C	0.80	4.20E-05	-1.1847	0.2686	6.81E-02	0.7615	0.4201	1.15E-02	0.7561	0.2896
11:24041531	rs10834263	C	T	0.68	8.87E-05	0.7718	0.1841	3.23E-01	-0.2448	0.2494	5.95E-03	-0.5541	0.1939

11:24043815	rs12295367	T	G	0.68	8.09E-05	0.7684	0.1821	3.16E-01	-0.2459	0.2467	6.36E-03	-0.5449	0.1923
11:24044674	rs10834264	G	A	0.69	9.07E-05	0.7540	0.1801	3.07E-01	-0.2478	0.2441	7.27E-03	-0.5298	0.1903
11:24045029	rs10834265	C	T	0.32	8.15E-05	-0.7663	0.1817	3.15E-01	0.2452	0.2459	6.28E-03	0.5444	0.1919
11:24046032	rs55851544	R	D	0.69	5.00E-05	0.8496	0.1948	3.00E-01	-0.2795	0.2713	8.84E-03	-0.5655	0.2086
11:24047010	rs4923149	C	A	0.68	5.84E-05	0.7810	0.1810	2.44E-01	-0.2852	0.2447	1.00E-02	-0.5157	0.1936
11:24048346	rs2403884	C	T	0.68	5.55E-05	0.7813	0.1804	2.43E-01	-0.2847	0.2441	1.03E-02	-0.5127	0.1933
11:24048870	rs10466429	T	C	0.33	5.48E-05	-0.7878	0.1818	2.41E-01	0.2880	0.2457	9.28E-03	0.5236	0.1944
11:24049673	rs5790382	I	R	0.33	5.64E-05	-0.7863	0.1818	2.41E-01	0.2878	0.2460	7.94E-03	0.5334	0.1939
11:24050159	rs11027667	G	A	0.71	4.82E-05	0.8115	0.1856	3.34E-01	-0.2374	0.2464	7.39E-03	-0.5509	0.1983
11:24050237	rs10767154	G	A	0.33	5.63E-05	-0.7862	0.1817	2.42E-01	0.2874	0.2458	7.95E-03	0.5332	0.1938
11:24051340	rs61655096	R	D	0.74	4.94E-05	0.8345	0.1912	3.72E-01	-0.2286	0.2568	1.42E-03	-0.6670	0.1988
11:24053257	rs61876465	G	A	0.72	4.89E-05	0.8016	0.1836	3.16E-01	-0.2429	0.2430	8.28E-03	-0.5372	0.1964
11:24056701	rs11027675	G	A	0.72	5.47E-05	0.7946	0.1833	3.22E-01	-0.2392	0.2421	8.72E-03	-0.5322	0.1959
11:24058345	rs11027676	G	C	0.36	1.97E-05	-0.7846	0.1695	2.41E-01	0.2730	0.2356	9.50E-03	0.4954	0.1846
11:24058731	rs35306297	C	G	0.68	6.00E-05	0.7743	0.1798	2.47E-01	-0.2817	0.2438	9.43E-03	-0.5161	0.1920
11:24060691	rs7949813	G	A	0.71	7.79E-05	0.7587	0.1793	3.44E-01	-0.2200	0.2324	2.48E-02	-0.4464	0.1936
11:24061085	rs4412756	T	G	0.71	4.06E-05	0.8210	0.1857	3.54E-01	-0.2290	0.2480	1.11E-02	-0.5258	0.2002
11:24061337	rs4372449	C	T	0.69	7.87E-05	0.7634	0.1805	3.34E-01	-0.2352	0.2453	6.85E-03	-0.5360	0.1910
11:24061481	rs4427563	G	A	0.72	4.69E-05	0.8104	0.1851	3.61E-01	-0.2250	0.2474	9.78E-03	-0.5310	0.1986
11:24064534	rs7115164	C	T	0.69	8.88E-05	0.7695	0.1835	3.73E-01	-0.2207	0.2504	7.01E-03	-0.5424	0.1939
11:24065625	rs72881122	G	T	0.71	3.01E-05	0.8480	0.1882	3.90E-01	-0.2176	0.2542	4.72E-03	-0.5916	0.2011
11:24067367	rs1915049	T	A	0.69	7.38E-05	0.7924	0.1866	4.54E-01	-0.1901	0.2576	8.44E-03	-0.5411	0.1983
11:24067498	rs1915050	G	C	0.34	3.84E-05	-0.8338	0.1879	4.88E-01	0.1811	0.2650	1.63E-02	0.5050	0.2040
11:24067879	rs10834269	A	G	0.71	6.62E-05	0.7968	0.1862	4.79E-01	-0.1722	0.2447	3.10E-02	-0.4476	0.2023
11:24070434	rs1996751	G	A	0.71	6.86E-05	0.8277	0.1939	5.45E-01	-0.1649	0.2773	9.86E-03	-0.5522	0.2068
11:24072469	rs11027684	A	G	0.71	8.06E-05	0.8332	0.1974	5.96E-01	-0.1384	0.2638	3.13E-02	-0.4719	0.2138
11:24072558	rs11027685	G	T	0.72	8.08E-05	0.8326	0.1973	5.99E-01	-0.1370	0.2636	3.16E-02	-0.4709	0.2137
11:24073794	rs1996652	A	G	0.66	9.62E-05	0.7810	0.1873	6.93E-01	-0.1010	0.2619	8.71E-02	-0.3575	0.2053
11:24074207	rs11027689	G	A	0.69	4.15E-05	0.8489	0.1923	7.79E-01	-0.0737	0.2666	4.51E-02	-0.4338	0.2118
11:24074862	rs11027691	C	T	0.66	9.91E-05	0.7835	0.1883	7.16E-01	-0.0937	0.2633	8.78E-02	-0.3584	0.2064
11:24075258	rs151303153	R	I	0.71	5.53E-05	0.8864	0.2047	7.83E-01	-0.0772	0.2836	3.97E-02	-0.4712	0.2239
11:24075628	rs146625252	R	D	0.69	4.28E-05	0.8521	0.1934	8.01E-01	-0.0666	0.2682	4.67E-02	-0.4331	0.2130
11:24076734	rs11027693	T	G	0.67	6.52E-05	0.8039	0.1877	6.72E-01	-0.1040	0.2496	1.35E-01	-0.3163	0.2084
11:24080703	rs58055352	D	R	0.36	7.35E-05	-0.8289	0.1951	7.47E-01	0.0862	0.2732	8.71E-02	0.3740	0.2149

11:24083290	rs11027698	T	C	0.69	4.92E-05	0.8693	0.1991	8.57E-01	-0.0490	0.2767	5.36E-02	-0.4320	0.2192
11:24083487	rs138997652	R	D	0.69	4.82E-05	0.8678	0.1985	8.74E-01	-0.0431	0.2764	5.29E-02	-0.4320	0.2186
11:24083553	rs11027699	A	G	0.68	4.53E-05	0.8733	0.1990	8.82E-01	-0.0408	0.2784	5.19E-02	-0.4352	0.2193
11:24086146	rs4145286	A	G	0.36	9.79E-05	-0.8430	0.2024	9.22E-01	0.0286	0.2999	5.27E-02	0.4357	0.2202
11:35233221	rs67804554	R	D	0.62	2.09E-01	-0.2928	0.2318	9.90E-01	-0.0035	0.2836	5.39E-05	0.8737	0.2001
11:43014018	rs5791545	I	R	0.63	6.60E-05	-0.7285	0.1702	7.65E-01	0.0760	0.2420	2.84E-02	0.4153	0.1847
11:43883464	rs73540482	T	C	0.86	7.31E-01	-0.0982	0.2860	3.18E-01	0.3846	0.4009	7.71E-05	1.0444	0.2451
11:43932336	rs558823932	R	I	0.88	6.06E-01	-0.1524	0.2957	5.12E-01	0.2545	0.4033	3.92E-05	1.1188	0.2509
11:43947348	rs4755757	C	G	0.89	3.85E-01	-0.2618	0.3012	5.20E-01	0.2523	0.4098	9.90E-05	1.0917	0.2607
11:45776525	rs2467285	C	T	0.32	1.54E-02	-0.5703	0.2297	8.98E-05	1.1463	0.2901	4.57E-01	0.1771	0.2368
11:45777813	rs1001595	G	A	0.32	1.59E-02	-0.5662	0.2293	8.33E-05	1.1490	0.2898	4.50E-01	0.1796	0.2361
11:48199897	rs2047815	C	T	0.75	8.63E-01	-0.0373	0.2160	9.46E-01	0.0196	0.2926	8.47E-05	0.7842	0.1853
11:48200265	rs10838817	A	G	0.75	8.63E-01	-0.0372	0.2160	9.47E-01	0.0194	0.2926	8.43E-05	0.7844	0.1852
11:48200532	rs537958379	I	R	0.72	8.78E-01	-0.0352	0.2299	8.47E-01	0.0586	0.3083	7.25E-05	0.8416	0.1967
11:48200586	rs2202249	T	C	0.75	8.65E-01	-0.0365	0.2158	9.47E-01	0.0191	0.2926	8.47E-05	0.7838	0.1851
11:48201283	rs10838819	T	A	0.88	9.79E-01	0.0073	0.2704	4.52E-02	0.7330	0.3797	9.97E-05	0.9732	0.2325
11:48203313	rs11039565	T	C	0.88	9.81E-01	0.0064	0.2664	4.42E-02	0.7239	0.3736	9.94E-05	0.9589	0.2291
11:48203373	rs10838820	C	A	0.88	9.81E-01	0.0064	0.2664	4.42E-02	0.7241	0.3736	9.95E-05	0.9590	0.2291
11:48203852	rs34332242	R	D	0.88	9.81E-01	0.0064	0.2664	4.42E-02	0.7239	0.3736	9.94E-05	0.9589	0.2291
11:48204922	rs12785331	A	G	0.88	9.81E-01	0.0063	0.2665	4.41E-02	0.7247	0.3737	1.00E-04	0.9590	0.2292
11:48205775	rs11039569	C	T	0.88	9.81E-01	0.0064	0.2664	4.42E-02	0.7241	0.3736	9.95E-05	0.9590	0.2291
11:48206806	rs12786800	A	G	0.88	9.81E-01	0.0064	0.2664	4.42E-02	0.7239	0.3736	9.94E-05	0.9589	0.2291
11:48207039	rs10838821	T	C	0.88	9.81E-01	0.0063	0.2665	4.41E-02	0.7247	0.3737	1.00E-04	0.9590	0.2292
11:48207511	rs11039572	A	G	0.88	9.81E-01	0.0062	0.2665	4.40E-02	0.7250	0.3737	9.98E-05	0.9591	0.2292
11:48208016	rs11604898	G	A	0.88	9.81E-01	0.0064	0.2664	4.42E-02	0.7239	0.3736	9.94E-05	0.9589	0.2291
11:48208428	rs10838822	T	C	0.88	9.81E-01	0.0065	0.2664	4.42E-02	0.7241	0.3737	9.96E-05	0.9590	0.2291
11:48209085	rs11039575	A	T	0.88	9.81E-01	0.0065	0.2664	4.42E-02	0.7241	0.3737	9.96E-05	0.9590	0.2291
11:48209187	rs11039576	T	C	0.88	9.81E-01	0.0064	0.2664	4.42E-02	0.7239	0.3736	9.94E-05	0.9589	0.2291
11:48209471	rs7479011	T	C	0.75	8.83E-01	-0.0316	0.2160	9.61E-01	0.0141	0.2931	8.42E-05	0.7846	0.1853
11:48209867	rs147007333	A	G	0.75	8.86E-01	-0.0311	0.2161	9.62E-01	0.0138	0.2932	8.44E-05	0.7847	0.1853
11:48210437	rs11530176	G	A	0.88	9.81E-01	0.0065	0.2664	4.42E-02	0.7241	0.3737	9.96E-05	0.9590	0.2291
11:48211600	rs12798054	T	C	0.88	9.81E-01	0.0065	0.2664	4.42E-02	0.7241	0.3737	9.96E-05	0.9590	0.2291
11:48211658	rs12796400	A	G	0.88	9.81E-01	0.0064	0.2664	4.42E-02	0.7239	0.3736	9.94E-05	0.9589	0.2291
11:48211823	rs12577527	C	T	0.88	9.81E-01	0.0064	0.2664	4.42E-02	0.7239	0.3736	9.94E-05	0.9589	0.2291

11:48212348	rs12803097	G	A	0.88	9.81E-01	0.0064	0.2664	4.42E-02	0.7241	0.3736	9.95E-05	0.9590	0.2291
11:48212687	rs12803854	G	A	0.88	9.81E-01	0.0064	0.2664	4.42E-02	0.7241	0.3736	9.95E-05	0.9590	0.2291
11:48212951	rs12577851	C	T	0.88	9.81E-01	0.0064	0.2664	4.42E-02	0.7239	0.3736	9.94E-05	0.9589	0.2291
11:48213002	rs12577881	G	C	0.88	9.81E-01	0.0065	0.2667	4.43E-02	0.7248	0.3741	9.96E-05	0.9600	0.2294
11:48213013	rs12575738	A	G	0.88	9.80E-01	0.0066	0.2665	4.42E-02	0.7241	0.3738	9.97E-05	0.9590	0.2292
11:48213377	rs12785433	A	C	0.88	9.81E-01	0.0064	0.2664	4.42E-02	0.7239	0.3736	9.94E-05	0.9589	0.2291
11:48213446	rs10769322	T	C	0.76	9.00E-01	-0.0271	0.2163	9.70E-01	0.0109	0.2937	8.55E-05	0.7849	0.1855
11:48213453	rs10769323	T	C	0.76	9.00E-01	-0.0271	0.2163	9.70E-01	0.0109	0.2937	8.55E-05	0.7849	0.1855
11:48213515	rs10769324	C	A	0.76	9.00E-01	-0.0271	0.2163	9.70E-01	0.0109	0.2937	8.55E-05	0.7849	0.1855
11:48213833	rs12573908	C	A	0.88	9.81E-01	0.0065	0.2664	4.42E-02	0.7239	0.3736	9.94E-05	0.9590	0.2291
11:48216749	rs2870084	T	C	0.88	9.80E-01	0.0066	0.2665	4.42E-02	0.7243	0.3737	9.97E-05	0.9591	0.2292
11:48216916	rs34711362	T	C	0.88	9.80E-01	0.0066	0.2665	4.42E-02	0.7243	0.3737	9.97E-05	0.9591	0.2292
11:48218668	rs10838825	A	G	0.88	9.81E-01	0.0062	0.2666	4.40E-02	0.7254	0.3739	9.91E-05	0.9597	0.2292
11:48219490	rs11602571	T	C	0.88	9.81E-01	0.0064	0.2666	4.40E-02	0.7253	0.3739	9.98E-05	0.9595	0.2293
11:48219812	rs10838826	T	G	0.75	8.97E-01	-0.0279	0.2165	9.72E-01	0.0103	0.2939	8.22E-05	0.7874	0.1856
11:48220989	rs201959245	C	T	0.75	8.78E-01	-0.0327	0.2140	9.75E-01	0.0089	0.2898	6.25E-05	0.7893	0.1826
11:48221095	rs145952381	I	R	0.69	7.08E-01	-0.0818	0.2182	9.66E-01	0.0130	0.3127	7.27E-05	0.7997	0.1869
11:48223283	rs1847261	T	G	0.75	8.79E-01	-0.0325	0.2137	9.75E-01	0.0091	0.2895	6.25E-05	0.7884	0.1824
11:48224060	rs4752810	T	A	0.75	8.79E-01	-0.0326	0.2136	9.75E-01	0.0088	0.2894	6.25E-05	0.7881	0.1823
11:48225680	rs7940877	G	A	0.75	8.77E-01	-0.0329	0.2134	9.75E-01	0.0089	0.2891	6.17E-05	0.7879	0.1821
11:48225839	rs7941311	A	G	0.75	8.77E-01	-0.0330	0.2134	9.75E-01	0.0090	0.2890	6.16E-05	0.7879	0.1821
11:48228788	rs4752916	G	A	0.75	8.77E-01	-0.0330	0.2132	9.76E-01	0.0087	0.2887	6.12E-05	0.7875	0.1819
11:48232547	rs1354295	T	A	0.75	9.00E-01	-0.0269	0.2138	9.72E-01	0.0102	0.2892	5.76E-05	0.7922	0.1822
11:48232548	rs1354294	A	C	0.75	8.77E-01	-0.0328	0.2130	9.81E-01	0.0066	0.2883	6.04E-05	0.7873	0.1817
11:48232853	rs1393794	C	T	0.75	8.77E-01	-0.0328	0.2129	9.80E-01	0.0072	0.2881	6.02E-05	0.7870	0.1816
11:48234813	rs11606200	C	G	0.88	9.80E-01	0.0067	0.2665	4.42E-02	0.7245	0.3738	9.98E-05	0.9591	0.2292
11:48236553	rs3923871	G	A	0.88	9.81E-01	0.0065	0.2664	4.42E-02	0.7239	0.3736	9.94E-05	0.9589	0.2291
11:48236725	rs11039590	C	A	0.88	9.81E-01	0.0064	0.2664	4.42E-02	0.7239	0.3736	9.94E-05	0.9589	0.2291
11:48237742	rs2102200	G	T	0.88	9.84E-01	0.0055	0.2671	4.58E-02	0.7211	0.3745	9.20E-05	0.9654	0.2294
11:48238019	rs1875697	C	T	0.88	9.81E-01	0.0065	0.2665	4.42E-02	0.7243	0.3737	9.96E-05	0.9590	0.2291
11:48238549	rs11606506	G	A	0.88	9.81E-01	0.0064	0.2664	4.42E-02	0.7239	0.3736	9.94E-05	0.9589	0.2291
11:48239182	rs7130086	A	C	0.19	9.45E-01	-0.0170	0.2470	3.38E-01	-0.2901	0.3063	1.59E-04	-0.8660	0.2140
11:48239908	rs74750395	G	T	0.88	9.79E-01	0.0071	0.2665	4.46E-02	0.7225	0.3735	9.97E-05	0.9592	0.2292
11:48248536	rs12221987	C	T	0.88	9.71E-01	0.0097	0.2642	5.09E-02	0.6927	0.3674	9.06E-05	0.9557	0.2268

11:48250675	rs7127946	T	C	0.74	8.26E-01	-0.0441	0.2006	8.63E-01	-0.0474	0.2791	8.42E-05	0.7290	0.1721
11:48251678	rs12792956	A	C	0.88	9.77E-01	0.0077	0.2669	4.54E-02	0.7210	0.3740	9.99E-05	0.9605	0.2295
11:48252957	rs185685972	A	G	0.88	9.76E-01	0.0080	0.2670	4.55E-02	0.7209	0.3741	1.00E-04	0.9608	0.2296
11:48254966	rs11529375	T	C	0.88	9.76E-01	0.0079	0.2669	4.57E-02	0.7196	0.3739	9.99E-05	0.9605	0.2295
11:48254967	rs11530183	G	A	0.88	9.76E-01	0.0079	0.2669	4.57E-02	0.7196	0.3739	9.99E-05	0.9605	0.2295
11:48259613	rs10838842	A	C	0.88	9.76E-01	0.0080	0.2669	4.62E-02	0.7180	0.3737	9.98E-05	0.9605	0.2295
11:48260741	rs11039605	A	C	0.88	9.76E-01	0.0081	0.2669	4.63E-02	0.7176	0.3737	1.00E-04	0.9605	0.2296
11:48260817	rs11039606	T	C	0.88	9.76E-01	0.0081	0.2669	4.63E-02	0.7176	0.3737	1.00E-04	0.9605	0.2296
11:48261751	rs12802708	T	C	0.88	9.76E-01	0.0081	0.2669	4.63E-02	0.7176	0.3737	1.00E-04	0.9605	0.2296
11:48262328	rs2134555	A	G	0.75	8.93E-01	-0.0280	0.2087	9.81E-01	-0.0066	0.2806	5.30E-05	0.7764	0.1776
11:48263354	rs11039609	G	C	0.88	9.76E-01	0.0082	0.2669	4.64E-02	0.7170	0.3737	9.99E-05	0.9605	0.2295
11:48278054	rs877719	A	T	0.88	9.73E-01	0.0090	0.2667	4.78E-02	0.7112	0.3728	9.96E-05	0.9598	0.2293
11:48284033	rs1109906	T	C	0.75	8.95E-01	-0.0271	0.2065	9.82E-01	-0.0061	0.2770	5.02E-05	0.7704	0.1756
11:48289368	rs11437042	I	R	0.88	9.78E-01	0.0074	0.2664	4.48E-02	0.7217	0.3734	9.87E-05	0.9593	0.2291
11:48292169	rs140845962	I	R	0.88	9.79E-01	0.0069	0.2664	4.51E-02	0.7209	0.3733	9.85E-05	0.9593	0.2290
11:48292172	rs202093789	I	R	0.88	9.79E-01	0.0069	0.2664	4.51E-02	0.7209	0.3733	9.85E-05	0.9593	0.2290
11:48292258	rs7479943	A	T	0.88	9.78E-01	0.0072	0.2664	4.48E-02	0.7218	0.3734	9.88E-05	0.9593	0.2291
11:48298269	rs4980430	A	G	0.75	8.84E-01	-0.0302	0.2074	9.79E-01	-0.0073	0.2775	4.49E-05	0.7781	0.1760
11:48303781	rs11530186	G	C	0.88	9.77E-01	0.0078	0.2669	4.38E-02	0.7279	0.3751	9.94E-05	0.9605	0.2295
11:48306990	rs11039638	T	G	0.75	8.87E-01	-0.0294	0.2076	9.81E-01	-0.0066	0.2784	4.66E-05	0.7772	0.1763
11:48307020	rs11039639	C	T	0.88	9.77E-01	0.0078	0.2670	4.35E-02	0.7299	0.3756	9.97E-05	0.9610	0.2296
11:48317706	rs7113825	C	T	0.85	9.28E-01	-0.0241	0.2687	4.32E-01	0.2622	0.3399	9.19E-05	0.9712	0.2307
11:48327476	rs12288484	G	T	0.75	8.97E-01	-0.0267	0.2073	9.89E-01	-0.0040	0.2807	5.35E-05	0.7709	0.1765
11:48328644	rs753095	C	T	0.75	8.97E-01	-0.0268	0.2071	9.87E-01	-0.0044	0.2801	5.29E-05	0.7703	0.1762
11:48347867	rs11039679	A	G	0.88	9.80E-01	0.0066	0.2664	4.42E-02	0.7238	0.3735	9.96E-05	0.9587	0.2291
11:48349903	rs11039681	G	A	0.88	9.80E-01	0.0067	0.2666	4.43E-02	0.7240	0.3738	9.95E-05	0.9595	0.2292
11:48350928	rs34995021	C	G	0.88	9.81E-01	0.0064	0.2664	4.42E-02	0.7239	0.3736	9.94E-05	0.9589	0.2291
11:48351216	rs11039682	T	C	0.88	9.81E-01	0.0065	0.2664	4.43E-02	0.7236	0.3735	9.94E-05	0.9589	0.2291
11:48351483	rs146463725	R	D	0.88	9.81E-01	0.0065	0.2664	4.43E-02	0.7237	0.3735	9.93E-05	0.9589	0.2291
11:48352204	rs1483116	C	T	0.75	8.94E-01	-0.0274	0.2066	9.82E-01	-0.0062	0.2772	4.99E-05	0.7709	0.1757
11:48360648	rs11039685	G	A	0.88	9.80E-01	0.0066	0.2665	4.44E-02	0.7236	0.3736	9.94E-05	0.9592	0.2291
11:48365325	rs11039688	G	C	0.88	9.80E-01	0.0066	0.2666	4.45E-02	0.7235	0.3737	9.94E-05	0.9596	0.2292
11:48371317	rs74824994	G	A	0.88	1.00E+00	-0.0001	0.2672	4.23E-02	0.7324	0.3749	9.32E-05	0.9651	0.2295
11:48371320	rs75731910	G	A	0.88	9.79E-01	0.0070	0.2670	4.38E-02	0.7268	0.3745	9.95E-05	0.9609	0.2296

11:48373407	rs71477930	G	A	0.88	9.81E-01	0.0065	0.2667	4.46E-02	0.7233	0.3740	9.97E-05	0.9600	0.2294
11:48373946	rs12797020	C	A	0.88	9.80E-01	0.0066	0.2667	4.47E-02	0.7232	0.3740	9.97E-05	0.9600	0.2294
11:48374173	rs11039694	G	T	0.88	9.80E-01	0.0066	0.2668	4.47E-02	0.7233	0.3740	9.97E-05	0.9600	0.2294
11:48380203	rs11039695	A	C	0.88	9.80E-01	0.0067	0.2668	4.46E-02	0.7235	0.3740	9.98E-05	0.9600	0.2294
11:48383465	rs71477931	C	T	0.88	9.79E-01	0.0069	0.2667	4.46E-02	0.7235	0.3739	9.94E-05	0.9601	0.2294
11:48391949	rs5791840	R	D	0.76	5.31E-01	-0.1475	0.2356	1.91E-01	0.3929	0.2981	7.35E-05	0.8647	0.2023
11:48508864	rs544306326	I	R	0.76	8.30E-01	-0.0604	0.2811	1.37E-01	0.5506	0.3760	9.82E-05	1.0131	0.2418
11:48568330	rs7482386	G	A	0.87	9.79E-01	0.0070	0.2679	6.01E-02	0.6715	0.3690	8.78E-05	0.9709	0.2299
11:48579092	rs7937596	T	C	0.88	9.80E-01	0.0068	0.2678	4.61E-02	0.7207	0.3752	9.99E-05	0.9637	0.2303
11:48580210	rs7105752	C	T	0.88	9.79E-01	0.0071	0.2674	4.60E-02	0.7201	0.3747	9.98E-05	0.9623	0.2299
11:48580821	rs6485873	C	T	0.88	9.79E-01	0.0071	0.2674	4.60E-02	0.7201	0.3747	9.98E-05	0.9623	0.2299
11:48581482	rs35552667	D	R	0.88	9.79E-01	0.0069	0.2674	4.58E-02	0.7207	0.3747	9.97E-05	0.9625	0.2300
11:48584029	rs7479629	T	C	0.88	9.78E-01	0.0073	0.2677	4.57E-02	0.7219	0.3751	9.99E-05	0.9632	0.2302
11:48595854	rs5791848	D	R	0.88	9.79E-01	0.0071	0.2681	4.60E-02	0.7234	0.3764	9.97E-05	0.9648	0.2305
11:48596547	rs11039847	C	G	0.80	7.97E-01	-0.0615	0.2399	4.77E-01	0.2309	0.3257	4.21E-05	0.9029	0.2034
11:48596818	rs10742856	C	T	0.87	9.81E-01	0.0062	0.2678	4.67E-02	0.7176	0.3741	9.81E-05	0.9646	0.2302
11:48596955	rs10742857	T	C	0.83	9.32E-01	0.0241	0.2824	3.94E-02	0.7728	0.3895	9.24E-05	1.0206	0.2426
11:48596959	rs36176069	T	C	0.83	9.32E-01	0.0241	0.2824	3.94E-02	0.7728	0.3895	9.24E-05	1.0206	0.2426
11:48596962	rs10742858	T	C	0.83	9.32E-01	0.0240	0.2824	3.93E-02	0.7729	0.3894	9.30E-05	1.0202	0.2426
11:48624725	rs12421145	C	A	0.41	2.49E-01	0.2535	0.2191	6.78E-01	-0.1262	0.3101	9.98E-05	-0.7977	0.1906
11:48629092	rs7481000	T	A	0.38	2.73E-01	0.2647	0.2405	4.72E-01	-0.2378	0.3383	8.32E-05	-0.8832	0.2084
11:49108070	rs150946011	D	R	0.88	4.30E-01	-0.2706	0.3427	2.03E-01	0.6036	0.4710	1.36E-05	1.3645	0.2866
11:60527294	rs10897110	T	C	0.58	7.64E-05	0.7935	0.1873	1.11E-01	-0.3828	0.2402	8.07E-01	0.0518	0.2114
11:69515696	rs79825511	G	A	0.87	7.22E-02	0.6476	0.3558	7.59E-01	-0.1350	0.4380	5.20E-06	-1.5068	0.2995
11:69516025	rs72475636	C	T	0.87	6.64E-02	0.6253	0.3363	1.93E-01	-0.5122	0.3855	1.09E-05	-1.3847	0.2870
11:69524103	rs10752563	A	G	0.86	1.21E-01	0.5106	0.3265	2.44E-01	-0.4466	0.3776	2.63E-05	-1.2830	0.2805
11:69525868	rs138542855	C	T	0.88	1.09E-01	0.5209	0.3221	2.56E-01	-0.4300	0.3714	1.99E-05	-1.2833	0.2758
11:69526015	rs72475637	T	C	0.88	1.07E-01	0.5240	0.3220	2.54E-01	-0.4311	0.3714	1.99E-05	-1.2835	0.2758
11:69526788	rs12419388	A	G	0.88	1.12E-01	0.5077	0.3163	2.71E-01	-0.4097	0.3656	2.61E-05	-1.2444	0.2720
11:69530290	rs7114345	G	A	0.87	1.26E-01	0.4902	0.3174	3.24E-01	-0.3678	0.3685	2.25E-05	-1.2554	0.2718
11:69530390	rs79079926	C	A	0.87	1.18E-01	0.4973	0.3152	2.95E-01	-0.3885	0.3653	2.44E-05	-1.2429	0.2705
11:69533873	rs73526337	C	G	0.85	8.61E-02	0.5539	0.3192	3.96E-01	-0.3232	0.3763	1.21E-05	-1.3036	0.2719
11:69534411	rs56656046	C	A	0.86	1.23E-01	0.4921	0.3165	3.86E-01	-0.3251	0.3706	2.97E-05	-1.2363	0.2724
11:69536350	rs61666930	G	C	0.87	1.33E-01	0.4764	0.3146	3.98E-01	-0.3157	0.3685	4.06E-05	-1.2094	0.2719

11:69537443	rs60603533	G	A	0.86	1.34E-01	0.4743	0.3138	4.04E-01	-0.3109	0.3678	4.16E-05	-1.2048	0.2713
11:69538558	rs72475638	A	C	0.87	1.36E-01	0.4708	0.3137	4.11E-01	-0.3067	0.3679	4.45E-05	-1.2003	0.2714
11:69538960	rs74852454	C	T	0.86	1.37E-01	0.4696	0.3133	4.12E-01	-0.3053	0.3675	4.41E-05	-1.1990	0.2710
11:69540514	rs59590661	G	A	0.86	1.41E-01	0.4634	0.3125	4.26E-01	-0.2959	0.3670	4.88E-05	-1.1897	0.2707
11:71956899	rs677685	G	C	0.51	1.38E-01	-0.3023	0.2023	5.39E-05	0.8188	0.2018	9.31E-01	0.0176	0.2028
11:71966582	rs628542	C	A	0.54	1.61E-01	-0.2684	0.1900	7.80E-05	0.7574	0.1913	7.23E-01	-0.0676	0.1899
11:75968200	rs11606029	C	T	0.84	7.91E-05	-0.9580	0.2266	5.47E-04	1.3869	0.4305	2.86E-02	0.5506	0.2452
11:76043831	rs11236718	G	A	0.79	9.91E-05	-0.9464	0.2275	3.78E-02	0.6936	0.3437	4.20E-01	0.2065	0.2543
11:76048876	rs2851473	T	C	0.79	9.05E-05	-0.9463	0.2260	4.30E-02	0.6718	0.3420	4.27E-01	0.2027	0.2531
11:78356497	rs12279681	C	T	0.35	6.62E-05	0.7491	0.1751	4.59E-01	-0.1983	0.2691	5.08E-02	-0.3824	0.1916
11:78356498	rs1945465	T	C	0.35	6.60E-05	0.7494	0.1751	4.58E-01	-0.1988	0.2692	5.10E-02	-0.3821	0.1917
11:78357331	rs11237577	A	G	0.35	6.45E-05	0.7489	0.1747	4.58E-01	-0.1986	0.2687	5.14E-02	-0.3807	0.1913
11:78357427	rs11237578	T	G	0.35	6.43E-05	0.7489	0.1747	4.59E-01	-0.1983	0.2687	5.15E-02	-0.3805	0.1913
11:78358037	rs2282650	A	G	0.35	5.69E-05	0.7518	0.1739	4.57E-01	-0.1988	0.2683	5.07E-02	-0.3809	0.1908
11:78358691	rs3740826	C	T	0.35	5.67E-05	0.7517	0.1738	4.56E-01	-0.1990	0.2681	5.09E-02	-0.3804	0.1908
11:78358779	rs3740827	T	C	0.66	9.64E-05	-0.7318	0.1756	3.52E-01	0.2505	0.2714	6.78E-02	0.3570	0.1918
11:78359115	rs12793480	A	G	0.35	5.83E-05	0.7513	0.1741	4.55E-01	-0.1998	0.2684	5.24E-02	-0.3784	0.1910
11:78377097	rs34566831	R	I	0.39	5.41E-05	0.7790	0.1796	2.32E-01	-0.3109	0.2632	8.59E-02	-0.3474	0.1988
11:78388699	rs55797205	R	I	0.36	6.41E-05	0.7650	0.1784	5.70E-01	-0.1496	0.2633	4.11E-02	-0.4070	0.1947
11:80647064	rs1391534	C	T	0.44	9.92E-05	-0.6366	0.1530	3.43E-01	0.2026	0.2118	9.28E-01	0.0155	0.1720
11:80648283	rs35852879	R	D	0.48	8.62E-05	-0.6862	0.1633	2.13E-01	0.2818	0.2249	8.35E-01	0.0386	0.1840
11:82009461	rs10897952	A	T	0.47	6.73E-05	0.7284	0.1704	2.24E-01	-0.3106	0.2546	6.95E-01	-0.0759	0.1926
12:119326370	rs4362197	C	A	0.88	1.02E-01	-0.4878	0.2954	8.63E-05	1.9911	0.5950	2.69E-01	0.3287	0.2942
12:119332567	rs7297419	G	A	0.89	4.69E-02	-0.6056	0.3001	9.13E-05	2.0832	0.6365	2.04E-01	0.3873	0.3012
12:119333209	rs149183744	R	D	0.89	5.13E-02	-0.5963	0.3015	9.38E-05	2.0734	0.6338	1.87E-01	0.4030	0.3019
12:119335638	rs10851051	G	A	0.89	4.17E-02	-0.6201	0.2995	8.26E-05	2.1134	0.6458	1.98E-01	0.3922	0.3010
12:119338572	rs7131867	A	G	0.89	3.90E-02	-0.6275	0.2989	7.86E-05	2.1344	0.6544	1.88E-01	0.4008	0.3005
12:119340380	rs34232964	G	C	0.89	3.76E-02	-0.6309	0.2982	7.76E-05	2.1445	0.6599	1.82E-01	0.4050	0.2999
12:119380540	rs7964215	G	A	0.92	1.53E-01	-0.5656	0.3929	8.40E-05	4.4475	2.2944	4.25E-02	0.7873	0.3793
12:119382394	rs4575336	G	A	0.93	1.11E-01	-0.6850	0.4253	9.94E-05	4.5241	2.2475	5.04E-02	0.8266	0.4135
12:119383059	rs1464949	A	G	0.93	1.09E-01	-0.6873	0.4254	9.60E-05	4.5786	2.2881	5.03E-02	0.8271	0.4135
12:119383296	rs143874569	R	D	0.93	1.08E-01	-0.6922	0.4266	9.60E-05	4.6072	2.3067	4.99E-02	0.8310	0.4148
12:12225207	rs10845469	C	T	0.59	6.55E-01	-0.0824	0.1849	3.59E-01	0.2086	0.2271	8.90E-05	0.6708	0.1590
12:19573650	rs10841227	A	G	0.40	5.27E-05	0.8564	0.1971	1.25E-01	-0.4018	0.2659	1.12E-01	-0.3534	0.2190

12:19578994	rs6486955	T	C	0.39	5.58E-05	0.8521	0.1968	1.30E-01	-0.3959	0.2647	1.19E-01	-0.3459	0.2187
12:19597097	rs4963527	T	A	0.39	5.30E-05	0.8542	0.1967	1.44E-01	-0.3835	0.2652	1.41E-01	-0.3274	0.2192
12:19599034	rs140386958	R	I	0.43	6.19E-05	0.8223	0.1913	1.10E-01	-0.4148	0.2591	8.74E-02	-0.3674	0.2113
12:19601137	rs7309303	T	G	0.42	6.17E-05	0.8197	0.1906	1.14E-01	-0.4097	0.2584	9.39E-02	-0.3591	0.2108
12:19604687	rs61912766	G	A	0.42	6.08E-05	0.8150	0.1894	1.19E-01	-0.4018	0.2570	1.05E-01	-0.3454	0.2097
12:19606213	rs11044571	A	T	0.42	5.97E-05	0.8158	0.1893	1.16E-01	-0.4057	0.2572	1.05E-01	-0.3454	0.2098
12:19609503	rs10734689	A	G	0.38	6.04E-05	0.8407	0.1953	1.55E-01	-0.3692	0.2621	1.74E-01	-0.3000	0.2178
12:19613820	rs7973261	T	C	0.38	6.01E-05	0.8408	0.1952	1.56E-01	-0.3688	0.2622	1.76E-01	-0.2985	0.2178
12:19621227	rs11044579	C	G	0.38	6.11E-05	0.8403	0.1953	1.56E-01	-0.3687	0.2625	1.80E-01	-0.2955	0.2179
12:19623533	rs10743324	C	T	0.42	6.28E-05	0.8095	0.1885	1.21E-01	-0.3991	0.2565	1.14E-01	-0.3350	0.2089
12:19634497	rs4963541	T	C	0.38	6.18E-05	0.8405	0.1955	1.58E-01	-0.3678	0.2628	1.84E-01	-0.2934	0.2181
12:19634580	rs4963542	A	T	0.38	6.18E-05	0.8404	0.1955	1.58E-01	-0.3678	0.2627	1.84E-01	-0.2934	0.2181
12:19647476	rs10770493	G	C	0.38	5.52E-05	0.8523	0.1967	1.54E-01	-0.3744	0.2652	1.92E-01	-0.2907	0.2201
12:19649096	rs10770496	T	C	0.41	5.21E-05	0.8197	0.1885	1.22E-01	-0.3995	0.2576	1.02E-01	-0.3477	0.2092
12:20665611	rs7305373	A	G	0.94	4.60E-01	0.3144	0.4253	2.19E-05	18762.3000	6.69E+06	6.72E-01	-0.1787	0.4195
12:25055512	rs3759085	A	G	0.66	5.39E-05	0.8179	0.1885	3.90E-02	-0.4994	0.2307	9.50E-03	-0.5416	0.2018
12:25056378	rs3759086	T	A	0.57	9.33E-05	0.7188	0.1720	5.32E-02	-0.4555	0.2305	2.59E-02	-0.4238	0.1853
12:25057206	rs3759087	A	G	0.55	6.04E-05	0.7174	0.1666	6.46E-02	-0.4382	0.2328	5.74E-02	-0.3549	0.1830
12:25058546	rs4963818	G	C	0.67	9.65E-05	0.8026	0.1926	5.89E-02	-0.4596	0.2318	1.46E-02	-0.5178	0.2055
12:3353636	rs7959621	C	T	0.66	4.18E-01	-0.1632	0.2013	6.11E-01	0.1306	0.2611	7.40E-05	0.7403	0.1732
12:3357135	rs660140	G	C	0.41	1.44E-01	0.2724	0.1849	3.06E-01	-0.2452	0.2442	2.74E-05	-0.7232	0.1586
12:3357785	rs140604579	R	D	0.41	1.51E-01	0.2645	0.1830	3.33E-01	-0.2286	0.2400	1.52E-05	-0.7343	0.1553
12:3357806	rs11418309	R	I	0.65	8.11E-02	-0.3460	0.1961	5.92E-01	0.1341	0.2563	4.17E-05	0.7581	0.1707
12:3357992	rs3782788	T	G	0.67	6.67E-02	-0.3607	0.1942	7.06E-01	0.0972	0.2608	8.99E-06	0.8059	0.1652
12:40202563	rs11173669	C	T	0.90	2.42E-01	0.4165	0.3544	1.49E-01	-0.5402	0.3649	2.34E-05	-1.3869	0.3011
12:42368632	rs9739108	C	T	0.73	2.45E-02	0.4958	0.2160	1.98E-05	-1.0466	0.2316	1.42E-01	-0.3244	0.2179
12:53000987	rs670142	C	A	0.71	2.61E-03	-0.5792	0.1851	1.77E-01	0.3648	0.2766	2.13E-06	0.8528	0.1616
12:545890	rs16930719	C	T	0.78	3.77E-02	-0.4656	0.2203	9.68E-05	1.3055	0.3742	2.92E-01	0.2369	0.2228
12:58607312	rs58467005	R	D	0.36	3.84E-01	-0.1708	0.1958	3.27E-05	0.8130	0.1924	4.91E-01	0.1337	0.1930
12:58609457	rs7970164	T	C	0.67	3.66E-01	0.1749	0.1933	3.58E-05	-0.7955	0.1883	3.93E-01	-0.1636	0.1902
12:58609685	rs10877082	G	A	0.38	3.94E-01	-0.1676	0.1963	3.41E-05	0.8647	0.2053	8.67E-01	0.0327	0.1942
12:58614449	rs10783885	T	C	0.38	3.79E-01	-0.1735	0.1969	2.51E-05	0.8896	0.2081	8.25E-01	0.0432	0.1949
12:58622382	rs11172503	G	A	0.39	3.58E-01	-0.1815	0.1971	1.60E-05	0.9284	0.2133	7.53E-01	0.0616	0.1951
12:58622987	rs139205516	A	G	0.39	3.57E-01	-0.1820	0.1971	1.57E-05	0.9297	0.2135	7.50E-01	0.0625	0.1951

12:58626004	rs10877083	T	C	0.39	3.50E-01	-0.1844	0.1968	1.40E-05	0.9404	0.2152	7.25E-01	0.0690	0.1948
12:58638253	rs12423429	G	T	0.80	5.64E-01	-0.1459	0.2529	6.74E-05	-1.2958	0.2940	3.61E-01	-0.3873	0.4205
12:58640325	rs1177777	G	A	0.39	3.50E-01	-0.1860	0.1985	1.35E-05	0.9522	0.2177	7.18E-01	0.0713	0.1965
12:58640529	rs1181377	T	C	0.39	3.62E-01	-0.1817	0.1990	1.31E-05	0.9559	0.2183	7.24E-01	0.0699	0.1969
12:58738035	rs1729795	T	G	0.48	1.06E-01	-0.2850	0.1747	9.19E-05	0.8025	0.2084	9.07E-02	0.2949	0.1714
12:62861745	rs10877853	T	C	0.62	4.68E-01	-0.1310	0.1805	9.03E-05	0.8819	0.2391	7.54E-01	0.0561	0.1782
12:63614956	rs11174871	A	G	0.71	1.25E-05	-0.9161	0.1927	6.56E-02	0.5733	0.3177	9.27E-01	0.0207	0.2243
12:70445461	rs149839455	A	G	0.93	8.77E-05	1.4710	0.3505	6.03E-02	-0.7912	0.3986	6.92E-01	-0.1568	0.3944
12:85814040	rs559707085	R	I	0.95	6.95E-02	0.8547	0.4649	6.70E-01	-0.2519	0.5769	7.30E-05	-1.7515	0.4095
12:85834860	rs7963815	G	A	0.95	8.25E-02	0.8018	0.4565	6.64E-01	-0.2521	0.5669	3.76E-05	-1.7732	0.3966
12:85873291	rs60552633	T	C	0.95	1.28E-01	0.6781	0.4415	5.60E-01	-0.3209	0.5328	7.29E-05	-1.6491	0.3855
12:85873925	rs55897040	A	T	0.95	1.28E-01	0.6779	0.4414	5.59E-01	-0.3217	0.5326	7.26E-05	-1.6491	0.3854
12:85877237	rs59712244	T	A	0.95	1.26E-01	0.6840	0.4429	5.59E-01	-0.3227	0.5345	7.19E-05	-1.6558	0.3867
12:85878293	rs112271310	R	D	0.95	1.26E-01	0.6839	0.4428	5.61E-01	-0.3211	0.5347	7.19E-05	-1.6557	0.3867
12:85883863	rs11116842	G	A	0.95	1.26E-01	0.6823	0.4423	5.61E-01	-0.3208	0.5341	7.20E-05	-1.6537	0.3862
12:85884507	rs11116843	C	T	0.95	1.26E-01	0.6818	0.4423	5.61E-01	-0.3206	0.5341	7.21E-05	-1.6533	0.3862
12:85884858	rs11116844	C	A	0.95	1.27E-01	0.6812	0.4422	5.61E-01	-0.3205	0.5340	7.19E-05	-1.6531	0.3861
12:85885635	rs74813881	A	T	0.95	1.27E-01	0.6805	0.4420	5.61E-01	-0.3201	0.5337	7.17E-05	-1.6525	0.3859
12:85887583	rs79996161	A	G	0.95	1.31E-01	0.6705	0.4400	5.60E-01	-0.3197	0.5309	7.31E-05	-1.6428	0.3841
12:85889505	rs111601198	R	D	0.95	1.28E-01	0.6783	0.4416	5.61E-01	-0.3204	0.5332	7.21E-05	-1.6506	0.3856
12:85893589	rs74112063	A	G	0.95	1.32E-01	0.6661	0.4392	5.59E-01	-0.3196	0.5297	7.36E-05	-1.6388	0.3834
12:85895211	rs12229603	T	C	0.95	1.29E-01	0.6743	0.4408	5.59E-01	-0.3214	0.5318	7.22E-05	-1.6472	0.3848
12:85895595	rs11116848	G	A	0.95	1.28E-01	0.6767	0.4409	5.63E-01	-0.3181	0.5325	7.30E-05	-1.6467	0.3850
12:85899173	rs57279875	C	T	0.95	1.28E-01	0.6757	0.4408	5.63E-01	-0.3178	0.5323	7.33E-05	-1.6458	0.3849
12:85907827	rs74112083	C	G	0.94	8.73E-02	0.7037	0.4071	1.19E-01	-0.6683	0.3998	9.60E-05	-1.5065	0.3590
12:85908231	rs11116852	A	T	0.95	1.35E-01	0.6586	0.4375	5.59E-01	-0.3189	0.5276	7.42E-05	-1.6315	0.3819
12:85909987	rs74112086	G	T	0.95	1.32E-01	0.6667	0.4392	5.60E-01	-0.3191	0.5300	7.32E-05	-1.6394	0.3834
12:85912432	rs7961629	G	A	0.95	1.32E-01	0.6671	0.4392	5.60E-01	-0.3191	0.5301	7.44E-05	-1.6382	0.3835
12:85912600	rs57059247	G	A	0.95	1.32E-01	0.6662	0.4391	5.60E-01	-0.3193	0.5298	7.24E-05	-1.6401	0.3832
12:89237659	rs7303698	C	A	0.89	7.88E-05	1.2398	0.2932	2.40E-01	-0.4151	0.3480	7.78E-01	-0.0935	0.3308
12:93758149	rs67475674	R	D	0.71	6.16E-02	-0.4973	0.2625	2.25E-01	0.4192	0.3507	6.42E-05	0.9974	0.2311
12:96549543	rs256200	G	A	0.72	1.87E-05	0.8386	0.1807	6.69E-01	-0.1115	0.2592	2.27E-02	-0.4674	0.1996
12:96549634	rs256199	T	C	0.72	1.78E-05	0.8356	0.1795	6.61E-01	-0.1137	0.2579	2.28E-02	-0.4642	0.1984
12:96552711	rs256198	A	T	0.71	1.57E-05	0.8659	0.1846	7.85E-01	-0.0737	0.2686	3.08E-02	-0.4550	0.2054

13:102477248	rs3007763	A	G	0.73	7.40E-05	-0.8521	0.2007	3.28E-02	0.6267	0.2845	6.07E-01	0.1170	0.2262
13:108033035	rs9559031	C	T	0.89	2.08E-01	0.5544	0.4376	3.62E-01	-0.4742	0.5187	8.07E-05	-1.6154	0.3803
13:111217422	rs766974	G	A	0.68	2.97E-01	-0.2103	0.2012	7.20E-05	1.0911	0.2917	2.16E-01	0.2464	0.1970
13:20812200	rs71198966	R	I	0.39	7.93E-05	0.8448	0.1999	4.97E-01	-0.2129	0.3188	7.29E-01	-0.0784	0.2254
13:29364808	rs78353449	G	A	0.94	2.80E-05	-1.6166	0.3571	6.32E-02	1.5718	1.0604	5.59E-02	0.7751	0.3971
13:30310959	rs7327855	G	T	0.63	4.06E-05	0.7953	0.1799	2.45E-03	-0.8022	0.2683	8.97E-03	-0.5231	0.1933
13:35209765	rs79529743	C	G	0.92	3.10E-01	0.3352	0.3295	4.49E-05	-1.0611	0.2232	5.46E-01	-0.1978	0.3259
13:57555759	rs9537525	A	T	0.46	1.93E-02	0.5009	0.2094	2.08E-02	-0.6401	0.2827	7.32E-05	-0.8043	0.1881
13:57556412	rs9537526	C	T	0.46	2.12E-02	0.4987	0.2118	2.35E-02	-0.6307	0.2842	7.86E-05	-0.8091	0.1901
13:57556464	rs9527566	G	A	0.46	2.12E-02	0.4987	0.2118	2.35E-02	-0.6309	0.2842	7.88E-05	-0.8090	0.1901
13:57556511	rs7490316	A	G	0.46	2.12E-02	0.4986	0.2118	2.35E-02	-0.6309	0.2842	7.88E-05	-0.8090	0.1901
13:57556765	rs6561905	C	T	0.46	2.15E-02	0.4973	0.2117	2.36E-02	-0.6300	0.2841	7.85E-05	-0.8085	0.1900
13:57566485	rs9316881	G	A	0.46	1.92E-02	0.5007	0.2091	2.08E-02	-0.6393	0.2824	7.30E-05	-0.8032	0.1878
13:57567539	rs9537541	G	A	0.46	1.92E-02	0.5007	0.2091	2.08E-02	-0.6394	0.2824	7.32E-05	-0.8031	0.1878
13:57570035	rs148081853	R	I	0.46	1.92E-02	0.5008	0.2091	2.08E-02	-0.6396	0.2824	7.33E-05	-0.8032	0.1878
13:57570394	rs201113483	R	D	0.46	1.93E-02	0.5007	0.2092	2.08E-02	-0.6396	0.2824	7.34E-05	-0.8033	0.1879
13:57581787	rs1591602	G	A	0.46	1.95E-02	0.5004	0.2095	2.06E-02	-0.6407	0.2825	7.44E-05	-0.8037	0.1881
13:57680484	rs200767240	R	I	0.60	2.28E-02	0.4332	0.1863	1.97E-02	-0.5030	0.2155	8.61E-05	-0.7075	0.1673
13:57714724	rs3967795	C	T	0.44	4.17E-03	0.6570	0.2215	5.72E-03	-0.8303	0.3036	8.21E-05	-0.8663	0.2042
13:57714737	rs372036953	A	G	0.46	4.73E-03	0.6274	0.2147	1.44E-02	-0.7004	0.2922	1.82E-05	-0.9010	0.1926
13:57759469	rs9591802	C	T	0.58	1.58E-02	-0.4730	0.1913	1.04E-01	0.3747	0.2319	6.05E-05	0.7442	0.1718
13:57764148	rs34077742	I	R	0.58	1.57E-02	-0.4740	0.1915	1.04E-01	0.3755	0.2326	6.25E-05	0.7440	0.1721
13:58248124	rs9597584	C	A	0.62	1.43E-01	-0.2850	0.1929	2.52E-01	0.2592	0.2335	8.03E-05	0.7157	0.1684
13:58591940	rs34941924	I	R	0.88	1.20E-01	-0.5217	0.3330	1.04E-01	0.7318	0.4541	1.40E-05	1.3454	0.2830
13:65005844	rs57721572	R	D	0.64	7.23E-05	-0.8287	0.1948	1.53E-01	0.3952	0.2780	5.35E-01	0.1371	0.2195
13:71301595	rs78215204	A	G	0.93	1.54E-01	0.6648	0.4626	1.69E-01	-0.7129	0.5061	5.32E-05	-1.7517	0.4008
13:81146008	rs6563177	G	A	0.68	7.33E-01	0.0683	0.2005	8.88E-01	-0.0345	0.2418	7.36E-05	-0.7340	0.1717
13:81193993	rs3813531	G	C	0.44	2.91E-01	-0.1924	0.1817	4.20E-01	0.1862	0.2322	2.18E-05	0.7118	0.1538
13:81194762	rs35982872	R	I	0.71	3.61E-01	-0.2153	0.2355	5.43E-02	0.5804	0.3079	8.33E-05	0.8619	0.2034
13:81229110	rs9574710	C	T	0.86	5.46E-01	-0.1625	0.2689	5.06E-01	0.2040	0.3097	5.83E-05	-0.9987	0.2299
13:81243274	rs34835254	R	I	0.48	8.40E-01	-0.0366	0.1813	6.09E-01	0.1175	0.2288	5.75E-05	0.6718	0.1545
13:81245032	rs1176286	T	A	0.49	8.03E-01	-0.0447	0.1800	6.33E-01	0.1096	0.2287	5.77E-05	0.6669	0.1534
13:81246353	rs1094508	A	C	0.48	8.41E-01	-0.0362	0.1806	6.09E-01	0.1175	0.2283	5.48E-05	0.6710	0.1538
13:81260479	rs822176	C	T	0.48	8.57E-01	-0.0322	0.1792	5.95E-01	0.1212	0.2264	4.97E-05	0.6689	0.1524

13:81260808	rs1744600	G	C	0.48	8.59E-01	-0.0318	0.1793	5.95E-01	0.1210	0.2265	5.03E-05	0.6689	0.1525
13:81261472	rs822177	T	C	0.48	8.60E-01	-0.0316	0.1794	5.96E-01	0.1207	0.2266	5.05E-05	0.6690	0.1526
13:81263321	rs1094504	G	A	0.49	8.81E-01	-0.0270	0.1806	6.11E-01	0.1167	0.2283	5.46E-05	0.6707	0.1537
13:81263389	rs1094506	C	T	0.48	8.89E-01	-0.0252	0.1807	6.10E-01	0.1172	0.2284	5.56E-05	0.6706	0.1539
13:81263807	rs1578494	T	G	0.48	8.75E-01	-0.0282	0.1803	6.07E-01	0.1177	0.2279	5.54E-05	0.6694	0.1536
13:81264249	rs1856994	C	T	0.49	8.85E-01	-0.0261	0.1808	6.14E-01	0.1158	0.2286	5.61E-05	0.6707	0.1540
13:81264285	rs1856993	G	T	0.49	8.85E-01	-0.0260	0.1808	6.14E-01	0.1157	0.2287	5.62E-05	0.6707	0.1540
13:81264465	rs2794222	A	G	0.49	8.86E-01	-0.0258	0.1809	6.15E-01	0.1156	0.2287	5.65E-05	0.6706	0.1541
13:81264868	rs138449603	D	R	0.49	8.87E-01	-0.0257	0.1809	6.16E-01	0.1152	0.2289	5.68E-05	0.6708	0.1542
13:81264878	rs61706737	T	C	0.49	8.87E-01	-0.0256	0.1809	6.15E-01	0.1155	0.2288	5.68E-05	0.6707	0.1542
13:81265009	rs2759229	T	G	0.49	8.88E-01	-0.0255	0.1811	6.18E-01	0.1147	0.2289	5.57E-05	0.6720	0.1543
13:81265026	rs2794223	C	T	0.49	8.88E-01	-0.0255	0.1810	6.15E-01	0.1154	0.2289	5.70E-05	0.6707	0.1542
13:81265591	rs2759228	A	C	0.49	8.88E-01	-0.0253	0.1810	6.17E-01	0.1150	0.2290	5.73E-05	0.6708	0.1543
13:81267583	rs113602029	C	T	0.49	8.96E-01	-0.0238	0.1814	6.21E-01	0.1139	0.2295	5.99E-05	0.6707	0.1547
13:81267664	rs113135326	G	C	0.49	8.96E-01	-0.0237	0.1815	6.21E-01	0.1138	0.2296	6.00E-05	0.6709	0.1548
13:81269784	rs2014392	G	A	0.49	8.93E-01	-0.0244	0.1814	6.21E-01	0.1140	0.2295	6.24E-05	0.6692	0.1548
13:81273772	rs2794226	G	A	0.49	9.13E-01	-0.0199	0.1825	6.36E-01	0.1096	0.2310	7.18E-05	0.6683	0.1561
13:81274183	rs2794228	C	T	0.49	9.14E-01	-0.0197	0.1825	6.36E-01	0.1095	0.2311	7.23E-05	0.6683	0.1561
13:81275600	rs34740532	A	G	0.49	9.20E-01	-0.0184	0.1828	6.41E-01	0.1082	0.2315	7.53E-05	0.6679	0.1565
13:81276612	rs9574724	T	C	0.49	9.21E-01	-0.0181	0.1829	6.43E-01	0.1074	0.2317	7.72E-05	0.6675	0.1567
13:81277958	rs9574725	G	T	0.49	9.27E-01	-0.0166	0.1832	6.47E-01	0.1063	0.2321	8.02E-05	0.6671	0.1570
13:81278305	rs35461826	G	A	0.49	9.30E-01	-0.0162	0.1833	6.49E-01	0.1058	0.2322	8.13E-05	0.6669	0.1571
13:90875075	rs10047731	G	A	0.75	8.38E-01	0.0688	0.3365	9.57E-02	-0.6305	0.3762	4.67E-05	-1.2599	0.2858
13:95842190	rs78608794	T	G	0.88	7.34E-01	-0.1005	0.2967	6.24E-01	-0.1639	0.3292	4.61E-05	-1.1123	0.2521
13:95843575	rs9524822	T	C	0.88	7.51E-01	-0.0940	0.2962	6.01E-01	-0.1740	0.3280	3.96E-05	-1.1187	0.2511
13:95852179	rs9516533	A	G	0.88	8.22E-01	-0.0644	0.2866	3.96E-01	-0.2678	0.3131	3.94E-05	-1.0819	0.2427
13:95860590	rs114357938	A	G	0.88	5.65E-01	-0.1679	0.2919	3.57E-01	-0.2972	0.3193	5.96E-05	-1.0825	0.2496
13:99090166	rs59023150	D	R	0.91	6.28E-02	0.7946	0.4214	3.85E-05	-1.5493	0.3448	1.92E-01	-0.5554	0.4209
14:101528110	rs6575812	G	T	0.79	8.12E-03	-0.7342	0.2693	7.76E-05	1.4826	0.4313	9.15E-02	0.4716	0.2748
14:101528846	rs11160619	G	A	0.79	8.46E-03	-0.7333	0.2705	8.70E-05	1.4757	0.4319	8.84E-02	0.4780	0.2757
14:101533692	rs72635181	C	T	0.85	6.56E-05	-1.7904	0.4181	8.92E-03	1.7867	0.7189	4.73E-02	0.9271	0.4572
14:101632104	rs34665795	R	D	0.69	7.66E-05	-0.7291	0.1721	2.21E-01	0.2897	0.2440	1.32E-01	0.2911	0.1905
14:101634566	rs35347011	R	D	0.69	6.11E-05	-0.7531	0.1750	2.21E-01	0.2941	0.2474	1.28E-01	0.3003	0.1944
14:101634591	rs12101230	C	T	0.69	7.65E-05	-0.7288	0.1720	2.22E-01	0.2894	0.2439	1.32E-01	0.2911	0.1904

14:104756318	rs58528480	C	T	0.83	2.60E-01	0.2775	0.2455	8.54E-05	-0.8556	0.2015	7.70E-01	-0.0716	0.2439
14:21144799	rs10498273	C	G	0.94	8.69E-02	-0.7007	0.4048	3.32E-05	328.3990	242.8050	3.05E-02	0.8695	0.3919
14:26677588	rs35838189	C	T	0.88	8.30E-02	-0.5438	0.3101	7.54E-05	3.0650	1.1437	7.90E-01	-0.0836	0.3130
14:28173377	rs2154145	G	T	0.60	2.70E-01	0.2223	0.2010	6.62E-01	0.1067	0.2508	4.60E-05	-0.7610	0.1725
14:41411051	rs1176956	C	T	0.78	2.07E-01	0.2714	0.2138	4.58E-01	-0.1765	0.2352	1.90E-05	-0.8464	0.1814
14:41412218	rs34510145	I	R	0.78	2.06E-01	0.2715	0.2138	4.58E-01	-0.1765	0.2352	1.90E-05	-0.8463	0.1814
14:41413100	rs1176957	T	C	0.78	2.09E-01	0.2703	0.2139	4.60E-01	-0.1757	0.2353	1.89E-05	-0.8468	0.1814
14:41413712	rs1182565	T	C	0.78	2.06E-01	0.2716	0.2138	4.58E-01	-0.1764	0.2352	1.90E-05	-0.8461	0.1813
14:41413845	rs5808117	D	R	0.85	5.23E-01	0.1633	0.2557	4.99E-01	-0.1901	0.2775	7.23E-05	-0.9397	0.2195
14:41416288	rs1176960	T	C	0.78	2.06E-01	0.2714	0.2137	4.58E-01	-0.1763	0.2351	1.90E-05	-0.8460	0.1813
14:41416699	rs1176962	C	A	0.78	2.06E-01	0.2715	0.2137	4.58E-01	-0.1762	0.2351	1.90E-05	-0.8460	0.1813
14:41417718	rs1176964	C	T	0.78	2.06E-01	0.2715	0.2137	4.58E-01	-0.1762	0.2351	1.89E-05	-0.8460	0.1813
14:41418113	rs1615517	G	C	0.78	2.06E-01	0.2715	0.2137	4.58E-01	-0.1762	0.2351	1.89E-05	-0.8460	0.1812
14:41420230	rs76226872	G	A	0.78	2.06E-01	0.2714	0.2137	4.58E-01	-0.1761	0.2351	1.90E-05	-0.8458	0.1812
14:41420865	rs141064389	T	C	0.77	2.31E-01	0.2578	0.2143	5.64E-01	-0.1381	0.2382	4.38E-05	-0.8147	0.1841
14:41422436	rs7493481	A	G	0.77	2.28E-01	0.2562	0.2118	5.18E-01	-0.1521	0.2334	3.79E-05	-0.8111	0.1815
14:41422987	rs147526797	G	A	0.85	5.14E-01	0.1663	0.2547	5.19E-01	-0.1800	0.2763	7.35E-05	-0.9353	0.2188
14:41423886	rs1779619	T	C	0.78	2.06E-01	0.2715	0.2136	4.59E-01	-0.1760	0.2350	1.89E-05	-0.8457	0.1812
14:41423954	rs2799810	A	G	0.78	2.06E-01	0.2716	0.2137	4.58E-01	-0.1762	0.2351	1.90E-05	-0.8458	0.1812
14:41426098	rs1176965	A	G	0.78	2.05E-01	0.2724	0.2138	4.58E-01	-0.1764	0.2354	1.92E-05	-0.8459	0.1814
14:41430655	rs1176967	G	T	0.78	2.06E-01	0.2715	0.2134	4.60E-01	-0.1755	0.2349	1.89E-05	-0.8451	0.1810
14:41433519	rs11310114	D	R	0.78	2.07E-01	0.2706	0.2135	4.62E-01	-0.1746	0.2350	1.88E-05	-0.8455	0.1811
14:41434415	rs11379854	I	R	0.78	2.06E-01	0.2713	0.2134	4.60E-01	-0.1753	0.2349	1.88E-05	-0.8453	0.1810
14:41435764	rs1176970	C	G	0.85	5.13E-01	0.1661	0.2540	5.25E-01	-0.1774	0.2756	7.44E-05	-0.9322	0.2182
14:41435835	rs1176971	G	A	0.78	2.05E-01	0.2716	0.2133	4.60E-01	-0.1753	0.2349	1.88E-05	-0.8449	0.1809
14:41437680	rs1176975	A	C	0.78	2.05E-01	0.2716	0.2133	4.60E-01	-0.1751	0.2348	1.88E-05	-0.8448	0.1809
14:41443217	rs1176982	G	A	0.78	2.05E-01	0.2715	0.2133	4.61E-01	-0.1750	0.2348	1.88E-05	-0.8448	0.1809
14:41444274	rs1176983	A	T	0.78	2.05E-01	0.2715	0.2133	4.61E-01	-0.1750	0.2348	1.88E-05	-0.8449	0.1809
14:41445446	rs1176984	A	T	0.78	2.05E-01	0.2715	0.2133	4.61E-01	-0.1750	0.2348	1.88E-05	-0.8449	0.1809
14:41447232	rs1176986	G	A	0.78	2.05E-01	0.2714	0.2133	4.61E-01	-0.1750	0.2348	1.88E-05	-0.8449	0.1809
14:41448393	rs1176989	G	A	0.78	2.06E-01	0.2714	0.2134	4.61E-01	-0.1750	0.2349	1.88E-05	-0.8451	0.1810
14:41449148	rs1176990	T	G	0.78	2.07E-01	0.2705	0.2134	4.63E-01	-0.1742	0.2349	1.88E-05	-0.8453	0.1810
14:41452298	rs4630445	T	G	0.78	2.06E-01	0.2711	0.2134	4.61E-01	-0.1749	0.2348	1.87E-05	-0.8452	0.1810
14:41455080	rs2600591	C	T	0.78	2.05E-01	0.2720	0.2134	4.62E-01	-0.1747	0.2348	1.86E-05	-0.8457	0.1810

14:41455966	N/A	R	I	0.89	2.98E-01	0.3330	0.3189	4.45E-01	-0.2654	0.3463	6.62E-05	-1.1841	0.2750
14:41458047	rs2600589	T	G	0.78	1.99E-01	0.2765	0.2140	4.67E-01	-0.1725	0.2350	1.74E-05	-0.8508	0.1813
14:41461010	rs1779591	T	A	0.78	1.95E-01	0.2790	0.2139	4.67E-01	-0.1727	0.2349	1.71E-05	-0.8513	0.1813
14:41461372	rs1669561	T	C	0.78	1.96E-01	0.2781	0.2141	4.69E-01	-0.1719	0.2350	1.71E-05	-0.8520	0.1814
14:41465511	rs1779594	T	A	0.78	1.92E-01	0.2810	0.2140	4.68E-01	-0.1720	0.2348	1.69E-05	-0.8521	0.1813
14:41465710	rs1779595	A	G	0.78	1.91E-01	0.2811	0.2140	4.68E-01	-0.1720	0.2348	1.69E-05	-0.8522	0.1813
14:41466010	rs1451956	A	T	0.85	5.13E-01	0.1659	0.2536	5.26E-01	-0.1764	0.2751	7.45E-05	-0.9303	0.2178
14:41466269	rs1451955	T	C	0.85	5.13E-01	0.1659	0.2536	5.26E-01	-0.1764	0.2751	7.45E-05	-0.9303	0.2178
14:41467742	rs1615343	T	G	0.85	5.13E-01	0.1658	0.2535	5.27E-01	-0.1762	0.2750	7.44E-05	-0.9300	0.2177
14:41468933	rs1779597	G	C	0.78	1.88E-01	0.2831	0.2139	4.70E-01	-0.1713	0.2349	1.69E-05	-0.8521	0.1813
14:41469731	rs1779598	G	A	0.77	1.77E-01	0.2893	0.2129	4.79E-01	-0.1676	0.2345	1.80E-05	-0.8467	0.1808
14:41471849	rs7159099	G	A	0.77	1.74E-01	0.2908	0.2128	4.62E-01	-0.1742	0.2345	1.79E-05	-0.8465	0.1807
14:41472098	rs1779602	C	T	0.76	2.54E-01	0.2488	0.2172	4.95E-01	-0.1616	0.2347	2.30E-05	-0.8501	0.1843
14:41472170	rs10654526	R	I	0.76	2.53E-01	0.2490	0.2172	4.95E-01	-0.1617	0.2347	2.30E-05	-0.8504	0.1844
14:41472182	rs1669597	A	G	0.76	2.54E-01	0.2487	0.2172	4.95E-01	-0.1617	0.2347	2.30E-05	-0.8502	0.1844
14:41472581	rs1669595	G	A	0.76	2.53E-01	0.2491	0.2170	4.95E-01	-0.1616	0.2346	2.33E-05	-0.8491	0.1842
14:41472941	rs34238285	R	D	0.76	2.50E-01	0.2502	0.2166	4.73E-01	-0.1695	0.2343	2.52E-05	-0.8446	0.1842
14:41473413	rs1779603	C	A	0.76	2.52E-01	0.2493	0.2169	4.95E-01	-0.1615	0.2346	2.34E-05	-0.8485	0.1842
14:41473611	rs1669594	A	G	0.76	2.52E-01	0.2493	0.2169	4.95E-01	-0.1615	0.2346	2.35E-05	-0.8484	0.1842
14:41473650	rs1779604	T	C	0.76	2.52E-01	0.2494	0.2169	4.95E-01	-0.1615	0.2346	2.35E-05	-0.8484	0.1842
14:41473651	rs1669593	A	C	0.76	2.52E-01	0.2494	0.2169	4.95E-01	-0.1615	0.2346	2.35E-05	-0.8484	0.1842
14:41473693	rs1779605	T	G	0.76	2.53E-01	0.2490	0.2170	4.95E-01	-0.1613	0.2346	2.34E-05	-0.8487	0.1842
14:41473721	rs1669592	G	A	0.76	2.52E-01	0.2495	0.2169	4.95E-01	-0.1616	0.2346	2.37E-05	-0.8479	0.1842
14:41474258	rs1779606	T	C	0.76	2.52E-01	0.2496	0.2168	4.94E-01	-0.1616	0.2345	2.37E-05	-0.8478	0.1842
14:41474317	rs7154583	C	T	0.76	2.49E-01	0.2511	0.2170	4.96E-01	-0.1610	0.2347	2.33E-05	-0.8493	0.1843
14:41474347	rs2600596	T	C	0.76	2.52E-01	0.2496	0.2168	4.94E-01	-0.1616	0.2345	2.37E-05	-0.8478	0.1842
14:41474634	rs35352678	G	C	0.76	2.51E-01	0.2496	0.2168	4.94E-01	-0.1617	0.2345	2.37E-05	-0.8477	0.1841
14:41474639	rs35874866	R	D	0.76	2.51E-01	0.2496	0.2168	4.94E-01	-0.1617	0.2345	2.37E-05	-0.8477	0.1841
14:41474682	rs2799812	G	A	0.77	2.46E-01	0.2563	0.2199	4.80E-01	-0.1693	0.2376	2.56E-05	-0.8568	0.1870
14:41474806	rs2600595	A	G	0.76	2.51E-01	0.2496	0.2168	4.94E-01	-0.1617	0.2345	2.37E-05	-0.8476	0.1841
14:41474974	rs1669590	G	C	0.76	2.52E-01	0.2496	0.2168	4.94E-01	-0.1617	0.2345	2.36E-05	-0.8480	0.1841
14:41475075	rs1669589	G	A	0.76	2.51E-01	0.2497	0.2168	4.94E-01	-0.1617	0.2345	2.38E-05	-0.8476	0.1841
14:41475103	rs562407310	R	I	0.77	2.32E-01	0.2674	0.2228	4.82E-01	-0.1712	0.2422	2.88E-05	-0.8643	0.1900
14:41475315	rs1669588	C	T	0.76	2.51E-01	0.2497	0.2168	4.94E-01	-0.1617	0.2345	2.38E-05	-0.8475	0.1841

14:41475513	rs113357272	R	I	0.76	2.51E-01	0.2497	0.2168	4.94E-01	-0.1617	0.2345	2.38E-05	-0.8475	0.1841
14:41475683	rs1612496	G	A	0.76	2.52E-01	0.2493	0.2168	4.94E-01	-0.1616	0.2345	2.37E-05	-0.8477	0.1841
14:41475734	rs1613228	G	C	0.76	2.51E-01	0.2499	0.2168	4.95E-01	-0.1615	0.2345	2.38E-05	-0.8476	0.1842
14:41475756	rs3031358	R	I	0.83	6.36E-02	-0.5200	0.2766	7.77E-01	-0.1004	0.3585	1.91E-05	-0.8419	0.1805
14:41476239	rs1779609	G	A	0.76	2.51E-01	0.2498	0.2168	4.94E-01	-0.1618	0.2345	2.38E-05	-0.8473	0.1841
14:41476282	rs1669585	A	C	0.76	2.51E-01	0.2498	0.2167	4.94E-01	-0.1618	0.2345	2.38E-05	-0.8473	0.1841
14:41476418	rs1779610	G	C	0.76	2.51E-01	0.2498	0.2167	4.94E-01	-0.1618	0.2345	2.38E-05	-0.8472	0.1841
14:41476952	rs1619176	A	G	0.76	2.51E-01	0.2499	0.2167	4.94E-01	-0.1618	0.2345	2.39E-05	-0.8471	0.1841
14:41477083	rs143210465	R	I	0.76	2.51E-01	0.2499	0.2168	4.94E-01	-0.1619	0.2345	2.39E-05	-0.8472	0.1841
14:41477359	rs1779611	T	A	0.77	1.73E-01	0.2912	0.2122	4.81E-01	-0.1666	0.2342	1.91E-05	-0.8416	0.1804
14:41477679	rs1612454	A	T	0.76	2.51E-01	0.2499	0.2167	4.94E-01	-0.1619	0.2345	2.39E-05	-0.8469	0.1841
14:41477727	rs1612328	C	T	0.76	2.51E-01	0.2499	0.2167	4.94E-01	-0.1619	0.2345	2.39E-05	-0.8469	0.1841
14:41477769	rs1669584	T	C	0.76	2.51E-01	0.2499	0.2167	4.94E-01	-0.1619	0.2344	2.39E-05	-0.8469	0.1841
14:41477781	rs149908111	R	I	0.76	2.51E-01	0.2499	0.2167	4.94E-01	-0.1619	0.2344	2.39E-05	-0.8468	0.1841
14:41478071	rs1779612	G	A	0.76	2.50E-01	0.2500	0.2167	4.94E-01	-0.1619	0.2344	2.39E-05	-0.8468	0.1841
14:41478129	rs1779614	G	A	0.76	2.50E-01	0.2500	0.2167	4.94E-01	-0.1619	0.2344	2.39E-05	-0.8468	0.1841
14:41478461	rs1669583	A	G	0.76	2.51E-01	0.2498	0.2167	4.94E-01	-0.1619	0.2345	2.40E-05	-0.8468	0.1841
14:41478666	rs1669582	A	G	0.76	2.50E-01	0.2500	0.2166	4.93E-01	-0.1619	0.2344	2.40E-05	-0.8466	0.1840
14:41478818	rs1779615	A	G	0.76	2.50E-01	0.2499	0.2167	4.93E-01	-0.1619	0.2344	2.39E-05	-0.8468	0.1840
14:41478952	rs1669581	G	A	0.77	1.72E-01	0.2917	0.2122	4.80E-01	-0.1671	0.2343	1.90E-05	-0.8422	0.1805
14:41479417	rs1376837	T	C	0.76	2.52E-01	0.2494	0.2167	4.94E-01	-0.1619	0.2345	2.39E-05	-0.8471	0.1841
14:41479846	rs11434618	R	I	0.76	2.53E-01	0.2496	0.2174	4.91E-01	-0.1630	0.2349	2.34E-05	-0.8504	0.1846
14:41479877	rs11280902	R	I	0.76	2.52E-01	0.2491	0.2168	4.94E-01	-0.1619	0.2345	2.38E-05	-0.8474	0.1841
14:41480093	rs2600594	A	G	0.76	2.53E-01	0.2490	0.2168	4.93E-01	-0.1620	0.2345	2.38E-05	-0.8475	0.1842
14:41480894	rs2799815	T	A	0.76	2.53E-01	0.2488	0.2168	4.94E-01	-0.1619	0.2345	2.37E-05	-0.8477	0.1842
14:41482769	rs2799816	C	T	0.76	2.54E-01	0.2483	0.2169	4.94E-01	-0.1619	0.2346	2.36E-05	-0.8482	0.1842
14:41482885	rs2799817	C	T	0.76	2.54E-01	0.2482	0.2169	4.94E-01	-0.1619	0.2346	2.36E-05	-0.8483	0.1842
14:41483437	rs2600593	C	T	0.76	2.54E-01	0.2489	0.2172	4.93E-01	-0.1624	0.2347	2.31E-05	-0.8502	0.1844
14:41483459	rs2600592	G	A	0.76	2.54E-01	0.2488	0.2172	4.93E-01	-0.1624	0.2347	2.31E-05	-0.8501	0.1844
14:41483719	rs8010651	G	T	0.76	2.55E-01	0.2478	0.2171	4.94E-01	-0.1620	0.2347	2.35E-05	-0.8487	0.1843
14:41483859	rs8010564	C	T	0.76	2.58E-01	0.2466	0.2172	4.94E-01	-0.1618	0.2347	2.32E-05	-0.8496	0.1843
14:41484094	rs8014861	A	G	0.76	2.56E-01	0.2476	0.2171	4.93E-01	-0.1621	0.2347	2.34E-05	-0.8490	0.1843
14:41484355	rs113750765	R	D	0.76	2.56E-01	0.2474	0.2171	4.93E-01	-0.1621	0.2347	2.34E-05	-0.8491	0.1843
14:41484459	rs12323538	T	C	0.76	2.56E-01	0.2475	0.2171	4.94E-01	-0.1619	0.2347	2.35E-05	-0.8490	0.1843

14:41484543	rs12323835	G	C	0.76	2.56E-01	0.2474	0.2171	4.94E-01	-0.1620	0.2347	2.34E-05	-0.8492	0.1843
14:41484584	rs12323833	C	T	0.76	2.56E-01	0.2473	0.2171	4.93E-01	-0.1621	0.2347	2.33E-05	-0.8493	0.1843
14:41484729	rs12323842	G	A	0.76	2.57E-01	0.2471	0.2171	4.93E-01	-0.1621	0.2347	2.33E-05	-0.8494	0.1843
14:41484813	rs12323548	T	C	0.76	2.57E-01	0.2471	0.2172	4.93E-01	-0.1621	0.2347	2.33E-05	-0.8495	0.1843
14:41485240	rs112123716	R	D	0.76	2.28E-01	0.2618	0.2162	5.27E-01	-0.1498	0.2351	2.47E-05	-0.8445	0.1839
14:41485251	rs72673799	A	T	0.76	2.58E-01	0.2467	0.2172	4.93E-01	-0.1622	0.2347	2.32E-05	-0.8497	0.1843
14:41485440	rs2799791	A	T	0.76	2.58E-01	0.2466	0.2172	4.93E-01	-0.1621	0.2348	2.32E-05	-0.8499	0.1844
14:41485668	rs2799792	A	T	0.76	2.59E-01	0.2463	0.2173	4.94E-01	-0.1621	0.2348	2.32E-05	-0.8501	0.1844
14:41485805	rs2600575	C	T	0.77	1.78E-01	0.2882	0.2126	4.82E-01	-0.1666	0.2345	1.86E-05	-0.8440	0.1806
14:41485862	rs2600576	C	T	0.77	1.79E-01	0.2878	0.2128	4.81E-01	-0.1667	0.2346	1.85E-05	-0.8448	0.1807
14:41486126	rs2799793	A	G	0.76	2.59E-01	0.2460	0.2173	4.94E-01	-0.1621	0.2348	2.31E-05	-0.8503	0.1844
14:41486622	rs2600578	G	A	0.76	2.60E-01	0.2459	0.2174	4.94E-01	-0.1621	0.2348	2.31E-05	-0.8505	0.1844
14:41486893	rs1947332	T	C	0.76	2.59E-01	0.2461	0.2174	4.93E-01	-0.1625	0.2349	2.26E-05	-0.8516	0.1845
14:41486917	rs1947333	G	A	0.76	2.60E-01	0.2457	0.2174	4.94E-01	-0.1622	0.2349	2.28E-05	-0.8512	0.1845
14:41487070	rs1349567	T	C	0.76	2.61E-01	0.2455	0.2174	4.94E-01	-0.1622	0.2349	2.30E-05	-0.8507	0.1845
14:41487623	rs1901471	C	G	0.77	1.49E-01	0.3103	0.2133	4.71E-01	-0.1712	0.2349	2.12E-05	-0.8440	0.1821
14:41487644	rs34259503	R	D	0.77	1.49E-01	0.3103	0.2133	4.71E-01	-0.1712	0.2349	2.11E-05	-0.8440	0.1821
14:41488833	rs2799796	C	G	0.76	2.21E-01	0.2683	0.2181	4.83E-01	-0.1666	0.2352	2.62E-05	-0.8502	0.1859
14:41489568	rs1376822	T	C	0.76	2.22E-01	0.2677	0.2182	4.83E-01	-0.1665	0.2353	2.61E-05	-0.8506	0.1859
14:41489743	rs1597738	G	T	0.76	2.22E-01	0.2677	0.2182	4.83E-01	-0.1665	0.2353	2.61E-05	-0.8507	0.1859
14:41489905	rs1597739	C	T	0.76	2.22E-01	0.2676	0.2182	4.83E-01	-0.1665	0.2353	2.61E-05	-0.8507	0.1859
14:41490613	rs2600586	G	C	0.76	2.23E-01	0.2671	0.2183	4.83E-01	-0.1665	0.2353	2.60E-05	-0.8510	0.1859
14:41491046	rs111722085	R	I	0.76	2.24E-01	0.2668	0.2186	4.84E-01	-0.1665	0.2356	2.62E-05	-0.8519	0.1862
14:41491121	rs1584703	A	C	0.77	1.52E-01	0.3085	0.2137	4.71E-01	-0.1713	0.2351	2.07E-05	-0.8459	0.1822
14:41492296	rs2799799	C	T	0.76	2.25E-01	0.2661	0.2184	4.83E-01	-0.1667	0.2354	2.58E-05	-0.8520	0.1860
14:41492374	rs2600587	T	A	0.76	2.25E-01	0.2661	0.2185	4.83E-01	-0.1666	0.2354	2.57E-05	-0.8521	0.1860
14:41492813	rs1669580	C	T	0.76	2.26E-01	0.2658	0.2185	4.83E-01	-0.1667	0.2354	2.57E-05	-0.8524	0.1861
14:41493007	rs58448393	R	D	0.77	1.53E-01	0.3074	0.2138	4.71E-01	-0.1714	0.2352	2.05E-05	-0.8469	0.1823
14:41493317	rs1779588	G	T	0.76	2.27E-01	0.2655	0.2185	4.83E-01	-0.1667	0.2354	2.56E-05	-0.8526	0.1861
14:41493592	rs67061054	C	G	0.76	2.27E-01	0.2653	0.2186	4.83E-01	-0.1666	0.2355	2.56E-05	-0.8527	0.1861
14:41493678	rs147500813	R	I	0.76	2.70E-01	0.2415	0.2182	4.93E-01	-0.1627	0.2353	2.23E-05	-0.8548	0.1850
14:41494257	rs9323038	A	T	0.76	2.27E-01	0.2651	0.2186	4.83E-01	-0.1667	0.2355	2.55E-05	-0.8530	0.1861
14:41494512	rs1954472	A	G	0.76	2.28E-01	0.2650	0.2186	4.83E-01	-0.1667	0.2355	2.54E-05	-0.8531	0.1861
14:41494518	rs1954473	A	G	0.76	2.28E-01	0.2650	0.2186	4.83E-01	-0.1667	0.2355	2.54E-05	-0.8531	0.1861

14:41494633	rs1954474	G	C	0.76	2.28E-01	0.2649	0.2187	4.83E-01	-0.1667	0.2355	2.54E-05	-0.8532	0.1862
14:41494778	rs2415654	A	G	0.77	1.86E-01	0.2836	0.2134	4.82E-01	-0.1668	0.2350	1.77E-05	-0.8486	0.1811
14:41495053	rs147758220	R	I	0.76	2.74E-01	0.2397	0.2183	4.96E-01	-0.1615	0.2354	2.26E-05	-0.8543	0.1851
14:41495075	rs17111340	C	T	0.76	2.71E-01	0.2408	0.2182	4.94E-01	-0.1623	0.2353	2.21E-05	-0.8550	0.1849
14:41495950	rs6572049	G	A	0.76	2.71E-01	0.2412	0.2183	4.94E-01	-0.1624	0.2354	2.19E-05	-0.8559	0.1850
14:41496113	rs75164373	R	D	0.76	2.73E-01	0.2401	0.2183	4.94E-01	-0.1623	0.2353	2.19E-05	-0.8556	0.1849
14:41496606	rs146718776	R	D	0.76	2.73E-01	0.2400	0.2183	4.94E-01	-0.1623	0.2353	2.19E-05	-0.8557	0.1850
14:41496693	rs10135980	T	A	0.76	2.73E-01	0.2400	0.2183	4.94E-01	-0.1623	0.2353	2.19E-05	-0.8557	0.1850
14:41497045	rs67038006	C	T	0.76	2.74E-01	0.2397	0.2183	4.94E-01	-0.1623	0.2354	2.18E-05	-0.8559	0.1850
14:41497387	rs72676007	A	T	0.77	1.91E-01	0.2814	0.2139	4.82E-01	-0.1669	0.2351	1.74E-05	-0.8507	0.1813
14:41498119	rs12050086	C	T	0.76	2.75E-01	0.2390	0.2185	4.94E-01	-0.1623	0.2354	2.16E-05	-0.8566	0.1850
14:41498376	rs8004304	A	G	0.76	2.76E-01	0.2389	0.2185	4.94E-01	-0.1623	0.2354	2.16E-05	-0.8568	0.1850
14:41498447	rs1954475	G	A	0.76	2.76E-01	0.2387	0.2185	4.94E-01	-0.1623	0.2354	2.16E-05	-0.8568	0.1851
14:41498655	rs8006135	T	G	0.77	1.92E-01	0.2809	0.2140	4.82E-01	-0.1668	0.2352	1.72E-05	-0.8516	0.1814
14:41498957	rs8005246	A	G	0.76	2.76E-01	0.2387	0.2185	4.94E-01	-0.1623	0.2355	2.16E-05	-0.8569	0.1851
14:41499225	rs7147383	C	T	0.76	2.76E-01	0.2386	0.2185	4.94E-01	-0.1624	0.2355	2.15E-05	-0.8571	0.1851
14:41499284	rs7147395	A	G	0.77	1.94E-01	0.2799	0.2141	4.82E-01	-0.1668	0.2352	1.73E-05	-0.8518	0.1815
14:41499733	rs1959931	G	A	0.77	1.93E-01	0.2800	0.2140	4.82E-01	-0.1668	0.2352	1.72E-05	-0.8515	0.1814
14:41501740	rs7493575	T	G	0.77	1.96E-01	0.2788	0.2142	4.82E-01	-0.1667	0.2353	1.71E-05	-0.8526	0.1815
14:41501802	rs7493594	T	A	0.77	1.97E-01	0.2783	0.2144	4.82E-01	-0.1669	0.2353	1.71E-05	-0.8530	0.1816
14:41502321	rs28593648	G	A	0.77	1.98E-01	0.2778	0.2144	4.82E-01	-0.1668	0.2353	1.70E-05	-0.8535	0.1817
14:41503268	rs555526672	A	G	0.77	1.99E-01	0.2772	0.2146	4.82E-01	-0.1669	0.2354	1.70E-05	-0.8540	0.1817
14:41503269	rs11325929	R	D	0.77	1.99E-01	0.2772	0.2146	4.82E-01	-0.1669	0.2354	1.70E-05	-0.8540	0.1817
14:41503270	rs114459456	A	G	0.77	1.99E-01	0.2771	0.2145	4.82E-01	-0.1669	0.2354	1.70E-05	-0.8539	0.1817
14:41504193	rs4494449	G	A	0.77	2.00E-01	0.2765	0.2146	4.82E-01	-0.1668	0.2354	1.69E-05	-0.8544	0.1818
14:41504619	rs1959934	T	C	0.77	2.01E-01	0.2763	0.2147	4.82E-01	-0.1668	0.2355	1.69E-05	-0.8546	0.1818
14:41505403	rs72676027	T	C	0.76	2.13E-01	0.2687	0.2147	4.76E-01	-0.1690	0.2358	4.16E-05	-0.8193	0.1844
14:41506666	rs113499595	R	D	0.77	2.04E-01	0.2747	0.2152	4.82E-01	-0.1674	0.2359	1.70E-05	-0.8561	0.1822
14:41514577	rs72676038	G	A	0.73	3.81E-01	0.1933	0.2204	2.30E-01	-0.2830	0.2347	6.30E-05	-0.8180	0.1893
14:49383170	rs34413835	C	T	0.87	6.65E-05	1.1544	0.2699	1.35E-01	-0.5920	0.3908	7.64E-02	-0.5361	0.2971
14:49384848	rs34626296	A	G	0.86	6.82E-05	1.1533	0.2701	1.33E-01	-0.5945	0.3907	7.72E-02	-0.5349	0.2973
14:58208926	rs62003380	A	C	0.92	1.02E-01	0.5840	0.3531	5.68E-01	-0.2274	0.3982	1.14E-05	-1.4425	0.2998
14:58209985	rs148516454	A	G	0.92	1.02E-01	0.5847	0.3543	5.68E-01	-0.2278	0.3995	1.12E-05	-1.4483	0.3007
14:66417960	rs71422412	C	T	0.88	1.70E-05	1.1559	0.2476	5.44E-01	-0.2051	0.3385	1.32E-01	-0.4300	0.2811

14:66421095	rs7143906	A	C	0.88	1.59E-05	1.1404	0.2433	5.21E-01	-0.2130	0.3321	1.33E-01	-0.4219	0.2765
14:71308915	rs36116033	D	R	0.94	2.17E-02	-0.9629	0.4104	9.72E-05	305.3290	253.4890	1.46E-01	0.6114	0.4150
14:82542265	rs1769392	A	G	0.84	4.89E-01	0.1800	0.2598	9.06E-01	0.0349	0.3037	8.21E-05	-0.9488	0.2237
14:93047455	rs1885747	A	G	0.81	8.37E-05	-1.0331	0.2454	5.40E-01	0.2243	0.3630	1.39E-01	0.4071	0.2714
14:94669318	rs569713795	I	R	0.68	2.46E-01	-0.2564	0.2201	5.32E-05	1.3693	0.3733	7.37E-01	0.0737	0.2188
14:94737266	rs7155943	C	A	0.72	3.25E-01	-0.2094	0.2124	5.00E-05	1.2644	0.3404	2.61E-01	0.2366	0.2083
15:101975468	rs12440721	A	G	0.83	8.84E-01	-0.0468	0.3214	5.29E-01	-0.2315	0.3621	6.21E-05	-1.1862	0.2743
15:24618467	rs569630056	R	D	0.62	9.28E-06	-0.9931	0.2053	7.65E-03	0.7731	0.2952	2.07E-01	0.3020	0.2368
15:33630839	rs8032058	A	C	0.53	7.66E-02	0.3425	0.1911	2.35E-01	-0.3197	0.2616	8.79E-05	-0.7121	0.1686
15:39273700	rs12591611	C	T	0.94	4.74E-01	0.4685	0.6535	1.14E-01	-1.2421	0.7337	7.15E-05	-2.4046	0.5614
15:39274460	rs72737120	C	A	0.94	4.73E-01	0.4683	0.6529	1.14E-01	-1.2407	0.7334	7.24E-05	-2.4010	0.5610
15:39303212	rs72737170	G	C	0.94	4.73E-01	0.4716	0.6573	1.15E-01	-1.2545	0.7424	4.69E-05	-2.4708	0.5607
15:52708679	rs7166275	G	A	0.86	2.71E-01	-0.3244	0.2940	5.72E-05	1.6893	0.4884	6.71E-02	0.5294	0.2836
15:53312876	rs1501028	A	G	0.92	9.74E-05	1.7003	0.4082	4.23E-01	-0.4846	0.5850	1.91E-01	-0.5981	0.4522
15:54209564	rs8023992	C	T	0.78	1.88E-01	0.2876	0.2174	8.91E-05	-0.8937	0.2136	3.30E-01	-0.2115	0.2153
15:54211471	rs59735059	G	C	0.78	1.96E-01	0.2801	0.2155	8.40E-05	-0.8879	0.2113	3.28E-01	-0.2103	0.2133
15:54211621	rs17198272	T	C	0.78	1.94E-01	0.2812	0.2153	8.20E-05	-0.8885	0.2112	3.28E-01	-0.2101	0.2131
15:54215302	rs72735043	C	T	0.78	1.97E-01	0.2801	0.2159	8.22E-05	-0.8889	0.2112	3.27E-01	-0.2112	0.2136
15:54219684	rs11637143	G	A	0.78	1.97E-01	0.2805	0.2160	8.19E-05	-0.8894	0.2113	3.26E-01	-0.2119	0.2137
15:54219956	rs11637199	C	T	0.78	1.97E-01	0.2805	0.2160	8.18E-05	-0.8895	0.2113	3.26E-01	-0.2119	0.2137
15:54220360	rs113228023	C	T	0.78	1.97E-01	0.2805	0.2161	8.18E-05	-0.8896	0.2113	3.26E-01	-0.2120	0.2138
15:54221154	rs72735050	G	C	0.78	1.96E-01	0.2807	0.2161	8.16E-05	-0.8899	0.2114	3.25E-01	-0.2124	0.2138
15:54221789	rs72735051	C	T	0.78	1.94E-01	0.2819	0.2159	8.04E-05	-0.8906	0.2114	3.25E-01	-0.2121	0.2137
15:54222727	rs80198808	C	T	0.78	1.94E-01	0.2820	0.2160	8.03E-05	-0.8907	0.2114	3.25E-01	-0.2122	0.2137
15:54223628	rs61046113	A	T	0.78	1.97E-01	0.2806	0.2161	8.17E-05	-0.8897	0.2113	3.25E-01	-0.2122	0.2138
15:54224420	rs111281496	C	T	0.78	2.00E-01	0.2778	0.2157	7.77E-05	-0.8917	0.2112	3.29E-01	-0.2101	0.2134
15:54225923	rs72735057	G	A	0.78	1.94E-01	0.2827	0.2164	7.82E-05	-0.8928	0.2115	3.20E-01	-0.2150	0.2141
15:54228849	rs57381713	G	C	0.78	1.92E-01	0.2840	0.2162	7.80E-05	-0.8936	0.2118	3.24E-01	-0.2130	0.2140
15:54232009	rs36066540	R	D	0.70	4.16E-01	-0.1594	0.1959	7.80E-05	-0.9019	0.2137	3.30E-01	-0.2107	0.2146
15:54232103	rs1841936	T	A	0.78	1.93E-01	0.2837	0.2168	7.72E-05	-0.8950	0.2119	3.23E-01	-0.2140	0.2145
15:54236282	rs146921946	R	D	0.78	1.92E-01	0.2849	0.2173	7.47E-05	-0.8980	0.2122	3.21E-01	-0.2150	0.2150
15:54240735	rs113015617	C	T	0.78	1.90E-01	0.2866	0.2176	7.29E-05	-0.9001	0.2124	3.20E-01	-0.2159	0.2153
15:54242483	rs1107319	T	C	0.78	1.91E-01	0.2863	0.2179	6.96E-05	-0.9033	0.2126	3.17E-01	-0.2177	0.2156
15:54245152	rs752475	A	G	0.78	1.95E-01	0.2845	0.2184	6.21E-05	-0.9103	0.2128	3.08E-01	-0.2220	0.2159

15:54248526	rs72736938	A	G	0.78	2.06E-01	0.2784	0.2188	5.43E-05	-0.9190	0.2131	2.96E-01	-0.2282	0.2161
15:56329824	rs1396744	C	T	0.64	6.97E-03	0.4729	0.1700	7.43E-05	-0.7261	0.1763	2.47E-01	-0.2062	0.1762
15:56329856	rs1509401	C	T	0.64	6.97E-03	0.4729	0.1700	7.43E-05	-0.7261	0.1763	2.47E-01	-0.2062	0.1762
15:56329919	rs1509402	G	A	0.64	7.07E-03	0.4734	0.1705	7.57E-05	-0.7269	0.1766	2.48E-01	-0.2063	0.1766
15:66547227	rs35479908	R	D	0.68	9.61E-02	0.3861	0.2296	1.00E-01	-0.4644	0.2800	7.99E-05	-0.8571	0.2016
15:66547641	rs1871006	G	A	0.68	9.54E-02	0.3868	0.2296	1.00E-01	-0.4638	0.2800	7.92E-05	-0.8575	0.2016
15:66551258	rs1020927	G	C	0.68	9.51E-02	0.3869	0.2294	1.06E-01	-0.4569	0.2805	7.94E-05	-0.8567	0.2015
15:66553425	rs11630157	C	T	0.68	9.48E-02	0.3869	0.2293	1.12E-01	-0.4505	0.2807	7.99E-05	-0.8557	0.2013
15:66558655	rs11637318	C	G	0.68	9.66E-02	0.3838	0.2286	1.01E-01	-0.4598	0.2782	8.26E-05	-0.8518	0.2008
15:66559261	rs11638535	A	C	0.68	9.72E-02	0.3830	0.2286	9.92E-02	-0.4622	0.2778	8.25E-05	-0.8516	0.2008
15:66560071	rs11632542	G	A	0.68	9.72E-02	0.3827	0.2284	9.80E-02	-0.4629	0.2772	8.36E-05	-0.8502	0.2007
15:66564406	rs11633568	T	C	0.68	1.00E-01	0.3777	0.2273	8.64E-02	-0.4724	0.2728	8.97E-05	-0.8427	0.1999
15:66564857	rs141499259	R	I	0.67	6.94E-02	0.4018	0.2185	5.61E-02	-0.4960	0.2576	7.50E-05	-0.8221	0.1926
15:66565120	rs34308029	R	I	0.66	7.68E-02	0.3967	0.2215	7.43E-02	-0.4675	0.2602	6.93E-05	-0.8355	0.1946
15:66565562	rs11629698	C	T	0.67	7.49E-02	0.3900	0.2164	5.61E-02	-0.4895	0.2542	9.32E-05	-0.8038	0.1911
15:66566842	rs113727994	A	G	0.67	7.55E-02	0.3889	0.2161	5.49E-02	-0.4905	0.2533	9.45E-05	-0.8023	0.1910
15:66567630	rs111401726	R	D	0.67	7.57E-02	0.3884	0.2160	5.42E-02	-0.4912	0.2529	9.53E-05	-0.8015	0.1909
15:66568111	rs11633808	C	T	0.67	7.57E-02	0.3884	0.2160	5.39E-02	-0.4915	0.2527	9.43E-05	-0.8019	0.1909
15:66568700	rs11635096	G	A	0.67	7.58E-02	0.3882	0.2160	5.29E-02	-0.4936	0.2526	9.62E-05	-0.8009	0.1909
15:66569207	rs11635253	C	T	0.67	7.62E-02	0.3876	0.2160	5.40E-02	-0.4913	0.2527	9.63E-05	-0.8009	0.1909
15:66569347	rs11630558	T	G	0.67	7.59E-02	0.3878	0.2159	5.40E-02	-0.4911	0.2526	9.61E-05	-0.8006	0.1908
15:66571246	rs11635867	T	C	0.67	7.56E-02	0.3881	0.2159	5.55E-02	-0.4888	0.2531	9.55E-05	-0.8008	0.1908
15:66571968	rs12908529	T	G	0.67	7.56E-02	0.3882	0.2159	5.61E-02	-0.4879	0.2532	9.52E-05	-0.8009	0.1907
15:66573234	rs12913465	C	A	0.67	7.47E-02	0.3911	0.2167	5.37E-02	-0.4953	0.2542	8.33E-05	-0.8101	0.1911
15:66575550	rs9920422	T	A	0.67	7.37E-02	0.3944	0.2178	5.11E-02	-0.5040	0.2555	7.12E-05	-0.8209	0.1916
15:66575781	rs11637450	C	T	0.67	7.36E-02	0.3946	0.2179	5.09E-02	-0.5046	0.2556	6.99E-05	-0.8221	0.1916
15:66575972	rs34807609	R	D	0.67	7.36E-02	0.3948	0.2180	5.07E-02	-0.5053	0.2557	6.91E-05	-0.8229	0.1917
15:66577043	rs17198181	T	C	0.67	7.72E-02	0.3980	0.2225	4.83E-02	-0.5239	0.2626	7.32E-05	-0.8369	0.1957
15:66582298	rs8038295	G	T	0.67	7.14E-02	0.4030	0.2207	4.35E-02	-0.5303	0.2588	4.35E-05	-0.8533	0.1927
15:66583433	rs13329434	T	C	0.67	7.10E-02	0.4046	0.2213	4.25E-02	-0.5344	0.2594	4.10E-05	-0.8578	0.1930
15:80257355	rs6495460	T	C	0.87	7.67E-05	-1.1129	0.2627	1.14E-01	0.6575	0.4265	1.98E-01	0.3806	0.2924
15:85720485	rs2342130	T	C	0.69	7.66E-05	0.8350	0.1971	3.79E-01	-0.2584	0.2876	7.78E-01	0.0630	0.2224
15:85720488	rs2342129	C	G	0.69	7.66E-05	0.8350	0.1971	3.79E-01	-0.2584	0.2876	7.78E-01	0.0630	0.2224
15:88457639	rs7182329	G	A	0.70	6.43E-05	0.9530	0.2223	4.53E-02	-0.6680	0.3430	6.38E-01	0.1187	0.2513

15:88457668	rs12595804	C	T	0.68	6.39E-05	0.9203	0.2146	7.34E-02	-0.5724	0.3250	7.00E-01	-0.0940	0.2427
15:88458188	rs7168017	T	C	0.68	5.53E-05	0.9241	0.2134	8.30E-02	-0.5528	0.3245	7.12E-01	-0.0898	0.2420
15:88462348	rs11402002	I	R	0.38	7.08E-05	-0.7763	0.1823	6.25E-02	0.5323	0.2879	7.07E-01	0.0779	0.2058
15:88463831	rs11855377	T	C	0.67	5.44E-05	0.9108	0.2101	5.69E-02	-0.6388	0.3457	7.53E-01	0.0754	0.2384
15:88464110	rs1465747	C	T	0.67	7.11E-05	0.9136	0.2146	5.89E-02	-0.6387	0.3492	7.25E-01	0.0858	0.2423
15:88464729	rs1435398	C	T	0.33	5.68E-05	-0.8848	0.2046	6.10E-02	0.6153	0.3374	7.75E-01	-0.0668	0.2321
15:88465787	rs6496456	T	G	0.67	6.57E-05	0.8977	0.2097	6.12E-02	-0.6227	0.3428	7.43E-01	0.0781	0.2372
15:88465968	rs1007533	C	T	0.34	6.28E-05	-0.8807	0.2051	6.08E-02	0.6191	0.3388	6.95E-01	-0.0914	0.2320
15:88466105	rs1007534	C	T	0.34	6.29E-05	-0.8805	0.2051	6.08E-02	0.6192	0.3388	6.95E-01	-0.0916	0.2320
15:97225761	rs4255753	C	T	0.81	8.71E-05	-0.8524	0.2030	3.67E-01	0.3009	0.3390	4.99E-01	0.1552	0.2279
15:97226109	rs4965123	C	T	0.81	8.65E-05	-0.8478	0.2018	3.65E-01	0.2998	0.3369	5.09E-01	0.1506	0.2266
15:97226225	rs34290938	T	C	0.81	8.68E-05	-0.8474	0.2018	3.65E-01	0.2997	0.3367	5.09E-01	0.1505	0.2265
15:97226327	rs34085497	A	G	0.81	8.66E-05	-0.8476	0.2018	3.65E-01	0.2997	0.3366	5.09E-01	0.1504	0.2265
15:97226562	rs4453438	T	C	0.81	8.70E-05	-0.8471	0.2017	3.65E-01	0.2997	0.3366	5.08E-01	0.1507	0.2265
15:97227549	rs4608300	G	A	0.81	9.07E-05	-0.8454	0.2019	3.65E-01	0.2994	0.3364	5.10E-01	0.1503	0.2265
15:97227644	rs4246284	T	C	0.81	8.52E-05	-0.8487	0.2018	3.66E-01	0.2992	0.3367	5.07E-01	0.1513	0.2266
15:97229189	rs5814751	D	R	0.81	8.60E-05	-0.8486	0.2019	3.66E-01	0.2993	0.3368	5.09E-01	0.1507	0.2267
15:97229324	rs4447389	G	A	0.81	8.49E-05	-0.8493	0.2019	3.67E-01	0.2988	0.3368	5.08E-01	0.1511	0.2268
15:97229809	rs4493012	A	G	0.81	8.45E-05	-0.8500	0.2020	3.67E-01	0.2989	0.3369	5.08E-01	0.1512	0.2269
16:10252909	rs8050482	T	C	0.59	1.94E-01	-0.2526	0.1933	7.34E-01	0.0810	0.2398	9.82E-05	0.7066	0.1686
16:10256135	rs4587976	G	C	0.57	1.63E-01	-0.2731	0.1944	7.67E-01	0.0712	0.2388	8.75E-05	0.7170	0.1698
16:10264737	rs34463380	R	D	0.59	3.08E-01	0.2153	0.2107	6.05E-01	-0.1340	0.2628	6.75E-05	-0.7812	0.1817
16:2331430	rs149532	G	A	0.89	3.48E-01	0.3072	0.3267	5.54E-01	-0.2070	0.3499	7.54E-05	-1.2025	0.2818
16:2340603	rs161424	C	G	0.89	3.62E-01	0.3022	0.3306	5.61E-01	-0.2070	0.3560	6.88E-05	-1.2223	0.2846
16:2348820	rs11647731	G	C	0.89	4.37E-01	0.2642	0.3399	7.84E-01	-0.1019	0.3733	2.59E-05	-1.3154	0.2873
16:2349206	rs114077531	C	T	0.88	4.93E-01	0.2297	0.3350	7.43E-01	-0.1191	0.3650	7.42E-05	-1.2299	0.2879
16:2355828	rs73500183	G	A	0.89	5.69E-01	0.1912	0.3360	9.30E-01	-0.0328	0.3769	2.87E-05	-1.2908	0.2838
16:4106786	rs2239314	G	A	0.77	2.98E-05	0.9205	0.2041	9.89E-01	0.0043	0.3025	9.86E-01	0.0042	0.2342
16:4116831	rs2041245	T	A	0.77	1.69E-05	0.9294	0.1990	8.52E-01	-0.0572	0.2952	9.63E-01	-0.0106	0.2305
16:4124108	rs2601812	G	T	0.77	2.03E-05	0.9139	0.1979	7.31E-01	-0.1040	0.2911	9.72E-01	-0.0081	0.2285
16:58246346	rs16959814	T	C	0.84	9.33E-05	0.8315	0.1990	4.10E-01	-0.2360	0.2782	3.63E-01	-0.2041	0.2224
16:58246889	rs4608323	A	G	0.84	9.88E-05	0.8260	0.1985	4.01E-01	-0.2398	0.2768	3.42E-01	-0.2120	0.2214
16:58261035	rs734893	C	T	0.88	8.42E-05	1.0633	0.2526	9.64E-01	-0.0180	0.3961	3.52E-01	-0.2656	0.2827
16:58262486	rs201058553	R	I	0.83	4.10E-05	1.0248	0.2319	4.04E-02	-0.6185	0.2842	4.72E-01	-0.1908	0.2635

16:77959909	rs2435079	T	C	0.71	2.85E-05	-1.1887	0.2629	8.78E-01	0.0609	0.3886	1.46E-01	0.4369	0.2962
16:84859107	rs12934143	G	A	0.40	2.68E-02	0.4817	0.2132	4.28E-05	-1.1742	0.3000	4.15E-01	-0.1787	0.2177
16:86617955	rs4843401	T	G	0.57	6.45E-01	0.1189	0.2583	3.52E-05	-1.2600	0.3086	3.75E-01	-0.2257	0.2526
16:86688819	rs300038	T	C	0.74	2.64E-02	-0.4799	0.2119	1.21E-02	0.7120	0.3025	3.04E-05	0.8459	0.1866
16:86689335	rs4843418	G	C	0.72	7.31E-03	-0.5179	0.1873	6.81E-03	0.7236	0.2875	8.34E-05	0.7255	0.1712
16:87951377	rs548123416	T	A	0.89	9.66E-01	0.0141	0.3317	2.97E-01	0.4624	0.4536	7.77E-05	1.2099	0.2841
16:87951378	rs566667449	G	A	0.89	9.67E-01	0.0138	0.3317	2.97E-01	0.4626	0.4537	7.77E-05	1.2099	0.2841
16:8908811	rs8057024	A	G	0.43	1.76E-01	0.3289	0.2416	2.73E-06	-1.4127	0.2959	7.49E-01	0.0774	0.2412
17:12800766	rs78520683	T	C	0.87	9.70E-01	-0.0116	0.3145	2.47E-01	0.4370	0.3873	2.67E-05	1.2087	0.2645
17:12815191	rs77337685	T	C	0.91	6.21E-01	-0.1846	0.3733	2.19E-01	0.5470	0.4533	4.13E-05	1.4087	0.3170
17:12819502	rs7225504	T	C	0.91	6.28E-01	-0.1810	0.3734	2.19E-01	0.5469	0.4531	4.36E-05	1.4054	0.3174
17:13388145	rs55863028	C	T	0.54	1.62E-05	-0.7862	0.1679	5.46E-01	0.1507	0.2548	6.56E-01	0.0871	0.1943
17:13388937	rs2190821	T	C	0.57	7.05E-05	-0.7457	0.1750	6.20E-01	0.1242	0.2556	8.14E-01	0.0467	0.1978
17:13390118	rs5819416	R	D	0.54	1.49E-05	-0.7857	0.1670	5.06E-01	0.1652	0.2545	6.52E-01	0.0877	0.1935
17:13391511	rs7213995	A	C	0.54	6.70E-06	0.7977	0.1619	1.61E-01	-0.3280	0.2394	7.23E-01	-0.0678	0.1902
17:13392543	rs7220676	T	C	0.57	5.24E-05	-0.7564	0.1740	5.90E-01	0.1353	0.2563	8.06E-01	0.0487	0.1977
17:13393244	rs8081141	T	G	0.54	4.37E-06	0.8148	0.1615	1.81E-01	-0.3126	0.2388	6.76E-01	-0.0802	0.1911
17:13393882	rs7209938	G	A	0.54	4.35E-06	0.8148	0.1614	1.80E-01	-0.3139	0.2389	6.81E-01	-0.0790	0.1911
17:13395795	rs9675262	C	T	0.54	1.27E-05	-0.7857	0.1654	4.95E-01	0.1694	0.2531	6.74E-01	0.0814	0.1922
17:13397297	rs4792364	C	G	0.57	1.41E-05	0.7922	0.1678	1.73E-01	-0.3281	0.2489	9.64E-01	-0.0088	0.1950
17:13399486	rs67951062	R	I	0.61	6.46E-05	-0.8370	0.1953	8.60E-01	0.0517	0.3001	4.45E-01	-0.1913	0.2489
17:14579953	rs76207825	A	G	0.95	8.58E-05	1.7604	0.4189	4.77E-01	-0.3683	0.5100	5.52E-01	-0.2813	0.4706
17:33512756	rs6505449	T	A	0.66	4.45E-01	-0.1510	0.1975	5.57E-02	0.4067	0.2169	5.76E-05	0.7353	0.1692
17:39133514	rs34386311	R	I	0.92	3.90E-05	-1.5209	0.3431	1.18E-01	0.9039	0.6135	2.91E-01	0.4137	0.3881
17:4498641	rs12939239	A	G	0.86	3.46E-01	0.2743	0.2905	6.89E-05	-1.0823	0.2545	2.11E-01	-0.3593	0.2839
17:4499121	rs2035262	G	A	0.84	5.35E-01	0.1598	0.2580	3.06E-05	-1.0486	0.2349	3.33E-01	-0.2466	0.2524
17:4500072	rs9906333	G	A	0.84	5.33E-01	0.1613	0.2591	3.18E-05	-1.0541	0.2368	3.37E-01	-0.2456	0.2535
17:4502083	rs4790656	C	T	0.84	5.54E-01	0.1588	0.2687	5.70E-05	-1.0739	0.2495	3.81E-01	-0.2322	0.2632
17:53537811	rs11397688	R	I	0.23	4.70E-03	-0.6398	0.2188	2.73E-01	0.2937	0.2654	3.87E-05	0.8871	0.1988
17:66433134	rs139267277	R	D	0.63	9.21E-05	0.7922	0.1894	2.60E-01	-0.2976	0.2553	2.02E-01	-0.2714	0.2102
18:22284668	rs339939	C	T	0.42	5.84E-05	0.7182	0.1664	3.28E-01	0.2265	0.2211	6.27E-02	-0.3476	0.1831
18:22296768	rs1668095	A	C	0.61	7.08E-05	-0.7002	0.1644	8.04E-01	-0.0618	0.2373	1.15E-01	0.2913	0.1818
18:22304787	rs2443015	A	G	0.42	9.96E-05	0.7066	0.1699	2.87E-01	0.2501	0.2241	9.93E-02	-0.3125	0.1865
18:22305096	rs1680320	C	T	0.42	9.91E-05	0.7068	0.1699	2.87E-01	0.2503	0.2241	9.94E-02	-0.3124	0.1865

18:22305197	rs10607323	D	R	0.59	6.27E-05	-0.6977	0.1625	5.17E-01	-0.1548	0.2255	6.95E-03	0.4833	0.1726
18:22305924	rs1668107	G	A	0.42	9.69E-05	0.7080	0.1699	2.92E-01	0.2482	0.2245	9.93E-02	-0.3126	0.1866
18:22306116	rs1668108	A	G	0.42	9.65E-05	0.7082	0.1699	2.93E-01	0.2478	0.2246	9.93E-02	-0.3126	0.1866
18:22306314	rs74889671	R	D	0.42	9.61E-05	0.7085	0.1699	2.93E-01	0.2474	0.2247	9.93E-02	-0.3127	0.1866
18:22306607	rs1680346	G	T	0.42	9.54E-05	0.7089	0.1699	2.95E-01	0.2467	0.2248	9.93E-02	-0.3127	0.1866
18:22306699	rs1668109	T	C	0.42	9.48E-05	0.7092	0.1699	2.96E-01	0.2462	0.2249	9.93E-02	-0.3127	0.1867
18:22306767	rs1680347	G	A	0.42	9.43E-05	0.7094	0.1699	2.97E-01	0.2458	0.2250	9.93E-02	-0.3128	0.1867
18:22307357	rs1668110	C	T	0.42	9.38E-05	0.7098	0.1699	2.97E-01	0.2459	0.2250	9.97E-02	-0.3125	0.1867
18:22307480	rs1680348	A	C	0.42	9.46E-05	0.7096	0.1700	2.96E-01	0.2462	0.2249	1.00E-01	-0.3123	0.1868
18:24286681	rs470841	T	C	0.65	7.10E-03	0.5434	0.1958	2.71E-01	-0.2661	0.2340	4.09E-05	-0.7864	0.1769
18:26585983	rs199597833	A	C	0.93	8.62E-01	-0.0939	0.5411	8.21E-05	3.9188	1.1726	8.49E-01	-0.1016	0.5320
18:26585984	rs200540108	C	T	0.93	8.62E-01	-0.0939	0.5411	8.23E-05	3.9180	1.1725	8.48E-01	-0.1025	0.5320
18:3273849	rs57640442	G	T	0.72	8.91E-05	0.8107	0.1934	9.06E-01	0.0356	0.3014	2.66E-01	-0.2420	0.2155
18:3700584	rs11662353	A	G	0.87	5.33E-01	0.2040	0.3274	9.03E-01	-0.0483	0.3969	9.81E-05	-1.1834	0.2824
18:3702197	rs55815210	G	A	0.88	5.23E-01	0.2143	0.3354	9.14E-01	-0.0439	0.4077	9.86E-05	-1.2121	0.2894
18:43083666	rs12454126	A	G	0.92	8.41E-01	0.0762	0.3813	3.63E-01	0.4328	0.4803	9.22E-05	1.3783	0.3275
18:50823502	rs10617714	R	D	0.93	4.14E-01	0.3211	0.3926	6.27E-05	1304.0700	661.7200	5.91E-01	0.2096	0.3873
18:50824089	rs12960007	A	C	0.93	4.13E-01	0.3214	0.3927	8.31E-05	1257.3100	661.6240	5.91E-01	0.2095	0.3874
18:50824183	rs12961446	T	C	0.93	4.13E-01	0.3221	0.3928	8.31E-05	1257.3100	661.6240	5.91E-01	0.2095	0.3875
18:59269343	rs33987401	R	D	0.51	5.55E-05	0.7921	0.1829	5.11E-01	-0.1772	0.2733	7.74E-01	-0.0600	0.2075
18:59269346	rs199618199	T	A	0.51	5.54E-05	0.7921	0.1829	5.12E-01	-0.1770	0.2733	7.74E-01	-0.0600	0.2075
18:60967699	rs2615195	C	G	0.85	4.32E-02	0.5893	0.2867	3.02E-01	-0.3663	0.3436	5.12E-05	-1.1077	0.2528
18:68839134	rs10460134	T	C	0.61	9.95E-05	0.6549	0.1574	7.47E-04	-0.7441	0.2206	5.39E-01	-0.1091	0.1764
18:68839205	rs72970356	A	G	0.79	1.33E-05	0.9746	0.2057	4.09E-03	-0.7607	0.2509	6.18E-01	-0.1198	0.2388
18:68839629	rs79057581	C	A	0.80	2.35E-05	1.0180	0.2224	2.84E-03	-0.8165	0.2621	6.40E-01	-0.1201	0.2557
18:68839803	rs8084484	C	T	0.61	9.10E-05	0.6530	0.1560	8.28E-04	-0.7340	0.2190	5.23E-01	-0.1126	0.1750
18:68840752	rs72970372	T	C	0.82	8.00E-05	0.9802	0.2321	3.67E-02	-0.5793	0.2658	8.51E-01	-0.0493	0.2618
18:68841687	rs561503976	T	G	0.83	7.06E-05	0.9952	0.2336	3.60E-02	-0.5852	0.2676	8.22E-01	-0.0597	0.2641
18:68841691	rs148585323	T	G	0.83	5.91E-05	1.0264	0.2380	3.24E-02	-0.6129	0.2750	9.26E-01	-0.0253	0.2700
18:68841699	rs146157448	T	G	0.83	7.68E-05	1.0091	0.2382	3.95E-02	-0.5877	0.2741	8.01E-01	-0.0682	0.2689
18:68843062	rs7243799	C	T	0.82	7.52E-05	0.9829	0.2317	3.70E-02	-0.5780	0.2657	8.47E-01	-0.0506	0.2617
18:68844408	rs9949570	A	G	0.59	9.75E-05	0.6517	0.1565	1.21E-03	-0.7165	0.2198	5.93E-01	-0.0942	0.1755
18:68845592	rs4524030	G	A	0.51	5.98E-05	-0.7510	0.1743	1.20E-03	0.7851	0.2415	4.42E-01	0.1522	0.1966
18:69018071	rs9947008	T	C	0.79	7.92E-05	0.9889	0.2340	3.90E-02	-0.5900	0.2752	5.22E-01	-0.1696	0.2631

18:69030842	rs17084304	C	T	0.79	9.56E-05	0.9797	0.2349	3.63E-02	-0.5980	0.2748	5.20E-01	-0.1704	0.2633
18:69039235	rs28470346	T	C	0.81	2.34E-05	1.0489	0.2291	1.61E-01	-0.4258	0.2999	3.16E-01	-0.2645	0.2616
18:70372800	rs9948999	C	A	0.86	4.64E-02	-0.6006	0.2969	1.07E-01	0.6371	0.4023	9.92E-05	1.1070	0.2644
18:70383839	rs55959329	A	G	0.87	7.14E-02	-0.5405	0.2960	1.12E-01	0.6185	0.3973	9.38E-05	1.1001	0.2617
18:70384025	rs55889413	A	G	0.87	7.14E-02	-0.5404	0.2961	1.12E-01	0.6185	0.3973	9.42E-05	1.1000	0.2618
18:70384421	rs4892033	T	C	0.87	7.14E-02	-0.5407	0.2962	1.12E-01	0.6184	0.3974	9.48E-05	1.1001	0.2619
18:70388163	rs57516430	C	T	0.87	9.33E-02	-0.5122	0.3020	1.19E-01	0.6113	0.4007	9.37E-05	1.1181	0.2660
18:71116340	rs7241104	C	T	0.91	5.59E-01	0.2069	0.3539	9.24E-05	2.1369	0.6183	9.07E-01	-0.0411	0.3489
18:71117075	rs74874796	T	C	0.91	5.54E-01	0.2098	0.3543	9.27E-05	2.1368	0.6184	9.03E-01	-0.0427	0.3494
18:71117231	rs76604675	C	T	0.91	5.53E-01	0.2101	0.3543	9.27E-05	2.1368	0.6184	9.03E-01	-0.0426	0.3494
18:71117387	rs113838898	A	G	0.91	5.51E-01	0.2114	0.3545	9.27E-05	2.1366	0.6183	9.02E-01	-0.0434	0.3496
18:71117660	rs77516206	A	G	0.91	5.50E-01	0.2120	0.3546	9.34E-05	2.1355	0.6182	9.02E-01	-0.0433	0.3497
18:71119741	rs17824575	T	A	0.91	5.37E-01	0.2196	0.3556	9.35E-05	2.1353	0.6181	8.96E-01	-0.0458	0.3507
18:72074817	rs4892205	T	G	0.77	1.61E-05	1.0418	0.2224	4.95E-01	-0.2384	0.3519	1.97E-01	-0.3316	0.2541
18:72076466	rs35183655	I	R	0.77	2.30E-05	1.0019	0.2186	5.09E-01	-0.2269	0.3465	1.71E-01	-0.3438	0.2478
18:72076567	rs9955537	C	T	0.77	2.42E-05	0.9787	0.2142	4.96E-01	-0.2287	0.3385	1.46E-01	-0.3572	0.2421
18:72077557	rs9319904	C	T	0.74	1.29E-05	1.0599	0.2233	9.93E-02	-0.6145	0.3691	1.99E-01	-0.3327	0.2561
19:22764872	rs74538802	G	A	0.95	3.50E-01	0.3588	0.3834	8.33E-05	-1.0216	0.2143	2.52E-01	-0.4348	0.3755
19:2818547	rs551489473	R	I	0.81	2.52E-03	-0.9355	0.2979	1.08E-01	0.6370	0.4016	8.57E-05	1.1717	0.2770
19:29122756	rs59404768	T	C	0.77	1.37E-02	-0.5910	0.2338	3.23E-01	0.2970	0.3004	5.42E-05	0.9167	0.2100
19:33538297	rs16967749	C	T	0.89	5.06E-05	-1.1857	0.2721	8.36E-02	0.8248	0.5016	9.31E-02	0.5155	0.3018
19:33538342	rs34363498	G	A	0.88	9.37E-05	-1.1515	0.2757	9.22E-02	0.7928	0.4927	1.68E-01	0.4265	0.3051
19:33540687	rs34621377	C	A	0.91	5.32E-05	-1.3054	0.3006	9.29E-03	1.5630	0.6911	1.67E-01	0.4697	0.3358
19:33544041	rs34475253	C	G	0.90	2.51E-05	-1.3458	0.2952	7.87E-03	1.6031	0.6949	1.51E-01	0.4860	0.3336
19:33545042	rs12462348	C	T	0.89	4.09E-05	-1.3106	0.2966	9.60E-03	1.5276	0.6660	2.00E-01	0.4326	0.3336
19:33545881	rs12462935	C	T	0.90	2.94E-05	-1.3230	0.2931	9.06E-03	1.5391	0.6684	2.05E-01	0.4255	0.3316
19:33547003	rs573316854	R	I	0.89	3.14E-05	-1.3036	0.2900	8.91E-03	1.5396	0.6676	2.25E-01	0.4023	0.3282
19:33548203	rs12983403	C	G	0.89	4.96E-05	-1.2808	0.2936	9.00E-03	1.5170	0.6543	2.36E-01	0.3948	0.3298
19:33552417	rs35913518	T	G	0.90	4.39E-05	-1.3043	0.2965	7.88E-03	1.5749	0.6728	2.07E-01	0.4258	0.3333
19:33556670	rs12459968	T	A	0.90	2.20E-05	-1.4490	0.3153	7.23E-03	1.6836	0.7118	1.97E-01	0.4675	0.3583
19:33559874	rs60203782	G	T	0.89	6.83E-05	-1.2464	0.2919	9.08E-03	1.4894	0.6410	2.67E-01	0.3664	0.3267
19:33562015	rs71176190	R	I	0.89	9.08E-05	-1.2333	0.2946	8.35E-03	1.4993	0.6376	3.25E-01	0.3265	0.3289
19:33562695	rs60197880	C	A	0.89	9.36E-05	-1.2176	0.2915	8.65E-03	1.4835	0.6342	3.15E-01	0.3297	0.3251
19:33562700	rs28661618	T	G	0.89	8.38E-05	-1.2169	0.2890	1.80E-01	-0.5135	0.3698	8.53E-01	-0.0615	0.3296

19:33562757	rs34154013	G	A	0.89	9.42E-05	-1.2167	0.2914	8.66E-03	1.4827	0.6339	3.16E-01	0.3290	0.3250
19:33564874	rs34266236	C	A	0.89	9.77E-05	-1.2116	0.2909	8.72E-03	1.4768	0.6317	3.20E-01	0.3254	0.3243
19:38992852	rs77022898	C	T	0.94	4.33E-01	-0.3404	0.4338	3.53E-05	6.3624	5.2101	1.55E-01	0.6073	0.4212
19:38998771	rs77696417	A	G	0.94	4.36E-01	-0.3346	0.4293	3.12E-05	6.9007	6.4646	1.54E-01	0.6016	0.4168
19:39007210	rs115308402	A	C	0.94	4.18E-01	-0.3467	0.4277	2.75E-05	5.8395	3.8946	1.36E-01	0.6271	0.4147
19:39011141	rs150685379	C	G	0.95	2.78E-01	-0.4931	0.4528	7.88E-05	13.5108	27.0109	2.67E-01	0.4991	0.4450
19:39030035	rs115404685	T	C	0.94	2.92E-01	-0.4845	0.4589	8.58E-05	10.9728	18.2850	2.51E-01	0.5224	0.4503
19:39031714	rs115755496	G	A	0.95	2.90E-01	-0.4868	0.4589	7.65E-05	16.7269	32.6600	2.48E-01	0.5252	0.4503
19:39036416	rs116072693	A	C	0.95	2.95E-01	-0.4852	0.4616	7.26E-05	18.3750	31.2175	2.40E-01	0.5374	0.4527
19:39037276	rs115019209	G	A	0.95	3.04E-01	-0.4780	0.4640	9.08E-05	9.6093	11.8363	2.35E-01	0.5462	0.4548
19:39398631	rs3136644	A	G	0.77	7.20E-07	-1.1388	0.2057	4.70E-02	0.7984	0.4240	6.08E-01	0.1293	0.2509
19:39398649	rs3136645	T	C	0.77	3.79E-06	-1.0441	0.2053	5.76E-02	0.7604	0.4231	7.08E-01	0.0918	0.2436
19:39403637	rs12462170	T	G	0.80	1.07E-05	-1.0265	0.2140	8.09E-02	0.6905	0.4172	9.21E-01	0.0250	0.2497
19:39407775	rs10415194	A	C	0.77	2.87E-05	-0.9053	0.2003	3.30E-01	0.3074	0.3201	7.56E-01	-0.0718	0.2297
19:39410986	rs12460327	C	G	0.77	1.55E-05	-0.9366	0.1995	3.96E-01	0.2665	0.3174	8.39E-01	-0.0473	0.2314
19:39411576	rs28736288	T	G	0.77	2.90E-05	-0.9037	0.2000	3.15E-01	0.3174	0.3203	8.14E-01	-0.0543	0.2295
19:39411588	rs201362509	C	G	0.76	4.87E-05	-0.8985	0.2057	3.40E-01	0.3093	0.3279	7.25E-01	-0.0828	0.2338
19:39411590	rs111212157	C	T	0.76	4.87E-05	-0.8985	0.2057	3.40E-01	0.3094	0.3279	7.26E-01	-0.0823	0.2338
19:39411610	rs200119729	G	C	0.77	3.36E-05	-0.8951	0.2000	3.30E-01	0.3054	0.3176	8.47E-01	-0.0444	0.2290
19:39412445	rs10412886	G	A	0.77	1.64E-05	-0.9336	0.1995	3.80E-01	0.2752	0.3173	8.99E-01	-0.0295	0.2312
19:39412756	rs539687857	R	I	0.79	1.38E-05	-1.0225	0.2163	1.99E-01	0.4466	0.3551	9.59E-01	-0.0130	0.2514
19:39414654	rs28375245	C	T	0.77	3.34E-05	-0.8943	0.1998	2.97E-01	0.3283	0.3185	9.13E-01	-0.0251	0.2287
19:39414771	rs112360575	R	I	0.79	4.40E-05	-0.9415	0.2141	2.23E-01	0.4325	0.3585	7.82E-01	-0.0677	0.2439
19:39416409	rs751941	G	A	0.76	2.03E-05	-0.9173	0.1986	3.58E-01	0.2860	0.3139	9.88E-01	0.0035	0.2294
19:39420194	rs11882588	C	T	0.77	5.56E-05	-0.8840	0.2042	2.75E-01	0.3603	0.3373	7.86E-01	0.0632	0.2317
19:39424421	rs10410176	G	A	0.80	2.97E-05	-0.9624	0.2134	4.30E-01	0.2476	0.3177	7.68E-01	0.0725	0.2446
19:39427860	rs2116854	C	T	0.80	2.98E-05	-0.9686	0.2148	4.38E-01	0.2455	0.3204	7.60E-01	0.0757	0.2462
19:39427886	rs2116855	T	C	0.80	2.98E-05	-0.9688	0.2148	4.39E-01	0.2451	0.3204	7.59E-01	0.0761	0.2463
19:39430902	rs10407879	A	G	0.80	3.13E-05	-0.9715	0.2161	4.47E-01	0.2420	0.3221	7.55E-01	0.0777	0.2475
19:39431374	rs111827352	R	D	0.80	3.07E-05	-0.9731	0.2162	4.53E-01	0.2384	0.3215	7.42E-01	0.0819	0.2477
19:39432136	rs1134180	G	A	0.80	3.10E-05	-0.9741	0.2165	4.55E-01	0.2376	0.3221	7.41E-01	0.0825	0.2481
19:46776156	rs12971298	G	T	0.70	4.04E-01	-0.2173	0.2600	4.70E-01	0.2281	0.3261	6.68E-06	1.0665	0.2150
19:53297017	rs377034734	D	R	0.63	5.03E-05	-1.1224	0.2575	1.17E-01	0.6794	0.4329	1.85E-01	0.3869	0.2883
1:150170113	rs144581493	G	T	0.94	5.77E-05	-1.6241	0.3760	2.66E-01	0.7560	0.7363	3.17E-02	0.9019	0.4096

1:152186042	rs12751022	A	G	0.60	3.51E-02	-0.4793	0.2234	7.46E-05	1.0302	0.2620	1.04E-01	0.3685	0.2232
1:152186178	rs4845748	C	T	0.41	3.38E-01	0.2206	0.2297	3.43E-05	-1.0249	0.2522	3.00E-01	-0.2359	0.2255
1:152301221	rs1933063	G	A	0.88	2.73E-01	0.3874	0.3525	9.31E-05	-1.3385	0.3279	9.19E-01	0.0359	0.3503
1:161269835	rs143159974	R	D	0.83	4.79E-05	1.3642	0.3120	8.64E-02	-0.7270	0.4118	3.01E-01	-0.3673	0.3517
1:171788216	N/A	R	D	0.79	5.95E-05	-0.9535	0.2212	8.14E-02	0.6010	0.3601	5.66E-01	0.1443	0.2502
1:191204765	rs71637267	C	A	0.93	2.42E-01	0.5089	0.4327	4.40E-02	-0.9575	0.4614	9.23E-05	-1.5829	0.3762
1:191241015	rs12744441	G	T	0.93	3.33E-01	0.4176	0.4300	4.73E-02	-0.9390	0.4601	7.53E-05	-1.5841	0.3711
1:201690200	rs12058357	T	A	0.83	9.43E-03	-0.6759	0.2532	9.40E-05	1.3775	0.3731	5.81E-02	0.4950	0.2559
1:201698200	rs12565521	C	G	0.83	1.07E-02	-0.6713	0.2561	9.15E-05	1.3838	0.3742	7.41E-02	0.4717	0.2593
1:201698751	rs61821710	G	T	0.83	1.07E-02	-0.6714	0.2560	9.17E-05	1.3830	0.3740	7.41E-02	0.4718	0.2592
1:201698890	rs61821711	C	T	0.83	9.61E-03	-0.6693	0.2513	8.46E-05	1.3840	0.3731	5.78E-02	0.4918	0.2540
1:201699454	rs12058481	G	A	0.83	9.53E-03	-0.6696	0.2512	8.48E-05	1.3831	0.3730	5.77E-02	0.4918	0.2539
1:209261228	rs60731826	C	T	0.65	1.33E-01	0.3611	0.2384	1.80E-02	-0.6146	0.2629	9.38E-06	-0.9781	0.2010
1:209729572	rs66672103	D	R	0.77	7.66E-01	-0.0767	0.2585	1.45E-01	0.4574	0.3312	1.63E-05	1.0164	0.2158
1:209729824	rs9429996	T	A	0.77	7.67E-01	-0.0765	0.2581	1.45E-01	0.4566	0.3309	1.60E-05	1.0157	0.2154
1:211414012	rs10863882	G	T	0.54	9.14E-05	-0.7043	0.1683	3.83E-01	0.2078	0.2368	1.03E-01	0.3069	0.1851
1:228829657	rs12028277	T	C	0.71	7.13E-05	0.8802	0.2067	8.98E-01	0.0371	0.2782	3.50E-01	-0.2185	0.2320
1:228836000	rs12728864	G	A	0.90	9.20E-05	-1.2967	0.3100	7.11E-01	0.2067	0.5628	1.87E-01	0.4593	0.3437
1:228837451	rs6426512	T	C	0.90	9.40E-05	-1.2939	0.3098	7.12E-01	0.2053	0.5619	1.88E-01	0.4577	0.3434
1:228846879	rs375374820	A	C	0.90	9.34E-05	-1.3087	0.3132	7.45E-01	0.1821	0.5627	1.92E-01	0.4586	0.3473
1:228852494	rs11270267	R	I	0.93	4.68E-01	-0.2822	0.3890	4.05E-01	-0.3279	0.3791	8.92E-01	-0.0525	0.3842
1:228854138	rs72750042	T	C	0.75	2.91E-05	0.8944	0.1980	7.10E-01	0.1086	0.2806	1.67E-01	-0.3128	0.2235
1:228860072	rs11587994	A	C	0.75	2.98E-05	0.8977	0.1991	7.10E-01	0.1090	0.2822	1.73E-01	-0.3102	0.2247
1:228862269	rs56221905	A	C	0.75	3.03E-05	0.8986	0.1995	7.11E-01	0.1090	0.2828	1.75E-01	-0.3091	0.2251
1:228865663	rs55730631	C	A	0.76	2.96E-05	0.8994	0.1993	7.32E-01	0.1005	0.2827	1.83E-01	-0.3034	0.2252
1:228867648	rs72751907	C	T	0.76	2.76E-05	0.9044	0.1996	7.72E-01	0.0852	0.2832	1.92E-01	-0.2983	0.2259
1:228868477	rs6701241	C	G	0.74	5.72E-05	0.8538	0.1976	8.07E-01	0.0695	0.2755	3.21E-01	-0.2225	0.2223
1:228868574	rs6665334	A	C	0.76	2.64E-05	0.9075	0.1997	8.03E-01	0.0736	0.2835	1.99E-01	-0.2939	0.2263
1:228870372	rs151192724	R	I	0.76	2.60E-05	0.9085	0.1997	8.18E-01	0.0678	0.2836	2.03E-01	-0.2915	0.2264
1:228870697	rs3840437	R	I	0.76	2.59E-05	0.9088	0.1998	8.21E-01	0.0668	0.2837	2.04E-01	-0.2912	0.2265
1:228871693	rs3738073	C	T	0.76	2.57E-05	0.9092	0.1997	8.31E-01	0.0631	0.2836	2.06E-01	-0.2897	0.2265
1:228872549	rs55956922	T	A	0.76	2.54E-05	0.9097	0.1997	8.41E-01	0.0592	0.2836	2.09E-01	-0.2880	0.2266
1:228872580	rs60203629	R	I	0.76	2.52E-05	0.9109	0.1999	8.45E-01	0.0577	0.2838	2.10E-01	-0.2876	0.2268
1:228878193	rs17352654	T	C	0.76	2.45E-05	0.9103	0.1994	9.51E-01	0.0180	0.2833	2.39E-01	-0.2700	0.2267

1:228878481	rs4391638	A	C	0.76	2.45E-05	0.9102	0.1994	9.58E-01	0.0155	0.2833	2.41E-01	-0.2689	0.2267
1:228878663	rs4641285	G	A	0.76	2.46E-05	0.9097	0.1993	9.61E-01	0.0143	0.2832	2.42E-01	-0.2679	0.2267
1:228878669	rs4993975	A	C	0.76	2.46E-05	0.9097	0.1993	9.62E-01	0.0140	0.2832	2.42E-01	-0.2678	0.2266
1:228878871	rs41270187	T	C	0.74	5.49E-05	0.8530	0.1969	9.75E-01	0.0090	0.2746	3.96E-01	-0.1898	0.2221
1:228878888	rs41270189	C	T	0.76	2.46E-05	0.9095	0.1993	9.67E-01	0.0121	0.2832	2.44E-01	-0.2669	0.2266
1:228880021	rs1062060	C	T	0.76	2.47E-05	0.9092	0.1992	9.75E-01	0.0091	0.2831	2.46E-01	-0.2654	0.2266
1:228880098	rs11578216	T	A	0.76	2.47E-05	0.9088	0.1992	9.74E-01	0.0097	0.2829	2.46E-01	-0.2653	0.2265
1:228880135	rs11593565	C	T	0.76	2.48E-05	0.9081	0.1991	9.71E-01	0.0108	0.2826	2.47E-01	-0.2651	0.2264
1:228880296	rs41271473	G	A	0.74	6.04E-05	0.8523	0.1979	9.65E-01	0.0125	0.2749	4.01E-01	-0.1886	0.2230
1:228880466	rs11580020	G	A	0.76	2.51E-05	0.9054	0.1986	9.59E-01	0.0149	0.2813	2.47E-01	-0.2642	0.2259
1:228884358	rs72751929	A	G	0.75	3.02E-05	0.8762	0.1945	8.65E-01	0.0482	0.2693	2.52E-01	-0.2551	0.2205
1:228887129	rs72751931	G	T	0.71	3.74E-07	0.9796	0.1716	6.43E-01	-0.1218	0.2518	4.32E-01	-0.1669	0.2109
1:228889664	rs17352829	A	G	0.71	4.67E-07	0.9677	0.1713	6.55E-01	-0.1165	0.2504	4.34E-01	-0.1653	0.2097
1:228902278	rs34575653	R	D	0.52	5.39E-05	0.7999	0.1844	5.62E-01	-0.1442	0.2447	2.63E-01	-0.2343	0.2071
1:228904726	rs11587278	G	T	0.71	2.97E-05	0.8260	0.1831	5.32E-01	-0.1567	0.2434	4.88E-01	-0.1461	0.2092
1:228904822	rs11585991	T	C	0.71	3.00E-05	0.8252	0.1831	5.31E-01	-0.1572	0.2433	4.87E-01	-0.1464	0.2091
1:228905135	rs72751955	A	C	0.72	2.85E-05	0.8241	0.1822	5.46E-01	-0.1510	0.2425	4.78E-01	-0.1488	0.2083
1:228905750	rs72751956	C	A	0.72	2.16E-05	0.8264	0.1796	5.57E-01	-0.1457	0.2411	4.65E-01	-0.1516	0.2062
1:228907291	rs6663301	T	G	0.72	3.32E-05	0.8180	0.1826	5.34E-01	-0.1552	0.2421	4.83E-01	-0.1470	0.2083
1:228907432	rs6696887	C	T	0.72	2.49E-05	0.8185	0.1795	5.56E-01	-0.1457	0.2403	4.62E-01	-0.1523	0.2056
1:228913267	rs72751961	C	T	0.73	3.37E-05	0.7978	0.1783	5.58E-01	-0.1435	0.2380	4.48E-01	-0.1551	0.2031
1:228913361	rs72751962	G	A	0.72	4.92E-05	0.7936	0.1818	5.38E-01	-0.1519	0.2395	4.67E-01	-0.1508	0.2059
1:228913490	rs140855875	R	D	0.72	4.97E-05	0.7930	0.1818	5.38E-01	-0.1519	0.2395	4.66E-01	-0.1509	0.2058
1:228913562	rs72751962	G	T	0.73	3.48E-05	0.7969	0.1785	5.59E-01	-0.1430	0.2381	4.45E-01	-0.1562	0.2032
1:228915602	rs72754025	C	T	0.73	4.18E-05	0.7859	0.1781	5.55E-01	-0.1438	0.2368	4.42E-01	-0.1565	0.2021
1:228918113	rs201035194	G	A	0.73	3.44E-05	0.8120	0.1817	5.24E-01	-0.1589	0.2441	3.99E-01	-0.1756	0.2067
1:230222924	rs35941759	C	A	0.88	7.45E-02	0.6034	0.3342	1.19E-01	-0.5887	0.3662	8.70E-05	-1.2464	0.2950
1:37773846	rs12564259	C	T	0.94	8.30E-01	-0.0907	0.4221	6.72E-05	546.0910	340.4330	3.58E-01	0.3821	0.4121
1:37773854	rs12564265	C	T	0.94	8.29E-01	-0.0909	0.4222	6.09E-05	581.8000	369.7070	3.58E-01	0.3822	0.4122
1:37774702	rs78431607	G	A	0.94	8.26E-01	-0.0935	0.4251	9.28E-05	281.6210	185.6000	3.49E-01	0.3915	0.4150
1:37774935	rs79047500	G	T	0.94	8.25E-01	-0.0938	0.4254	9.25E-05	280.5200	186.0840	3.49E-01	0.3923	0.4152
1:37775795	rs12564821	C	T	0.94	8.24E-01	-0.0943	0.4259	8.91E-05	272.9890	184.8550	3.49E-01	0.3927	0.4157
1:37775871	rs12564825	C	G	0.94	8.25E-01	-0.0942	0.4260	8.91E-05	272.9880	184.8590	3.49E-01	0.3930	0.4157
1:37775982	rs12562839	G	A	0.94	8.23E-01	-0.0955	0.4283	8.99E-05	191.0610	126.2820	3.43E-01	0.3994	0.4180

1:37776808	rs80157204	G	T	0.95	6.24E-01	-0.2242	0.4575	7.70E-05	158.0860	99.1530	3.01E-01	0.4665	0.4465
1:37777290	rs56198153	A	C	0.95	6.21E-01	-0.2254	0.4561	5.40E-05	162.3330	98.2534	3.00E-01	0.4653	0.4451
1:37778527	rs75604453	C	A	0.95	6.16E-01	-0.2293	0.4580	6.84E-05	125.0730	79.6297	2.95E-01	0.4724	0.4469
1:37778564	rs75340595	C	T	0.95	6.16E-01	-0.2296	0.4581	3.88E-05	158.8530	100.2190	2.94E-01	0.4731	0.4470
1:37778661	rs1331335	A	G	0.95	6.16E-01	-0.2297	0.4582	4.08E-05	153.1560	95.8240	2.95E-01	0.4731	0.4472
1:37778687	rs1331334	A	G	0.95	6.15E-01	-0.2314	0.4599	5.57E-05	123.2690	80.2458	2.90E-01	0.4792	0.4488
1:66863969	rs55868520	C	T	0.78	5.60E-01	-0.1434	0.2458	9.60E-05	1.1976	0.3257	1.54E-01	0.3438	0.2381
1:66864865	rs11208846	T	C	0.78	5.58E-01	-0.1440	0.2455	9.57E-05	1.1968	0.3255	1.54E-01	0.3434	0.2379
1:66866041	rs11208847	T	C	0.78	5.55E-01	-0.1465	0.2481	9.85E-05	1.2001	0.3268	1.48E-01	0.3519	0.2402
1:66877545	rs11208853	A	G	0.78	5.36E-01	-0.1504	0.2429	9.89E-05	1.1822	0.3224	1.59E-01	0.3358	0.2355
1:66886311	rs35152634	R	I	0.78	5.18E-01	-0.1556	0.2406	9.45E-05	1.1754	0.3200	1.63E-01	0.3298	0.2334
1:66889479	rs11208859	G	A	0.78	5.26E-01	-0.1525	0.2406	9.68E-05	1.1720	0.3194	1.61E-01	0.3315	0.2334
1:66891210	rs12067979	A	G	0.78	5.33E-01	-0.1500	0.2410	9.88E-05	1.1705	0.3193	1.58E-01	0.3342	0.2336
1:7520980	rs1342480	G	A	0.69	5.39E-01	-0.1240	0.2019	8.47E-01	-0.0489	0.2490	9.82E-05	0.7296	0.1742
1:79062751	rs1475735	T	A	0.68	4.31E-05	0.9319	0.2116	6.89E-01	-0.1222	0.3050	1.27E-01	-0.3659	0.2364
1:80037315	rs7411157	C	T	0.65	1.67E-01	0.4724	0.3398	6.16E-05	-1.6620	0.4194	2.41E-01	-0.3974	0.3357
1:92628911	rs545896679	R	D	0.53	5.50E-05	-0.7463	0.1722	1.86E-03	0.7538	0.2440	2.95E-03	0.5620	0.1809
1:96316274	rs4949973	T	C	0.89	1.16E-01	-0.5032	0.3175	8.43E-03	1.2700	0.5210	9.12E-05	1.1736	0.2787
1:96332886	rs12035636	T	C	0.89	1.14E-01	-0.5074	0.3182	8.36E-03	1.2749	0.5223	9.14E-05	1.1763	0.2793
1:96333489	rs61786499	T	C	0.89	1.01E-01	-0.5325	0.3219	7.43E-03	1.3136	0.5319	6.46E-05	1.2137	0.2814
1:96337925	rs12040559	T	G	0.89	1.11E-01	-0.5225	0.3245	7.69E-03	1.3124	0.5336	6.91E-05	1.2178	0.2836
1:96344574	rs12023633	C	A	0.89	1.02E-01	-0.5309	0.3214	7.45E-03	1.3116	0.5312	6.72E-05	1.2094	0.2811
1:96350922	rs12048190	T	C	0.89	9.96E-02	-0.5358	0.3221	7.30E-03	1.3187	0.5330	6.35E-05	1.2161	0.2816
1:96360873	rs61786503	T	C	0.89	9.91E-02	-0.5388	0.3235	7.72E-03	1.3169	0.5367	6.82E-05	1.2168	0.2831
1:96361709	rs12035421	C	A	0.89	9.89E-02	-0.5391	0.3234	7.71E-03	1.3171	0.5367	6.81E-05	1.2165	0.2831
1:96368054	rs61786505	G	A	0.89	9.89E-02	-0.5369	0.3221	7.24E-03	1.3207	0.5332	6.40E-05	1.2155	0.2817
1:96377746	rs11165516	C	T	0.89	1.08E-01	-0.5181	0.3194	7.90E-03	1.2908	0.5253	8.48E-05	1.1863	0.2802
1:96468615	rs10458509	C	T	0.88	5.85E-01	-0.1810	0.3318	1.05E-02	1.2270	0.5138	7.43E-05	1.2161	0.2847
20:14399700	rs6042722	T	G	0.87	9.39E-05	1.1428	0.2736	5.04E-01	-0.2485	0.3643	9.67E-01	-0.0127	0.3080
20:14414373	rs6079437	T	C	0.86	7.06E-05	1.0959	0.2573	4.79E-01	-0.2534	0.3500	9.24E-01	-0.0277	0.2909
20:14416323	rs79015429	A	G	0.87	8.61E-05	1.1346	0.2700	5.16E-01	-0.2396	0.3611	9.89E-01	-0.0041	0.3043
20:14422187	rs4814306	C	T	0.85	7.16E-05	1.1192	0.2630	5.96E-01	-0.1869	0.3463	9.54E-01	0.0171	0.2973
20:14432472	rs73612355	C	T	0.84	6.60E-05	1.1154	0.2606	5.92E-01	-0.1883	0.3451	9.67E-01	0.0124	0.2951
20:14454344	rs6079458	A	G	0.85	8.76E-05	1.1327	0.2699	4.56E-01	-0.2641	0.3471	9.42E-01	0.0223	0.3041

20:14467217	rs2145008	G	A	0.84	5.06E-05	1.1335	0.2601	1.84E-01	-0.4405	0.3264	9.67E-01	-0.0124	0.2958
20:14476048	rs2327854	A	G	0.84	2.79E-05	1.1380	0.2513	1.67E-01	-0.4437	0.3172	9.20E-01	-0.0291	0.2886
20:14478215	rs7269525	C	T	0.84	2.54E-05	1.1473	0.2519	1.61E-01	-0.4514	0.3180	9.14E-01	-0.0313	0.2898
20:14479730	rs6079464	C	T	0.84	4.34E-05	1.1340	0.2577	1.48E-01	-0.4695	0.3201	9.43E-01	-0.0210	0.2937
20:14482770	rs73612363	G	A	0.84	4.59E-05	1.1254	0.2566	1.48E-01	-0.4669	0.3187	9.16E-01	-0.0310	0.2923
20:14491658	rs6074746	A	G	0.84	4.46E-05	1.1358	0.2585	1.42E-01	-0.4761	0.3201	9.25E-01	-0.0278	0.2946
20:14493800	rs6079465	G	T	0.84	4.50E-05	1.1381	0.2592	1.41E-01	-0.4784	0.3206	9.27E-01	-0.0272	0.2953
20:14500103	rs4813160	T	C	0.84	4.47E-05	1.1433	0.2603	1.39E-01	-0.4830	0.3213	9.33E-01	-0.0250	0.2966
20:14501598	rs202009454	R	I	0.84	4.55E-05	1.1462	0.2612	1.37E-01	-0.4856	0.3219	9.40E-01	-0.0227	0.2976
20:14503474	rs4814309	G	C	0.84	4.71E-05	1.1459	0.2618	1.39E-01	-0.4845	0.3222	9.42E-01	-0.0218	0.2980
20:14505942	rs6074749	C	A	0.84	4.59E-05	1.1472	0.2616	1.37E-01	-0.4872	0.3223	9.37E-01	-0.0238	0.2980
20:3067254	rs61138008	C	T	0.78	3.66E-06	-1.5243	0.2992	1.86E-01	0.5933	0.4514	6.87E-04	1.1532	0.3212
20:36793529	rs2076380	G	A	0.64	8.28E-06	-0.9316	0.1913	4.15E-01	0.2291	0.2832	4.07E-02	0.4525	0.2161
20:49830783	rs4811130	C	T	0.59	3.95E-01	0.2035	0.2387	5.64E-01	-0.1602	0.2815	9.22E-05	-0.8681	0.2063
20:52738421	rs62214475	T	C	0.90	5.36E-05	-1.1054	0.2547	9.87E-02	0.9189	0.6411	8.79E-02	0.4896	0.2820
20:52738555	rs16998967	T	A	0.90	5.33E-05	-1.1053	0.2546	9.86E-02	0.9192	0.6411	8.77E-02	0.4897	0.2819
20:6099593	rs528959334	R	D	0.82	5.08E-02	0.5608	0.2829	5.75E-05	-1.0437	0.2369	6.43E-02	-0.5266	0.2791
21:23611869	rs11911981	G	A	0.75	8.75E-01	-0.0337	0.2148	3.98E-05	1.4004	0.3815	1.48E-02	0.5036	0.2004
22:17385750	rs5748727	A	T	0.95	9.80E-01	0.0128	0.5013	9.39E-05	-2.0878	0.4469	7.18E-01	-0.1787	0.4923
22:22463325	rs12172019	G	C	0.79	6.89E-03	-0.7123	0.2557	1.30E-01	0.5133	0.3542	6.66E-05	1.0028	0.2330
22:22874320	rs362044	A	G	0.63	7.41E-01	0.1039	0.3142	7.94E-05	-1.3037	0.3292	6.76E-04	-1.0038	0.2791
22:22874965	rs362208	G	A	0.65	9.64E-01	0.0149	0.3296	7.57E-05	-1.3590	0.3374	5.06E-04	-1.0738	0.2911
22:28016683	rs11702931	C	T	0.84	3.01E-02	-0.5084	0.2300	2.19E-01	0.3837	0.3234	4.46E-05	0.8994	0.2034
22:28022026	rs73162330	G	A	0.85	1.24E-01	-0.3714	0.2395	2.61E-01	0.3548	0.3268	6.38E-05	0.9012	0.2088
22:34863375	rs130578	G	A	0.81	5.11E-05	0.8874	0.2038	7.09E-01	0.1191	0.3078	5.29E-01	-0.1464	0.2309
22:34863764	rs130579	C	T	0.81	5.08E-05	0.8868	0.2036	7.10E-01	0.1188	0.3076	5.27E-01	-0.1467	0.2307
22:34864558	rs28971240	A	C	0.81	4.98E-05	0.8872	0.2034	7.12E-01	0.1178	0.3074	5.26E-01	-0.1470	0.2306
22:34864692	rs145660198	I	R	0.81	5.04E-05	0.8875	0.2036	7.11E-01	0.1182	0.3076	5.28E-01	-0.1467	0.2308
22:34865011	rs200139501	I	R	0.81	4.96E-05	0.8876	0.2034	7.13E-01	0.1174	0.3075	5.26E-01	-0.1471	0.2306
22:34869196	rs4423	G	A	0.81	5.95E-05	0.8783	0.2038	6.97E-01	0.1246	0.3081	5.29E-01	-0.1458	0.2303
22:34869571	rs130586	C	A	0.81	4.56E-05	0.8936	0.2037	7.12E-01	0.1180	0.3085	5.23E-01	-0.1485	0.2312
22:34871008	rs135163	T	C	0.81	5.02E-05	0.8850	0.2030	7.05E-01	0.1207	0.3072	5.24E-01	-0.1475	0.2301
22:34871742	rs130588	G	T	0.81	4.75E-05	0.8867	0.2027	7.15E-01	0.1163	0.3068	5.22E-01	-0.1480	0.2299
22:34872806	rs127301	T	C	0.81	4.72E-05	0.8873	0.2027	7.16E-01	0.1156	0.3068	5.22E-01	-0.1482	0.2299

22:34873663	rs135165	A	T	0.81	4.69E-05	0.8873	0.2026	7.17E-01	0.1152	0.3067	5.22E-01	-0.1483	0.2299
22:34874101	rs130592	T	G	0.81	4.69E-05	0.8871	0.2026	7.17E-01	0.1154	0.3066	5.21E-01	-0.1483	0.2298
22:34875943	rs5994940	A	G	0.81	4.44E-05	0.8886	0.2022	7.27E-01	0.1108	0.3062	5.18E-01	-0.1492	0.2296
22:34882064	rs130694	A	G	0.81	4.16E-05	0.8904	0.2017	7.32E-01	0.1089	0.3059	5.16E-01	-0.1499	0.2293
22:34882579	rs130695	A	G	0.81	4.26E-05	0.8898	0.2019	7.30E-01	0.1095	0.3061	5.11E-01	-0.1517	0.2294
22:34884877	rs5755064	C	G	0.81	3.72E-05	0.8959	0.2015	7.42E-01	0.1048	0.3062	5.17E-01	-0.1495	0.2295
22:34887270	rs4424	G	C	0.81	4.01E-05	0.8906	0.2013	7.39E-01	0.1055	0.3053	5.13E-01	-0.1507	0.2289
22:34889460	rs130699	A	C	0.81	4.12E-05	0.8885	0.2012	7.43E-01	0.1040	0.3049	5.12E-01	-0.1509	0.2287
22:34889516	rs130700	G	A	0.81	4.12E-05	0.8887	0.2012	7.43E-01	0.1038	0.3050	5.12E-01	-0.1508	0.2288
22:34889754	rs130701	T	C	0.81	4.11E-05	0.8885	0.2012	7.43E-01	0.1038	0.3049	5.12E-01	-0.1510	0.2287
22:34890899	rs130703	A	T	0.81	4.07E-05	0.8888	0.2011	7.45E-01	0.1027	0.3048	5.11E-01	-0.1511	0.2286
22:34893052	rs130704	A	G	0.81	4.03E-05	0.8892	0.2010	7.47E-01	0.1019	0.3047	5.11E-01	-0.1512	0.2286
22:34894283	rs130706	G	T	0.81	4.03E-05	0.8892	0.2010	7.48E-01	0.1018	0.3047	5.11E-01	-0.1512	0.2286
22:34894906	rs9607147	A	G	0.81	4.03E-05	0.8892	0.2010	7.48E-01	0.1018	0.3047	5.11E-01	-0.1512	0.2286
22:34896076	rs112578268	R	I	0.81	4.03E-05	0.8892	0.2011	7.47E-01	0.1018	0.3047	5.11E-01	-0.1511	0.2286
22:34896078	rs112556622	A	G	0.81	4.04E-05	0.8892	0.2011	7.47E-01	0.1019	0.3048	5.11E-01	-0.1513	0.2287
22:34896210	rs73160354	A	G	0.81	4.03E-05	0.8892	0.2010	7.48E-01	0.1018	0.3047	5.11E-01	-0.1512	0.2286
22:34896294	rs535429597	R	I	0.81	4.06E-05	0.8897	0.2013	7.47E-01	0.1022	0.3050	5.13E-01	-0.1507	0.2289
22:34896350	rs115349747	A	G	0.83	3.13E-05	1.0084	0.2243	8.93E-01	0.0482	0.3423	3.72E-01	-0.2296	0.2554
22:34896502	rs111394994	G	A	0.81	3.89E-05	0.8970	0.2023	7.69E-01	0.0938	0.3068	5.11E-01	-0.1524	0.2302
22:34896515	rs556476384	R	D	0.86	5.85E-05	1.1092	0.2570	4.59E-01	0.3069	0.4006	8.76E-01	-0.0457	0.2915
22:34896518	rs375805256	R	D	0.86	5.85E-05	1.1092	0.2571	4.58E-01	0.3073	0.4007	8.76E-01	-0.0459	0.2916
22:34898385	rs2227030	C	T	0.81	4.01E-05	0.8892	0.2010	7.48E-01	0.1014	0.3047	5.11E-01	-0.1513	0.2286
22:34898423	rs9607149	T	C	0.84	9.14E-05	0.8980	0.2146	9.65E-01	0.0144	0.3230	4.49E-01	-0.1835	0.2404
22:34899564	rs2032464	C	T	0.81	4.02E-05	0.8895	0.2011	7.48E-01	0.1016	0.3049	5.11E-01	-0.1514	0.2287
22:34901525	rs5755071	A	G	0.81	3.88E-05	0.8913	0.2010	7.47E-01	0.1020	0.3050	5.10E-01	-0.1515	0.2288
22:34902758	rs130709	T	G	0.84	9.37E-05	0.8975	0.2149	9.61E-01	0.0161	0.3235	4.49E-01	-0.1835	0.2406
22:34903306	rs130710	T	C	0.81	4.15E-05	0.8893	0.2015	7.42E-01	0.1043	0.3054	5.11E-01	-0.1516	0.2290
22:34904107	rs130715	G	T	0.81	4.13E-05	0.8891	0.2013	7.43E-01	0.1037	0.3052	5.11E-01	-0.1515	0.2289
22:34904220	rs130716	R	D	0.81	4.13E-05	0.8891	0.2013	7.43E-01	0.1037	0.3052	5.11E-01	-0.1515	0.2289
22:34904762	rs73160361	C	T	0.81	4.29E-05	0.8873	0.2014	7.42E-01	0.1041	0.3052	5.10E-01	-0.1516	0.2288
22:34905689	rs130717	A	T	0.81	4.30E-05	0.8873	0.2015	7.42E-01	0.1041	0.3052	5.10E-01	-0.1516	0.2289
22:34905776	rs130718	A	G	0.81	4.30E-05	0.8873	0.2015	7.42E-01	0.1041	0.3052	5.10E-01	-0.1516	0.2289
22:34906181	rs5755072	C	A	0.81	4.31E-05	0.8872	0.2015	7.42E-01	0.1043	0.3052	5.10E-01	-0.1516	0.2289

22:34906241	rs130719	A	G	0.81	4.15E-05	0.8891	0.2014	7.43E-01	0.1040	0.3053	5.11E-01	-0.1515	0.2289
22:34906816	rs713568	C	T	0.81	4.32E-05	0.8872	0.2015	7.42E-01	0.1044	0.3052	5.10E-01	-0.1516	0.2289
22:34906973	rs19445	C	T	0.81	4.22E-05	0.8890	0.2016	7.43E-01	0.1040	0.3056	5.08E-01	-0.1526	0.2291
22:34908053	rs130720	C	T	0.81	4.29E-05	0.8874	0.2015	7.42E-01	0.1042	0.3052	5.10E-01	-0.1516	0.2289
22:34909613	rs4820149	A	G	0.81	3.95E-05	0.8909	0.2012	7.27E-01	0.1109	0.3058	5.10E-01	-0.1517	0.2289
22:34910004	rs130721	R	D	0.84	9.06E-05	0.8990	0.2147	9.42E-01	0.0241	0.3241	4.51E-01	-0.1827	0.2406
22:34910847	rs130722	C	T	0.81	4.30E-05	0.8874	0.2015	7.42E-01	0.1043	0.3052	5.10E-01	-0.1517	0.2289
22:34911429	rs130723	T	C	0.81	4.02E-05	0.8889	0.2009	7.26E-01	0.1108	0.3053	5.13E-01	-0.1505	0.2285
22:34914352	rs4592951	C	T	0.81	4.09E-05	0.8896	0.2013	7.44E-01	0.1034	0.3051	5.11E-01	-0.1515	0.2289
22:34914969	rs2179149	G	C	0.81	4.09E-05	0.8895	0.2013	7.44E-01	0.1035	0.3051	5.10E-01	-0.1516	0.2289
22:34915292	rs2413237	C	T	0.81	4.09E-05	0.8896	0.2013	7.45E-01	0.1032	0.3051	5.10E-01	-0.1517	0.2289
22:34915547	rs5749868	A	G	0.81	4.09E-05	0.8896	0.2013	7.45E-01	0.1032	0.3051	5.10E-01	-0.1517	0.2289
22:34916665	rs5999421	C	A	0.81	4.10E-05	0.8895	0.2013	7.44E-01	0.1033	0.3051	5.10E-01	-0.1517	0.2289
22:34917698	rs1985898	A	G	0.81	3.83E-05	0.8914	0.2009	7.30E-01	0.1092	0.3054	5.12E-01	-0.1510	0.2286
22:34917744	rs1985897	G	A	0.81	4.00E-05	0.8911	0.2014	7.44E-01	0.1035	0.3053	5.10E-01	-0.1518	0.2290
22:34918333	rs5755076	C	T	0.81	3.95E-05	0.8918	0.2014	7.44E-01	0.1036	0.3054	5.11E-01	-0.1514	0.2291
22:34920698	rs4820151	C	T	0.81	4.11E-05	0.8897	0.2014	7.43E-01	0.1037	0.3052	5.10E-01	-0.1518	0.2290
22:34923409	rs9621965	A	G	0.81	3.85E-05	0.8913	0.2009	7.29E-01	0.1098	0.3053	5.12E-01	-0.1509	0.2287
22:34923678	rs2064089	A	G	0.81	3.85E-05	0.8913	0.2009	7.29E-01	0.1098	0.3053	5.12E-01	-0.1509	0.2287
22:34923959	rs2064091	G	C	0.81	3.95E-05	0.8900	0.2010	7.29E-01	0.1097	0.3053	5.12E-01	-0.1509	0.2286
22:34926457	rs8135889	A	G	0.81	4.00E-05	0.8913	0.2014	7.43E-01	0.1038	0.3055	5.09E-01	-0.1521	0.2291
22:34926540	rs8136010	A	G	0.81	4.00E-05	0.8913	0.2014	7.43E-01	0.1038	0.3055	5.09E-01	-0.1521	0.2291
22:34928548	rs5755080	T	C	0.81	4.12E-05	0.8930	0.2022	7.47E-01	0.1025	0.3059	5.10E-01	-0.1524	0.2299
22:34930216	rs5749872	A	G	0.81	3.99E-05	0.8915	0.2015	7.43E-01	0.1038	0.3055	5.10E-01	-0.1520	0.2291
22:34934832	rs5845179	I	R	0.80	6.78E-05	0.8812	0.2063	7.30E-01	0.1089	0.3048	5.57E-01	-0.1376	0.2327
22:34936420	rs71322925	I	R	0.82	2.21E-05	0.9324	0.2029	9.58E-01	-0.0169	0.3123	2.58E-01	-0.2643	0.2314
22:34937276	rs5749873	T	A	0.81	4.07E-05	0.8906	0.2015	7.43E-01	0.1038	0.3054	5.10E-01	-0.1519	0.2291
22:34938045	rs5994964	A	C	0.81	4.16E-05	0.8891	0.2014	7.45E-01	0.1030	0.3050	5.05E-01	-0.1537	0.2289
22:34939063	rs4621256	C	G	0.81	4.17E-05	0.8907	0.2018	7.38E-01	0.1061	0.3057	5.09E-01	-0.1526	0.2294
22:34940082	rs5999431	A	G	0.81	4.08E-05	0.8906	0.2015	7.43E-01	0.1039	0.3054	5.10E-01	-0.1520	0.2291
22:34940942	rs5755093	G	T	0.81	4.16E-05	0.8908	0.2018	7.37E-01	0.1064	0.3058	5.08E-01	-0.1528	0.2294
22:34941576	rs4821261	C	T	0.84	9.11E-05	0.8985	0.2147	9.61E-01	0.0160	0.3234	4.51E-01	-0.1826	0.2405
22:34942090	rs5755095	T	C	0.81	4.02E-05	0.8913	0.2015	7.43E-01	0.1041	0.3054	5.10E-01	-0.1519	0.2291
22:34944156	rs5755101	C	A	0.81	4.17E-05	0.8908	0.2019	7.37E-01	0.1065	0.3058	5.08E-01	-0.1527	0.2294

22:34944235	rs5994965	C	T	0.81	4.08E-05	0.8905	0.2015	7.43E-01	0.1038	0.3054	5.10E-01	-0.1519	0.2291
22:34944420	rs5994966	T	C	0.81	4.12E-05	0.8902	0.2016	7.42E-01	0.1043	0.3055	5.10E-01	-0.1518	0.2291
22:34945295	rs5749877	A	T	0.81	4.09E-05	0.8905	0.2015	7.44E-01	0.1037	0.3054	5.10E-01	-0.1520	0.2291
22:34945447	rs5755102	T	C	0.81	4.09E-05	0.8905	0.2015	7.44E-01	0.1037	0.3054	5.10E-01	-0.1520	0.2291
22:34945675	rs56854366	D	R	0.77	3.89E-05	0.8879	0.2003	6.87E-01	-0.1169	0.2857	2.07E-01	-0.2880	0.2256
22:34948085	rs58174594	T	A	0.80	4.79E-05	0.8853	0.2024	6.15E-01	0.1611	0.3092	7.43E-01	-0.0760	0.2302
22:34951545	rs5994969	G	A	0.81	4.12E-05	0.8901	0.2015	7.43E-01	0.1041	0.3054	5.10E-01	-0.1521	0.2291
22:34955094	rs5999438	A	C	0.80	6.85E-05	0.8802	0.2062	7.24E-01	0.1121	0.3066	5.46E-01	-0.1413	0.2325
22:34955097	rs5994972	A	C	0.80	6.84E-05	0.8802	0.2062	7.24E-01	0.1121	0.3066	5.46E-01	-0.1413	0.2325
22:34955877	rs7290415	G	T	0.81	4.05E-05	0.8904	0.2014	7.45E-01	0.1031	0.3052	5.10E-01	-0.1520	0.2290
22:34956307	rs6518871	C	T	0.81	4.10E-05	0.8904	0.2016	7.44E-01	0.1034	0.3055	5.10E-01	-0.1520	0.2291
22:34956338	rs67375911	I	R	0.81	4.04E-05	0.8906	0.2014	7.45E-01	0.1032	0.3052	5.09E-01	-0.1521	0.2290
22:34957088	rs2179150	C	T	0.81	4.10E-05	0.8900	0.2014	7.42E-01	0.1042	0.3053	5.11E-01	-0.1516	0.2290
22:34959246	rs742084	T	C	0.81	4.22E-05	0.8911	0.2021	7.35E-01	0.1075	0.3060	5.08E-01	-0.1530	0.2296
22:34960675	rs71322928	D	R	0.81	4.15E-05	0.8901	0.2016	7.39E-01	0.1053	0.3053	5.12E-01	-0.1513	0.2292
22:34960988	rs9619459	G	A	0.81	4.05E-05	0.8900	0.2013	7.44E-01	0.1033	0.3051	5.10E-01	-0.1516	0.2289
22:34961453	rs377057880	I	R	0.81	3.67E-05	0.8980	0.2018	7.91E-01	0.0844	0.3057	5.10E-01	-0.1525	0.2298
22:34961850	rs9607157	A	C	0.81	4.03E-05	0.8897	0.2012	7.45E-01	0.1029	0.3050	5.10E-01	-0.1515	0.2288
22:34962139	rs5755109	G	A	0.81	4.04E-05	0.8897	0.2012	7.45E-01	0.1029	0.3050	5.11E-01	-0.1515	0.2288
22:34964097	rs5999444	C	T	0.81	3.94E-05	0.8932	0.2017	7.45E-01	0.1032	0.3058	5.03E-01	-0.1545	0.2294
22:34964955	rs5749882	G	A	0.81	4.02E-05	0.8895	0.2011	7.47E-01	0.1021	0.3049	5.10E-01	-0.1515	0.2287
22:34965192	rs34127314	I	R	0.81	4.02E-05	0.8905	0.2013	7.45E-01	0.1030	0.3052	5.09E-01	-0.1522	0.2289
22:34965213	rs2205812	T	C	0.81	3.99E-05	0.8901	0.2011	7.48E-01	0.1017	0.3049	5.10E-01	-0.1517	0.2287
22:34965261	rs2205813	C	G	0.81	3.99E-05	0.8901	0.2011	7.48E-01	0.1017	0.3049	5.10E-01	-0.1517	0.2287
22:34965486	rs2205814	C	T	0.81	3.84E-05	0.8926	0.2012	7.47E-01	0.1021	0.3051	5.09E-01	-0.1521	0.2290
22:34965893	rs59875330	I	R	0.84	9.19E-05	0.8937	0.2137	9.44E-01	0.0232	0.3221	4.61E-01	-0.1776	0.2394
22:34965995	rs58702312	I	R	0.81	3.98E-05	0.8917	0.2014	7.31E-01	0.1087	0.3052	5.11E-01	-0.1516	0.2291
22:34966012	rs5749883	A	G	0.81	4.02E-05	0.8892	0.2010	7.48E-01	0.1016	0.3047	5.11E-01	-0.1513	0.2286
22:34967282	rs5994976	C	T	0.81	4.01E-05	0.8892	0.2010	7.48E-01	0.1015	0.3047	5.11E-01	-0.1513	0.2286
22:34967585	rs5994977	T	A	0.81	4.01E-05	0.8892	0.2010	7.48E-01	0.1014	0.3047	5.11E-01	-0.1513	0.2286
22:34967969	rs5755112	G	A	0.81	4.01E-05	0.8892	0.2010	7.48E-01	0.1014	0.3047	5.11E-01	-0.1513	0.2286
22:34968166	rs5755113	T	G	0.81	4.01E-05	0.8892	0.2010	7.48E-01	0.1014	0.3047	5.11E-01	-0.1513	0.2286
22:34968614	rs2205815	G	A	0.81	4.02E-05	0.8896	0.2011	7.46E-01	0.1023	0.3049	5.10E-01	-0.1518	0.2287
22:34969269	rs10775729	C	T	0.81	4.14E-05	0.8896	0.2015	7.41E-01	0.1045	0.3052	5.09E-01	-0.1521	0.2290

22:34969816	rs138087815	I	R	0.81	4.18E-05	0.8890	0.2015	7.46E-01	0.1026	0.3051	5.09E-01	-0.1520	0.2290
22:34970357	rs9607158	T	A	0.81	4.01E-05	0.8893	0.2010	7.48E-01	0.1016	0.3047	5.11E-01	-0.1512	0.2286
22:34970840	rs9610055	T	A	0.81	4.01E-05	0.8892	0.2010	7.48E-01	0.1015	0.3047	5.11E-01	-0.1513	0.2286
22:34973064	rs5999446	C	T	0.84	8.85E-05	0.8937	0.2131	9.51E-01	0.0202	0.3212	4.54E-01	-0.1801	0.2389
22:34974578	rs185270732	A	G	0.81	3.90E-05	0.8931	0.2015	7.47E-01	0.1021	0.3056	5.11E-01	-0.1516	0.2293
22:34974757	rs374396311	T	A	0.85	3.81E-05	0.9375	0.2112	9.19E-01	0.0332	0.3198	6.02E-01	-0.1261	0.2407
22:34974758	rs377279192	A	T	0.85	3.81E-05	0.9375	0.2112	9.19E-01	0.0332	0.3198	6.03E-01	-0.1260	0.2407
22:34974959	rs9607160	T	A	0.81	4.12E-05	0.8957	0.2028	7.58E-01	0.0981	0.3073	5.09E-01	-0.1531	0.2305
22:34981629	rs5749885	C	T	0.83	4.25E-05	0.8955	0.2032	8.04E-01	0.0785	0.3034	6.68E-01	-0.0996	0.2313
22:43826818	rs12628226	C	A	0.93	4.36E-02	-0.6611	0.3224	1.01E-05	1037.5000	639.3740	3.81E-01	0.2884	0.3264
22:44725640	rs66803227	G	A	0.53	6.70E-05	0.8057	0.1884	5.35E-01	-0.1770	0.2866	5.50E-01	-0.1277	0.2126
22:44741271	rs7410782	G	A	0.53	6.33E-05	0.7926	0.1847	7.79E-01	-0.0774	0.2823	3.78E-01	-0.1845	0.2078
22:50776096	rs564180957	T	G	0.73	3.41E-05	1.0692	0.2391	8.70E-01	-0.0504	0.3149	7.41E-01	-0.0908	0.2735
2:107794848	rs10184143	A	C	0.61	1.56E-01	-0.2677	0.1875	3.23E-03	0.6194	0.2079	2.43E-05	0.7368	0.1603
2:110265558	rs9330310	T	G	0.46	5.36E-05	0.8705	0.2006	7.83E-03	-0.6776	0.2463	1.47E-02	-0.5441	0.2162
2:110273380	rs9330311	T	C	0.56	4.61E-05	-0.8953	0.2042	3.87E-03	0.7965	0.2623	2.88E-02	0.5001	0.2230
2:110283216	rs12998808	C	A	0.56	7.40E-05	-0.8396	0.1977	7.31E-03	0.7548	0.2661	5.53E-02	0.4232	0.2163
2:110286299	rs3934873	G	A	0.45	8.16E-05	0.8434	0.2000	6.88E-03	-0.7788	0.2732	6.05E-02	-0.4188	0.2187
2:110289984	rs9330320	G	C	0.45	8.45E-05	0.8411	0.1999	7.23E-03	-0.7774	0.2744	6.54E-02	-0.4109	0.2187
2:110305754	rs36031306	D	R	0.47	9.30E-05	0.8791	0.2103	6.86E-03	-0.7915	0.2737	1.84E-02	-0.5472	0.2254
2:121269553	rs66467070	G	A	0.82	2.00E-01	0.3620	0.2810	4.87E-01	-0.2243	0.3170	3.86E-05	-1.0771	0.2413
2:121271649	rs4849900	T	C	0.88	2.22E-01	0.4045	0.3297	3.42E-01	-0.3635	0.3771	5.39E-05	-1.2416	0.2844
2:126989635	rs35374245	R	D	0.65	1.80E-01	0.2681	0.1990	9.35E-01	-0.0201	0.2542	3.48E-05	-0.7677	0.1709
2:126990186	rs28530590	A	C	0.64	1.99E-01	0.2566	0.1988	8.66E-01	-0.0414	0.2541	4.50E-05	-0.7562	0.1711
2:126991224	rs68175812	A	C	0.64	1.92E-01	0.2626	0.2000	9.41E-01	-0.0183	0.2546	3.72E-05	-0.7685	0.1718
2:126991274	rs68107644	T	G	0.64	1.96E-01	0.2596	0.1997	8.56E-01	-0.0449	0.2557	5.07E-05	-0.7556	0.1724
2:126994966	rs390949	T	C	0.64	2.13E-01	0.2504	0.2002	8.72E-01	-0.0397	0.2557	3.84E-05	-0.7669	0.1718
2:126996142	rs374585	G	A	0.64	2.15E-01	0.2496	0.2003	8.73E-01	-0.0396	0.2559	3.79E-05	-0.7679	0.1718
2:127008196	rs771078	T	G	0.64	2.31E-01	0.2427	0.2019	8.76E-01	-0.0390	0.2577	3.35E-05	-0.7775	0.1726
2:127009083	rs360256	T	A	0.64	2.36E-01	0.2414	0.2028	8.67E-01	-0.0420	0.2589	3.42E-05	-0.7800	0.1734
2:127011178	rs360253	A	G	0.64	2.35E-01	0.2410	0.2022	8.77E-01	-0.0387	0.2581	3.33E-05	-0.7788	0.1728
2:127012268	rs360252	C	T	0.64	2.35E-01	0.2411	0.2021	8.76E-01	-0.0391	0.2581	3.27E-05	-0.7793	0.1727
2:127013973	rs360249	C	G	0.64	2.36E-01	0.2404	0.2022	8.75E-01	-0.0392	0.2582	3.25E-05	-0.7798	0.1728
2:127015379	rs360248	C	G	0.64	2.38E-01	0.2396	0.2024	8.76E-01	-0.0390	0.2584	3.22E-05	-0.7806	0.1729

2:127016832	rs61411848	A	G	0.71	7.15E-02	0.3967	0.2174	9.85E-01	-0.0050	0.2736	4.66E-05	-0.8376	0.1900
2:127913835	rs729666	C	T	0.70	3.61E-01	0.1857	0.2030	9.10E-01	0.0292	0.2615	9.67E-05	-0.7372	0.1758
2:127918248	rs4663107	A	T	0.71	3.62E-01	0.1818	0.1990	8.64E-01	0.0437	0.2609	9.94E-05	-0.7217	0.1724
2:127919641	rs11682330	A	G	0.70	3.77E-01	0.1763	0.1993	8.73E-01	0.0408	0.2609	9.15E-05	-0.7254	0.1723
2:153342552	rs10195380	G	C	0.66	4.50E-02	-0.3747	0.1839	6.16E-02	0.4826	0.2661	8.88E-05	0.6903	0.1636
2:163128824	rs3747517	C	T	0.67	8.92E-02	0.3520	0.2048	9.61E-05	-0.8533	0.2085	2.42E-01	-0.2414	0.2041
2:169740286	rs560630789	R	D	0.86	4.01E-05	-1.0912	0.2466	1.93E-01	0.5247	0.4242	1.82E-01	0.3742	0.2772
2:170638416	rs74420714	A	G	0.94	8.91E-05	-1.6595	0.3959	5.04E-01	0.5911	0.9309	1.68E-01	0.6126	0.4385
2:170639616	rs116604999	A	C	0.94	8.94E-05	-1.6596	0.3960	5.05E-01	0.5895	0.9307	1.67E-01	0.6134	0.4386
2:170640965	rs145152157	G	C	0.94	8.93E-05	-1.6626	0.3967	5.02E-01	0.5943	0.9326	1.59E-01	0.6260	0.4391
2:17131093	rs36119023	G	T	0.84	8.89E-02	0.4206	0.2446	7.36E-02	-0.4477	0.2381	9.42E-05	-0.9060	0.2156
2:17132435	rs34555172	T	C	0.84	9.20E-02	0.4087	0.2400	7.43E-02	-0.4377	0.2335	9.06E-05	-0.8903	0.2113
2:17137705	rs4411692	G	A	0.84	7.99E-02	0.4145	0.2339	7.70E-02	-0.4243	0.2292	8.53E-05	-0.8723	0.2062
2:17140213	rs2341496	G	A	0.84	8.62E-02	0.3961	0.2284	8.14E-02	-0.4086	0.2246	7.86E-05	-0.8543	0.2008
2:17140218	rs2341497	G	C	0.84	8.62E-02	0.3962	0.2283	8.15E-02	-0.4085	0.2246	7.86E-05	-0.8543	0.2007
2:17182375	rs12989499	C	T	0.82	7.92E-02	0.4217	0.2374	4.68E-02	-0.4833	0.2274	9.08E-05	-0.8824	0.2095
2:17506784	rs4832491	G	A	0.88	5.31E-05	1.0439	0.2404	7.19E-01	-0.1240	0.3497	4.34E-02	-0.5441	0.2634
2:179134960	rs333985	G	T	0.93	2.67E-01	-0.4225	0.3796	5.41E-05	12.8419	13.6581	4.01E-01	0.3171	0.3750
2:181698441	rs4666624	G	T	0.41	3.22E-01	-0.2060	0.2076	6.51E-02	0.4825	0.2635	7.26E-05	0.7666	0.1792
2:181710499	rs56378630	T	C	0.53	5.44E-01	-0.1408	0.2319	7.17E-02	0.4502	0.2549	4.99E-05	0.8681	0.1978
2:181725206	rs11679476	A	G	0.54	5.46E-01	-0.1393	0.2310	5.98E-02	0.4638	0.2503	7.63E-05	0.8461	0.1984
2:205843688	rs34432147	R	D	0.43	3.31E-01	0.2095	0.2151	9.28E-05	-0.9837	0.2532	4.14E-01	-0.1746	0.2120
2:221024539	rs6748896	G	A	0.79	1.40E-01	0.3627	0.2442	9.11E-01	0.0330	0.2922	4.98E-06	-1.0261	0.2035
2:241949703	rs7571354	T	C	0.81	5.34E-05	-0.9681	0.2230	3.72E-02	0.7765	0.4050	5.04E-01	0.1697	0.2523
2:3101773	rs767885	G	A	0.88	9.14E-05	1.1996	0.2867	1.10E-01	-0.5659	0.3449	3.76E-01	-0.2863	0.3206
2:37498688	rs72792829	C	T	0.76	1.45E-03	0.6893	0.2072	1.54E-02	-0.5948	0.2468	9.96E-05	-0.8158	0.1949
2:37528311	rs57361399	C	T	0.76	1.92E-03	0.6785	0.2098	1.30E-02	-0.6143	0.2493	9.25E-05	-0.8257	0.1963
2:37603839	rs17020628	A	G	0.91	3.07E-01	0.3695	0.3606	1.49E-05	-1.4314	0.3104	9.97E-01	-0.0015	0.3579
2:41192477	rs55661203	C	A	0.93	8.55E-03	-1.0630	0.3927	8.76E-05	12.4679	7.3704	1.53E-01	0.5835	0.4032
2:51304238	rs2163018	C	T	0.71	2.35E-01	-0.2837	0.2380	4.01E-01	0.2292	0.2702	9.67E-05	0.8687	0.2071
2:65084253	rs764011	A	T	0.54	6.31E-02	-0.4248	0.2255	9.95E-05	1.0059	0.2630	8.33E-02	0.3925	0.2227
2:65092059	rs554062303	R	I	0.49	2.33E-02	-0.5196	0.2243	2.24E-05	1.0808	0.2597	1.19E-01	0.3574	0.2259
2:65448938	rs111600830	T	C	0.91	7.86E-05	-1.1938	0.2823	5.11E-02	1.0650	0.6392	7.72E-01	0.0926	0.3184
2:74059356	rs414255	C	T	0.85	2.70E-01	0.3474	0.3137	3.25E-05	-1.1769	0.2549	7.92E-02	-0.5422	0.3034

2:79968158	rs11681484	G	A	0.56	2.74E-06	0.7630	0.1475	3.10E-01	-0.2168	0.2130	3.06E-02	-0.3748	0.1690
2:82049625	rs62149760	G	A	0.88	4.66E-01	0.2264	0.3105	9.41E-01	0.0300	0.4002	8.19E-05	-1.1348	0.2674
2:82053791	rs62149762	G	A	0.85	1.97E-01	0.3757	0.2898	8.71E-01	0.0551	0.3326	3.54E-05	-1.1156	0.2486
2:82056707	rs62149763	A	G	0.82	3.52E-01	0.2638	0.2828	6.71E-01	0.1430	0.3355	3.80E-05	-1.0775	0.2412
2:82056798	rs142473614	R	I	0.82	3.52E-01	0.2638	0.2828	6.71E-01	0.1430	0.3355	3.80E-05	-1.0775	0.2412
2:82060592	rs11686486	G	T	0.85	1.97E-01	0.3758	0.2899	8.70E-01	0.0554	0.3327	3.56E-05	-1.1157	0.2487
2:82068261	rs62149764	G	A	0.85	1.97E-01	0.3759	0.2899	8.70E-01	0.0554	0.3327	3.54E-05	-1.1159	0.2486
2:82069059	rs36148655	C	A	0.85	1.96E-01	0.3773	0.2900	8.73E-01	0.0543	0.3328	3.49E-05	-1.1174	0.2487
2:82069432	rs62149766	A	G	0.85	1.96E-01	0.3774	0.2900	8.73E-01	0.0543	0.3328	3.48E-05	-1.1175	0.2487
2:82069595	rs530678060	R	I	0.82	3.49E-01	0.2654	0.2826	6.74E-01	0.1414	0.3351	3.64E-05	-1.0790	0.2408
2:82077473	rs11674245	C	A	0.85	1.97E-01	0.3764	0.2902	8.69E-01	0.0559	0.3329	3.50E-05	-1.1175	0.2488
2:82077928	rs62149767	G	A	0.82	3.47E-01	0.2663	0.2826	6.73E-01	0.1420	0.3352	3.64E-05	-1.0791	0.2409
2:82077957	rs142877367	R	I	0.82	3.47E-01	0.2663	0.2826	6.73E-01	0.1420	0.3352	3.64E-05	-1.0791	0.2409
2:82078983	rs62149768	T	A	0.85	1.95E-01	0.3777	0.2902	8.72E-01	0.0545	0.3329	3.43E-05	-1.1188	0.2488
2:82085610	rs62149770	G	A	0.85	1.96E-01	0.3781	0.2907	8.69E-01	0.0558	0.3332	3.38E-05	-1.1215	0.2491
2:82088076	rs7602295	G	A	0.82	3.48E-01	0.2657	0.2826	6.69E-01	0.1439	0.3351	3.76E-05	-1.0773	0.2410
2:82088565	rs10496253	T	C	0.85	1.97E-01	0.3764	0.2902	8.68E-01	0.0563	0.3330	3.55E-05	-1.1170	0.2489
2:82093632	rs17021075	A	C	0.85	1.97E-01	0.3761	0.2901	8.69E-01	0.0560	0.3329	3.57E-05	-1.1163	0.2489
2:82094735	rs62149771	A	G	0.85	1.97E-01	0.3762	0.2902	8.69E-01	0.0560	0.3330	3.57E-05	-1.1165	0.2489
2:82106093	rs10520309	G	A	0.82	3.42E-01	0.2690	0.2828	6.73E-01	0.1421	0.3353	3.67E-05	-1.0794	0.2411
2:82116415	rs17021134	A	G	0.85	1.97E-01	0.3767	0.2903	8.68E-01	0.0564	0.3332	3.60E-05	-1.1168	0.2491
2:82149016	rs62149794	C	G	0.85	1.97E-01	0.3769	0.2904	8.67E-01	0.0566	0.3333	3.61E-05	-1.1169	0.2492
2:82149115	rs62149795	A	G	0.85	1.83E-01	0.3892	0.2908	8.83E-01	0.0500	0.3337	2.94E-05	-1.1304	0.2489
2:82150948	rs7571397	T	C	0.82	3.01E-01	0.2907	0.2802	7.23E-01	0.1192	0.3339	3.04E-05	-1.0809	0.2385
2:82162243	rs62149798	T	C	0.85	1.81E-01	0.3918	0.2913	8.74E-01	0.0538	0.3341	3.08E-05	-1.1300	0.2496
2:82170774	rs10520307	T	C	0.85	1.80E-01	0.3934	0.2916	8.66E-01	0.0574	0.3343	3.23E-05	-1.1289	0.2500
2:82171820	rs62149799	A	G	0.85	1.80E-01	0.3936	0.2917	8.65E-01	0.0581	0.3344	3.26E-05	-1.1287	0.2501
2:82172681	rs141430405	T	G	0.85	1.80E-01	0.3938	0.2917	8.64E-01	0.0584	0.3344	3.28E-05	-1.1285	0.2502
2:82173337	rs62149800	T	C	0.85	1.80E-01	0.3939	0.2918	8.63E-01	0.0587	0.3344	3.29E-05	-1.1284	0.2502
2:82173740	rs62149801	G	A	0.85	1.80E-01	0.3939	0.2917	8.63E-01	0.0589	0.3344	3.31E-05	-1.1281	0.2502
2:82176951	rs78268192	C	T	0.85	1.79E-01	0.3948	0.2919	8.58E-01	0.0609	0.3345	3.41E-05	-1.1272	0.2505
2:82180200	rs62149819	A	G	0.85	1.78E-01	0.3955	0.2920	8.54E-01	0.0626	0.3345	3.50E-05	-1.1262	0.2507
2:82180329	rs77222195	R	D	0.85	1.78E-01	0.3956	0.2921	8.54E-01	0.0627	0.3346	3.50E-05	-1.1264	0.2508
2:82180481	rs45552344	T	C	0.85	1.78E-01	0.3955	0.2920	8.54E-01	0.0625	0.3346	3.48E-05	-1.1266	0.2507

2:82183544	rs62149821	G	A	0.85	1.78E-01	0.3960	0.2921	8.52E-01	0.0637	0.3345	3.57E-05	-1.1254	0.2509
2:82184724	rs10496249	T	C	0.85	1.78E-01	0.3962	0.2921	8.51E-01	0.0641	0.3345	3.59E-05	-1.1252	0.2509
2:82185174	rs62149822	C	T	0.85	1.78E-01	0.3962	0.2921	8.50E-01	0.0643	0.3345	3.60E-05	-1.1251	0.2509
2:82186981	rs75258287	R	D	0.85	1.77E-01	0.3965	0.2921	8.49E-01	0.0650	0.3346	3.65E-05	-1.1245	0.2510
2:82189438	rs62149824	C	A	0.85	1.77E-01	0.3969	0.2922	8.46E-01	0.0663	0.3346	3.73E-05	-1.1234	0.2512
2:82195358	rs995108	T	C	0.85	1.76E-01	0.3977	0.2922	8.39E-01	0.0694	0.3346	3.93E-05	-1.1211	0.2515
2:82200967	rs62149834	A	G	0.85	1.74E-01	0.4003	0.2924	8.33E-01	0.0718	0.3347	3.93E-05	-1.1220	0.2517
2:82206093	rs17021275	A	C	0.85	1.81E-01	0.3941	0.2930	8.28E-01	0.0740	0.3346	3.74E-05	-1.1264	0.2518
2:82207758	rs17021281	G	A	0.85	1.70E-01	0.4029	0.2919	8.24E-01	0.0755	0.3341	4.64E-05	-1.1110	0.2519
2:83572567	rs79960757	G	A	0.95	4.64E-05	1.8229	0.4160	4.81E-01	-0.4167	0.5804	5.22E-01	-0.3042	0.4720
2:8790973	rs3889716	C	T	0.88	2.16E-01	0.4017	0.3231	6.25E-01	-0.2246	0.4590	6.16E-05	-1.2088	0.2794
3:100187582	rs35558966	I	R	0.48	1.07E-01	0.3119	0.1916	7.86E-02	-0.4026	0.2192	5.08E-05	-0.7307	0.1667
3:100193722	rs7426388	G	A	0.48	1.13E-01	0.3052	0.1908	7.86E-02	-0.4017	0.2190	5.08E-05	-0.7273	0.1659
3:100193743	rs7426390	G	A	0.48	1.09E-01	0.3089	0.1908	7.79E-02	-0.4022	0.2188	4.58E-05	-0.7312	0.1657
3:100194134	rs149741858	G	A	0.48	1.09E-01	0.3086	0.1907	7.79E-02	-0.4020	0.2187	4.54E-05	-0.7310	0.1655
3:100196539	rs76717130	C	G	0.48	1.11E-01	0.3057	0.1902	7.67E-02	-0.4027	0.2184	4.67E-05	-0.7282	0.1652
3:10379194	rs76596509	T	G	0.78	2.71E-02	0.7554	0.3351	9.65E-05	-1.3819	0.3380	3.29E-01	-0.3360	0.3411
3:10379195	rs75647954	C	T	0.78	2.71E-02	0.7553	0.3350	9.66E-05	-1.3818	0.3380	3.29E-01	-0.3361	0.3411
3:10379196	rs77630473	G	C	0.78	2.65E-02	0.7590	0.3354	9.58E-05	-1.3854	0.3388	3.23E-01	-0.3407	0.3415
3:118551325	rs73177871	A	T	0.88	2.42E-05	-1.3924	0.3047	5.76E-03	1.6215	0.6624	3.65E-01	0.3180	0.3483
3:118553229	rs56230528	C	T	0.88	2.48E-05	-1.3842	0.3034	5.79E-03	1.6069	0.6565	3.80E-01	0.3071	0.3468
3:118554583	rs1402582	G	A	0.88	2.79E-05	-1.3526	0.2987	6.69E-03	1.5431	0.6373	4.27E-01	0.2729	0.3412
3:118557259	rs73177882	C	A	0.88	3.41E-05	-1.3206	0.2954	7.79E-03	1.4784	0.6198	4.48E-01	0.2571	0.3364
3:118557719	rs73177884	A	G	0.88	3.45E-05	-1.3193	0.2953	7.90E-03	1.4744	0.6191	4.48E-01	0.2568	0.3363
3:118557856	rs76764161	G	C	0.88	3.46E-05	-1.3184	0.2952	7.86E-03	1.4744	0.6187	4.49E-01	0.2561	0.3361
3:118559664	rs149005255	R	D	0.88	3.68E-05	-1.3124	0.2950	8.20E-03	1.4605	0.6156	4.55E-01	0.2526	0.3356
3:118560824	rs73177893	A	G	0.88	3.74E-05	-1.3081	0.2943	8.30E-03	1.4533	0.6134	4.56E-01	0.2511	0.3348
3:118565544	rs60216851	C	A	0.88	4.81E-05	-1.2733	0.2912	9.59E-03	1.3938	0.5979	4.87E-01	0.2310	0.3301
3:118565657	rs58062630	T	C	0.88	4.79E-05	-1.2679	0.2899	9.49E-03	1.3897	0.5954	4.92E-01	0.2275	0.3286
3:118565659	rs58394464	T	C	0.88	4.80E-05	-1.2678	0.2900	9.50E-03	1.3894	0.5953	4.92E-01	0.2274	0.3286
3:119278540	rs16829984	G	C	0.90	4.20E-05	1.5813	0.3585	9.54E-01	-0.0271	0.4718	8.39E-01	-0.0834	0.4088
3:119289916	rs7617301	A	C	0.91	1.97E-05	1.6578	0.3583	7.15E-01	-0.1764	0.4818	3.86E-01	-0.3594	0.4112
3:13695824	rs6790948	T	C	0.92	2.27E-01	-0.4449	0.3663	1.07E-01	0.7475	0.4738	4.87E-05	1.3860	0.3153
3:13696621	rs9812762	C	T	0.92	2.46E-01	-0.4229	0.3628	1.24E-01	0.7040	0.4659	3.99E-05	1.3846	0.3109

3:13702407	rs60376071	G	A	0.92	2.35E-01	-0.4317	0.3622	1.23E-01	0.7054	0.4660	4.33E-05	1.3776	0.3110
3:14883570	rs55751892	A	G	0.82	2.04E-05	-1.0282	0.2227	1.54E-01	0.4921	0.3549	3.07E-01	0.2627	0.2548
3:14893803	rs1689529	G	A	0.82	2.10E-05	-1.0243	0.2222	1.53E-01	0.4920	0.3544	3.16E-01	0.2570	0.2542
3:14898156	rs11294286	R	D	0.81	2.08E-05	-1.0311	0.2236	1.05E-01	0.5657	0.3612	1.76E-01	0.3477	0.2540
3:14902315	rs34317529	R	I	0.83	2.30E-05	-1.0532	0.2298	1.40E-01	0.5266	0.3679	2.53E-01	0.3024	0.2618
3:14903150	rs1689576	T	C	0.82	2.31E-05	-1.0171	0.2220	1.49E-01	0.4953	0.3541	3.31E-01	0.2489	0.2537
3:14903536	rs294629	A	G	0.82	2.23E-05	-1.0169	0.2215	1.48E-01	0.4967	0.3537	3.31E-01	0.2482	0.2532
3:14911736	rs1689571	A	G	0.82	2.58E-05	-1.0093	0.2218	1.52E-01	0.4903	0.3530	3.47E-01	0.2402	0.2531
3:14911808	rs1687290	C	T	0.82	2.59E-05	-1.0092	0.2218	1.52E-01	0.4905	0.3530	3.47E-01	0.2402	0.2531
3:14912169	rs1656441	C	T	0.82	2.59E-05	-1.0089	0.2218	1.52E-01	0.4903	0.3529	3.47E-01	0.2397	0.2531
3:14912310	rs1689570	A	G	0.82	2.60E-05	-1.0089	0.2218	1.52E-01	0.4901	0.3529	3.48E-01	0.2395	0.2531
3:14912402	rs1687291	G	A	0.82	2.60E-05	-1.0088	0.2218	1.52E-01	0.4902	0.3529	3.48E-01	0.2396	0.2531
3:14913294	rs1622485	A	T	0.82	2.62E-05	-1.0082	0.2218	1.53E-01	0.4898	0.3528	3.49E-01	0.2390	0.2530
3:14913407	rs1687292	G	T	0.82	2.62E-05	-1.0082	0.2218	1.53E-01	0.4898	0.3528	3.49E-01	0.2390	0.2530
3:14913548	rs1656440	C	T	0.82	2.63E-05	-1.0080	0.2218	1.53E-01	0.4897	0.3528	3.49E-01	0.2388	0.2530
3:14913756	rs536984778	R	D	0.82	2.71E-05	-1.0085	0.2223	1.61E-01	0.4810	0.3526	3.51E-01	0.2383	0.2535
3:14913815	rs1656439	T	G	0.82	2.63E-05	-1.0079	0.2218	1.53E-01	0.4896	0.3528	3.50E-01	0.2387	0.2530
3:14913882	rs1689569	A	G	0.82	2.63E-05	-1.0080	0.2218	1.53E-01	0.4897	0.3528	3.50E-01	0.2386	0.2530
3:14914149	rs1656438	G	T	0.82	2.63E-05	-1.0091	0.2220	1.51E-01	0.4920	0.3534	3.46E-01	0.2406	0.2533
3:14914212	rs1656437	T	A	0.82	2.84E-05	-1.0074	0.2227	1.69E-01	0.4718	0.3523	3.55E-01	0.2366	0.2538
3:14914385	rs1625709	C	T	0.82	2.83E-05	-1.0076	0.2227	1.69E-01	0.4716	0.3524	3.56E-01	0.2364	0.2538
3:14914409	rs1602297	G	A	0.82	2.62E-05	-1.0088	0.2219	1.52E-01	0.4901	0.3530	3.52E-01	0.2378	0.2532
3:14914578	rs1602298	A	G	0.82	2.62E-05	-1.0088	0.2219	1.53E-01	0.4899	0.3530	3.52E-01	0.2377	0.2532
3:14914657	rs1602299	G	A	0.82	2.62E-05	-1.0089	0.2219	1.53E-01	0.4900	0.3530	3.52E-01	0.2377	0.2532
3:14914699	rs1602300	A	G	0.82	2.62E-05	-1.0093	0.2220	1.53E-01	0.4902	0.3531	3.52E-01	0.2377	0.2533
3:14914965	rs17040460	A	G	0.82	2.61E-05	-1.0095	0.2220	1.52E-01	0.4904	0.3531	3.53E-01	0.2373	0.2534
3:14915046	rs1627256	C	A	0.82	2.62E-05	-1.0095	0.2220	1.53E-01	0.4901	0.3531	3.53E-01	0.2372	0.2534
3:14915088	rs1627146	T	G	0.82	2.61E-05	-1.0096	0.2220	1.53E-01	0.4902	0.3531	3.53E-01	0.2372	0.2534
3:14915131	rs1631793	C	T	0.82	2.61E-05	-1.0097	0.2221	1.53E-01	0.4903	0.3532	3.54E-01	0.2370	0.2534
3:14915132	rs1626374	A	G	0.82	2.61E-05	-1.0099	0.2221	1.52E-01	0.4904	0.3532	3.54E-01	0.2370	0.2534
3:14915512	rs2636753	A	G	0.82	2.61E-05	-1.0104	0.2222	1.52E-01	0.4906	0.3533	3.55E-01	0.2364	0.2536
3:14915532	rs2729696	C	T	0.82	2.61E-05	-1.0105	0.2222	1.52E-01	0.4908	0.3534	3.55E-01	0.2364	0.2536
3:14915596	rs113459364	R	D	0.82	2.60E-05	-1.0107	0.2222	1.52E-01	0.4909	0.3534	3.56E-01	0.2362	0.2536
3:14915600	rs201570736	A	G	0.82	2.60E-05	-1.0107	0.2222	1.52E-01	0.4907	0.3534	3.56E-01	0.2361	0.2536

3:14915662	rs1656434	G	A	0.82	2.61E-05	-1.0108	0.2223	1.52E-01	0.4909	0.3534	3.56E-01	0.2359	0.2537
3:14915673	rs1687293	G	A	0.82	2.60E-05	-1.0109	0.2223	1.52E-01	0.4910	0.3534	3.56E-01	0.2358	0.2537
3:14916048	rs9822464	T	C	0.82	2.81E-05	-1.0099	0.2231	1.68E-01	0.4739	0.3530	3.63E-01	0.2331	0.2544
3:14916684	rs1656433	C	T	0.82	2.59E-05	-1.0123	0.2225	1.52E-01	0.4916	0.3538	3.59E-01	0.2348	0.2540
3:14916813	rs1656432	T	C	0.82	2.72E-05	-1.0108	0.2228	1.51E-01	0.4937	0.3540	3.60E-01	0.2347	0.2542
3:14916830	rs75182489	R	D	0.82	2.58E-05	-1.0125	0.2225	1.52E-01	0.4917	0.3538	3.60E-01	0.2346	0.2540
3:14916858	rs1656431	C	T	0.82	2.59E-05	-1.0125	0.2226	1.52E-01	0.4920	0.3539	3.60E-01	0.2343	0.2541
3:14918986	rs293918	G	A	0.82	2.56E-05	-1.0156	0.2230	1.51E-01	0.4937	0.3546	3.67E-01	0.2314	0.2547
3:14919254	rs293919	A	G	0.82	2.61E-05	-1.0159	0.2234	1.59E-01	0.4846	0.3542	3.71E-01	0.2302	0.2551
3:14919352	rs293920	A	G	0.82	2.62E-05	-1.0157	0.2234	1.59E-01	0.4851	0.3543	3.71E-01	0.2302	0.2551
3:14919378	rs293921	A	G	0.82	2.62E-05	-1.0158	0.2234	1.59E-01	0.4849	0.3543	3.71E-01	0.2300	0.2551
3:14919996	rs293922	C	T	0.82	2.54E-05	-1.0166	0.2232	1.51E-01	0.4942	0.3548	3.71E-01	0.2300	0.2550
3:14920010	rs293923	G	T	0.82	2.54E-05	-1.0167	0.2232	1.51E-01	0.4943	0.3548	3.71E-01	0.2299	0.2550
3:14920105	rs293924	G	A	0.82	2.46E-05	-1.0171	0.2228	1.50E-01	0.4953	0.3545	3.71E-01	0.2294	0.2547
3:14924204	rs140724200	R	D	0.72	7.63E-05	-1.0265	0.2422	6.93E-01	0.1302	0.3315	1.65E-01	0.3780	0.2690
3:151057219	rs10645486	I	R	0.82	4.82E-02	0.5007	0.2496	2.74E-02	-0.6353	0.2769	4.90E-05	-0.9646	0.2195
3:169946404	rs142544612	R	I	0.85	6.73E-01	0.1578	0.3739	9.61E-01	-0.0233	0.4638	9.98E-05	1.3478	0.3221
3:169966168	rs145718729	D	R	0.47	8.22E-01	0.0530	0.2364	2.20E-01	-0.3385	0.2795	2.04E-05	-0.9202	0.1980
3:169968220	rs141812733	R	D	0.42	7.74E-01	0.0722	0.2523	4.20E-01	-0.2414	0.3026	1.14E-05	-1.0072	0.2093
3:169980956	N/A	R	D	0.51	4.14E-01	-0.1698	0.2078	8.06E-01	-0.0802	0.3139	1.45E-01	-0.2981	0.2017
3:187652956	rs138458084	I	R	0.78	6.09E-05	0.9351	0.2173	2.10E-02	-0.6497	0.2679	4.72E-01	-0.1777	0.2452
3:20076191	rs1915924	C	G	0.72	2.73E-01	-0.2519	0.2288	7.63E-01	-0.0807	0.2651	3.32E-05	0.8800	0.1953
3:20077262	rs35747495	C	T	0.72	2.70E-01	-0.2516	0.2274	7.46E-01	-0.0861	0.2634	4.10E-05	0.8656	0.1947
3:20079878	rs12639078	A	G	0.72	2.83E-01	-0.2420	0.2245	6.97E-01	-0.1015	0.2586	5.23E-05	0.8442	0.1930
3:20479899	rs17007012	A	G	0.82	9.76E-05	0.9656	0.2318	4.82E-01	0.2293	0.3306	2.92E-01	-0.2744	0.2582
3:20480253	rs12492284	A	G	0.82	9.74E-05	0.9657	0.2318	4.82E-01	0.2293	0.3306	2.93E-01	-0.2742	0.2582
3:20482674	rs13347246	G	A	0.83	9.58E-05	0.9664	0.2317	4.81E-01	0.2296	0.3305	2.96E-01	-0.2722	0.2582
3:20483056	rs13347248	C	A	0.83	9.57E-05	0.9664	0.2317	4.81E-01	0.2296	0.3305	2.97E-01	-0.2720	0.2582
3:20483808	rs12488716	C	A	0.83	8.82E-05	0.9820	0.2341	4.66E-01	0.2427	0.3365	3.17E-01	-0.2637	0.2614
3:20487081	rs2366076	A	G	0.83	9.31E-05	0.9673	0.2315	4.79E-01	0.2305	0.3304	3.06E-01	-0.2669	0.2581
3:20489192	rs35688043	G	T	0.83	9.21E-05	0.9676	0.2314	4.79E-01	0.2306	0.3303	3.09E-01	-0.2650	0.2581
3:20495570	rs59608357	T	A	0.83	8.81E-05	0.9694	0.2311	4.78E-01	0.2308	0.3301	3.18E-01	-0.2600	0.2581
3:20496715	rs2203934	C	G	0.83	8.93E-05	0.9682	0.2310	4.77E-01	0.2313	0.3299	3.21E-01	-0.2581	0.2579
3:20497659	rs6550523	G	A	0.83	8.91E-05	0.9681	0.2309	4.77E-01	0.2314	0.3299	3.23E-01	-0.2572	0.2579

3:20497740	rs6550524	G	A	0.83	8.90E-05	0.9681	0.2309	4.77E-01	0.2314	0.3299	3.23E-01	-0.2571	0.2579
3:20497835	rs10510502	A	G	0.83	8.90E-05	0.9681	0.2309	4.77E-01	0.2313	0.3298	3.23E-01	-0.2571	0.2579
3:20499291	rs60773738	G	A	0.82	7.02E-05	0.9855	0.2312	5.00E-01	0.2203	0.3310	3.91E-01	-0.2247	0.2598
3:20499492	rs145623105	G	A	0.83	8.62E-05	0.9696	0.2308	4.76E-01	0.2315	0.3297	3.26E-01	-0.2556	0.2579
3:20501936	rs12495532	C	T	0.83	8.75E-05	0.9680	0.2306	4.76E-01	0.2315	0.3295	3.30E-01	-0.2532	0.2577
3:20502704	rs10716124	R	D	0.83	8.71E-05	0.9680	0.2306	4.76E-01	0.2315	0.3295	3.31E-01	-0.2525	0.2576
3:20503129	rs6762123	G	A	0.83	8.91E-05	0.9690	0.2312	4.69E-01	0.2356	0.3303	3.42E-01	-0.2476	0.2583
3:20503477	rs5847054	R	D	0.57	4.83E-01	0.1287	0.1834	4.02E-02	-0.4429	0.2124	1.98E-01	-0.3296	0.2528
3:20503600	rs61526106	C	T	0.83	8.70E-05	0.9680	0.2305	4.76E-01	0.2315	0.3294	3.32E-01	-0.2521	0.2576
3:20503795	rs17007057	A	C	0.83	8.72E-05	0.9679	0.2305	4.76E-01	0.2316	0.3295	3.32E-01	-0.2520	0.2576
3:20503821	rs57344311	G	A	0.83	8.72E-05	0.9679	0.2305	4.76E-01	0.2316	0.3295	3.32E-01	-0.2520	0.2576
3:20503859	rs60145530	R	D	0.83	8.72E-05	0.9679	0.2305	4.76E-01	0.2316	0.3295	3.32E-01	-0.2520	0.2576
3:20504026	rs17007063	T	C	0.83	8.57E-05	0.9680	0.2303	4.74E-01	0.2324	0.3293	3.32E-01	-0.2520	0.2574
3:20504066	rs59264312	A	G	0.83	8.51E-05	0.9686	0.2303	4.77E-01	0.2309	0.3293	3.32E-01	-0.2519	0.2575
3:20504149	rs56332973	R	D	0.83	8.88E-05	0.9724	0.2319	4.69E-01	0.2362	0.3310	2.84E-01	-0.2794	0.2586
3:20504714	rs6808521	T	A	0.83	8.72E-05	0.9679	0.2305	4.76E-01	0.2316	0.3295	3.32E-01	-0.2520	0.2576
3:20504881	rs73187189	G	A	0.83	8.73E-05	0.9679	0.2306	4.76E-01	0.2317	0.3295	3.32E-01	-0.2520	0.2577
3:20504883	rs76205109	T	C	0.83	8.73E-05	0.9679	0.2306	4.76E-01	0.2317	0.3295	3.32E-01	-0.2520	0.2577
3:20505047	rs6808851	T	C	0.83	8.73E-05	0.9679	0.2306	4.76E-01	0.2317	0.3295	3.32E-01	-0.2520	0.2577
3:20505156	rs6808959	T	C	0.83	8.73E-05	0.9679	0.2306	4.76E-01	0.2317	0.3295	3.32E-01	-0.2520	0.2577
3:20505431	rs6809254	T	C	0.83	8.72E-05	0.9679	0.2305	4.76E-01	0.2317	0.3295	3.32E-01	-0.2520	0.2576
3:20505603	rs6794717	C	T	0.83	8.72E-05	0.9679	0.2306	4.75E-01	0.2318	0.3295	3.32E-01	-0.2519	0.2577
3:20527133	rs648238	A	G	0.83	8.72E-05	0.9679	0.2306	4.76E-01	0.2316	0.3295	3.32E-01	-0.2519	0.2577
3:20527529	rs646454	T	C	0.83	8.72E-05	0.9679	0.2305	4.76E-01	0.2315	0.3294	3.32E-01	-0.2520	0.2576
3:36711297	rs9869380	G	T	0.52	3.61E-06	0.9182	0.1801	3.04E-01	-0.2539	0.2459	2.18E-01	-0.2632	0.2113
3:36740273	rs10865875	G	A	0.52	8.40E-05	-0.7592	0.1804	6.67E-01	0.0996	0.2229	3.02E-01	0.2101	0.2015
3:5568521	rs202109419	T	C	0.94	5.90E-05	-1.6399	0.3803	1.27E-02	4.3007	2.9213	7.52E-01	0.1369	0.4309
3:5568636	rs202024380	T	A	0.93	7.00E-05	-1.5395	0.3612	1.09E-02	3.7504	2.3215	8.85E-01	0.0592	0.4084
3:5568937	rs200425927	G	A	0.93	8.27E-05	-1.5239	0.3616	7.66E-03	3.8386	2.2293	8.94E-01	0.0548	0.4078
3:5569086	rs112583124	G	A	0.93	6.02E-05	-1.5355	0.3565	8.01E-03	3.8428	2.2509	9.13E-01	0.0442	0.4042
3:5569231	rs141985842	G	A	0.93	6.03E-05	-1.5346	0.3564	8.17E-03	3.8150	2.2369	9.18E-01	0.0416	0.4040
3:5569301	rs145943875	C	G	0.93	6.01E-05	-1.5353	0.3565	8.18E-03	3.8180	2.2392	9.18E-01	0.0419	0.4042
3:5569429	rs200802860	R	D	0.93	7.64E-05	-1.5064	0.3555	7.00E-03	3.8075	2.1828	9.02E-01	0.0496	0.4014
3:5571785	rs148415352	A	G	0.91	2.15E-05	-1.8622	0.4047	1.78E-02	2.9725	1.6281	7.36E-01	0.1577	0.4664

3:63319012	rs545756228	R	I	0.88	2.37E-02	0.6596	0.2855	2.68E-01	-0.4160	0.3629	5.54E-05	-1.1086	0.2544
3:82769356	rs112063763	C	G	0.63	8.17E-01	-0.0598	0.2582	4.65E-01	-0.2055	0.2830	4.89E-05	-0.9648	0.2195
3:89081611	rs138754151	R	I	0.64	1.63E-01	0.4132	0.2944	3.93E-01	-0.3153	0.3670	5.03E-06	-1.2345	0.2450
3:8912559	rs6798498	C	T	0.89	5.76E-01	-0.1827	0.3270	7.25E-05	1.8677	0.5384	9.21E-01	-0.0323	0.3224
3:8912579	rs6773293	G	C	0.93	6.32E-01	0.1890	0.3953	7.60E-05	2.2387	0.6519	7.10E-01	0.1455	0.3890
3:8913024	rs150747738	R	I	0.91	6.02E-01	-0.1892	0.3634	2.00E-05	2.3622	0.6482	4.09E-01	0.2960	0.3560
3:8928891	rs34003290	A	C	0.87	6.44E-01	-0.1437	0.3115	8.51E-05	1.6907	0.4907	5.42E-01	0.1876	0.3058
4:101922250	rs61482615	R	D	0.61	7.21E-05	-0.7040	0.1655	6.85E-02	0.4556	0.2521	2.99E-02	0.3997	0.1794
4:109600898	rs2851358	A	G	0.41	1.47E-01	-0.2823	0.1932	7.74E-01	0.0660	0.2279	7.12E-05	0.7210	0.1683
4:109610141	rs2575635	T	C	0.59	1.49E-01	0.2809	0.1932	7.82E-01	-0.0638	0.2277	7.17E-05	-0.7209	0.1683
4:109611234	rs6832162	C	T	0.82	4.72E-02	0.5110	0.2536	4.00E-01	-0.2598	0.3113	6.92E-05	-0.9634	0.2244
4:109612784	rs144247702	R	I	0.49	2.30E-01	-0.2540	0.2109	9.80E-01	0.0061	0.2434	6.48E-05	0.7865	0.1824
4:109612900	rs7687338	C	A	0.59	1.39E-01	0.2880	0.1930	7.83E-01	-0.0633	0.2275	6.74E-05	-0.7232	0.1682
4:109612903	rs7665650	G	T	0.59	1.39E-01	0.2881	0.1931	7.83E-01	-0.0633	0.2275	6.73E-05	-0.7233	0.1682
4:109612910	rs7687346	G	A	0.59	1.40E-01	0.2869	0.1931	7.81E-01	-0.0639	0.2275	6.74E-05	-0.7233	0.1682
4:109612966	rs200030909	I	R	0.59	1.43E-01	0.2852	0.1932	7.91E-01	-0.0608	0.2272	6.90E-05	-0.7226	0.1683
4:109613591	rs146337048	D	R	0.59	1.38E-01	0.2882	0.1929	7.85E-01	-0.0627	0.2272	6.67E-05	-0.7232	0.1681
4:109614319	rs2082966	G	A	0.59	1.38E-01	0.2883	0.1930	7.84E-01	-0.0629	0.2272	6.68E-05	-0.7233	0.1681
4:109619199	rs199721649	R	D	0.75	8.32E-02	0.4055	0.2314	4.46E-01	-0.2140	0.2848	5.74E-05	-0.8804	0.2025
4:109621884	rs2077246	A	G	0.59	1.34E-01	0.2912	0.1927	7.87E-01	-0.0619	0.2269	6.47E-05	-0.7239	0.1679
4:109627964	rs2575617	A	G	0.59	1.29E-01	0.2958	0.1933	7.91E-01	-0.0607	0.2273	6.50E-05	-0.7261	0.1684
4:110569850	rs58722526	R	D	0.75	4.92E-02	0.5505	0.2757	4.42E-01	-0.2703	0.3460	6.19E-06	-1.1664	0.2341
4:127692720	rs12647245	A	G	0.68	6.08E-01	0.1136	0.2214	6.60E-05	-0.9071	0.2144	1.91E-01	-0.2841	0.2149
4:134467690	rs568971068	R	D	0.90	9.63E-05	1.8105	0.4343	6.49E-02	1.4628	0.8712	4.96E-02	-0.9471	0.4722
4:14925721	rs71597565	C	G	0.75	1.57E-01	-0.3210	0.2253	1.92E-01	0.3544	0.2732	4.60E-05	0.8589	0.1946
4:14933392	rs35039217	A	G	0.78	2.90E-02	-0.4908	0.2205	2.83E-01	0.2955	0.2761	5.05E-05	0.8576	0.1956
4:14939389	rs34479500	G	A	0.78	2.95E-02	-0.4967	0.2238	2.62E-01	0.3130	0.2803	4.98E-05	0.8708	0.1984
4:14948645	rs33979623	C	G	0.78	3.15E-02	-0.4958	0.2262	5.02E-01	0.1993	0.2905	6.85E-05	0.8652	0.2014
4:14986918	rs71648191	R	D	0.73	1.49E-01	-0.3118	0.2146	7.04E-02	0.4982	0.2744	5.30E-06	0.8988	0.1789
4:15036835	rs71648192	R	I	0.78	2.15E-01	-0.3095	0.2487	6.56E-02	0.6497	0.3519	4.01E-06	1.0477	0.2053
4:156616621	rs7688323	A	G	0.82	9.74E-03	0.5690	0.2141	6.51E-01	-0.1213	0.2656	8.48E-05	-0.8246	0.1948
4:156620217	rs72685791	G	A	0.82	1.03E-02	0.5621	0.2132	6.42E-01	-0.1238	0.2641	8.99E-05	-0.8178	0.1940
4:156628756	rs568661109	R	I	0.79	5.63E-03	0.6083	0.2128	5.19E-01	-0.1638	0.2500	2.72E-05	-0.8744	0.1916
4:157604689	rs66885035	R	D	0.79	7.97E-05	-1.0977	0.2598	9.63E-02	0.6165	0.3742	1.79E-02	0.6805	0.2790

4:168802014	rs7678715	T	C	0.76	2.32E-01	0.2940	0.2451	3.97E-01	-0.2337	0.2759	7.39E-05	-0.9078	0.2124
4:172139320	rs11378625	I	R	0.73	7.00E-05	0.9762	0.2290	9.94E-01	0.0026	0.3322	5.65E-01	-0.1496	0.2583
4:185156234	rs13137454	G	A	0.53	5.60E-05	0.7456	0.1723	2.30E-01	-0.2681	0.2213	1.30E-01	-0.2945	0.1917
4:185156248	rs371049171	C	A	0.57	3.41E-05	0.8263	0.1848	1.06E-01	-0.3878	0.2366	6.53E-02	-0.3858	0.2053
4:185156258	rs72699758	A	G	0.56	2.41E-05	0.8196	0.1793	1.60E-01	-0.3310	0.2336	6.00E-02	-0.3840	0.2001
4:185156853	rs13119105	T	G	0.52	2.27E-05	0.7594	0.1655	2.26E-01	-0.2665	0.2186	8.86E-02	-0.3222	0.1860
4:185159929	rs55799474	C	T	0.56	3.81E-05	0.7511	0.1692	1.39E-01	-0.3246	0.2116	1.15E-01	-0.3028	0.1891
4:188072816	rs78740791	C	G	0.81	1.68E-01	-0.3802	0.2737	2.18E-05	1.8094	0.5079	1.83E-01	0.3632	0.2694
4:188072863	rs77449565	T	C	0.81	1.68E-01	-0.3802	0.2737	2.18E-05	1.8094	0.5079	1.83E-01	0.3632	0.2694
4:188073063	rs78937528	T	C	0.81	1.68E-01	-0.3802	0.2737	2.18E-05	1.8094	0.5079	1.83E-01	0.3632	0.2694
4:188074208	rs113546188	G	A	0.81	1.56E-01	-0.3974	0.2784	2.57E-05	1.8287	0.5150	1.73E-01	0.3783	0.2741
4:188074282	rs111470397	G	A	0.81	1.57E-01	-0.3975	0.2786	2.58E-05	1.8295	0.5152	1.73E-01	0.3786	0.2743
4:188074434	rs113298113	G	C	0.81	1.56E-01	-0.3998	0.2795	3.16E-05	1.8035	0.5100	1.67E-01	0.3852	0.2751
4:188078040	rs36047796	D	R	0.79	6.02E-02	-0.5550	0.2913	1.31E-05	2.0310	0.5386	4.71E-01	0.2133	0.2942
4:21453446	rs168583	A	G	0.61	3.03E-02	0.4463	0.2021	3.18E-02	-0.4979	0.2319	4.59E-05	-0.7893	0.1789
4:22165635	rs6847773	G	C	0.40	7.34E-05	0.7688	0.1809	1.90E-02	-0.6341	0.2528	8.97E-02	-0.3441	0.1993
4:29533219	rs143880255	R	I	0.61	8.10E-01	-0.0690	0.2872	7.85E-01	0.1020	0.3784	2.64E-05	1.1049	0.2416
4:31358599	rs73125237	A	G	0.85	8.90E-05	-1.0011	0.2388	9.64E-02	0.6563	0.4159	1.92E-01	0.3502	0.2650
4:4739625	rs4689929	G	T	0.72	1.49E-05	-0.8659	0.1840	6.22E-03	0.7248	0.2736	1.59E-02	0.5041	0.2029
4:4742547	rs10937890	T	A	0.73	5.76E-05	-0.8376	0.1939	8.06E-03	0.7167	0.2785	2.18E-02	0.4954	0.2100
4:54284448	rs34196016	R	I	0.66	1.78E-01	0.2618	0.1931	6.80E-01	-0.0933	0.2246	5.64E-05	-0.7274	0.1671
4:54294762	rs71197022	R	D	0.70	9.46E-02	0.3327	0.1969	4.07E-01	-0.1974	0.2360	5.67E-05	-0.7483	0.1720
4:54328522	rs35767390	R	I	0.66	1.69E-01	0.2647	0.1911	7.76E-01	-0.0638	0.2235	3.95E-05	-0.7334	0.1646
4:54354237	rs6852422	G	A	0.89	1.85E-01	0.4249	0.3190	9.46E-01	0.0243	0.3543	4.43E-05	-1.2156	0.2748
4:54354797	rs11728872	C	G	0.70	1.87E-01	0.2581	0.1946	9.46E-01	0.0159	0.2282	2.77E-05	-0.7584	0.1664
4:54369718	rs11934906	G	A	0.70	1.65E-01	0.2679	0.1918	8.81E-01	0.0345	0.2257	2.57E-05	-0.7512	0.1640
4:54377597	rs11935144	T	C	0.70	1.64E-01	0.2670	0.1906	8.68E-01	0.0382	0.2246	2.60E-05	-0.7461	0.1630
4:5757046	rs3774873	C	T	0.87	8.89E-05	1.2319	0.2938	7.37E-01	-0.1300	0.3897	4.47E-01	-0.2522	0.3293
4:67751105	rs6552041	G	T	0.62	9.58E-05	-0.7891	0.1892	8.37E-01	0.0637	0.3064	4.83E-02	0.4151	0.2057
4:67753247	rs13125049	C	T	0.39	9.87E-05	0.7774	0.1868	9.06E-01	-0.0357	0.2980	5.35E-02	-0.4007	0.2032
4:67754775	rs7659876	G	C	0.61	9.71E-05	-0.7760	0.1862	9.05E-01	0.0358	0.2973	5.35E-02	0.3996	0.2027
4:67761511	rs2127947	G	T	0.39	9.74E-05	0.7648	0.1836	9.05E-01	-0.0355	0.2924	5.06E-02	-0.3988	0.1996
4:67770518	rs35299043	R	D	0.60	5.35E-05	-0.7800	0.1797	8.60E-01	0.0515	0.2883	3.84E-02	0.4165	0.1966
4:73253586	rs138949703	R	I	0.95	1.76E-01	-0.7356	0.5407	1.99E-01	0.9282	0.7465	8.14E-05	1.9992	0.4709

4:77868402	rs77308699	C	A	0.94	5.40E-05	1.4303	0.3297	4.44E-01	-0.3634	0.4645	2.50E-01	-0.4298	0.3702
4:77875918	rs77580140	C	T	0.94	5.06E-05	1.3752	0.3156	4.42E-01	-0.3501	0.4460	2.20E-01	-0.4396	0.3542
4:77892042	rs7674825	C	T	0.94	4.99E-05	1.3655	0.3131	4.44E-01	-0.3465	0.4427	2.19E-01	-0.4366	0.3514
4:77957813	rs1127583	T	A	0.93	8.27E-05	1.3662	0.3242	4.64E-01	-0.3396	0.4558	3.06E-01	-0.3743	0.3623
4:77965703	rs139528119	R	D	0.94	7.14E-05	1.3835	0.3250	4.27E-01	-0.3686	0.4539	2.69E-01	-0.4058	0.3635
4:77978180	rs4252883	G	C	0.94	7.53E-05	1.3894	0.3276	4.20E-01	-0.3761	0.4562	2.70E-01	-0.4074	0.3661
4:78028143	rs78721254	C	T	0.94	9.13E-05	1.4759	0.3527	3.91E-01	-0.4291	0.4869	2.82E-01	-0.4274	0.3931
4:78037888	rs79683636	G	A	0.94	9.16E-05	1.4786	0.3534	3.87E-01	-0.4329	0.4872	2.85E-01	-0.4254	0.3939
4:78038200	rs143992069	T	C	0.94	9.18E-05	1.4786	0.3535	3.87E-01	-0.4331	0.4872	2.85E-01	-0.4255	0.3940
4:78039036	rs7682171	C	A	0.94	9.16E-05	1.4785	0.3534	3.87E-01	-0.4332	0.4871	2.85E-01	-0.4253	0.3939
4:78040139	rs61287574	R	I	0.94	8.60E-05	1.4868	0.3538	3.86E-01	-0.4343	0.4872	2.86E-01	-0.4255	0.3948
4:78045463	rs75298026	C	T	0.94	9.15E-05	1.4807	0.3539	3.85E-01	-0.4350	0.4871	2.93E-01	-0.4192	0.3946
4:79014017	rs17418552	C	T	0.83	5.30E-05	-0.9770	0.2249	1.88E-02	0.8414	0.3799	2.43E-01	0.2982	0.2525
4:79053555	rs58118093	T	C	0.82	9.58E-05	-0.9566	0.2294	3.33E-02	0.7593	0.3754	2.91E-01	0.2726	0.2555
4:79066261	rs72654668	T	C	0.82	8.25E-05	-0.9814	0.2329	3.12E-02	0.7822	0.3825	2.67E-01	0.2911	0.2598
4:79067375	rs28542863	C	A	0.92	8.27E-01	-0.0820	0.3765	3.11E-02	0.7834	0.3828	2.66E-01	0.2923	0.2600
4:79073650	rs6848061	G	T	0.83	7.36E-05	-0.9949	0.2342	2.87E-02	0.8032	0.3874	2.53E-01	0.3022	0.2616
4:79075183	rs35967777	T	G	0.83	7.50E-05	-0.9972	0.2351	2.86E-02	0.8063	0.3885	2.52E-01	0.3040	0.2625
4:85560000	rs3840105	R	I	0.66	5.19E-01	0.1522	0.2362	2.30E-01	-0.3275	0.2739	7.02E-05	-0.8692	0.2027
4:8644328	rs62288627	C	T	0.93	6.11E-01	-0.2114	0.4160	8.18E-05	4.5078	2.1385	5.32E-01	0.2570	0.4085
5:122685727	rs1047437	C	G	0.84	3.85E-01	-0.1960	0.2255	3.71E-05	1.6418	0.4739	8.10E-02	0.3859	0.2172
5:122699812	rs12522139	T	G	0.84	4.37E-01	-0.1757	0.2261	5.53E-05	1.5492	0.4520	8.13E-02	0.3861	0.2176
5:122744178	rs13154356	T	C	0.85	3.39E-01	-0.2190	0.2284	5.34E-05	1.6647	0.4874	7.32E-02	0.4017	0.2201
5:122765884	rs4073780	T	G	0.85	2.41E-01	-0.2742	0.2328	8.31E-05	1.5868	0.4742	1.72E-01	0.3156	0.2279
5:122766724	rs146812994	G	A	0.85	2.41E-01	-0.2742	0.2329	8.31E-05	1.5831	0.4725	1.71E-01	0.3163	0.2279
5:122767863	rs12521390	G	T	0.85	2.43E-01	-0.2734	0.2330	8.26E-05	1.5823	0.4720	1.69E-01	0.3174	0.2280
5:122767933	rs12521417	G	C	0.85	2.43E-01	-0.2734	0.2330	8.27E-05	1.5818	0.4719	1.69E-01	0.3175	0.2281
5:122772373	rs8180503	C	G	0.85	2.30E-01	-0.2839	0.2356	8.49E-05	1.5848	0.4714	1.72E-01	0.3192	0.2308
5:122773605	rs13167307	A	G	0.85	2.31E-01	-0.2836	0.2356	8.36E-05	1.5858	0.4713	1.71E-01	0.3200	0.2308
5:134663997	rs6877719	A	G	0.93	4.86E-05	2.1092	0.4828	7.01E-01	-0.2793	0.7188	9.51E-01	-0.0342	0.5494
5:134664085	rs6877890	A	C	0.94	2.26E-05	2.2179	0.4835	3.90E-01	-0.6253	0.6998	9.20E-01	-0.0562	0.5572
5:141949123	rs11744900	A	G	0.85	9.35E-05	-1.1443	0.2739	7.56E-01	0.1207	0.3965	1.19E-01	0.4772	0.3017
5:145035449	rs200376997	R	I	0.92	3.03E-01	0.3522	0.3412	3.34E-01	-0.3733	0.3744	9.79E-05	-1.2406	0.2961
5:145083153	rs11741931	C	G	0.92	1.25E-01	0.5465	0.3531	1.94E-01	-0.5098	0.3726	4.71E-05	-1.3488	0.3062

5:145104494	rs72811547	T	C	0.92	1.04E-01	0.5784	0.3526	1.68E-01	-0.5404	0.3714	1.61E-05	-1.4187	0.3010
5:156111450	rs32074	T	C	0.94	1.96E-01	0.5989	0.4609	8.27E-01	0.1101	0.5188	2.81E-05	-1.7939	0.3938
5:169691982	rs202013714	D	R	0.66	1.19E-05	0.9289	0.1948	4.99E-01	-0.2059	0.3032	2.78E-02	-0.4911	0.2175
5:169692554	rs2338872	A	G	0.55	8.78E-05	0.6699	0.1597	1.01E-01	-0.3980	0.2433	4.81E-02	-0.3510	0.1738
5:169696233	rs315750	G	C	0.43	2.07E-05	0.8027	0.1740	2.68E-01	-0.2670	0.2429	3.85E-01	-0.1748	0.1995
5:169697707	rs315745	A	G	0.41	5.31E-05	0.8114	0.1868	3.25E-01	-0.2464	0.2523	4.62E-01	-0.1566	0.2113
5:169698028	rs315744	G	A	0.43	1.93E-05	0.8116	0.1752	2.59E-01	-0.2742	0.2445	3.89E-01	-0.1747	0.2011
5:169701017	rs36094591	C	T	0.55	2.27E-06	0.8653	0.1656	5.45E-02	-0.4874	0.2530	5.04E-02	-0.3835	0.1919
5:169704462	rs34027359	G	T	0.54	2.89E-06	0.8858	0.1717	4.35E-02	-0.5291	0.2611	6.84E-02	-0.3697	0.1990
5:170196183	rs111989762	R	I	0.65	7.24E-05	0.8461	0.1989	3.36E-01	-0.2578	0.2650	5.43E-01	-0.1373	0.2241
5:1801426	rs13176353	C	G	0.70	6.57E-02	-0.4435	0.2379	4.91E-01	0.2374	0.3534	6.19E-06	1.0022	0.2012
5:23633738	rs60376038	A	G	0.94	5.05E-02	-0.8515	0.4289	9.06E-05	32.5726	22.2868	1.76E-01	0.5887	0.4292
5:23634339	rs78218086	T	C	0.94	4.98E-02	-0.8534	0.4285	8.72E-05	34.7942	23.9711	1.74E-01	0.5899	0.4288
5:23634477	rs77774270	A	G	0.94	4.98E-02	-0.8531	0.4284	8.22E-05	36.5227	25.0589	1.74E-01	0.5900	0.4287
5:23638196	rs58010901	C	T	0.94	4.64E-02	-0.8626	0.4264	4.59E-05	83.5779	65.4456	1.67E-01	0.5981	0.4269
5:23640678	rs12651892	T	C	0.94	3.49E-02	-0.8789	0.4093	6.06E-05	81.7952	65.8688	2.08E-01	0.5253	0.4128
5:23644775	rs141017921	G	A	0.94	3.53E-02	-0.8766	0.4092	4.47E-05	164.4920	170.4450	2.23E-01	0.5084	0.4130
5:23645653	rs114842633	C	T	0.94	3.47E-02	-0.8792	0.4089	5.45E-05	102.7510	95.0237	2.21E-01	0.5103	0.4127
5:23646176	rs9942370	G	A	0.94	3.42E-02	-0.8812	0.4087	7.15E-05	75.0835	72.7690	2.22E-01	0.5092	0.4126
5:23649257	rs16892718	A	G	0.94	3.41E-02	-0.8822	0.4089	5.20E-05	55.9141	41.3805	2.38E-01	0.4925	0.4133
5:23649406	rs76912780	C	T	0.94	3.47E-02	-0.8790	0.4089	6.20E-05	88.7696	83.3292	2.41E-01	0.4900	0.4132
5:23684132	rs16892798	A	G	0.94	1.19E-02	-0.9854	0.3818	4.00E-05	1273.0300	717.3220	2.87E-01	0.4227	0.3930
5:38757173	rs57488025	T	C	0.79	3.97E-05	-1.0209	0.2306	2.73E-02	0.7493	0.3524	2.73E-01	0.2885	0.2605
5:38757547	rs72746044	G	A	0.87	8.59E-05	-1.0672	0.2539	3.10E-02	0.9048	0.4490	3.63E-01	0.2606	0.2841
5:38759817	rs72746051	G	A	0.87	8.15E-05	-1.0713	0.2540	3.02E-02	0.9137	0.4513	3.59E-01	0.2628	0.2844
5:38762288	rs10045504	C	A	0.82	1.80E-05	-1.0838	0.2329	4.49E-02	0.6851	0.3590	1.18E-01	0.4189	0.2637
5:38762993	rs10512692	G	A	0.88	8.43E-05	-1.1062	0.2629	3.00E-02	0.9291	0.4560	3.48E-01	0.2781	0.2941
5:38768482	rs7736938	A	G	0.88	9.01E-05	-1.0765	0.2570	3.04E-02	0.9317	0.4613	3.56E-01	0.2671	0.2873
5:4659838	rs78516850	R	I	0.66	9.50E-06	0.9011	0.1865	9.59E-01	-0.0146	0.2863	4.02E-01	-0.1831	0.2168
5:4660902	rs2115525	A	G	0.60	7.12E-06	0.8636	0.1758	2.46E-01	-0.3179	0.2735	4.75E-01	-0.1478	0.2057
5:4661919	rs551056882	R	I	0.60	9.21E-06	0.8564	0.1769	2.57E-01	-0.3106	0.2734	4.71E-01	-0.1497	0.2061
5:4662270	rs891192	A	G	0.60	7.67E-06	0.8602	0.1759	2.44E-01	-0.3188	0.2730	4.67E-01	-0.1506	0.2055
5:4662521	rs891193	A	T	0.61	5.60E-06	0.8882	0.1784	2.24E-01	-0.3376	0.2763	3.11E-01	-0.2131	0.2087
5:4663383	rs4701948	A	G	0.60	9.14E-06	0.8538	0.1763	2.35E-01	-0.3243	0.2719	4.40E-01	-0.1596	0.2053

5:4665369	rs2399778	G	T	0.60	1.06E-05	0.8438	0.1758	2.37E-01	-0.3206	0.2701	4.31E-01	-0.1617	0.2041
5:4665670	rs4370242	A	G	0.60	1.10E-05	0.8417	0.1756	2.38E-01	-0.3198	0.2697	4.30E-01	-0.1618	0.2038
5:4665967	rs4397099	C	A	0.60	1.26E-05	0.8346	0.1756	2.37E-01	-0.3187	0.2686	4.21E-01	-0.1647	0.2033
5:4666164	rs7726500	A	G	0.60	1.29E-05	0.8337	0.1757	2.37E-01	-0.3192	0.2685	4.20E-01	-0.1651	0.2032
5:4666170	rs66559905	G	A	0.66	1.29E-05	0.8810	0.1856	9.10E-01	-0.0319	0.2801	3.36E-01	-0.2078	0.2142
5:53853890	rs72750058	A	G	0.91	5.48E-03	0.8341	0.2907	6.91E-05	-0.9747	0.2164	1.61E-01	-0.4267	0.3008
5:53853975	rs72750060	G	A	0.91	5.45E-03	0.8346	0.2907	6.92E-05	-0.9748	0.2164	1.61E-01	-0.4270	0.3008
5:53855263	rs56211050	A	G	0.91	5.29E-03	0.8382	0.2909	6.97E-05	-0.9749	0.2166	1.60E-01	-0.4282	0.3011
5:53855468	rs55989945	T	C	0.91	5.27E-03	0.8387	0.2909	6.98E-05	-0.9750	0.2166	1.60E-01	-0.4283	0.3011
5:53881925	rs2548628	A	G	0.85	2.26E-02	0.6472	0.2779	2.30E-05	-1.0947	0.2366	5.55E-02	-0.5413	0.2769
5:56400390	rs331060	T	G	0.59	3.35E-05	-0.7345	0.1641	3.01E-01	0.2561	0.2492	1.84E-01	0.2488	0.1850
5:56401144	rs331061	C	T	0.59	3.28E-05	-0.7348	0.1639	3.00E-01	0.2567	0.2491	1.83E-01	0.2492	0.1849
5:56401177	rs150845730	R	I	0.66	6.73E-05	-0.8522	0.1994	4.25E-01	0.2307	0.2920	3.03E-01	0.2322	0.2235
5:56401308	rs331063	G	C	0.59	3.27E-05	-0.7350	0.1639	2.97E-01	0.2583	0.2490	1.82E-01	0.2500	0.1849
5:56406503	rs831833	T	A	0.59	3.13E-05	-0.7324	0.1629	2.86E-01	0.2628	0.2480	1.71E-01	0.2544	0.1837
5:56406535	rs831834	C	T	0.59	2.70E-05	-0.7352	0.1620	2.82E-01	0.2645	0.2473	1.73E-01	0.2528	0.1832
5:56406805	rs831835	A	T	0.59	2.06E-05	-0.7516	0.1629	3.00E-01	0.2565	0.2491	1.75E-01	0.2540	0.1850
5:56407479	rs831836	C	T	0.59	2.60E-05	-0.7352	0.1616	2.80E-01	0.2649	0.2469	1.72E-01	0.2530	0.1828
5:56407505	rs831837	A	G	0.59	2.61E-05	-0.7349	0.1616	2.80E-01	0.2653	0.2469	1.72E-01	0.2531	0.1828
5:56407611	rs831839	T	C	0.59	2.58E-05	-0.7347	0.1615	2.78E-01	0.2658	0.2468	1.71E-01	0.2532	0.1827
5:56410116	rs831841	G	A	0.58	2.36E-05	-0.7329	0.1602	2.71E-01	0.2688	0.2456	1.67E-01	0.2541	0.1814
5:56410413	rs831842	A	C	0.58	2.36E-05	-0.7322	0.1600	2.69E-01	0.2694	0.2455	1.66E-01	0.2542	0.1812
5:56411228	rs831844	T	A	0.58	2.32E-05	-0.7315	0.1597	2.68E-01	0.2701	0.2452	1.65E-01	0.2544	0.1809
5:56412285	rs831846	A	T	0.58	2.47E-05	-0.7268	0.1593	2.57E-01	0.2750	0.2441	1.73E-01	0.2490	0.1803
5:56413057	rs831848	A	C	0.58	2.25E-05	-0.7291	0.1589	2.63E-01	0.2719	0.2444	1.63E-01	0.2547	0.1800
5:56415072	rs831849	T	C	0.58	2.21E-05	-0.7275	0.1583	2.59E-01	0.2732	0.2439	1.61E-01	0.2549	0.1794
5:56415596	rs831850	C	T	0.58	2.01E-05	-0.7354	0.1591	2.61E-01	0.2741	0.2454	1.61E-01	0.2567	0.1806
5:56415649	rs831851	C	T	0.58	2.21E-05	-0.7275	0.1584	2.59E-01	0.2732	0.2439	1.61E-01	0.2549	0.1795
5:56415918	rs831852	T	G	0.58	2.21E-05	-0.7275	0.1584	2.59E-01	0.2732	0.2439	1.61E-01	0.2549	0.1795
5:56417472	rs831854	A	G	0.58	2.21E-05	-0.7276	0.1584	2.59E-01	0.2732	0.2439	1.61E-01	0.2549	0.1795
5:56417561	rs831855	C	T	0.58	2.21E-05	-0.7276	0.1584	2.59E-01	0.2732	0.2439	1.61E-01	0.2549	0.1795
5:56418425	rs331050	G	A	0.58	2.25E-05	-0.7273	0.1585	2.59E-01	0.2733	0.2441	1.60E-01	0.2556	0.1795
5:56420434	rs71602979	R	I	0.62	1.53E-05	-0.7787	0.1657	9.61E-02	0.4470	0.2691	2.36E-01	0.2272	0.1899
5:56420468	rs331052	T	C	0.58	2.21E-05	-0.7276	0.1584	2.59E-01	0.2731	0.2440	1.61E-01	0.2550	0.1795

5:56420880	rs331053	T	C	0.58	2.21E-05	-0.7276	0.1584	2.59E-01	0.2731	0.2440	1.61E-01	0.2550	0.1795
5:56421267	rs331054	C	A	0.58	2.21E-05	-0.7276	0.1584	2.59E-01	0.2731	0.2440	1.61E-01	0.2550	0.1795
5:56422894	rs142251087	R	D	0.58	2.21E-05	-0.7275	0.1584	2.59E-01	0.2732	0.2439	1.61E-01	0.2549	0.1795
5:56422960	rs331055	C	T	0.58	2.07E-05	-0.7323	0.1588	2.60E-01	0.2740	0.2447	1.61E-01	0.2560	0.1801
5:56424014	rs331056	A	G	0.58	2.21E-05	-0.7276	0.1584	2.60E-01	0.2731	0.2440	1.61E-01	0.2549	0.1795
5:56425952	rs331029	A	G	0.58	2.20E-05	-0.7281	0.1584	2.60E-01	0.2729	0.2441	1.61E-01	0.2553	0.1796
5:56427689	rs35753690	R	I	0.58	2.20E-05	-0.7282	0.1584	2.59E-01	0.2736	0.2440	1.61E-01	0.2552	0.1796
5:56427777	rs331028	A	C	0.58	2.21E-05	-0.7275	0.1583	2.59E-01	0.2732	0.2439	1.61E-01	0.2549	0.1794
5:56428061	rs179463	G	T	0.58	2.21E-05	-0.7275	0.1583	2.59E-01	0.2732	0.2439	1.61E-01	0.2549	0.1794
5:56428564	rs331027	T	C	0.58	2.21E-05	-0.7275	0.1583	2.59E-01	0.2732	0.2439	1.61E-01	0.2549	0.1794
5:56428724	rs331026	T	G	0.58	2.21E-05	-0.7277	0.1584	2.60E-01	0.2730	0.2440	1.61E-01	0.2551	0.1795
5:56428955	rs2694490	T	G	0.58	1.95E-05	-0.7356	0.1589	2.58E-01	0.2744	0.2444	1.58E-01	0.2580	0.1804
5:56428956	rs2694489	T	A	0.58	1.95E-05	-0.7356	0.1589	2.58E-01	0.2744	0.2444	1.58E-01	0.2580	0.1804
5:56429366	rs331023	G	C	0.58	2.07E-05	-0.7323	0.1588	2.60E-01	0.2740	0.2447	1.61E-01	0.2560	0.1801
5:56429838	rs34711995	R	D	0.58	2.48E-05	-0.7231	0.1585	2.96E-01	0.2508	0.2425	1.62E-01	0.2541	0.1793
5:56429961	rs331022	C	A	0.58	2.21E-05	-0.7275	0.1583	2.59E-01	0.2732	0.2439	1.61E-01	0.2549	0.1794
5:56430330	rs331021	C	A	0.58	2.21E-05	-0.7275	0.1583	2.59E-01	0.2732	0.2439	1.61E-01	0.2549	0.1794
5:56431154	rs331020	C	T	0.58	2.19E-05	-0.7281	0.1584	2.59E-01	0.2734	0.2440	1.61E-01	0.2551	0.1795
5:56432156	rs191346	C	T	0.58	2.10E-05	-0.7339	0.1592	2.59E-01	0.2749	0.2454	1.64E-01	0.2544	0.1806
5:56434286	rs331018	A	G	0.58	2.15E-05	-0.7296	0.1585	2.59E-01	0.2735	0.2442	1.60E-01	0.2556	0.1797
5:56434685	rs33987444	R	D	0.58	2.14E-05	-0.7299	0.1586	2.59E-01	0.2736	0.2443	1.60E-01	0.2557	0.1798
5:56435406	rs831858	T	C	0.58	2.13E-05	-0.7304	0.1586	2.60E-01	0.2736	0.2444	1.60E-01	0.2558	0.1798
5:56435767	rs831859	T	C	0.58	2.12E-05	-0.7305	0.1586	2.59E-01	0.2737	0.2444	1.60E-01	0.2558	0.1799
5:56436119	rs831860	T	A	0.58	2.12E-05	-0.7309	0.1587	2.60E-01	0.2738	0.2445	1.60E-01	0.2560	0.1799
5:56437846	rs1096605	T	C	0.58	2.09E-05	-0.7317	0.1587	2.59E-01	0.2740	0.2446	1.60E-01	0.2562	0.1800
5:56437919	rs1096606	G	A	0.58	2.09E-05	-0.7318	0.1587	2.59E-01	0.2740	0.2446	1.60E-01	0.2563	0.1800
5:56438077	rs35986638	R	I	0.59	6.47E-05	-0.7273	0.1697	3.67E-01	0.2251	0.2512	1.98E-01	0.2467	0.1894
5:56439105	rs1096608	C	T	0.58	2.03E-05	-0.7339	0.1589	2.60E-01	0.2743	0.2450	1.60E-01	0.2570	0.1803
5:56439559	rs831861	C	G	0.58	2.03E-05	-0.7342	0.1590	2.60E-01	0.2744	0.2450	1.60E-01	0.2571	0.1804
5:56439898	rs1096609	A	C	0.58	2.02E-05	-0.7344	0.1590	2.59E-01	0.2745	0.2451	1.60E-01	0.2571	0.1804
5:56441324	rs1096611	T	G	0.58	1.96E-05	-0.7369	0.1592	2.59E-01	0.2751	0.2455	1.59E-01	0.2580	0.1808
5:56441617	rs1096612	G	A	0.58	1.91E-05	-0.7390	0.1594	2.60E-01	0.2752	0.2459	1.59E-01	0.2584	0.1811
5:56442625	rs1096615	G	A	0.58	1.94E-05	-0.7377	0.1593	2.59E-01	0.2753	0.2457	1.59E-01	0.2582	0.1809
5:56442862	rs1096616	G	A	0.58	1.94E-05	-0.7379	0.1593	2.59E-01	0.2753	0.2457	1.59E-01	0.2583	0.1809

5:56455651	rs1976569	A	G	0.60	8.34E-05	-0.7558	0.1795	8.39E-01	0.0555	0.2712	8.84E-02	0.3419	0.1973
5:56456595	rs6450419	C	T	0.53	5.59E-06	-0.8618	0.1731	9.84E-01	0.0054	0.2619	8.42E-02	0.3498	0.1990
5:56457598	rs7712889	T	C	0.60	8.02E-05	-0.7570	0.1793	8.38E-01	0.0557	0.2708	9.12E-02	0.3389	0.1972
5:56477261	rs11417693	R	I	0.65	2.80E-05	-0.8757	0.1934	9.47E-01	0.0204	0.3073	6.93E-02	0.3995	0.2158
5:56481122	rs831642	G	C	0.60	7.63E-05	-0.7595	0.1792	8.43E-01	0.0542	0.2717	9.33E-02	0.3370	0.1974
5:56482215	rs34384211	R	I	0.65	2.11E-05	-0.8644	0.1876	1.72E-01	0.3906	0.2902	4.61E-02	0.4261	0.2090
5:56482501	rs864963	T	C	0.59	2.04E-05	-0.8107	0.1756	7.54E-01	0.0848	0.2691	6.88E-02	0.3653	0.1969
5:56483713	rs35653365	R	D	0.60	7.51E-05	-0.7613	0.1795	8.40E-01	0.0552	0.2725	9.09E-02	0.3399	0.1977
5:56494467	rs831650	A	G	0.58	2.38E-05	-0.7973	0.1743	8.77E-01	-0.0385	0.2485	9.43E-02	0.3333	0.1959
5:56504311	rs831654	T	C	0.60	7.17E-05	-0.7644	0.1796	8.50E-01	0.0518	0.2732	9.43E-02	0.3370	0.1981
5:56504664	rs1096565	C	T	0.60	7.18E-05	-0.7644	0.1796	8.51E-01	0.0515	0.2732	9.43E-02	0.3371	0.1981
5:56512536	rs6869334	G	A	0.60	7.03E-05	-0.7656	0.1797	8.53E-01	0.0511	0.2736	9.50E-02	0.3366	0.1982
5:56515260	rs5012224	G	A	0.52	2.61E-05	-0.8015	0.1763	9.90E-01	0.0033	0.2709	7.29E-02	0.3599	0.1970
5:56516742	rs77026127	D	R	0.60	1.39E-05	-0.8298	0.1756	7.42E-01	0.0901	0.2715	6.66E-02	0.3706	0.1981
5:56538855	rs12153538	T	C	0.60	1.21E-05	-0.8362	0.1755	7.40E-01	0.0912	0.2723	6.59E-02	0.3720	0.1984
5:56544347	rs2408687	T	C	0.60	1.30E-05	-0.8337	0.1758	7.81E-01	0.0763	0.2720	6.77E-02	0.3698	0.1985
5:56574452	rs4700181	C	T	0.61	6.44E-05	-0.7736	0.1805	8.67E-01	0.0466	0.2763	9.72E-02	0.3364	0.1995
5:56596940	rs185307	A	G	0.66	7.34E-05	-0.8742	0.2058	3.38E-01	0.2735	0.2996	4.16E-01	0.1895	0.2312
5:65145584	rs147731910	T	C	0.75	4.63E-01	0.1729	0.2356	7.46E-05	-0.9896	0.2391	8.97E-02	-0.3917	0.2269
5:71204261	rs2879027	T	G	0.57	3.61E-02	-0.3540	0.1660	7.16E-04	0.7561	0.2220	4.31E-05	0.6485	0.1463
5:73765232	rs59074070	C	T	0.63	2.83E-03	-0.5398	0.1741	2.32E-05	1.0398	0.2640	6.57E-01	0.0824	0.1849
5:73776280	rs60150178	T	C	0.79	8.75E-05	-0.7979	0.1901	1.41E-03	1.2263	0.4391	4.89E-01	0.1486	0.2133
5:75225918	rs72770380	T	C	0.90	9.76E-01	-0.0080	0.2719	3.18E-01	0.3386	0.3403	4.37E-05	1.0209	0.2306
5:75244122	rs1404990	A	T	0.92	3.26E-01	-0.2872	0.2914	4.75E-02	0.7848	0.4254	4.23E-05	1.1055	0.2492
5:7789175	rs12656715	A	G	0.59	9.98E-05	0.7620	0.1832	2.04E-02	-0.5524	0.2340	2.83E-01	-0.2209	0.2039
5:7790217	rs3816620	C	A	0.58	6.24E-05	0.7604	0.1770	1.56E-02	-0.5623	0.2288	1.60E-01	-0.2808	0.1971
5:7792171	rs16879108	C	G	0.58	6.60E-05	0.7599	0.1776	1.65E-02	-0.5592	0.2297	1.61E-01	-0.2807	0.1976
5:7794503	rs16879116	A	G	0.58	7.00E-05	0.7593	0.1781	1.76E-02	-0.5551	0.2307	1.62E-01	-0.2803	0.1980
5:7794694	rs76946048	R	D	0.58	6.94E-05	0.7583	0.1778	1.81E-02	-0.5528	0.2306	1.63E-01	-0.2793	0.1977
5:7796625	rs7728943	G	A	0.58	6.76E-05	0.7636	0.1787	1.77E-02	-0.5566	0.2317	1.57E-01	-0.2848	0.1987
5:79855876	rs187262	C	T	0.76	3.90E-01	-0.2058	0.2391	3.03E-02	0.6530	0.3038	9.40E-05	0.8690	0.2068
5:9447717	rs3798028	G	T	0.65	3.24E-05	0.7931	0.1768	5.90E-01	-0.1405	0.2453	8.45E-01	-0.0398	0.2025
5:9451937	rs6867962	C	T	0.66	8.08E-05	0.8165	0.1935	5.00E-01	-0.1888	0.2620	8.96E-01	-0.0286	0.2182
6:104213975	rs12192060	A	T	0.64	7.17E-01	-0.0926	0.2560	9.72E-01	-0.0091	0.2625	3.91E-05	0.9675	0.2169

6:130778955	rs11154583	C	A	0.91	5.88E-01	-0.1758	0.3249	7.09E-05	8.8347	9.2401	4.54E-01	0.2405	0.3187
6:144151026	rs117010774	T	A	0.93	5.97E-01	0.2086	0.3945	9.65E-05	477.6610	361.2880	3.99E-01	0.3285	0.3864
6:144152872	rs117055821	T	G	0.93	5.97E-01	0.2086	0.3945	9.65E-05	477.6610	361.2880	3.99E-01	0.3285	0.3864
6:145207218	rs7762951	T	C	0.79	9.73E-01	0.0082	0.2431	3.55E-01	0.3122	0.3475	8.07E-05	-0.8848	0.2083
6:145207267	rs7742815	C	G	0.79	9.72E-01	0.0085	0.2431	3.52E-01	0.3143	0.3476	8.20E-05	-0.8842	0.2084
6:145207301	rs7743204	G	A	0.80	7.16E-01	-0.0887	0.2439	4.52E-01	0.2542	0.3476	9.05E-05	-0.8833	0.2096
6:145302412	rs7742978	C	G	0.86	6.19E-01	0.1409	0.2833	5.80E-01	0.2023	0.3764	4.00E-05	-1.0706	0.2404
6:145304007	rs9373439	T	C	0.87	6.29E-01	0.1398	0.2893	6.95E-01	0.1449	0.3795	9.63E-05	-1.0452	0.2491
6:145304099	rs9386094	T	G	0.87	6.28E-01	0.1399	0.2893	6.95E-01	0.1450	0.3795	9.63E-05	-1.0451	0.2491
6:145304594	rs9373440	T	C	0.86	6.07E-01	0.1453	0.2828	5.75E-01	0.2047	0.3760	4.04E-05	-1.0686	0.2401
6:145306013	rs9399523	A	G	0.87	6.17E-01	0.1442	0.2888	6.87E-01	0.1489	0.3792	9.84E-05	-1.0423	0.2488
6:149569618	rs564507597	R	D	0.50	4.86E-01	0.1461	0.2097	1.81E-05	-1.0588	0.2568	2.37E-02	-0.4599	0.1979
6:152502217	rs11439578	I	R	0.44	1.23E-05	0.9516	0.1999	5.60E-02	-0.6237	0.3203	7.25E-01	0.0824	0.2326
6:152504123	rs113956064	I	R	0.50	1.59E-05	0.8265	0.1763	1.91E-02	-0.6326	0.2686	7.68E-01	-0.0605	0.2043
6:152504874	rs2813511	T	A	0.50	1.60E-05	0.8256	0.1762	1.94E-02	-0.6301	0.2683	7.67E-01	-0.0608	0.2041
6:152505056	rs2813512	G	T	0.50	1.59E-05	0.8256	0.1761	1.95E-02	-0.6294	0.2682	7.67E-01	-0.0607	0.2041
6:152506734	rs2813513	T	C	0.50	1.54E-05	0.8251	0.1757	1.99E-02	-0.6261	0.2677	7.58E-01	-0.0632	0.2037
6:152506825	rs2813514	T	G	0.50	1.55E-05	0.8250	0.1758	1.99E-02	-0.6263	0.2677	7.56E-01	-0.0635	0.2037
6:152507116	rs2635436	A	G	0.50	1.55E-05	0.8245	0.1756	2.00E-02	-0.6255	0.2676	7.54E-01	-0.0641	0.2036
6:152508197	rs2763015	C	A	0.47	2.47E-05	0.7417	0.1625	4.21E-02	-0.5053	0.2496	7.38E-01	-0.0683	0.2029
6:152509307	rs2635431	A	T	0.50	1.49E-05	0.8221	0.1747	2.09E-02	-0.6190	0.2668	7.27E-01	-0.0711	0.2025
6:153507300	rs191150	A	G	0.48	5.36E-05	0.7269	0.1675	8.18E-02	-0.3744	0.2128	6.16E-01	-0.0958	0.1898
6:153509860	rs600083	T	C	0.48	6.25E-05	0.7175	0.1670	8.54E-02	-0.3688	0.2122	6.08E-01	-0.0975	0.1888
6:153523669	rs316296	C	G	0.46	5.52E-05	0.6831	0.1577	1.38E-01	-0.3030	0.2031	5.44E-01	-0.1089	0.1785
6:153524338	rs160784	A	C	0.46	5.56E-05	0.6833	0.1578	1.38E-01	-0.3033	0.2032	5.44E-01	-0.1091	0.1786
6:153528141	rs317077	G	A	0.48	3.30E-05	0.7240	0.1616	1.61E-01	-0.2939	0.2071	4.34E-01	-0.1451	0.1841
6:162170039	rs1569836	C	T	0.69	9.31E-02	0.4712	0.2777	6.82E-01	-0.1356	0.3274	4.34E-05	-1.0694	0.2414
6:166109355	rs12055769	C	T	0.84	6.12E-05	-1.2068	0.2805	2.94E-01	0.5377	0.5346	1.69E-01	0.4359	0.3127
6:29646165	rs396660	C	T	0.74	1.08E-02	-0.5665	0.2164	2.51E-01	0.3552	0.3110	9.94E-05	0.8250	0.1971
6:29646879	rs445150	T	C	0.74	1.08E-02	-0.5665	0.2164	2.51E-01	0.3552	0.3110	9.94E-05	0.8250	0.1971
6:29647628	rs416568	T	A	0.78	1.52E-02	-0.5639	0.2268	1.54E-01	0.4134	0.2929	2.64E-05	0.9189	0.2010
6:29648398	rs365052	G	C	0.78	1.53E-02	-0.5637	0.2268	1.54E-01	0.4133	0.2930	2.64E-05	0.9189	0.2010
6:29648506	rs2747430	G	A	0.74	1.08E-02	-0.5665	0.2164	2.51E-01	0.3552	0.3110	9.94E-05	0.8250	0.1971
6:29648564	rs2747431	C	T	0.78	1.52E-02	-0.5639	0.2268	1.54E-01	0.4134	0.2929	2.64E-05	0.9189	0.2010

6:29654409	rs3129044	G	A	0.80	1.08E-02	-0.6328	0.2418	1.72E-01	0.4192	0.3050	8.18E-05	0.9314	0.2195
6:29740477	rs1610638	C	T	0.88	6.36E-03	-0.8235	0.2925	8.14E-02	0.6825	0.3996	7.46E-05	1.1419	0.2674
6:29742003	rs1610639	G	A	0.88	6.26E-03	-0.8240	0.2921	8.10E-02	0.6829	0.3994	7.50E-05	1.1402	0.2671
6:29771621	rs1610660	C	G	0.88	6.37E-03	-0.8235	0.2925	8.14E-02	0.6826	0.3996	7.44E-05	1.1422	0.2674
6:29902279	rs9259928	C	G	0.85	5.43E-03	-0.8305	0.2891	1.06E-01	0.6239	0.3945	4.37E-05	1.1625	0.2626
6:31315706	rs9265969	G	A	0.92	8.27E-02	-0.6171	0.3516	1.28E-01	0.7122	0.4854	6.99E-05	1.3243	0.3087
6:35044456	rs2762337	A	C	0.50	3.48E-01	-0.1901	0.2020	7.87E-05	0.9275	0.2345	2.11E-01	0.2497	0.1974
6:52556771	rs11969858	T	C	0.76	4.04E-02	0.4909	0.2355	6.24E-02	-0.5398	0.2876	6.87E-05	-0.8972	0.2089
6:52558017	rs6902435	T	C	0.76	4.02E-02	0.4913	0.2355	6.26E-02	-0.5393	0.2875	6.85E-05	-0.8974	0.2089
6:52560231	rs11759535	G	A	0.76	4.02E-02	0.4914	0.2356	6.22E-02	-0.5404	0.2877	7.00E-05	-0.8967	0.2090
6:52560788	rs6458856	A	G	0.76	4.04E-02	0.4907	0.2355	6.22E-02	-0.5402	0.2876	7.00E-05	-0.8963	0.2089
6:52563007	rs70977377	R	D	0.82	1.30E-01	-0.5162	0.3382	5.87E-02	-0.5511	0.2886	6.97E-05	-0.9025	0.2103
6:52563255	rs9474303	C	T	0.76	3.98E-02	0.4923	0.2355	6.20E-02	-0.5407	0.2876	6.95E-05	-0.8968	0.2090
6:52564490	rs10484873	A	G	0.76	4.10E-02	0.4900	0.2358	6.33E-02	-0.5389	0.2881	7.01E-05	-0.8972	0.2092
6:52564785	rs62405938	T	G	0.76	4.06E-02	0.4909	0.2358	6.32E-02	-0.5391	0.2881	7.00E-05	-0.8975	0.2092
6:52566178	rs62405939	C	T	0.76	3.96E-02	0.4932	0.2357	6.29E-02	-0.5396	0.2881	7.06E-05	-0.8968	0.2092
6:54648365	rs62412578	T	C	0.93	8.87E-01	0.0587	0.4138	5.01E-01	-0.2957	0.4358	8.70E-05	-1.5005	0.3551
6:54650151	rs62412580	C	A	0.93	8.73E-01	0.0663	0.4140	4.98E-01	-0.2979	0.4356	8.15E-05	-1.5065	0.3549
6:54660244	rs79244342	C	G	0.93	7.60E-01	0.1264	0.4150	4.61E-01	-0.3236	0.4334	4.99E-05	-1.5495	0.3531
6:54664045	rs62412597	C	A	0.93	7.27E-01	0.1438	0.4129	4.43E-01	-0.3354	0.4318	4.33E-05	-1.5526	0.3505
6:54669411	rs12661022	A	G	0.94	7.25E-01	0.1457	0.4146	4.63E-01	-0.3215	0.4335	4.62E-05	-1.5542	0.3524
6:54670334	rs73430625	C	T	0.93	7.20E-01	0.1481	0.4140	4.44E-01	-0.3368	0.4336	4.39E-05	-1.5558	0.3515
6:70814202	rs6907532	G	A	0.67	1.43E-02	-0.4429	0.1765	1.08E-01	0.3502	0.2230	9.38E-05	0.6719	0.1599
6:70816221	rs59608953	R	I	0.52	4.33E-05	0.7222	0.1641	5.34E-02	-0.4581	0.2401	7.97E-05	-0.6929	0.1630
6:70832305	rs3840402	D	R	0.67	1.36E-02	-0.4478	0.1770	1.13E-01	0.3477	0.2243	7.35E-05	0.6834	0.1599
6:70833413	rs9454978	C	T	0.66	1.88E-02	-0.4299	0.1789	1.28E-01	0.3352	0.2243	7.53E-05	0.6863	0.1608
6:70858817	rs5877241	D	R	0.66	3.57E-02	-0.3981	0.1862	1.34E-01	0.3567	0.2435	2.38E-06	0.8202	0.1563
6:70858863	rs2025283	C	T	0.68	3.48E-02	-0.3885	0.1808	1.03E-01	0.3779	0.2371	1.66E-05	0.7382	0.1569
6:70861911	rs11302917	D	R	0.69	3.68E-02	-0.3888	0.1830	9.44E-02	0.3961	0.2424	1.50E-05	0.7499	0.1584
6:70870045	rs9454994	G	A	0.70	3.86E-02	-0.3815	0.1813	9.50E-02	0.3920	0.2406	1.90E-05	0.7350	0.1575
6:86150547	rs373978998	R	I	0.86	2.33E-02	0.6813	0.2941	3.31E-01	-0.3351	0.3366	5.61E-05	-1.1417	0.2622
6:92123133	rs78071196	T	C	0.95	1.01E-01	-0.8576	0.5174	8.55E-05	4.4222	1.8483	1.99E-01	0.6672	0.5134
7:101562674	rs17407001	C	T	0.90	4.31E-01	-0.2687	0.3412	9.81E-01	0.0092	0.4109	2.54E-05	1.3218	0.2883
7:101568195	rs35808488	C	T	0.90	4.38E-01	-0.2631	0.3388	9.92E-01	0.0039	0.4081	2.18E-05	1.3212	0.2855

7:103395578	rs147422867	R	I	0.89	1.12E-05	1.3877	0.2900	2.15E-01	-0.4508	0.3580	5.42E-01	-0.2069	0.3371
7:103676845	rs67058168	R	D	0.86	3.01E-02	-0.6963	0.3151	1.46E-02	1.1062	0.4783	9.92E-05	1.1827	0.2825
7:103704359	rs200787519	C	T	0.90	2.67E-02	-1.0006	0.4426	7.36E-02	1.1480	0.6807	3.06E-05	1.7655	0.3897
7:11404383	rs17623238	G	T	0.55	8.71E-01	-0.0325	0.2010	6.23E-01	-0.1176	0.2334	9.55E-05	-0.7251	0.1727
7:13678247	rs13438397	T	C	0.75	7.11E-02	-0.3858	0.2111	1.05E-01	0.5052	0.3203	6.27E-05	0.8013	0.1854
7:148629591	rs76123998	C	T	0.94	9.03E-05	-1.6079	0.3839	2.34E-01	0.7045	0.6379	3.29E-01	0.4225	0.4288
7:148647655	rs79879124	A	C	0.92	3.97E-05	-1.3361	0.3018	2.20E-01	0.6199	0.5514	3.10E-01	0.3500	0.3415
7:148667418	rs34076499	G	A	0.88	1.85E-05	-1.1682	0.2515	1.96E-01	0.4672	0.3768	4.05E-01	0.2424	0.2891
7:148667593	rs35069049	T	C	0.92	1.03E-05	-1.3242	0.2753	1.91E-01	0.6123	0.5092	2.61E-01	0.3612	0.3180
7:154509366	rs10276166	C	G	0.53	8.01E-05	0.8814	0.2087	5.63E-01	-0.1557	0.2712	2.34E-01	-0.2797	0.2326
7:24245677	rs73074503	C	T	0.85	3.09E-02	0.5878	0.2673	4.33E-05	-1.0215	0.2322	6.69E-01	-0.1177	0.2734
7:24257645	rs34291079	R	I	0.84	2.99E-02	0.5952	0.2689	1.67E-05	-1.0709	0.2298	5.67E-01	-0.1583	0.2748
7:24432431	rs2722390	G	A	0.66	2.23E-01	0.2549	0.2081	9.97E-05	-0.7844	0.1930	2.38E-01	-0.2439	0.2048
7:28946439	rs144799702	C	T	0.71	3.26E-01	0.2092	0.2123	8.07E-05	-0.8448	0.2086	1.66E-02	-0.4940	0.2001
7:29000374	rs717891	T	C	0.67	5.66E-03	-0.5587	0.1955	9.86E-05	1.0702	0.2845	5.89E-01	0.1114	0.2052
7:31243943	rs177980	G	A	0.51	6.10E-05	0.7741	0.1799	2.87E-01	-0.2622	0.2428	1.64E-01	-0.2829	0.2005
7:31261967	rs373352538	R	D	0.52	5.20E-05	0.8544	0.1964	1.44E-01	-0.3741	0.2545	9.77E-02	-0.3670	0.2179
7:42215217	rs545191898	R	I	0.90	4.23E-05	-1.5279	0.3465	8.90E-02	1.1783	0.7787	9.68E-01	-0.0160	0.3953
7:52383136	rs139611864	R	I	0.69	8.72E-01	-0.0439	0.2725	5.19E-02	0.6974	0.3490	7.62E-05	0.9952	0.2334
7:55939374	rs6958601	C	T	0.56	3.55E-03	-0.6278	0.2077	2.74E-02	0.5559	0.2511	8.42E-05	0.8133	0.1920
7:64609834	rs556658156	R	D	0.54	5.82E-05	0.9059	0.2099	1.41E-01	-0.4768	0.3261	7.55E-02	-0.4192	0.2315
7:64611403	rs62455680	G	A	0.51	9.95E-05	0.7977	0.1918	2.75E-01	-0.3140	0.2737	7.57E-02	-0.3793	0.2097
7:64620389	rs62455682	G	C	0.51	8.51E-05	0.8052	0.1915	2.70E-01	-0.3171	0.2737	7.44E-02	-0.3815	0.2099
7:64686829	rs199794749	R	D	0.50	9.73E-05	0.7716	0.1852	2.60E-01	-0.3084	0.2625	7.73E-02	-0.3646	0.2027
7:64687855	rs9769451	G	A	0.49	8.00E-05	0.7723	0.1829	2.22E-01	-0.3305	0.2606	1.44E-01	-0.3002	0.2025
7:64699988	rs3983284	C	A	0.57	5.94E-05	0.7735	0.1794	1.73E-01	-0.3574	0.2514	5.21E-02	-0.3905	0.1968
7:64712708	rs10252247	G	A	0.54	4.28E-05	0.8029	0.1823	2.12E-01	-0.3346	0.2558	1.80E-02	-0.4817	0.1978
7:64725199	rs11760459	G	A	0.55	8.20E-05	0.7735	0.1835	1.14E-01	-0.4133	0.2520	5.36E-02	-0.3946	0.2002
7:64805015	rs55843957	A	G	0.69	9.40E-05	0.8095	0.1938	1.47E-02	-0.6670	0.2770	9.94E-02	-0.3567	0.2130
7:64809792	rs6948045	T	C	0.70	6.84E-05	0.8277	0.1939	8.57E-03	-0.7266	0.2817	6.88E-02	-0.3951	0.2130
7:64901271	rs118049311	A	G	0.70	8.04E-05	0.8127	0.1925	3.49E-04	-0.9304	0.2584	4.46E-02	-0.4305	0.2096
7:66780745	rs62466399	G	A	0.89	2.68E-01	-0.3979	0.3584	4.64E-01	-0.3512	0.4747	6.75E-05	1.3309	0.3095
7:86090323	rs55811404	D	R	0.60	1.77E-01	0.2773	0.2040	3.10E-01	-0.2597	0.2536	1.75E-05	-0.8121	0.1731
7:96935922	rs117665874	A	T	0.95	4.54E-02	0.8764	0.4311	6.01E-01	-0.2799	0.5261	6.72E-05	-1.6413	0.3815

8:106507619	rs2622636	A	G	0.69	2.30E-01	0.2212	0.1833	6.85E-01	0.0919	0.2248	6.17E-05	-0.6851	0.1584
8:106509975	rs2622633	G	A	0.69	2.35E-01	0.2180	0.1830	6.92E-01	0.0895	0.2247	6.69E-05	-0.6810	0.1583
8:113394520	rs535052089	R	I	0.68	1.13E-01	-0.3538	0.2211	2.65E-01	0.3021	0.2802	7.60E-05	0.8256	0.1936
8:113659531	rs149033623	R	I	0.75	2.13E-02	0.4865	0.2068	1.01E-01	-0.3605	0.2242	8.17E-05	-0.7883	0.1857
8:113663094	rs372360280	R	I	0.80	3.74E-02	0.5093	0.2405	4.59E-01	-0.1968	0.2722	9.12E-05	-0.9039	0.2146
8:117400679	rs57634789	G	T	0.84	5.47E-02	0.4948	0.2538	7.30E-05	-0.9200	0.2139	5.97E-01	-0.1369	0.2572
8:117415285	rs11986435	C	T	0.82	5.84E-02	0.5219	0.2720	4.24E-05	-1.0095	0.2260	6.05E-01	-0.1432	0.2754
8:133983048	rs10106773	A	G	0.78	3.30E-01	-0.2298	0.2355	1.45E-01	0.4413	0.3112	5.73E-05	0.8799	0.2023
8:133983516	rs72727452	A	T	0.78	3.30E-01	-0.2297	0.2352	1.46E-01	0.4394	0.3106	5.70E-05	0.8791	0.2021
8:133983955	rs72727453	A	G	0.78	3.30E-01	-0.2295	0.2351	1.47E-01	0.4376	0.3104	5.67E-05	0.8788	0.2020
8:133984471	rs77233731	T	C	0.78	3.40E-01	-0.2234	0.2336	1.48E-01	0.4327	0.3078	5.59E-05	0.8734	0.2005
8:133985019	rs1403488	G	A	0.78	3.89E-01	-0.2001	0.2321	1.40E-01	0.4388	0.3057	6.33E-05	0.8610	0.1994
8:133985059	rs1403487	A	G	0.78	3.89E-01	-0.2000	0.2320	1.41E-01	0.4370	0.3055	6.34E-05	0.8607	0.1993
8:133985395	rs2222452	G	A	0.78	3.89E-01	-0.2002	0.2320	1.40E-01	0.4388	0.3057	6.34E-05	0.8609	0.1993
8:133985931	rs28680551	G	A	0.78	3.90E-01	-0.1995	0.2319	1.40E-01	0.4386	0.3054	6.36E-05	0.8602	0.1992
8:133986126	rs56184142	T	C	0.78	3.86E-01	-0.2015	0.2322	1.44E-01	0.4347	0.3060	6.83E-05	0.8582	0.1997
8:133986609	rs6982788	C	T	0.78	3.91E-01	-0.1990	0.2316	1.40E-01	0.4371	0.3050	6.36E-05	0.8592	0.1990
8:133986723	rs1403485	G	A	0.78	3.91E-01	-0.1989	0.2316	1.41E-01	0.4367	0.3049	6.39E-05	0.8588	0.1990
8:133986945	rs1403484	G	C	0.77	3.03E-01	-0.2431	0.2356	1.78E-01	0.4067	0.3088	6.69E-05	0.8740	0.2031
8:133987011	rs2091036	A	G	0.78	3.90E-01	-0.2010	0.2335	1.43E-01	0.4367	0.3074	7.18E-05	0.8606	0.2010
8:133988414	rs6993411	G	A	0.77	3.15E-01	-0.2347	0.2328	1.61E-01	0.4182	0.3050	5.52E-05	0.8720	0.2000
8:133988608	rs7013696	T	G	0.78	3.96E-01	-0.1966	0.2314	1.56E-01	0.4207	0.3039	6.46E-05	0.8576	0.1988
8:133989099	rs33951544	R	D	0.78	3.96E-01	-0.1965	0.2311	1.41E-01	0.4352	0.3040	6.40E-05	0.8569	0.1985
8:133989954	rs9297860	C	T	0.78	3.98E-01	-0.1955	0.2309	1.42E-01	0.4335	0.3035	6.46E-05	0.8555	0.1983
8:133990609	rs10956685	C	T	0.78	3.98E-01	-0.1953	0.2307	1.42E-01	0.4325	0.3032	6.45E-05	0.8549	0.1982
8:133992211	rs10093801	G	A	0.78	4.09E-01	-0.1910	0.2309	1.53E-01	0.4210	0.3027	6.41E-05	0.8558	0.1983
8:133994143	rs10101716	G	T	0.78	4.04E-01	-0.1920	0.2298	1.44E-01	0.4286	0.3017	6.53E-05	0.8510	0.1975
8:133994520	rs10101897	C	T	0.78	4.03E-01	-0.1923	0.2298	1.45E-01	0.4279	0.3018	6.51E-05	0.8513	0.1975
8:133995376	rs28433340	A	G	0.78	4.03E-01	-0.1926	0.2299	1.45E-01	0.4279	0.3018	6.52E-05	0.8514	0.1975
8:133996378	rs56263647	C	T	0.78	4.02E-01	-0.1929	0.2300	1.46E-01	0.4270	0.3020	6.42E-05	0.8523	0.1975
8:133998625	rs528421340	G	A	0.79	3.18E-01	-0.2674	0.2673	1.62E-01	0.4799	0.3546	3.85E-05	1.0188	0.2282
8:133998729	rs60808830	C	T	0.77	3.23E-01	-0.2287	0.2308	1.33E-01	0.4442	0.3036	4.60E-05	0.8719	0.1976
8:133999610	rs142160158	R	I	0.77	3.54E-01	-0.2107	0.2271	1.23E-01	0.4486	0.2991	3.73E-05	0.8657	0.1935
8:134002609	rs10099487	T	C	0.78	3.89E-01	-0.1993	0.2311	1.28E-01	0.4555	0.3078	5.47E-05	0.8637	0.1980

8:134003063	rs7844859	C	G	0.78	3.88E-01	-0.1997	0.2312	1.28E-01	0.4564	0.3083	5.40E-05	0.8648	0.1981
8:134003128	rs7844761	A	G	0.78	3.86E-01	-0.2022	0.2328	1.75E-01	0.4095	0.3078	5.45E-05	0.8703	0.1995
8:134003752	rs28406375	C	T	0.78	3.86E-01	-0.2007	0.2312	1.25E-01	0.4602	0.3088	5.31E-05	0.8657	0.1981
8:134003923	rs28547062	T	C	0.78	3.87E-01	-0.2005	0.2313	1.26E-01	0.4595	0.3089	5.30E-05	0.8660	0.1981
8:134004012	rs55669707	C	G	0.78	3.86E-01	-0.2007	0.2313	1.26E-01	0.4600	0.3090	5.29E-05	0.8661	0.1981
8:134009434	rs28758516	G	C	0.78	3.14E-01	-0.2351	0.2327	1.07E-01	0.4906	0.3130	3.41E-05	0.8922	0.1983
8:134010608	rs17693464	G	A	0.78	3.88E-01	-0.2018	0.2337	1.57E-01	0.4331	0.3125	4.56E-05	0.8817	0.1997
8:134011224	rs7842044	T	C	0.78	3.88E-01	-0.2019	0.2338	1.57E-01	0.4330	0.3127	4.56E-05	0.8818	0.1997
8:134011389	rs72727479	G	C	0.78	3.83E-01	-0.2032	0.2325	1.14E-01	0.4810	0.3136	4.60E-05	0.8766	0.1987
8:134011395	rs56171639	G	A	0.78	3.83E-01	-0.2030	0.2325	1.14E-01	0.4813	0.3137	4.62E-05	0.8765	0.1987
8:134012038	rs10102602	A	G	0.78	3.87E-01	-0.2026	0.2339	1.55E-01	0.4367	0.3136	4.55E-05	0.8825	0.1999
8:134013470	rs34052396	R	I	0.78	3.85E-01	-0.2035	0.2340	1.52E-01	0.4407	0.3143	4.56E-05	0.8828	0.1999
8:134014795	rs2403911	C	T	0.78	3.81E-01	-0.2040	0.2326	1.09E-01	0.4907	0.3158	4.73E-05	0.8759	0.1989
8:13889381	rs6530707	C	T	0.80	7.06E-05	0.9220	0.2164	2.88E-02	-0.5985	0.2699	5.22E-01	-0.1569	0.2439
8:13890188	rs34647371	G	A	0.80	7.11E-05	0.9147	0.2148	2.94E-02	-0.5939	0.2688	5.12E-01	-0.1595	0.2420
8:13893837	rs75126334	C	T	0.80	5.33E-05	0.9419	0.2169	2.55E-02	-0.6161	0.2718	4.41E-01	-0.1901	0.2452
8:14684548	rs143299274	G	C	0.93	3.09E-01	0.4024	0.3949	8.67E-05	-1.3017	0.2944	7.19E-01	-0.1414	0.3914
8:14686468	rs535270610	R	I	0.93	3.12E-01	0.4008	0.3956	9.19E-05	-1.3023	0.2956	6.91E-01	-0.1566	0.3919
8:14687259	rs138768888	R	I	0.95	8.65E-02	0.7724	0.4456	6.03E-05	-1.3571	0.2965	3.67E-01	-0.4063	0.4465
8:14687689	rs11997535	T	G	0.95	8.63E-02	0.7730	0.4457	6.03E-05	-1.3574	0.2965	3.66E-01	-0.4065	0.4466
8:14687767	rs552564510	R	I	0.95	6.99E-02	0.8151	0.4440	6.68E-05	-1.3532	0.2974	3.29E-01	-0.4389	0.4456
8:14688188	rs17120379	T	G	0.95	8.36E-02	0.7802	0.4458	5.74E-05	-1.3605	0.2964	3.69E-01	-0.4046	0.4469
8:14688728	rs7824647	G	T	0.95	8.19E-02	0.7896	0.4487	6.27E-05	-1.3587	0.2977	3.51E-01	-0.4225	0.4497
8:14689168	rs76314409	T	C	0.95	8.14E-02	0.7902	0.4482	6.20E-05	-1.3581	0.2973	3.57E-01	-0.4172	0.4493
8:14691229	rs73535030	T	A	0.95	8.05E-02	0.7934	0.4488	6.24E-05	-1.3584	0.2975	3.55E-01	-0.4197	0.4499
8:14691675	rs73535031	A	T	0.95	8.04E-02	0.7939	0.4489	6.24E-05	-1.3585	0.2976	3.55E-01	-0.4197	0.4500
8:14691726	rs17120394	T	C	0.95	8.05E-02	0.7937	0.4489	6.24E-05	-1.3584	0.2976	3.55E-01	-0.4193	0.4500
8:14691926	rs12677960	A	C	0.95	8.12E-02	0.7913	0.4486	6.20E-05	-1.3588	0.2975	3.56E-01	-0.4188	0.4497
8:14692096	rs73535033	A	C	0.95	8.48E-02	0.7787	0.4468	6.08E-05	-1.3582	0.2969	3.63E-01	-0.4103	0.4477
8:14692456	rs6999273	C	A	0.93	2.51E-01	0.4612	0.4004	6.78E-05	-1.3310	0.2973	6.24E-01	-0.1958	0.3975
8:14692823	rs6530809	A	T	0.95	8.19E-02	0.7911	0.4495	6.31E-05	-1.3602	0.2982	3.49E-01	-0.4254	0.4505
8:14692938	rs7830186	C	T	0.95	8.23E-02	0.7916	0.4505	6.40E-05	-1.3628	0.2990	3.63E-01	-0.4138	0.4516
8:15387804	rs13273455	C	G	0.61	8.76E-05	0.6430	0.1532	4.60E-02	-0.4402	0.2186	6.99E-04	-0.5591	0.1559
8:15469237	rs1366667	G	A	0.67	9.27E-05	-0.8237	0.1970	1.21E-01	0.4246	0.2757	2.31E-02	0.4948	0.2119

8:15469662	rs13249988	A	T	0.67	9.09E-05	-0.8219	0.1963	1.18E-01	0.4263	0.2749	2.30E-02	0.4934	0.2112
8:15470729	rs17577614	A	G	0.47	2.04E-05	0.7975	0.1727	3.36E-02	-0.5705	0.2683	9.23E-03	-0.5062	0.1878
8:15474579	rs17612219	A	G	0.65	5.10E-05	-0.8146	0.1870	1.64E-01	0.3664	0.2647	7.30E-02	0.3776	0.2067
8:15474869	rs80095027	G	A	0.65	4.98E-05	-0.8153	0.1869	1.65E-01	0.3653	0.2647	7.27E-02	0.3778	0.2067
8:15475002	rs12547644	A	T	0.67	8.02E-05	-0.8218	0.1946	1.18E-01	0.4222	0.2726	2.09E-02	0.4977	0.2095
8:15476922	rs13281940	G	A	0.65	4.89E-05	-0.8160	0.1868	1.61E-01	0.3685	0.2647	7.16E-02	0.3792	0.2066
8:15479013	rs35411066	R	D	0.65	4.97E-05	-0.8137	0.1865	1.58E-01	0.3705	0.2642	7.10E-02	0.3792	0.2061
8:15479074	rs6988217	A	T	0.65	4.96E-05	-0.8136	0.1865	1.58E-01	0.3706	0.2641	7.10E-02	0.3792	0.2061
8:15480039	rs1429079	A	G	0.65	4.89E-05	-0.8130	0.1862	1.55E-01	0.3722	0.2637	7.02E-02	0.3797	0.2058
8:15484801	rs13275624	C	T	0.67	7.67E-05	-0.8182	0.1932	1.08E-01	0.4290	0.2698	1.65E-02	0.5120	0.2073
8:15485371	rs11777204	G	C	0.65	4.56E-05	-0.8111	0.1849	1.46E-01	0.3771	0.2616	6.05E-02	0.3910	0.2042
8:15486898	rs13260667	C	T	0.65	4.52E-05	-0.8113	0.1848	1.44E-01	0.3788	0.2614	5.95E-02	0.3924	0.2040
8:15487674	rs9693652	T	A	0.65	4.30E-05	-0.8135	0.1847	1.42E-01	0.3819	0.2619	6.45E-02	0.3853	0.2043
8:15487843	rs35130332	A	G	0.53	6.26E-05	0.7518	0.1750	3.43E-01	-0.2428	0.2586	1.30E-02	-0.4813	0.1878
8:15487936	rs9694476	G	C	0.65	4.34E-05	-0.8124	0.1846	1.44E-01	0.3790	0.2612	5.91E-02	0.3927	0.2039
8:15488656	rs1864236	C	A	0.65	4.31E-05	-0.8123	0.1845	1.38E-01	0.3838	0.2611	5.81E-02	0.3940	0.2038
8:15490890	rs9650388	T	A	0.66	7.34E-05	-0.8174	0.1924	1.07E-01	0.4290	0.2686	1.63E-02	0.5112	0.2066
8:15491989	rs12541025	G	A	0.66	7.13E-05	-0.8167	0.1919	9.85E-02	0.4384	0.2683	1.58E-02	0.5126	0.2060
8:15492991	rs35965940	C	G	0.65	4.48E-05	-0.8126	0.1850	1.46E-01	0.3773	0.2612	5.88E-02	0.3938	0.2043
8:15505952	rs7012084	T	C	0.67	7.94E-05	-0.8016	0.1897	8.03E-02	0.4601	0.2672	4.68E-02	0.4201	0.2067
8:15506023	rs6992252	C	G	0.67	7.94E-05	-0.8017	0.1897	8.03E-02	0.4601	0.2672	4.69E-02	0.4200	0.2068
8:15506440	rs34689098	T	C	0.67	7.92E-05	-0.8017	0.1897	8.02E-02	0.4602	0.2672	4.69E-02	0.4199	0.2067
8:15508200	rs1035972	T	C	0.67	8.50E-05	-0.8020	0.1907	7.90E-02	0.4626	0.2675	4.68E-02	0.4219	0.2076
8:15513987	rs35199368	G	C	0.67	8.42E-05	-0.8026	0.1907	7.35E-02	0.4711	0.2676	4.61E-02	0.4232	0.2076
8:15569376	rs10102177	T	G	0.63	2.30E-05	-0.8076	0.1762	6.83E-02	0.4596	0.2545	4.33E-02	0.4048	0.1959
8:15576035	rs1421254	G	C	0.64	3.26E-05	-0.8032	0.1791	6.51E-02	0.4668	0.2557	4.31E-02	0.4095	0.1979
8:15596335	rs2017708	G	T	0.64	6.04E-05	-0.8022	0.1863	4.23E-02	0.5261	0.2630	4.98E-02	0.4092	0.2042
8:15608896	rs751687	A	G	0.48	8.26E-05	0.7408	0.1758	2.83E-02	-0.5749	0.2608	1.20E-01	-0.3063	0.1941
8:15628324	rs7842027	G	A	0.47	5.01E-05	0.7615	0.1747	1.04E-02	-0.6567	0.2543	1.13E-01	-0.3129	0.1943
8:15629658	rs12682188	T	C	0.50	8.25E-06	0.8264	0.1696	1.01E-03	-0.8472	0.2508	6.06E-02	-0.3691	0.1928
8:15631755	rs137982947	R	D	0.50	1.51E-05	0.8125	0.1728	9.34E-04	-0.8540	0.2512	6.51E-02	-0.3660	0.1946
8:15633195	rs72359824	R	D	0.57	3.87E-05	0.8504	0.1918	5.72E-02	-0.5232	0.2813	9.74E-03	-0.5523	0.2065
8:1925843	rs7386187	C	G	0.80	3.61E-01	0.2538	0.2774	1.83E-02	-0.8634	0.3559	6.02E-05	-1.0327	0.2383
8:1925864	rs11365795	D	R	0.80	3.63E-01	0.2528	0.2777	1.81E-02	-0.8654	0.3562	5.56E-05	-1.0379	0.2382

8:1926880	rs11782830	T	G	0.80	3.51E-01	0.2605	0.2786	2.65E-02	-0.8177	0.3596	3.77E-05	-1.0619	0.2376
8:1926888	rs11136450	A	G	0.80	3.51E-01	0.2606	0.2787	2.65E-02	-0.8178	0.3596	3.76E-05	-1.0621	0.2376
8:1926911	rs11776183	G	C	0.80	3.37E-01	0.2667	0.2770	1.77E-02	-0.8660	0.3556	3.76E-05	-1.0564	0.2363
8:1926917	rs11782860	C	G	0.80	3.37E-01	0.2666	0.2770	1.77E-02	-0.8660	0.3555	3.79E-05	-1.0559	0.2363
8:1926958	rs13271843	T	C	0.80	3.40E-01	0.2643	0.2763	1.77E-02	-0.8651	0.3552	3.97E-05	-1.0507	0.2358
8:1927012	rs33962631	D	R	0.80	3.39E-01	0.2643	0.2762	1.74E-02	-0.8666	0.3549	3.87E-05	-1.0516	0.2357
8:1927209	rs12675904	G	C	0.80	3.39E-01	0.2646	0.2762	1.74E-02	-0.8673	0.3549	3.83E-05	-1.0521	0.2356
8:1927244	rs12678015	G	A	0.80	3.35E-01	0.2666	0.2762	1.70E-02	-0.8701	0.3551	3.56E-05	-1.0562	0.2354
8:1927353	rs12679875	C	G	0.80	3.36E-01	0.2661	0.2762	1.71E-02	-0.8697	0.3550	3.63E-05	-1.0551	0.2355
8:1927410	rs12679898	A	G	0.80	3.34E-01	0.2672	0.2762	1.69E-02	-0.8711	0.3551	3.48E-05	-1.0575	0.2353
8:1927524	rs11779933	C	A	0.80	3.34E-01	0.2675	0.2762	1.69E-02	-0.8716	0.3551	3.44E-05	-1.0579	0.2353
8:1927576	rs13274738	C	A	0.80	3.34E-01	0.2676	0.2763	1.69E-02	-0.8717	0.3552	3.43E-05	-1.0582	0.2353
8:1927617	rs13278691	A	G	0.80	3.34E-01	0.2677	0.2763	1.68E-02	-0.8718	0.3552	3.42E-05	-1.0583	0.2353
8:1927799	rs11777154	G	C	0.80	3.33E-01	0.2680	0.2763	1.68E-02	-0.8724	0.3552	3.37E-05	-1.0591	0.2352
8:1927821	rs11781392	C	T	0.80	3.33E-01	0.2681	0.2763	1.68E-02	-0.8725	0.3552	3.36E-05	-1.0593	0.2352
8:1927822	rs11783857	C	G	0.80	3.33E-01	0.2682	0.2763	1.68E-02	-0.8725	0.3552	3.37E-05	-1.0592	0.2353
8:1927828	rs11781393	C	T	0.80	3.33E-01	0.2681	0.2763	1.68E-02	-0.8725	0.3553	3.39E-05	-1.0589	0.2353
8:1927947	rs11783884	A	G	0.80	3.33E-01	0.2680	0.2764	1.69E-02	-0.8725	0.3555	3.48E-05	-1.0582	0.2355
8:1928033	rs34929443	G	A	0.80	3.34E-01	0.2678	0.2766	1.69E-02	-0.8726	0.3558	3.58E-05	-1.0575	0.2358
8:1928054	rs17064479	G	C	0.80	3.35E-01	0.2676	0.2767	1.70E-02	-0.8724	0.3559	3.61E-05	-1.0572	0.2358
8:1928333	rs34806713	A	T	0.80	3.33E-01	0.2684	0.2769	1.70E-02	-0.8729	0.3562	3.69E-05	-1.0570	0.2361
8:1928424	rs9657383	C	T	0.80	3.35E-01	0.2673	0.2768	1.71E-02	-0.8724	0.3562	3.76E-05	-1.0558	0.2361
8:1928444	rs9657384	T	G	0.80	3.35E-01	0.2674	0.2769	1.71E-02	-0.8725	0.3563	3.77E-05	-1.0557	0.2362
8:1928577	rs9657385	G	C	0.80	3.35E-01	0.2677	0.2770	1.72E-02	-0.8727	0.3566	3.88E-05	-1.0549	0.2364
8:1928921	rs10102560	C	G	0.80	3.37E-01	0.2671	0.2775	1.72E-02	-0.8742	0.3572	4.27E-05	-1.0516	0.2372
8:1928953	rs10102569	C	G	0.80	3.37E-01	0.2671	0.2774	1.72E-02	-0.8740	0.3572	4.26E-05	-1.0516	0.2371
8:1929523	rs7387430	C	T	0.81	4.18E-01	0.2263	0.2794	4.63E-02	-0.7564	0.3673	2.55E-05	-1.0824	0.2362
8:1929596	rs7387163	C	A	0.80	3.39E-01	0.2661	0.2775	1.76E-02	-0.8712	0.3574	4.45E-05	-1.0495	0.2373
8:23433511	rs12545866	A	C	0.63	2.99E-02	-0.3985	0.1800	7.23E-01	0.0794	0.2309	5.41E-05	0.6974	0.1598
8:25968591	rs2976330	T	C	0.45	3.76E-05	0.8590	0.1933	2.74E-01	-0.3072	0.2775	2.17E-01	-0.2724	0.2180
8:27112733	rs573832758	R	I	0.84	3.54E-05	1.0650	0.2388	7.40E-02	-0.5343	0.2735	1.31E-01	-0.4107	0.2677
8:27424988	rs1532275	G	A	0.93	2.78E-01	0.4154	0.3814	1.25E-01	-0.6702	0.4193	7.28E-05	-1.4105	0.3297
8:27444277	rs881146	A	G	0.93	2.64E-01	0.4335	0.3866	1.23E-01	-0.6822	0.4239	6.28E-05	-1.4415	0.3336
8:30583904	rs8190893	C	T	0.94	1.04E-01	0.7794	0.4742	6.17E-01	0.2847	0.5745	8.89E-05	-1.7581	0.4167

8:57690127	rs10543737	D	R	0.52	8.42E-05	-0.7290	0.1732	5.35E-03	0.6106	0.2091	2.98E-02	0.4174	0.1873
8:67369551	rs34743572	R	I	0.75	8.98E-05	-1.1095	0.2648	2.84E-01	0.4207	0.3928	1.26E-01	0.4532	0.2922
8:6907268	rs114300314	C	G	0.88	8.25E-02	0.4779	0.2722	4.46E-05	-1.0466	0.2348	4.62E-01	-0.2026	0.2735
8:6913125	rs45613133	C	A	0.88	1.83E-01	0.3909	0.2918	8.15E-05	-1.1039	0.2578	5.57E-01	-0.1716	0.2905
8:6915645	rs80104006	A	G	0.86	1.58E-01	0.4295	0.3022	1.84E-05	-1.2392	0.2743	5.88E-01	-0.1644	0.3016
8:69732284	rs2958329	G	A	0.46	2.66E-05	-0.7305	0.1608	1.72E-01	0.3223	0.2392	1.30E-02	0.4487	0.1751
8:69732811	rs12155640	C	G	0.42	5.01E-07	-0.8729	0.1550	4.43E-01	-0.2960	0.3846	7.98E-01	-0.0709	0.2756
8:69737609	rs2978235	C	T	0.42	4.93E-07	-0.8658	0.1536	1.17E-01	0.3937	0.2536	6.14E-02	0.3495	0.1832
8:69741018	rs2978234	A	G	0.42	4.37E-07	-0.8609	0.1519	1.16E-01	0.3897	0.2504	5.69E-02	0.3524	0.1813
8:69741316	rs2978233	G	A	0.43	4.15E-07	-0.8597	0.1513	1.18E-01	0.3872	0.2498	5.65E-02	0.3518	0.1807
8:74383408	rs35701266	R	I	0.80	1.91E-01	-0.4138	0.3148	9.82E-05	1.5758	0.4281	7.74E-01	0.0906	0.3140
8:75049687	rs61637679	D	R	0.58	9.70E-03	-0.4841	0.1820	1.51E-02	0.6154	0.2395	8.49E-05	0.7011	0.1656
8:98910894	rs72675237	C	T	0.93	2.58E-01	-0.4550	0.4006	7.42E-05	37.5170	2.23E+07	3.34E-01	0.3851	0.3951
8:98911550	rs72675238	T	C	0.93	2.57E-01	-0.4556	0.4007	7.43E-05	11.1491	74.3286	3.34E-01	0.3854	0.3952
8:98911571	rs72675240	C	T	0.93	2.58E-01	-0.4549	0.4009	7.15E-05	28.2744	113.2900	3.34E-01	0.3851	0.3953
8:98913979	rs72675242	G	A	0.93	2.54E-01	-0.4584	0.4004	7.56E-05	8.7473	22.5776	3.33E-01	0.3857	0.3950
8:98915478	rs149199242	R	D	0.93	2.54E-01	-0.4587	0.4003	5.62E-05	175.7990	241.3660	3.33E-01	0.3856	0.3949
8:98915826	rs4734378	A	G	0.93	2.54E-01	-0.4584	0.4003	6.36E-05	170.8840	316.1490	3.33E-01	0.3856	0.3949
8:98917056	rs11430670	R	I	0.93	2.53E-01	-0.4593	0.4004	5.81E-05	153.7750	230.8140	3.33E-01	0.3853	0.3949
8:98917058	rs78374144	T	A	0.93	2.53E-01	-0.4593	0.4004	5.81E-05	153.7750	230.8140	3.33E-01	0.3853	0.3949
8:98917811	rs4735508	G	A	0.93	2.54E-01	-0.4582	0.4004	7.53E-05	8.8780	23.6598	3.33E-01	0.3857	0.3950
8:98918056	rs111687902	T	A	0.93	2.58E-01	-0.4553	0.4007	4.75E-05	569.2470	634.0790	3.34E-01	0.3850	0.3952
8:98919268	rs113625770	G	A	0.93	2.57E-01	-0.4563	0.4007	5.61E-05	266.6210	383.8000	3.34E-01	0.3850	0.3952
8:98919626	rs147020105	A	G	0.93	2.58E-01	-0.4553	0.4007	4.75E-05	569.2470	634.0790	3.34E-01	0.3850	0.3952
8:98920951	rs112018057	C	T	0.93	2.58E-01	-0.4553	0.4007	4.75E-05	569.2470	634.0790	3.34E-01	0.3850	0.3952
8:98927810	rs72675251	G	A	0.93	2.57E-01	-0.4560	0.4005	7.42E-05	35.9150	1.78E+07	3.33E-01	0.3854	0.3950
8:98927849	rs72675254	T	C	0.93	2.58E-01	-0.4548	0.4007	8.06E-05	7.0867	9.8132	3.30E-01	0.3884	0.3951
8:98930702	rs753800	A	G	0.93	2.56E-01	-0.4562	0.4005	7.42E-05	40.8971	2.14E+07	3.33E-01	0.3855	0.3950
9:12287198	rs10465038	T	C	0.71	3.21E-05	1.0848	0.2417	6.91E-02	-0.5939	0.3346	8.38E-02	-0.4747	0.2697
9:12287871	rs1416582	G	C	0.72	7.71E-05	1.0264	0.2424	1.35E-01	-0.4853	0.3339	7.24E-02	-0.4869	0.2660
9:12288078	rs10960566	A	G	0.70	5.03E-05	1.0668	0.2447	1.02E-01	-0.5117	0.3226	1.38E-01	-0.4107	0.2730
9:12289527	rs7020196	T	C	0.56	7.89E-05	0.8841	0.2091	1.40E-01	-0.4172	0.2816	1.62E-02	-0.5557	0.2243
9:12290310	rs12551805	T	C	0.71	2.20E-05	1.0586	0.2303	1.29E-01	-0.4882	0.3312	6.34E-02	-0.4878	0.2576
9:12290333	rs12555542	C	T	0.71	2.63E-05	1.0781	0.2372	1.19E-01	-0.5092	0.3361	5.72E-02	-0.5128	0.2641

9:12290653	rs10960567	T	C	0.71	2.63E-05	1.0773	0.2370	1.18E-01	-0.5089	0.3359	5.70E-02	-0.5128	0.2639
9:12290837	rs11794979	G	T	0.73	3.74E-05	1.0508	0.2364	1.15E-01	-0.5123	0.3343	4.37E-02	-0.5376	0.2607
9:122926890	rs576745861	R	D	0.44	6.05E-01	-0.1471	0.2849	8.95E-05	1.1493	0.2878	4.55E-02	0.5541	0.2710
9:12538480	rs10960701	C	A	0.83	4.53E-02	0.5275	0.2594	3.53E-05	-1.2602	0.2992	7.69E-01	0.0780	0.2640
9:12539122	rs72700779	G	A	0.83	4.68E-02	0.5227	0.2589	3.74E-05	-1.2535	0.2985	7.42E-01	0.0871	0.2633
9:12539582	rs59860343	G	A	0.83	4.43E-02	0.5289	0.2588	3.46E-05	-1.2599	0.2990	7.68E-01	0.0780	0.2635
9:12539719	rs116720470	C	T	0.83	4.62E-02	0.5235	0.2585	3.73E-05	-1.2525	0.2984	7.42E-01	0.0870	0.2630
9:12540358	rs12235745	G	A	0.83	4.61E-02	0.5237	0.2584	3.73E-05	-1.2523	0.2984	7.42E-01	0.0869	0.2630
9:125542991	rs33965089	R	I	0.80	1.52E-02	0.8333	0.3350	6.16E-01	-0.2145	0.4267	4.66E-05	-1.3211	0.2997
9:12561164	rs12000212	T	A	0.83	4.91E-02	0.5235	0.2621	3.74E-05	-1.2708	0.3027	8.30E-01	0.0576	0.2665
9:13479775	rs56412453	A	G	0.65	2.11E-01	0.2901	0.2306	7.55E-05	-1.0192	0.2541	5.60E-02	-0.4341	0.2226
9:138274187	rs12238007	A	G	0.85	1.27E-01	-0.4951	0.3221	7.29E-05	1.7396	0.4940	4.90E-01	0.2238	0.3219
9:138740350	rs35091764	R	D	0.80	2.39E-02	0.5593	0.2425	9.57E-05	-0.7979	0.1892	3.73E-02	-0.5118	0.2401
9:138742077	rs35981196	C	G	0.82	3.42E-02	0.5248	0.2434	9.29E-05	-0.7879	0.1866	5.24E-02	-0.4771	0.2408
9:138744527	rs56988536	C	T	0.82	3.42E-02	0.5260	0.2440	9.40E-05	-0.7892	0.1868	5.26E-02	-0.4780	0.2415
9:138746319	rs111395128	R	I	0.82	3.78E-02	0.5230	0.2476	9.75E-05	-0.7946	0.1876	5.72E-02	-0.4753	0.2449
9:138748802	rs1929527	A	G	0.82	3.44E-02	0.5287	0.2455	9.93E-05	-0.7904	0.1872	5.36E-02	-0.4789	0.2430
9:138760015	rs372013273	R	I	0.81	1.33E-02	0.6449	0.2540	6.03E-05	-0.8370	0.1915	7.29E-02	-0.4682	0.2562
9:20196505	rs1456958	G	T	0.76	6.91E-05	-0.9057	0.2123	3.12E-01	0.3255	0.3332	2.01E-01	0.3062	0.2367
9:22154664	rs7046289	C	T	0.59	5.00E-05	0.8182	0.1876	2.51E-01	-0.3179	0.2690	1.19E-02	-0.5245	0.2018
9:22154682	rs7029976	T	A	0.55	7.84E-05	0.8556	0.2023	2.17E-01	-0.3602	0.2846	4.26E-03	-0.6325	0.2124
9:22154684	rs7046298	C	T	0.55	7.84E-05	0.8556	0.2023	2.18E-01	-0.3599	0.2846	4.27E-03	-0.6324	0.2124
9:25499673	rs10966936	A	T	0.58	5.06E-01	0.1475	0.2218	3.37E-01	-0.2620	0.2756	9.95E-05	-0.8016	0.1915
9:25500174	rs10966937	C	T	0.58	5.03E-01	0.1486	0.2217	3.24E-01	-0.2692	0.2754	9.70E-05	-0.8022	0.1913
9:25500909	rs1806305	C	A	0.57	5.05E-01	0.1442	0.2164	2.60E-01	-0.2987	0.2655	9.20E-05	-0.7852	0.1866
9:36231127	rs201933111	R	D	0.74	9.22E-03	0.8365	0.3123	1.01E-06	-1.4628	0.2753	2.43E-02	-0.7211	0.3118
9:36478814	rs58286962	D	R	0.69	1.05E-01	0.3642	0.2227	7.41E-05	-0.9075	0.2175	7.83E-01	-0.0619	0.2239
9:36480711	rs71531226	G	C	0.74	6.45E-02	0.4136	0.2208	4.58E-05	-0.9035	0.2105	7.88E-01	-0.0603	0.2236
9:78028974	rs200939903	R	D	0.90	1.69E-01	0.4581	0.3306	7.78E-03	-0.9400	0.3152	5.10E-05	-1.2528	0.2859
9:78036584	rs13294994	C	T	0.93	5.89E-01	0.1948	0.3610	7.23E-02	-0.7147	0.3643	9.42E-05	-1.3066	0.3110
9:86015579	rs4877758	G	T	0.78	1.73E-01	0.3912	0.2853	2.22E-01	-0.4130	0.3393	4.19E-05	-1.0913	0.2458
9:92863041	rs10992083	T	C	0.74	2.06E-02	0.5299	0.2239	5.67E-05	-0.8727	0.2035	1.19E-01	-0.3575	0.2259
X:122365098	rs5911554	C	T	0.77	4.13E-01	0.1535	0.1873	6.66E-01	-0.0924	0.2154	4.11E-05	-0.7097	0.1597
X:122365147	rs5911555	G	A	0.77	4.06E-01	0.1573	0.1893	6.44E-01	-0.0999	0.2177	4.36E-05	-0.7151	0.1615

X:122369317	rs1476014	A	G	0.77	4.18E-01	0.1525	0.1881	6.59E-01	-0.0949	0.2163	5.08E-05	-0.7050	0.1608
X:122370414	rs10594525	D	R	0.77	4.25E-01	0.1499	0.1879	6.61E-01	-0.0941	0.2160	5.53E-05	-0.7015	0.1609
X:122394372	rs189865332	A	G	0.72	2.74E-01	0.2126	0.1937	7.51E-01	0.0708	0.2206	7.30E-05	-0.7164	0.1675
X:137943387	rs7060413	G	A	0.72	9.63E-01	0.0087	0.1912	1.97E-01	-0.2810	0.2184	1.11E-05	-0.7635	0.1585
X:139917555	rs5907696	C	T	0.87	2.26E-01	-0.2996	0.2466	8.86E-05	1.8675	0.7708	7.29E-02	0.4362	0.2387
X:146121482	N/A	R	D	0.94	4.37E-01	0.5327	0.6842	7.82E-05	-2.0210	0.4513	2.11E-01	-0.8431	0.6671
X:3548751	rs2398586	C	G	0.50	2.52E-05	-0.8388	0.1840	7.93E-02	0.4579	0.2616	2.56E-02	0.4644	0.2026
X:38877272	rs2223159	T	C	0.72	4.81E-01	-0.1448	0.2054	9.60E-01	0.0111	0.2242	8.78E-05	-0.7477	0.1771
X:40467447	rs199896654	I	R	0.83	9.18E-05	-0.8942	0.2138	2.12E-01	0.4593	0.3854	3.12E-01	0.2432	0.2385
X:40475498	rs72623243	A	G	0.81	9.38E-05	-0.8480	0.2030	2.09E-01	0.4404	0.3643	2.90E-01	0.2417	0.2262
X:54500421	rs5961069	T	A	0.78	5.05E-01	0.1555	0.2333	2.51E-01	-0.3185	0.2792	5.91E-05	-0.8663	0.1996
X:57120828	rs5960898	G	A	0.80	4.93E-01	0.1405	0.2049	9.76E-01	0.0075	0.2445	9.43E-05	-0.7426	0.1767
X:57122570	rs10481897	C	T	0.81	5.00E-01	0.1444	0.2138	9.76E-01	0.0077	0.2545	9.26E-05	-0.7757	0.1844
X:57189999	rs139635869	C	T	0.80	5.20E-01	0.1318	0.2049	9.80E-01	-0.0061	0.2441	6.04E-05	-0.7599	0.1754
X:57193835	rs28730481	A	G	0.81	5.25E-01	0.1359	0.2137	9.82E-01	-0.0057	0.2540	6.07E-05	-0.7922	0.1829
X:57241324	rs5960927	G	A	0.80	5.29E-01	0.1279	0.2033	9.81E-01	-0.0057	0.2420	5.88E-05	-0.7547	0.1739
X:57326746	rs151008828	A	G	0.80	5.04E-01	0.1411	0.2113	9.60E-01	-0.0127	0.2531	5.49E-05	-0.7875	0.1806
X:57362156	rs112562331	A	T	0.80	4.96E-01	0.1463	0.2149	9.52E-01	-0.0155	0.2581	5.30E-05	-0.8025	0.1836
X:57421091	rs200105825	R	D	0.81	4.98E-01	0.1552	0.2289	9.45E-01	-0.0192	0.2754	5.51E-05	-0.8531	0.1957
X:57469183	rs56092657	G	C	0.89	5.21E-01	0.2426	0.3781	9.37E-01	-0.0384	0.4776	4.75E-05	-1.4194	0.3223
X:57513729	rs373236218	R	D	0.80	4.89E-01	0.1548	0.2236	9.37E-01	-0.0218	0.2725	5.21E-05	-0.8357	0.1910
X:57543583	rs12392773	C	A	0.81	4.51E-01	0.1774	0.2352	9.03E-01	-0.0350	0.2870	5.64E-05	-0.8766	0.2014
X:57580906	rs73226014	G	A	0.81	4.53E-01	0.1785	0.2380	9.00E-01	-0.0371	0.2915	5.90E-05	-0.8848	0.2039
X:57693862	rs5961028	A	G	0.81	4.92E-01	0.1613	0.2349	9.29E-01	-0.0256	0.2862	6.55E-05	-0.8678	0.2014
X:57705777	rs73226022	G	T	0.80	4.81E-01	0.1607	0.2278	9.28E-01	-0.0255	0.2781	6.76E-05	-0.8405	0.1955
X:57718471	rs73226025	A	G	0.81	4.90E-01	0.1633	0.2366	9.31E-01	-0.0252	0.2888	7.26E-05	-0.8697	0.2032
X:57802424	rs5988719	C	T	0.80	5.01E-01	0.1518	0.2253	9.24E-01	-0.0267	0.2760	9.30E-05	-0.8173	0.1943
X:57955169	rs73226045	G	A	0.80	4.52E-01	0.1579	0.2101	8.65E-01	-0.0441	0.2578	8.41E-05	-0.7669	0.1811
X:57995561	rs5947383	G	T	0.54	9.26E-01	0.0176	0.1912	1.77E-02	-0.5842	0.2429	9.90E-05	-0.6882	0.1644
X:58021668	rs73209406	A	T	0.80	4.98E-01	0.1448	0.2139	9.23E-01	-0.0258	0.2659	6.45E-05	-0.7910	0.1834
X:58052229	rs5988699	A	G	0.80	6.07E-01	0.1139	0.2219	9.68E-01	0.0115	0.2805	7.69E-05	-0.8118	0.1905
X:88972248	rs5940734	A	G	0.55	3.75E-02	-0.3481	0.1645	6.89E-05	0.8467	0.2194	8.84E-01	0.0245	0.1680
X:88972361	rs5940735	T	C	0.55	3.73E-02	-0.3483	0.1644	6.87E-05	0.8466	0.2193	8.84E-01	0.0246	0.1679
X:88972637	rs5941306	C	T	0.56	3.68E-02	-0.3482	0.1639	6.73E-05	0.8464	0.2192	8.77E-01	0.0261	0.1675

X:88973755	rs11799023	G	A	0.55	3.39E-02	-0.3502	0.1622	6.40E-05	0.8432	0.2178	8.59E-01	0.0297	0.1659
X:88974248	rs5984371	G	A	0.55	3.31E-02	-0.3503	0.1614	6.38E-05	0.8413	0.2174	8.59E-01	0.0295	0.1652
X:88974449	rs5984372	G	A	0.55	3.29E-02	-0.3506	0.1614	6.39E-05	0.8414	0.2175	8.58E-01	0.0296	0.1652
X:88976964	rs112138412	C	T	0.55	3.15E-02	-0.3515	0.1604	6.17E-05	0.8396	0.2167	8.51E-01	0.0310	0.1642
X:88977243	rs143182906	C	T	0.55	3.13E-02	-0.3516	0.1603	6.18E-05	0.8393	0.2166	8.51E-01	0.0310	0.1642
X:88977421	rs77033100	A	C	0.60	4.15E-02	-0.3645	0.1759	7.75E-05	0.8830	0.2310	8.46E-01	0.0351	0.1794
X:88977800	rs149903691	T	C	0.55	3.11E-02	-0.3515	0.1600	6.11E-05	0.8387	0.2164	8.47E-01	0.0318	0.1639
X:88978080	N/A	C	A	0.55	3.09E-02	-0.3517	0.1599	6.07E-05	0.8388	0.2163	8.46E-01	0.0319	0.1638
X:88978081	N/A	T	C	0.55	3.09E-02	-0.3517	0.1599	6.07E-05	0.8387	0.2163	8.46E-01	0.0319	0.1638
X:88978213	rs201473310	T	G	0.58	4.56E-02	-0.3427	0.1688	6.00E-05	0.8981	0.2326	9.54E-01	0.0099	0.1719
X:88979181	rs181389657	G	A	0.55	3.06E-02	-0.3518	0.1597	6.05E-05	0.8381	0.2162	8.46E-01	0.0319	0.1636
X:88979334	rs5941312	T	G	0.55	3.04E-02	-0.3520	0.1596	6.03E-05	0.8381	0.2161	8.45E-01	0.0321	0.1635
X:88979493	rs2498105	A	G	0.59	4.87E-02	-0.3420	0.1709	7.48E-05	0.8833	0.2309	9.77E-01	0.0051	0.1739
X:88979554	rs5984373	C	T	0.57	4.73E-02	-0.3350	0.1663	7.54E-05	0.8612	0.2248	9.57E-01	0.0091	0.1693
X:88979812	rs5984375	C	G	0.55	3.03E-02	-0.3523	0.1596	5.90E-05	0.8388	0.2161	8.46E-01	0.0319	0.1635
X:88979839	rs2498106	T	G	0.55	3.02E-02	-0.3528	0.1597	5.78E-05	0.8414	0.2166	8.47E-01	0.0318	0.1637
X:88980030	rs5941313	T	C	0.55	3.01E-02	-0.3522	0.1594	5.98E-05	0.8379	0.2160	8.44E-01	0.0322	0.1633
X:88980851	rs62594581	G	T	0.55	2.82E-02	-0.3529	0.1577	5.69E-05	0.8343	0.2147	8.31E-01	0.0348	0.1618
X:88981132	rs199525938	G	T	0.55	2.82E-02	-0.3528	0.1577	5.71E-05	0.8340	0.2147	8.31E-01	0.0347	0.1617
X:88981890	rs2498110	A	G	0.58	3.70E-02	-0.3502	0.1650	6.42E-05	0.8743	0.2275	9.30E-01	0.0150	0.1686
X:88982200	rs5983964	A	G	0.54	2.55E-02	-0.3542	0.1554	5.46E-05	0.8289	0.2132	8.22E-01	0.0361	0.1596
X:88982702	rs2498113	C	G	0.54	2.54E-02	-0.3548	0.1555	5.43E-05	0.8302	0.2134	8.14E-01	0.0377	0.1598
X:88982770	rs62593379	T	C	0.54	2.54E-02	-0.3545	0.1555	5.47E-05	0.8291	0.2132	8.25E-01	0.0355	0.1597
X:88982933	rs111880173	R	D	0.54	2.53E-02	-0.3543	0.1552	5.46E-05	0.8283	0.2131	8.21E-01	0.0362	0.1594
X:88982935	rs115421359	T	A	0.54	2.43E-02	-0.3548	0.1543	5.45E-05	0.8259	0.2125	8.22E-01	0.0358	0.1586
X:88983774	rs113155846	R	D	0.54	2.40E-02	-0.3548	0.1540	5.41E-05	0.8253	0.2124	8.21E-01	0.0360	0.1584
X:88983954	rs5941316	A	C	0.54	2.39E-02	-0.3548	0.1539	5.40E-05	0.8248	0.2122	8.20E-01	0.0361	0.1582
X:88984203	rs2436299	A	C	0.54	2.41E-02	-0.3552	0.1542	5.42E-05	0.8259	0.2125	8.24E-01	0.0354	0.1585
X:88985460	rs60905905	R	D	0.54	2.40E-02	-0.3553	0.1542	5.40E-05	0.8261	0.2125	8.21E-01	0.0360	0.1585
X:88985976	rs62593401	T	C	0.54	2.40E-02	-0.3553	0.1542	5.40E-05	0.8264	0.2126	8.22E-01	0.0359	0.1586
X:88986086	rs56001694	G	A	0.54	2.41E-02	-0.3555	0.1545	5.42E-05	0.8269	0.2127	8.24E-01	0.0354	0.1588
X:88986600	rs143864702	A	C	0.54	2.41E-02	-0.3554	0.1544	5.41E-05	0.8269	0.2128	8.22E-01	0.0359	0.1587
X:88986657	rs116619432	T	G	0.54	2.41E-02	-0.3555	0.1544	5.40E-05	0.8268	0.2127	8.22E-01	0.0358	0.1587
X:88986880	rs377395343	C	G	0.54	2.41E-02	-0.3556	0.1544	5.40E-05	0.8271	0.2128	8.22E-01	0.0359	0.1587

X:88986920	rs371198939	A	C	0.54	2.41E-02	-0.3556	0.1544	5.40E-05	0.8271	0.2128	8.22E-01	0.0359	0.1587
X:88987112	rs139671882	A	C	0.54	2.41E-02	-0.3556	0.1545	5.40E-05	0.8273	0.2128	8.22E-01	0.0359	0.1588
X:88987252	rs2498126	A	C	0.54	2.41E-02	-0.3557	0.1545	5.40E-05	0.8277	0.2129	8.23E-01	0.0358	0.1589
X:88987431	rs5941319	A	G	0.54	2.42E-02	-0.3557	0.1546	5.40E-05	0.8278	0.2130	8.23E-01	0.0358	0.1589
X:88987738	rs5940740	C	T	0.54	2.42E-02	-0.3558	0.1546	5.39E-05	0.8279	0.2130	8.23E-01	0.0358	0.1589
X:88987773	rs5941320	C	T	0.54	2.42E-02	-0.3560	0.1547	5.39E-05	0.8284	0.2131	8.22E-01	0.0359	0.1590
X:88987779	rs5941321	A	G	0.54	2.42E-02	-0.3558	0.1546	5.39E-05	0.8281	0.2130	8.23E-01	0.0358	0.1589
X:88987886	N/A	R	I	0.54	2.42E-02	-0.3558	0.1546	5.39E-05	0.8281	0.2130	8.23E-01	0.0358	0.1590
X:88988448	rs3924952	T	C	0.54	2.42E-02	-0.3560	0.1547	5.39E-05	0.8286	0.2131	8.23E-01	0.0358	0.1591
X:88988567	rs3924951	A	T	0.54	2.43E-02	-0.3563	0.1550	5.40E-05	0.8295	0.2133	8.26E-01	0.0353	0.1593
X:88988691	rs55890612	G	C	0.54	2.43E-02	-0.3566	0.1551	5.36E-05	0.8303	0.2135	8.25E-01	0.0354	0.1594
X:88989009	rs55908407	G	A	0.54	2.44E-02	-0.3569	0.1553	5.38E-05	0.8310	0.2137	8.25E-01	0.0355	0.1596
X:88989019	rs55728255	C	T	0.54	2.44E-02	-0.3568	0.1553	5.38E-05	0.8310	0.2137	8.25E-01	0.0355	0.1596
X:88989265	rs7883216	T	C	0.54	2.44E-02	-0.3569	0.1553	5.36E-05	0.8314	0.2138	8.25E-01	0.0354	0.1597
X:88989941	rs5941323	C	A	0.54	2.45E-02	-0.3570	0.1555	5.38E-05	0.8319	0.2139	8.26E-01	0.0354	0.1598
X:88990017	rs5941324	C	T	0.54	2.45E-02	-0.3570	0.1555	5.37E-05	0.8321	0.2139	8.26E-01	0.0354	0.1598
X:88990045	rs5940741	T	C	0.54	2.45E-02	-0.3570	0.1555	5.37E-05	0.8321	0.2139	8.26E-01	0.0354	0.1599
X:88990257	rs5940742	A	G	0.54	2.46E-02	-0.3572	0.1557	5.39E-05	0.8326	0.2141	8.28E-01	0.0350	0.1600
X:88990444	rs28893821	T	C	0.54	2.45E-02	-0.3571	0.1556	5.39E-05	0.8323	0.2140	8.26E-01	0.0353	0.1599
X:88990476	rs28789747	C	G	0.54	2.45E-02	-0.3571	0.1556	5.39E-05	0.8323	0.2140	8.26E-01	0.0352	0.1599
X:88990742	rs5941326	G	A	0.54	2.45E-02	-0.3576	0.1558	5.45E-05	0.8336	0.2146	8.29E-01	0.0347	0.1602
X:88991056	rs5941327	A	G	0.54	2.45E-02	-0.3574	0.1557	5.38E-05	0.8328	0.2142	8.26E-01	0.0353	0.1600
X:88991175	rs11796443	G	A	0.54	2.46E-02	-0.3574	0.1557	5.39E-05	0.8329	0.2142	8.26E-01	0.0353	0.1601
X:88991396	rs201159284	R	I	0.54	2.46E-02	-0.3574	0.1557	5.39E-05	0.8330	0.2142	8.27E-01	0.0352	0.1601
X:88992089	rs74445676	G	C	0.54	2.46E-02	-0.3575	0.1558	5.40E-05	0.8332	0.2143	8.27E-01	0.0351	0.1602
X:88992104	rs28864731	G	A	0.54	2.46E-02	-0.3576	0.1559	5.39E-05	0.8334	0.2143	8.27E-01	0.0351	0.1602
X:88993137	rs4535915	G	A	0.54	2.46E-02	-0.3577	0.1560	5.39E-05	0.8340	0.2144	8.28E-01	0.0350	0.1603
X:88993140	rs5984384	T	C	0.54	2.46E-02	-0.3578	0.1560	5.39E-05	0.8340	0.2144	8.28E-01	0.0350	0.1603
X:88993274	rs4372165	T	C	0.54	2.47E-02	-0.3577	0.1560	5.39E-05	0.8341	0.2145	8.28E-01	0.0350	0.1604
X:88993676	rs62593418	G	T	0.54	2.48E-02	-0.3578	0.1561	5.39E-05	0.8344	0.2145	8.28E-01	0.0350	0.1605
X:88993851	rs66733457	T	C	0.54	2.48E-02	-0.3577	0.1561	5.39E-05	0.8344	0.2145	8.28E-01	0.0351	0.1605
X:88994011	rs62593419	T	C	0.54	2.48E-02	-0.3578	0.1562	5.39E-05	0.8345	0.2146	8.28E-01	0.0350	0.1605
X:88994303	rs7880736	A	G	0.54	2.50E-02	-0.3586	0.1568	5.15E-05	0.8388	0.2152	8.38E-01	0.0331	0.1611
X:88994388	rs7881978	T	C	0.54	2.48E-02	-0.3579	0.1563	5.40E-05	0.8350	0.2147	8.29E-01	0.0349	0.1606

X:88994909	rs5941328	G	A	0.54	2.48E-02	-0.3582	0.1564	5.39E-05	0.8355	0.2148	8.29E-01	0.0349	0.1607
X:88995097	rs5941329	C	T	0.54	2.48E-02	-0.3582	0.1564	5.39E-05	0.8355	0.2148	8.29E-01	0.0349	0.1607
X:88995217	rs5941330	T	G	0.54	2.48E-02	-0.3582	0.1564	5.40E-05	0.8354	0.2148	8.29E-01	0.0349	0.1607
X:88995324	rs5941331	G	T	0.54	2.48E-02	-0.3583	0.1565	5.40E-05	0.8357	0.2149	8.29E-01	0.0348	0.1608
X:88995964	rs5941332	G	A	0.54	2.49E-02	-0.3583	0.1565	5.41E-05	0.8359	0.2150	8.29E-01	0.0349	0.1609
X:88996012	rs5941333	C	G	0.54	2.49E-02	-0.3584	0.1566	5.40E-05	0.8361	0.2150	8.29E-01	0.0349	0.1609
X:88996245	rs5941334	C	T	0.54	2.49E-02	-0.3585	0.1566	5.40E-05	0.8363	0.2150	8.29E-01	0.0349	0.1609
X:88996463	rs5940743	T	C	0.54	2.49E-02	-0.3585	0.1566	5.41E-05	0.8363	0.2150	8.29E-01	0.0349	0.1610
X:88996799	rs1928406	A	G	0.54	2.50E-02	-0.3585	0.1567	5.40E-05	0.8366	0.2151	8.30E-01	0.0347	0.1610
X:88997222	rs1928404	A	G	0.54	2.50E-02	-0.3585	0.1567	5.40E-05	0.8366	0.2151	8.30E-01	0.0348	0.1610
X:88997273	rs5983967	A	G	0.54	2.50E-02	-0.3585	0.1568	5.42E-05	0.8366	0.2151	8.30E-01	0.0348	0.1611
X:88997343	rs5983968	T	C	0.56	8.26E-03	-0.4232	0.1556	3.10E-05	0.8936	0.2250	4.73E-01	0.1170	0.1620
X:88997418	rs200284748	R	I	0.54	2.51E-02	-0.3586	0.1569	5.42E-05	0.8371	0.2152	8.31E-01	0.0346	0.1612
X:88997470	rs5941335	A	G	0.54	2.50E-02	-0.3586	0.1568	5.42E-05	0.8370	0.2152	8.30E-01	0.0348	0.1611
X:88997856	rs5940744	T	C	0.54	2.50E-02	-0.3588	0.1569	5.40E-05	0.8374	0.2153	8.30E-01	0.0347	0.1612
X:88998033	rs113583265	C	G	0.54	2.51E-02	-0.3590	0.1570	5.42E-05	0.8379	0.2154	8.31E-01	0.0346	0.1613
X:88998095	rs62593441	T	C	0.54	2.51E-02	-0.3588	0.1570	5.41E-05	0.8376	0.2154	8.31E-01	0.0347	0.1613
X:88998237	rs12850391	A	G	0.54	2.51E-02	-0.3589	0.1570	5.40E-05	0.8379	0.2154	8.31E-01	0.0346	0.1613
X:88998562	rs55875767	T	A	0.54	2.51E-02	-0.3595	0.1573	5.44E-05	0.8379	0.2155	8.35E-01	0.0338	0.1616
X:89000953	rs140787096	C	T	0.54	2.53E-02	-0.3602	0.1577	5.58E-05	0.8373	0.2155	8.33E-01	0.0342	0.1621
X:89003061	rs5984389	C	T	0.54	2.65E-02	-0.3593	0.1588	5.69E-05	0.8401	0.2164	8.40E-01	0.0331	0.1630
X:89003666	rs5983969	T	A	0.54	2.55E-02	-0.3607	0.1583	5.63E-05	0.8389	0.2160	8.35E-01	0.0340	0.1626
X:89004443	rs1928402	A	G	0.54	2.57E-02	-0.3598	0.1581	5.61E-05	0.8390	0.2160	8.29E-01	0.0353	0.1624
X:89005266	rs5984392	G	A	0.54	2.57E-02	-0.3609	0.1585	5.65E-05	0.8400	0.2163	8.37E-01	0.0337	0.1628
X:89006238	rs5983970	C	A	0.54	2.56E-02	-0.3612	0.1586	5.62E-05	0.8404	0.2163	8.34E-01	0.0342	0.1629
X:89006765	rs201752520	R	D	0.54	2.57E-02	-0.3611	0.1586	5.65E-05	0.8404	0.2164	8.35E-01	0.0340	0.1629
X:89007318	rs4086250	T	C	0.54	2.57E-02	-0.3611	0.1587	5.64E-05	0.8406	0.2164	8.35E-01	0.0341	0.1630
X:89007999	rs150430625	C	T	0.54	2.58E-02	-0.3613	0.1588	5.66E-05	0.8411	0.2166	8.36E-01	0.0340	0.1631
X:89008346	rs62593443	C	T	0.54	2.58E-02	-0.3613	0.1588	5.66E-05	0.8411	0.2166	8.36E-01	0.0339	0.1631
X:89008400	rs2771605	G	T	0.54	2.58E-02	-0.3614	0.1589	5.66E-05	0.8412	0.2166	8.37E-01	0.0338	0.1632
X:89008626	rs2771612	A	G	0.54	2.60E-02	-0.3606	0.1587	5.64E-05	0.8411	0.2165	8.30E-01	0.0352	0.1630
X:89010519	rs5984397	G	T	0.55	2.53E-02	-0.3662	0.1604	5.71E-05	0.8519	0.2203	8.63E-01	0.0286	0.1648
X:89010521	rs5984398	A	G	0.55	2.53E-02	-0.3664	0.1605	5.68E-05	0.8522	0.2203	8.62E-01	0.0287	0.1649
X:89010553	rs5984399	C	G	0.54	2.71E-02	-0.3602	0.1598	5.64E-05	0.8456	0.2177	8.45E-01	0.0323	0.1640

X:89011086	rs2771673	G	T	0.54	2.70E-02	-0.3599	0.1596	6.07E-05	0.8412	0.2174	8.40E-01	0.0332	0.1638
X:89011719	rs5941339	T	A	0.54	2.69E-02	-0.3593	0.1591	6.12E-05	0.8396	0.2172	8.32E-01	0.0348	0.1633
X:89013049	rs5940747	C	T	0.54	2.69E-02	-0.3603	0.1596	6.15E-05	0.8405	0.2173	8.28E-01	0.0357	0.1638
X:89013973	rs5941342	A	C	0.54	2.67E-02	-0.3608	0.1597	6.15E-05	0.8411	0.2175	8.31E-01	0.0352	0.1639
X:89014091	rs5941343	C	T	0.54	2.68E-02	-0.3606	0.1597	6.18E-05	0.8408	0.2175	8.31E-01	0.0352	0.1639
X:89015450	rs5940748	T	G	0.54	2.78E-02	-0.3608	0.1609	6.28E-05	0.8437	0.2183	8.38E-01	0.0339	0.1650
X:89016167	rs5941348	T	C	0.54	2.79E-02	-0.3610	0.1609	6.29E-05	0.8440	0.2184	8.38E-01	0.0339	0.1651
X:89016282	rs5941349	T	C	0.54	2.79E-02	-0.3609	0.1610	6.28E-05	0.8441	0.2184	8.38E-01	0.0339	0.1651
X:89016747	rs11796768	C	T	0.54	2.81E-02	-0.3610	0.1612	6.38E-05	0.8437	0.2185	8.43E-01	0.0328	0.1654
X:89017511	rs62590968	A	G	0.54	2.81E-02	-0.3610	0.1613	6.40E-05	0.8438	0.2185	8.43E-01	0.0329	0.1654
X:89017626	rs5940749	T	A	0.54	2.84E-02	-0.3607	0.1614	6.53E-05	0.8439	0.2185	8.47E-01	0.0321	0.1655
X:89017645	rs5941353	G	A	0.54	2.85E-02	-0.3602	0.1614	6.58E-05	0.8435	0.2185	8.47E-01	0.0322	0.1655
